# Target-based evaluation of ‘drug-like’ properties and ligand efficiencies

**DOI:** 10.1021/acs.jmedchem.1c00416

**Published:** 2021-05-13

**Authors:** Paul D. Leeson, A. Patricia Bento, Anna Gaulton, Anne Hersey, Emma J. Manners, Chris J. Radoux, Andrew R. Leach

**Affiliations:** 1 Paul Leeson Consulting Ltd, The Malt House, Main Street, Congerstone, Nuneaton, Warkwickshire CV13 6LZ, UK; 2 European Molecular Biology Laboratory, European Bioinformatics Institute, Wellcome Genome Campus, Hinxton, Cambridgeshire CB10 1SD, UK

## Abstract

Physicochemical descriptors commonly used to define ‘drug-likeness’, and ligand efficiency measures, are assessed for their ability to differentiate marketed drugs from compounds reported to bind to their efficacious target or targets. Using ChEMBL version 26, a dataset of 643 drugs acting on 271 targets was assembled, comprising 1104 drug-target pairs having ≥ 100 published compounds per target. Taking into account changes in their physicochemical properties over time, drugs are analysed according to target class, therapy area and route of administration. Recent drugs, approved in 2010-2020, display no overall differences in molecular weight, lipophilicity, hydrogen bonding or polar surface area from their target comparator compounds. Drugs are differentiated from target comparators by higher potency, ligand efficiency (LE), lipophilic ligand efficiency (LLE), and lower carboaromaticity. Overall, 96% of drugs have LE or LLE values, or both, greater than the median values of their target comparator compounds.

## Introduction

The physicochemical properties of small molecule drugs and discovery compounds, such as lipophilicity, size, hydrogen-bonding and ionisation state, broadly influence their absorption, distribution, metabolism, elimination and toxicity (ADMET) profiles, in particular their permeability, solubility, and metabolic clearance.^1–5^ ‘Drug-likeness’ is often said to be defined by the ranges of physicochemical properties possessed by marketed drugs. The rule of 5 (Ro5), introduced in 1997, is often cited as a ‘drug-like’ rule, though its intention was primarily to address poor solubility and permeability of oral drugs, by keeping the molecular weight (MW) <500, lipophilicity (cLogP) <5, the H-bond donor count (sum of OH+NH groups) <5 and the H-bond acceptor count (sum of O+N atoms) <10.^6,7^ The Ro5 stimulated numerous further investigations into physicochemical properties and compound quality guidance, including the use of aromaticity and shape descriptors,^8,9^ and ligand efficiency measures.^10,11^ Most importantly, the Ro5 has elevated the awareness of the drug discovery community to the need for property-based design, leading to deeper understanding of the compromises encountered in balancing the key property-associated parameters of potency, permeability and metabolic clearance.

It has become clear that applying ‘drug-like’ properties in a generic manner can be questioned, for three main reasons. Firstly and most obviously, physicochemical descriptors themselves are surrogates for compound quality and are no substitute for robust experimental data.^12,13^ Secondly, the physical properties of drugs^14^ as well as discovery compounds^15,16^ vary according to their biological target (as well as target accessibility in vivo), observations that are important for the future as the number of new targets pursued in drug discovery efforts is significantly expanding.^17,18^ Thirdly, some of these physicochemical descriptors, notably MW, structural complexity and polar surface area (PSA), are increasing significantly over time in marketed drugs,^19–22^ and in discovery and patented compounds,^23–26^ going into chemical space beyond the boundaries of the Ro5 (termed bRo5).^27–30^ Collectively these observations question the existence of a fixed ‘drug-like’ physicochemical space.

In an earlier study using a limited set of 25 biological targets, most drugs acting at these targets possessed increased values of both ligand efficiency (LE = p(Activity) x 1.37/ heavy atom (HA) count) and lipophilic ligand efficiency (LLE = p(Activity) – LogP or D) versus other reported molecules acting at the same targets.^11^ In addition, successful lead optimisation campaigns across 15 targets often resulted in increased LE and LLE values.^31^ The goal of this study is to exhaustively and critically expand on these observations. We assess a selection of commonly used physicochemical properties and ligand efficiency metrics, by comparing their values for marketed drugs with the cohort of published compounds acting at the target or targets linked to the drugs’ efficacy, taking into account the progression in drug properties over time.

## Data assembly

### Sources

An exhaustive analysis of the publicly available ChEMBL database^32^ (version 26), was conducted. Each small molecule marketed drug in ChEMBL, defined as reaching phase IV, is annotated to the specific biological molecular target(s) believed to be responsible for the drug’s therapeutic efficacy. In some cases the assigned molecular target is a protein family or complex. Source information for drug-target mapping includes journal articles and regulatory documents (cited in ChEMBL). A small number of drugs where a therapeutic target was not assigned in ChEMBL were manually curated. Biological drugs (proteins, antibodies, etc) are similarly annotated in ChEMBL but were excluded from this study. In all, there were 2028 small molecule drugs with calculated lipophilicity (ALogP values) and MW ≤1000, of which approximately 70% had a protein target. Following further curation (see below) a total of 686 drugs (MW range 94 – 994) were identified where there was a potency value, designated pChEMBL, in an assay at the drug’s therapeutic target(s) annotated by ChEMBL as a binding assay, together with at least one other comparator molecule acting at the same target(s). Biological assays annotated by ChEMBL as functional or phenotypic were not used, and there was no differentiation made according to mode of action (agonists, antagonists or modulators).

For drugs and comparator compounds with more than one pChEMBL value at a specific target, median values were computed and used in the subsequent analyses. In curating these data, prodrugs, or drugs which are known to be transformed to pharmacologically active compounds, were removed and replaced by their active metabolites where pChEMBL data were available (a total of 38 drugs, see Supporting Spreadsheet S1). The route of administration of the parent drug was used for the active metabolites. A significant proportion of mutant protein activity data was found for kinase targets and additional filters were applied to extract potency data against the wild-type kinase enzymes and to exclude assays performed with a curated mutant. For other target classes data against the wild-type and any mutant proteins were combined. For three targets where the drugs are known to be optimised for a mutant protein (V600E B-raf kinase, R132H isocitrate dehydrogenase 1 and T790M EGRF kinase), the mutant target comparative compound data were used. Additional routes of administration (106 drugs) and year of first approval (76 drugs) were manually curated where this information was not present in ChEMBL (see Supporting Spreadsheet S1; approval dates are typically FDA approvals, with global first approvals for a handful of manually curated drugs). Therapeutic areas were assigned using the Anatomical Therapeutic Chemical (ATC) Classification and Medical Subject Headings (MeSH).^33^ The biological targets were assigned to broad target classes, with G-protein receptors being subdivided according to their ligands as aminergic, peptidergic and other. Any drugs acting on targets from more than one target class were assigned to the target class most commonly indicative of their therapeutic use. Oral drug maximum daily doses were annotated from literature sources with some additions from online labelling information as described earlier.^34^

### Drug and target comparator compound dataset

We required the number of target comparator compounds for each drug to be ≥100 (range 101-7582, 360,049 compounds in total), resulting in a drug and comparator compound dataset of 643 drugs acting on 271 targets. Of the 271 biological targets, 259 are human, 9 are viral, 2 are bacterial and one is a parasite target. Of the 643 drugs, 426 had a single target and 217 had more than one target assigned, resulting in 1104 unique drug-target pairs (see Supporting Spreadsheet S1 for the full list of the biological targets and associated counts of drugs and comparator compounds). The set of comparator compounds reflects typical drug discovery and lead optimisation compounds as reported in the medicinal chemical literature and subsequently curated into ChEMBL.Primary drug-target pairs are classified by the annotated therapeutic target where the drug has the highest pChEMBL value. The remainder are classified as secondary drug-target pairs; 77 of the 271 targets were secondary targets only. To avoid duplication of drug properties, primary drug-target pairs (643 drugs and 194 targets) are predominantly used here, except for target class analyses where all drug-target pairs (n=1104) are used. We analyse commonly employed properties and descriptors listed in Table 1. Simple physicochemical properties were calculated using RDKit^35^ and are available in the ChEMBL database. LogD_7.4_ was calculated using the Chemaxon software and other properties such as number of stereocenters and Fsp3 were calculated using algorithms available in the BIOVIA pipeline pilot chemistry collection.^35^ Calculations and visualisation were done using DataWarrior^36^ and Microsoft Excel. The full dataset for the 1104 drug-target pairs is provided in Supporting Spreadsheet S1.

The properties and descriptors in Table 1 are analysed in this paper in three groups as defined in Box 1.

### Accounting for the influence of time on drug properties

**Figure 1:**
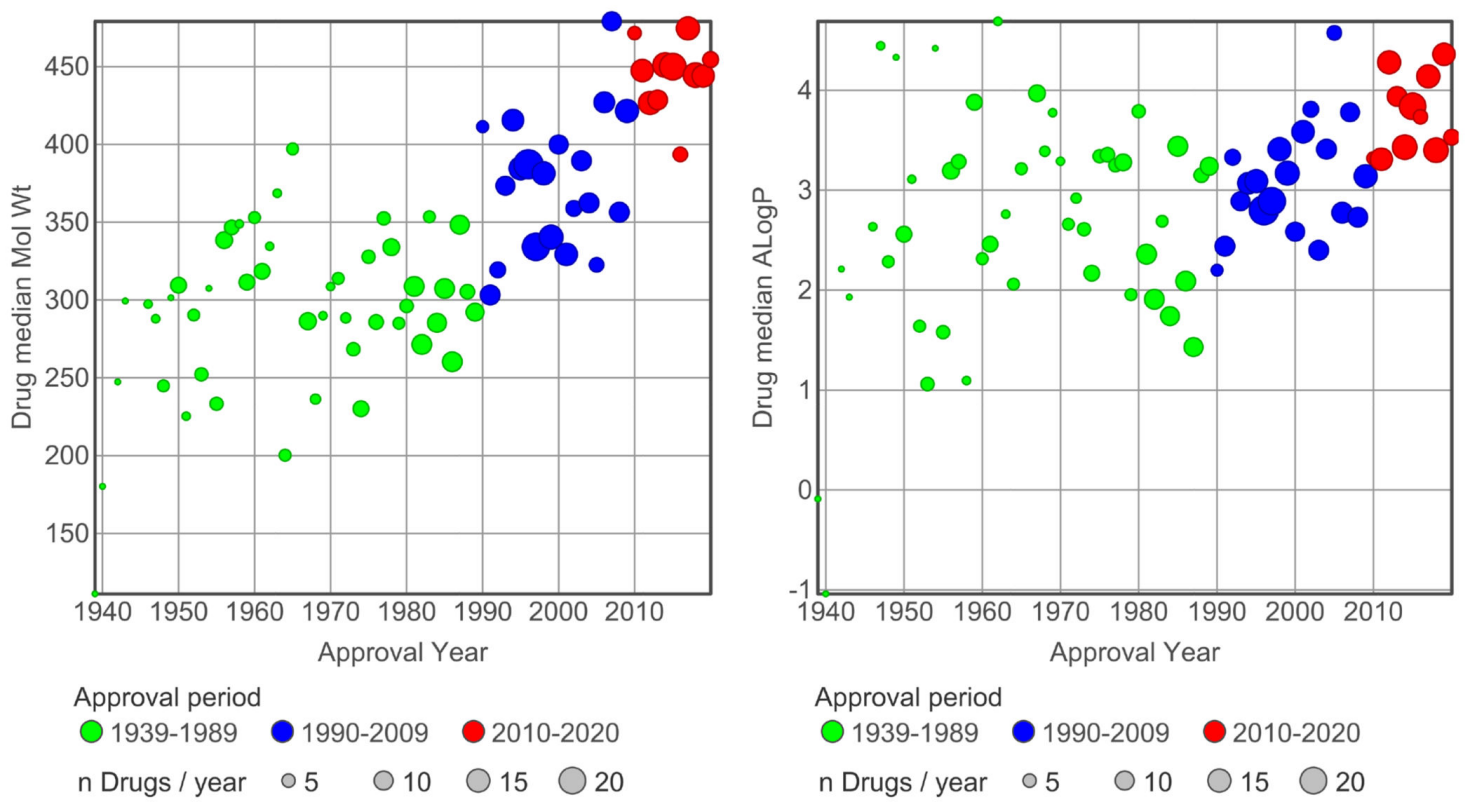
Three drug approval time periods used in this paper were selected on basis of the increases in molecular weight and lipophilicity (ALogP) seen in the 643 drugs with ≥ 100 comparator compounds acting at their target(s). The differences in molecular weight and ALogP between the three approval periods are statistically significant (*t*-test p <0.05). Mean and median values for the three time periods are given in Table 3.

The drugs had approval dates ranging from 1939 to 2020. Based on the progression over approval time of drug MW and lipophilicity (ALogP, Figure 1), the drug set was split into three broad time frames, 1939-1989, 1990-2009 and 2010-2020. For a few drugs, approval dates could not be found; based on their first publication dates in Scifinder^®^, these drugs were assigned to the 1939-1989 group. The observed time-related increases in MW and ALogP for this data set are consistent with previous reports on both drugs and literature compounds, showing MW is increasing at a greater rate than ALogP.^19–21,23,30^ There is however a notable increase in ALogP in 2010-2020 drugs (Figure 1), which is also observed for LogD_7.4_ (see Table 3).

The assigned time frame of drug-target pairs refers to the period in which the drug was approved. Comparator compounds from ChEMBL comprise those with reported binding affinities for the drug targets and for practical reasons they were not time-stamped. Because assays for cloned human targets did not begin to become available until the mid to late 1980s, the pChEMBL values used here for drugs and targets in the earliest 1939-1989 approval time frame post-date the discovery of these drugs, which would have occurred several years prior to the approval dates. Contemporaneous comparator compounds for the older drug set will almost certainly have lower values of MW and ALogP. Evidence for this comes from the increases over time in physical properties observed in published compounds from the *Journal of Medicinal Chemistry*, which mirror the changes seen in drugs, showing median MW increased by 94 Daltons from the 1960s to the 2000s.^23^ The drugs we use from these periods have similar median MWs to their contemporary published compounds from the *Journal of Medicinal Chemistry* (Table 2). As expected, the median MW for target comparator compounds from ChEMBL is also similar in both time frames, but is higher than for drugs in the 1960s (Table 2). For these reasons the older drugs might have been excluded from this study altogether, but we decided to retain them for completeness and for comparison with newer drugs.

Changes to the physicochemical profile of drugs and research compounds over the time frame of this study have been accompanied by major advances in drug discovery and development science, processes and standards. Many drugs in the early approval period were invented in the absence of specific knowledge of the molecular target, often by using primary functional assays in animal tissues, driven by endogenous agonists. Differential tissue activity between compounds^37^ was typically sought to define activity at hypothesised receptor subtypes, as exemplified by the classic examples of beta 2 receptor and histamine H2 antagonists,^38^ discoveries which illustrated fundamental concepts of receptor theory and drug action. In vivo efficacy screening was commonly applied, while ADME assays were rarely employed at the discovery stage. Drugs approved in 1990-2009 would have seen a transition of primary assays from predominantly animal-based to predominantly human, and the introduction of ADME optimisation. Those approved in the 2010-2020 period would be impacted further by the widespread adoption from the late 1990s onwards of high throughput screens of human targets, structure-based drug design, predictive computational chemistry, and multiparameter optimisation. Target and pharmacology based discovery dominated FDA approvals in the period 1999-2013, with only 8 of 113 first in class drugs discovered using phenotypic assays.^39^

### Caveats

Some limitations accompanying the dataset should be noted. Drugs are assigned to specific therapeutic targets on the basis of current knowledge, and these assignments could change in future as more mechanistic understanding is obtained. For example, some clinical anticancer drugs do not act via their intended mechanisms.^40^ As well as drugs with fewer than 100 comparator compounds, those lacking annotated therapeutic targets or having published potencies not abstracted by ChEMBL version 26, are absent. pChEMBL binding values for an individual target include all different assay types (e.g. IC_50_, Kd, Ki and other values) and for practical reasons, no account is taken of differences in assay protocols. There is variability seen in reported pChEMBL values for a compound, with pIC_50_ differences between assays generally within one log unit.^41^ For practical reasons, only binding potencies are used, taking no account of mode of action.

The target comparator cohort of compounds depends entirely on authors’ decisions on what to publish. There is a likelihood of unintended bias, because compounds appearing in the literature would be selected by authors specifically to exemplify their observations and discoveries, and these selections may not always be fully representative of all the discovery activities undertaken. For example weakly active compounds, and those with poor ADME properties, may, unsurprisingly, have been omitted from some publications. However, it is reassuring that the trends seen in the results of this work show no significant dependency on the size of the target comparator cohort (101 - 7582 compounds).

## Results and discussion

### Impact of time on targets, target class, therapy areas and route of administration

**Figure 2:**
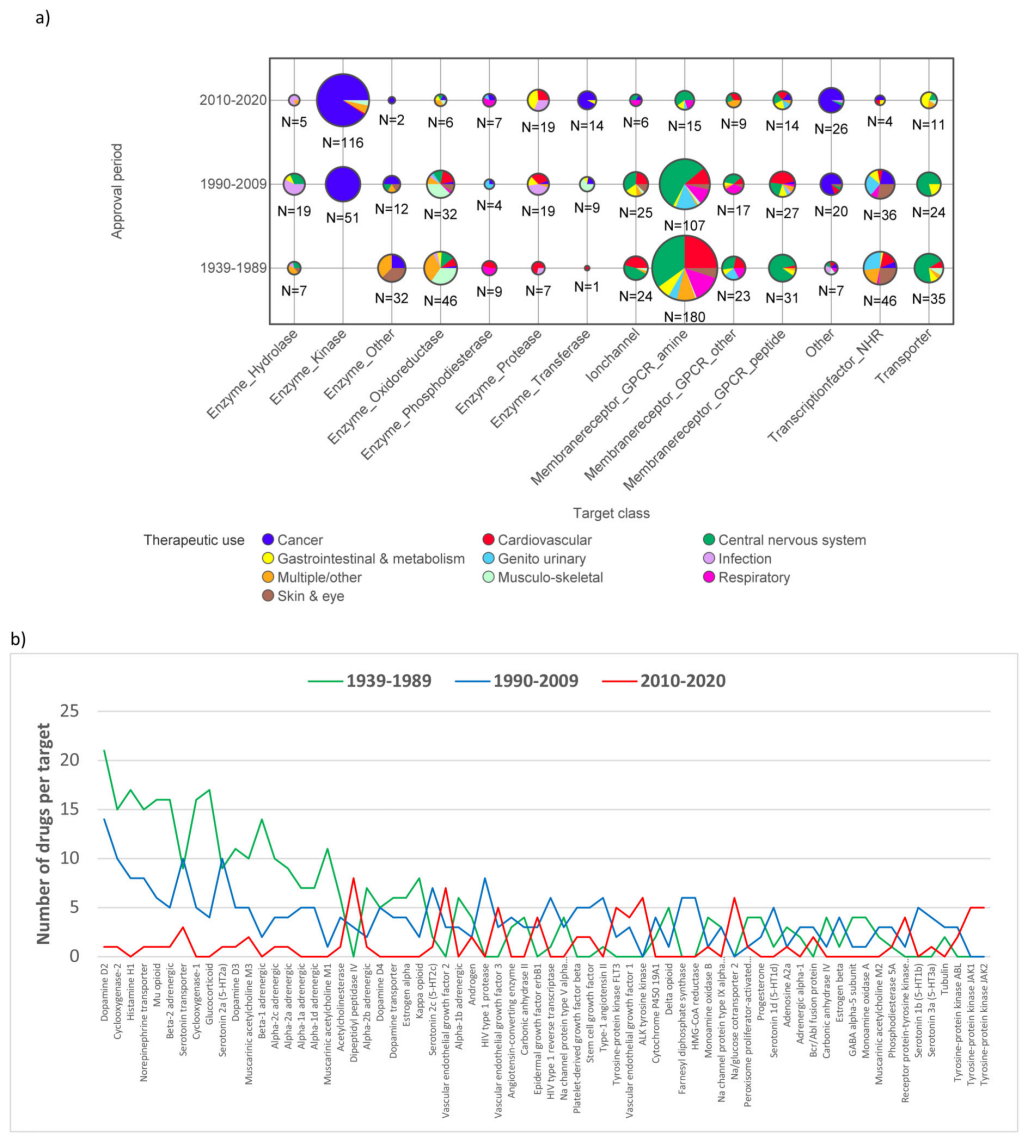
Distribution of drug-target pairs from CHEMBL with ≥ 100 comparator compounds (n=1104) acting at the target(s) linked to the drug’s therapeutic action, in the three selected drug approval time periods. a) Target class and therapeutic use. b) Number of drugs per target in each time frame, for all targets with a total of ≥ 5 drugs. In all there are 643 drugs acting at 271 targets, of which 426 have a single defined target; 117 have 2 targets; 47 have 3 targets; 29 have 4 targets; and 24 have 5 or more targets. All targets and associated data used for this study are provided in the Supporting Spreadsheet S1.

The division of the full set of 1104 primary and secondary drug-target pairs by target class, drug approval time period and therapeutic use are shown in Figure 2a. As well as changes in MW and ALogP of the drugs (Figure 1), major shifts over time in the target classes pursued are evident, from predominantly aminergic G-protein coupled receptors in 1939-1989 to predominantly kinases in 2010-2020. This is linked to changes in therapy area focus, from largely cardiovascular and central nervous system (CNS) drugs in 1939-1989 to cancer drugs in 2010-2020. Moreover, the distribution of 1939-1989 drugs and, to a lesser extent the 1990-2009 drugs, are skewed by heavily pursued historical targets, such as the dopamine D2 receptor, adrenergic receptor subtypes, cyclooxygenases, the mu opioid receptor, the corticosteroid receptor and aminergic transporters, which are targets for a minority of 2010-2020 drugs (Figure 2b). Overall there are fewer drug-target pairs per target in 2010-2020 (1.9) than in 1990-2009 (2.5) and 1939-1989 (3.6).

**Figure 3:**
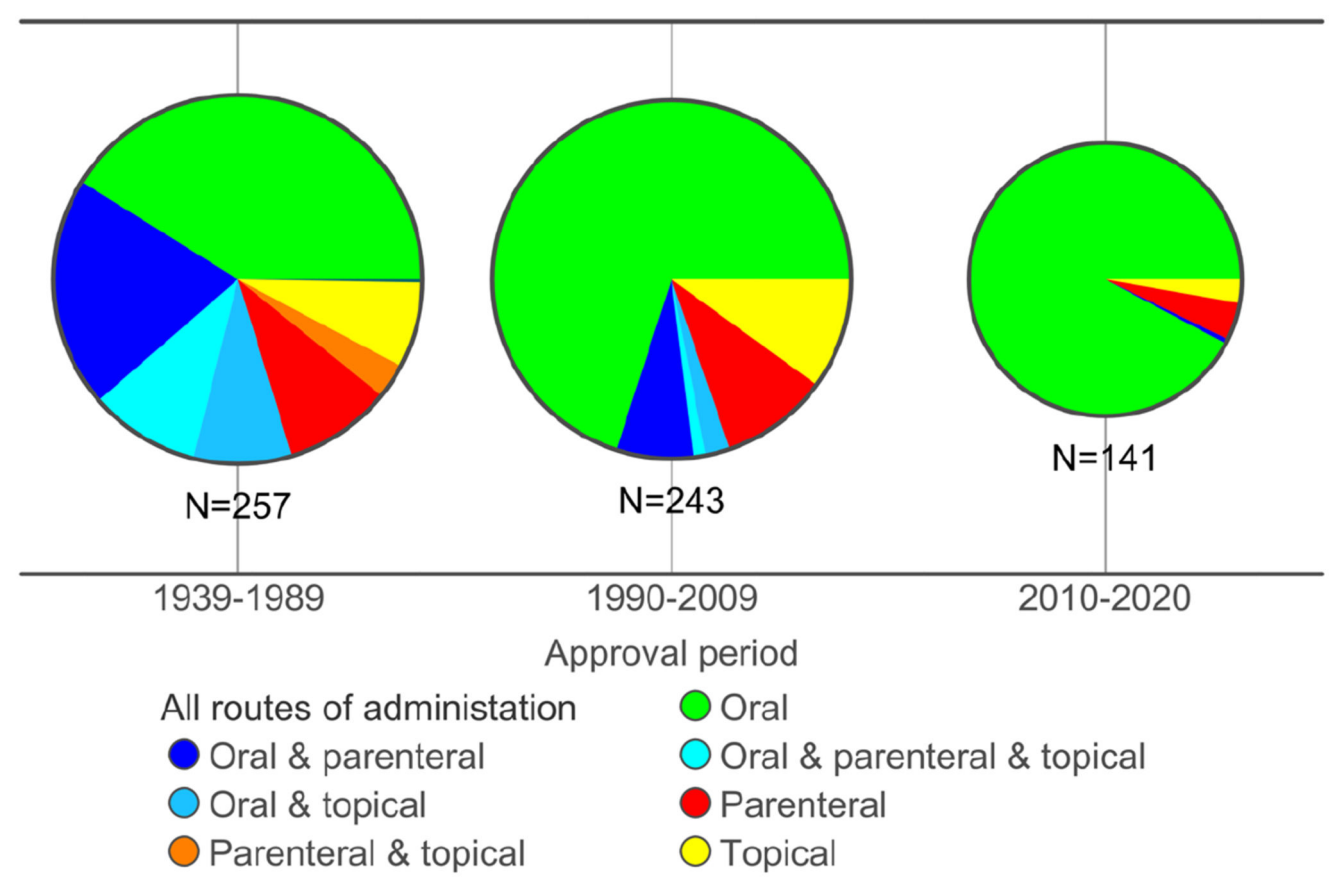
Routes of administration of drugs with ≥ 100 comparator compounds acting at their target(s). The numbers of drugs that can be given non-orally in the three time periods is 150 (58%) in 1939-1989, 73 (30%) in 1990-2009 and 11 (8%) in 2010-2020.

The drugs are given by oral, topical and parenteral routes of administration and in many cases by more than one route. Oral drugs dominate overall, but over time the proportion of drugs that can be used by topical and parenteral routes has decreased (Figure 3), from 58% in 1939-1989 drugs to 8% in 2010-2020. Increased targeting to the oral route over time may account in part for this observation. Additionally, older drugs have had more time in clinical use for exploration of various routes of administration. The increase in lipophilicity over time (Figure 1) is also likely to be a factor limiting the aqueous solubility required for preparation of solution formulations for parenteral administration.

### Properties of drugs versus target comparator compounds

Tables 3–6 contain mean and median properties of drugs, their target median comparators and the [drug - target median] differences for the properties examined, split by the three designated time periods. To allow direct comparisons of the impact of all properties in differentiating drugs from their comparator target compounds, the counts of drugs with properties either higher, lower, or equal to the median value of the target compounds are included in Tables 3–6, together with the correlation coefficients (r values) between drug and target medians. Increasing values of the drug-like ratios in Tables 3–6 reflect increasing probability that drug properties are more ‘drug-like’ than their corresponding target median properties. For example, in comparison with target values, drugs with lower ALogP and MW, and higher pChEMBL or ligand efficiencies, are considered more ‘drug-like.’ For physical properties comprising only low integer counts, such as hydrogen bond donors and acceptors, aromatic ring count and stereocenter count, there are significant numbers of drugs where their properties are the same as the target median.

**Figure 4:**
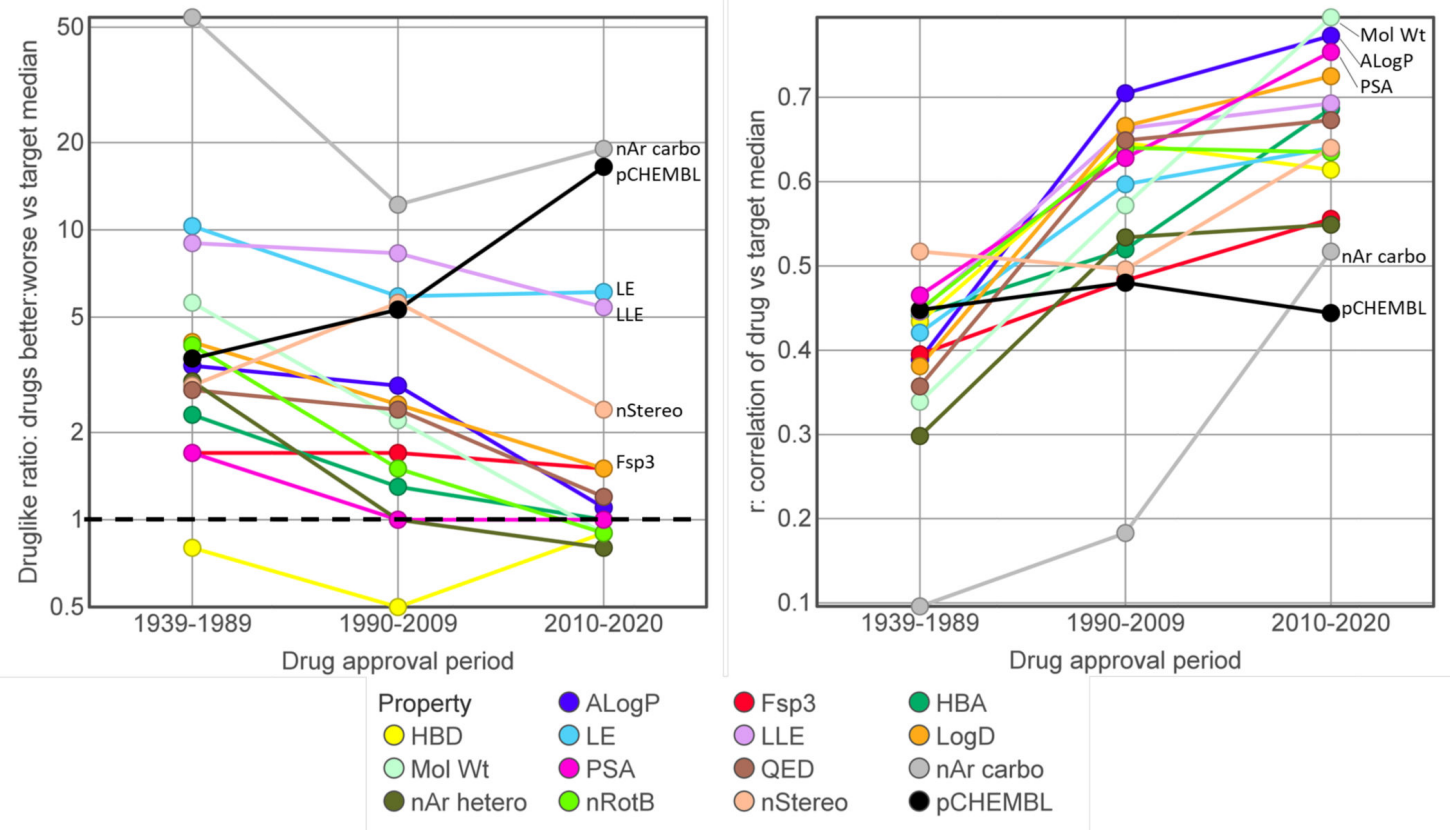
a) The drug-like ratio, the ratio of numbers of drugs with ‘more drug-like’ (better) to ‘less drug-like’ (worse) properties over the three time periods for selected key parameters. b) Drug versus target median correlation coefficients (r values) for the same data. All values of the datapoints in a) and b) are from Tables 3–6.

The progression over time of drug-like ratios and drug versus target r values for selected key properties are summarised in Figure 4. Figure 4 shows an important finding of this study, that overall [drug - target median compound] property differentiation is narrowing consistently and substantially over the time periods. Correspondingly, the correlations between drugs and target medians are increasing over time. In the 2010-2020 drugs, the drug versus target correlations are highest for the primary physicochemical properties, namely MW, ALogP and polar surface area (PSA) (Figure 4b). Of particular interest are those properties that significantly distinguish the most recent drugs (2010-2020) from their target comparators, and so are least resistant to diminishing over time. In order of their drug-like ratios (Figure 4a), these are pChEMBL and carboaromatic ring count;^42^ LE^43^ and LLE;^44^ the number of stereocenters; and Fsp3 (the fraction of carbon atoms that are sp3 hybridised).^45,46^ In each drug approval period, [drug - target median] property differences are largely independent of the corresponding target median values (see Supporting Figure S1 for MW and ALogP analyses).

**Figure 5:**
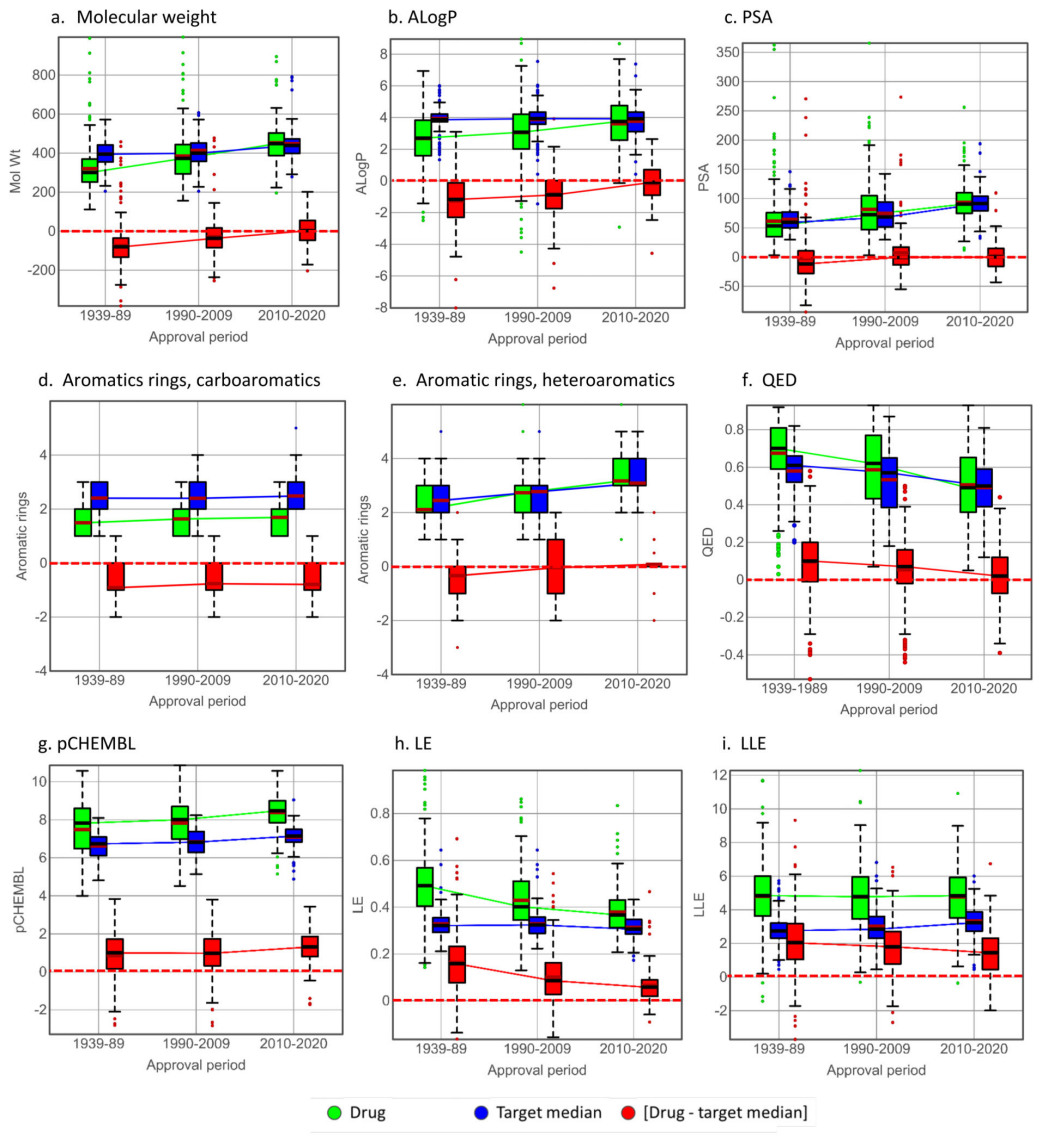
Box plots showing selected drug, target median and [drug - target median] properties in the three time periods for drug primary targets. The time periods are connected by median values, except for aromatic ring counts, where means are used. Mean and median values for all properties are in Tables 3–6, statistical values are in Supporting Spreadsheet 2.

**Figure 6:**
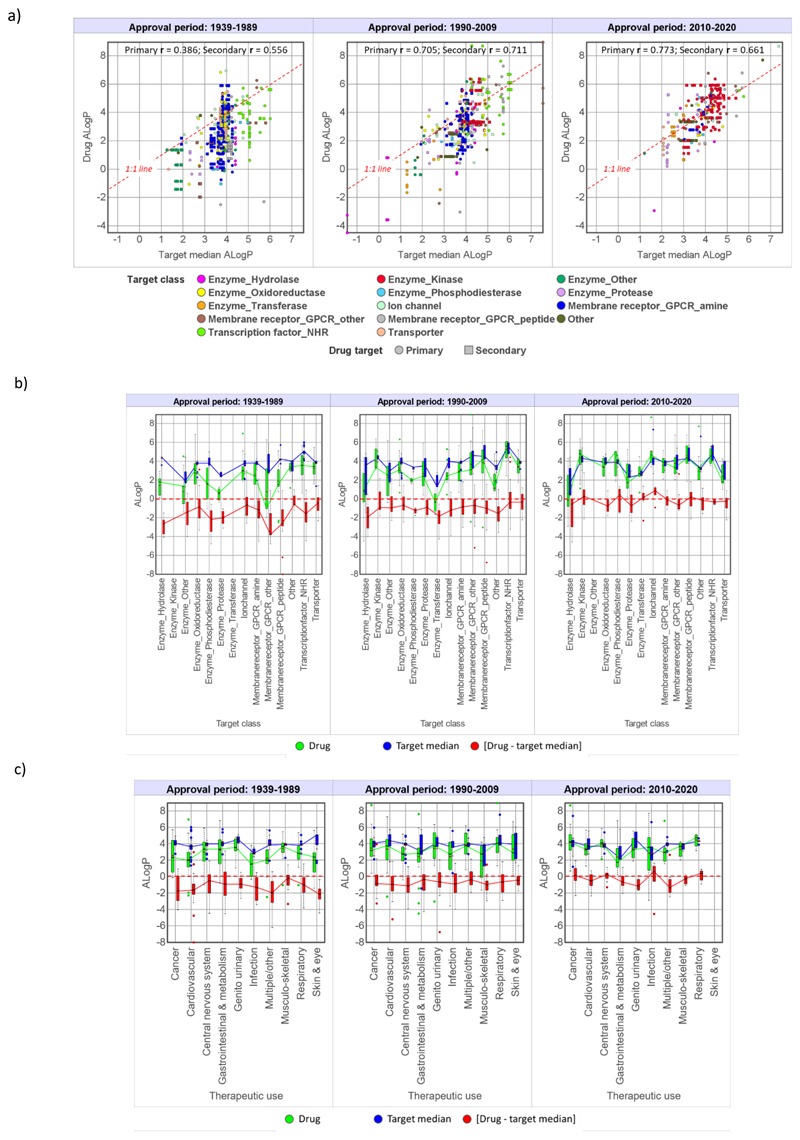
Drug and target comparator ALogP values by drug approval time period. a) Drug versus corresponding target medians. b) The data in a) split by target class. c) Therapeutic use. Statistical values for boxplots b) and c) are in the Supporting Spreadsheet S2.

**Figure 7:**
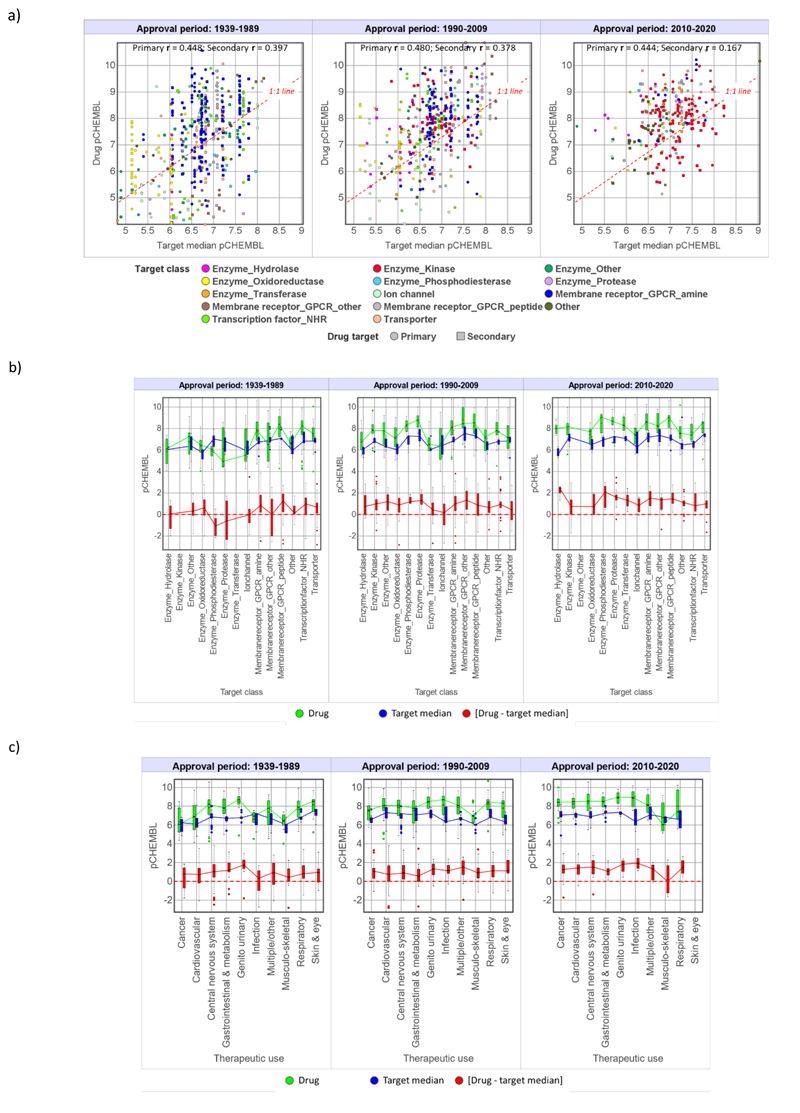
Drug and target comparator pCHEMBL values by drug approval time period. a) Drug versus corresponding target medians. b) The data in a) split by target class. c) Therapeutic use. Statistical values for boxplots b) and c) are in the Supporting Spreadsheet S2.

A selection of property versus time boxplots for drugs, target comparators and [drug - target median] differences are illustrated in Figure 5 and Supporting Figure S2. In addition, analysis of each property was performed using plots of drug versus target medians for each time period, and the corresponding boxplots showing drug, target comparator and [drug - target median] difference by target class and therapeutic use. Representative examples are shown for ALogP (Figure 6), pChEMBL (Figure 7) and MW, QED (the quantitative estimate of drug-likeness),^47^ LE and LLE (Supporting Figures S3–S6). For the physicochemical properties examined, a general observation, which follows from the drug versus target comparator correlations, is that drug and their target comparator properties tend to track each other, showing similar variation by target class and therapy area.

### Size, lipophilicity and polarity

With both size (MW and HA count) and lipophilicity (ALogP and LogD_7.4_), [drug - target median] differences reduce over time and the 2010-2020 drugs have the same median values as their target comparators (Table 3, Figure 5a and 5b, Supporting Figure S2a and S2b). Drug - target median] differences are slightly lower in all time frames for LogD_7.4_ versus ALogP (Table 3). This is probably a result of some ionised drugs having lower LogD_7.4_ values than neutral target comparator molecules, and for this reason the ALogP [drug - target median] values better reflect intrinsic differences in lipophilicity. With MW (and HA count with which is it strongly correlated - r=0.981 for the drugs), the increase over time for the drug sets dominate, but the corresponding target comparators also show a small but significant increase in 2010-2020 (median 439) versus 1990-2009 (median 399). With ALogP, target comparator median values are constant over time, with the increased lipophilicity of the 2010-2020 drugs being equal to their target compounds. For both ALogP (Figure 6) and MW (Supporting Figure S3), the increased correlation between drug and target comparators over time results in convergence of target class and therapeutic area differences in the 2010-2020 drugs. For example, drugs acting in the central nervous system^48,49^ have lower MW than anti-infective compounds,^50,51^ as expected, and their corresponding target comparators also show the same differences (Supporting Figure S3). Similarly, the difference in lipophilicity between kinase inhibitors and protease inhibitors in the 2010-2020 time frame is reflected by their corresponding targets (Figure 6).

Hydrogen bond donors (HBD) have higher values in drugs versus target medians for the 1939-1989 and 1990-2009 drugs, but there is no difference between drug and target in 2010-2020 drugs (Table 3, Supporting Figure S2d). Hydrogen bond acceptors (HBA) differ between drugs and target medians only in 1939-1989 drugs (Supporting Figure S2c). Polar surface area (PSA) is increasing equally over time in both drugs and their target comparators and this property does not significantly distinguish drugs from target compounds in any time period (Table 3 and Figure 5c). The flat PSA profile over time follows from the well-known fact that lipophilicity increases with size (MW) and decreases with polarity and hydrogen bonding (PSA).^52^ Hence PSA depends on [size (MW)] minus [lipophilicity (ALogP)]. Because MW and ALogP show very similar time profiles (Figures 5a and 5b), the difference between them (i.e. polarity) is small.

### Aromatic and aliphatic properties

Carboaromatic compounds are reported to carry greater developability risks (based on solubility, protein binding, P450 inhibition and hERG binding) than heteroaromatic compounds, a likely consequence of higher lipophilicity.^42^ Over time, the proportions of both carboaromatic drugs (having zero aromatic heteroatoms) and aliphatic drugs in this study have decreased, while heteroaromatic drugs (having ≥ 1 aromatic heteroatom) have increased, the latter dominating (76%) the 2010-2020 drugs (Table 7). There are strikingly fewer carboaromatic drugs with aromatic ring count ≥ 3 than heteroaromatic drugs, which is consistent with earlier studies.^42^ (Table 7 and Supporting Figure S7).

Drugs containing the steroid nucleus, acting at androgen, glucocorticoid or progesterone receptors, are historically significant and account for 22 of the 43 aliphatic drugs in the 1939-1989 approval set. In the 2010-2020 drugs, 55% have ≥ 3 aromatic rings, accounting for much of the overall increase in drug aromatic ring count over time (Table 4). In the carboaromatic drugs, target comparators have higher aromatic ring count than drugs, independently of time (Figure 5d), with very few drugs having aromatic ring count higher than the target median (Table 4). In contrast, both drug and target compound aromatic ring counts are increasing in heteroaromatics, with the [drug - target median] difference remaining negligible over time (Figure 5e). Higher aromatic ring count is seen in both drugs and target comparators with heteroaromatics versus carboaromatics in the 2010-2020 time frame. Increasing aromatic ring count is associated with increased lipophilicity,^9^ and indeed the ALogP trends follow the aromatic ring count observations for the carboaromatic and heteroaromatic drugs, with the carboaromatics notably showing a flat ALogP profile over time (see Supporting Figure S8).

The parameter Fsp3, the fraction of carbon atoms in a molecule that are sp3 hybridised, is widely used as an approximate estimate of 3-dimensionality, and has been linked to aqueous solubility.^45,46^ It should be noted that Fsp3 is correlated negatively with nAr,^9,21^ and high nAr is also linked to low solubility.^8^ Over time, Fsp3 in drugs is falling (median values 0.43 in 1939-1989, 0.35 in 2010-2020, Table 3). The overall [drug - target median] Fsp3 values are positive in each time frame, though reducing over time. However, this trend is caused by the carboaromatic and aliphatic drugs; heterocyclic drugs, as seen with their ALogP and aromatic ring counts, are not distinguished by Fsp3 from their target comparators (Supporting Figure S9). Thus it appears that the requisite solubility in drugs is more often achieved by reducing lipophilicity through introducing aromatic heteroatoms, than by increasing Fsp3.

Drug stereocenter count is consistently higher than for their corresponding target compounds, independently of time (Table 4). However it should be noted that in in each time period, the majority of drugs have the same stereocenter count as their median target compounds (326 of the total of 643 primary drug-target pairs). Rotatable bond count shows the same trends over time as other bulk properties, with no differentiation seen between drugs and target compounds in the 2010-2020 drugs (Table 4).

### Composite descriptors

The QED parameter for drug-likeness combines eight property values generated from desirability scores^47^ (see Tables 1 and 5) and shows a reduction in each successive time period for both drugs and target compounds (Figure 5f). Although, in common with most of its component properties, QED shows little or no overall discrimination between drugs and their target compounds, the variation in QED values by target class and therapy area is prominent in the 1900-2009 and 2010-2020 drugs (Supporting Figure S4). In common with MW and ALogP, QED values by target class and therapy area are similar for both drugs and target comparators in both time periods.

LogD_7.4_ + nAr (Table 5) provides little improvement over LogD_7.4_ alone (Table 3) in differentiating drugs from target compounds. It should be noted that LogD_7.4_ + nAr uses calculated LogD_7.4_ instead of an experimentally determined, and differently scaled, chromatographic LogD_7.4_, as recommended in the derivation of this metric, known as the Property Forecast Index (PFI).^8^ The parameter AB-MPS, designed to rationalise bioavailability trends in bRo5 chemical space,^53^ has also been linked to passive permeability within the Ro5.^13^ However, AB-MPS, which is correlated with rotatable bonds in the drug set (r=0.894, n=643), only differentiates the 1939-1989 drugs from their target comparators (Table 5). Overall, the multiparameter metrics QED, LogD_7.4_ + nAr and AB-MPS are no more effective than individual physicochemical properties in distinguishing drugs from their target compounds.

### Potency (pChEMBL)

Over all three time periods, drugs have consistently higher pChEMBL values than their target compounds (Table 6 and Figure 5g). pChEMBL shows greater drug versus target compound differentiation than any of the physicochemical properties in Tables 3–5, with the exception of aromatic ring counts in carboaromatic drugs. For the 643 drugs acting at their primary targets, 536 (83%) have higher pChEMBL values than their target medians. Since seeking high potency is a central focus of optimisation in drug discovery, perhaps even an obsession,^54^ it is notable that as many as 107 of the 643 drugs (17%) have pChEMBL values less than or equal to their target medians. However, drug pChEMBL is increasing, and increasingly differentiating drugs from targets over time (see drug-like ratio, Table 6). Potency alone is the most important drug versus target differentiator for the 2010-2020 drugs, where only 9 of the 141 drugs have potency less than or equal to than their target medians (Table 6). The widespread application of structure-based drug design, for example in the discovery of kinase inhibitors,^60^ is likely to have had a significant impact on the optimisation of potency in 2010-2020 drugs.

Figure 7a illustrates the poor correlations between drug and target comparator pChEMBL over all time periods noted above (Figure 4b). The target class breakdown (Figure 7b) shows a more consistently positive separation between drug and target comparator in the 1990-2009 and 2010-2020 periods versus the 1939-1989 drugs. The overall pChEMBL trends by time (Figure 5g) are similar because the aminergic GPCRs and nuclear hormone target classes, where drug pChEMBL is greater than their target medians (Figure 7b), are numerically dominant in the 1939-1989 set (Figure 2a). Median potencies of ion channel drugs are lower than most other target classes in all time frames (Figure 7b). By therapeutic use however, the pChEMBL [drug - target median] differences are consistent and nearly all positive over all time frames (Figure 7c).

### Ligand efficiency metrics

A key question with the various ligand efficiency metrics is do they improve on pChEMBL alone in differentiating drugs from their target compounds? Using the drug counts in Table 6, odds ratio calculations (applying significance level p <0.05) support the following conclusions regarding differentiation of drugs from their target compounds: a) SEI^10^ is a poorer differentiator than pChEMBL in all time periods; b) all efficiency metrics other than SEI improve on pChEMBL in the 1939-1989 period; c) pChEMBL and all efficiency metrics other than SEI perform similarly in the 1990-2009 period; and d) pChEMBL is a superior differentiator to all efficiency metrics in the 2010-2020 period, except for SILE^55^ and FQ^56^ with which it is similar.

It is important to note that there is considerable redundancy between the various ligand efficiency metrics and their constituent parameters (pChEMBL, MW, HA count and ALogP), as shown by the cross-correlation matrix for the 643 drugs in this study, shown in full in Supporting Figure S10a. As expected BEI and LE are strongly correlated (r = 0.975), but so too are SILE and FQ (r = 0.987, Supporting Figure S10b). SILE and FQ were designed^55,56^ to overcome the negative correlation with HA count seen for LE (r = -0.221 and -0.098 for SILE and FQ respectively; r for LE versus HA count = -0.793) and as expected, show no time dependent changes in either drugs or targets in comparison with LE (Table 6). However, SILE and FQ are correlated with pChEMBL (r = 0.819 and 0.882 for SILE and FQ respectively; Supporting Figure 10b) while LE is not (r = 0.101). This co-correlation explains the similar drug versus target comparator performance of pChEMBL, SILE and FQ (Table 6). AEI^57^ has better performance than SEI in discriminating drugs from target comparators (Table 6) although these parameters are also correlated (r = 0.939). LLEAT^58^ and LELP,^59^ which combine pChEMBL, ALogP and HA count, are correlated respectively with LE (r = 0.819) and ALogP (0.881, Supporting figure S10b), while LE and LLE however are not strongly correlated (r = 0.376, Supporting Figure S10b). We conclude that LE (Figure 5h) and LLE (Figure 5i) are sufficient to capture the variance in size and lipophilicity efficiencies. LE and LLE, along with pChEMBL and carboaromatic count, show drug versus target comparator discrimination in all time periods (Figure 4).

The [drug - target median] difference in LE is reducing over time because drug size is increasing, yet remains positive in the 2010-2020 drugs for all target classes except ion channels and nuclear hormone receptors (Supporting Figure S5). However, the numerically dominant kinase inhibitors^60^ (Figure 2a) have a lower [drug - target median] LE difference than the remaining target classes and therefore strongly influence the 2010-2020 overall LE trend (Figure 5h, Supporting Figure S5). The median LLE value of all drugs is 4.8 and is not changing over time (Figure 5i), supporting the importance of this metric in lead optimisation. The median [drug - target median] LLE difference is reducing over time, from 2.0 in 1939-1989, to 1.8 in 1990-2010 and 1.4 in 2010-2020. This is a consequence of the corresponding target comparator compounds showing an increase in LLE (Table 6, Figure 5i). As with LE, ion channels and nuclear hormone receptors display the lowest [drug - target median] differences in LLE in 2010-2020 drugs (Supporting Figure S6). Anticancer drugs in 2010-2020 comprise the largest therapy area and are predominantly kinase inhibitors (Figure 2a), showing below average LE and LLE [drug - target median] differences relative to other therapy areas (Supporting Figures S5 and S6).

### Combinations of size, lipophilicity and potency

**Figure 8:**
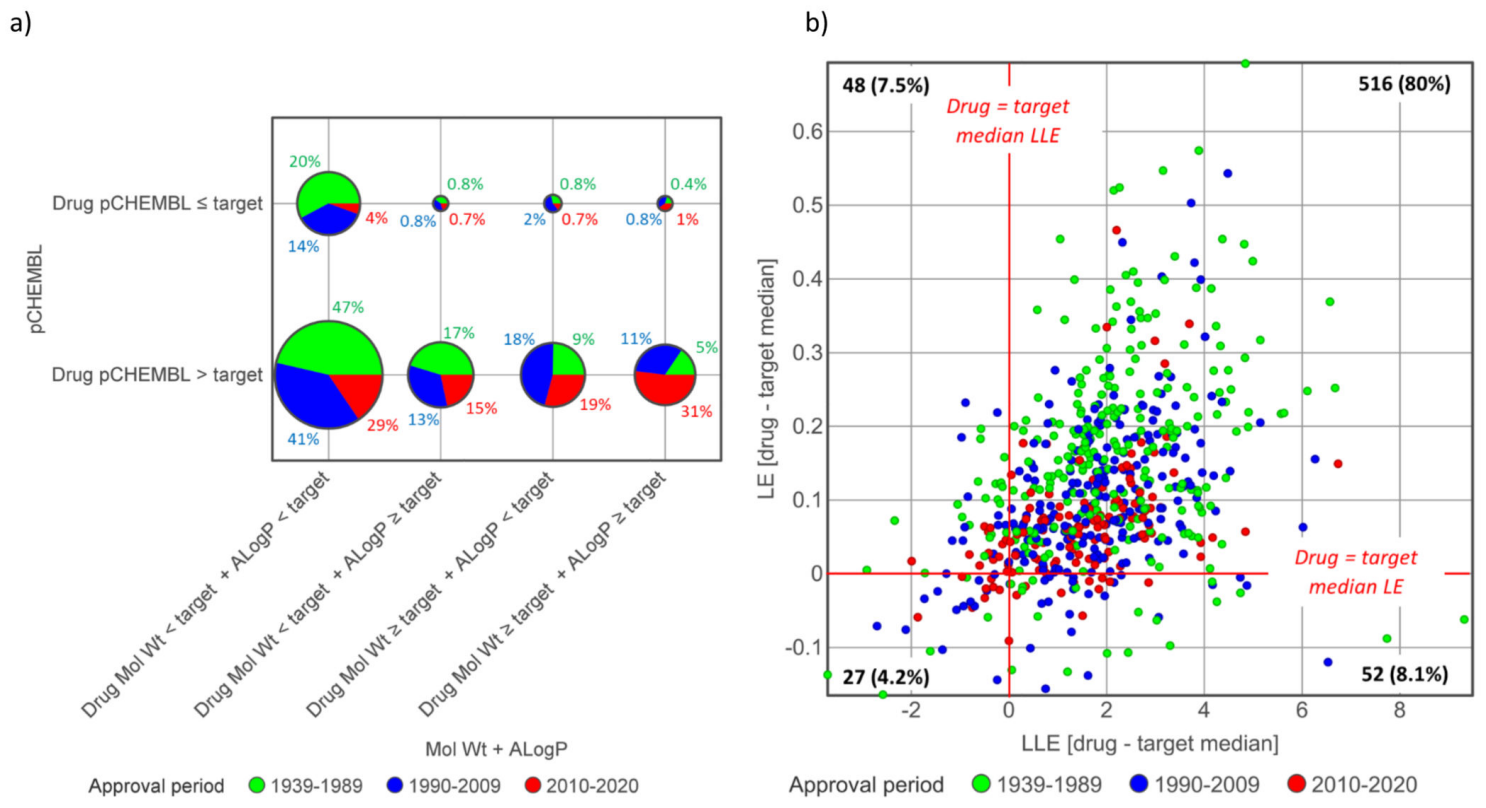
Differentiation of drugs from their primary target compounds using combinations of potency, size and lipophilicity. a) Percentages of drugs with molecular weight and ALogP greater or lower than their primary target’s median values, split by their potencies (pCHEMBL) being greater or lower than their primary target median values. b) Plot of LE versus LLE [drug - target median] differences. The population of drugs in each quadrant is shown.

Of the 107 drugs where the drug pChEMBL is lower than or equal to their target median, only 9 have one or both of MW and ALogP greater than or equal their target medians (Figure 8a). In other words, 98.6% (634/643) of drugs have one or more of higher pChEMBL, lower MW or lower ALogP relative to their target median values. Similarly, 27 of the 643 drugs (4.2%) have both LE and LLE values lower than their target medians, and 516 (80%) have both LE and LLE greater than their target medians (Figure 8b). In all time frames there are more drugs with both LE and LLE values higher than the target 90 percentiles than there are below the target medians, but with differences between these groups reducing over time (Table 8).

These results are similar to earlier findings with much smaller datasets,^11,31^ and confirm that higher LE and LLE are both equally important in differentiating drugs from their median target compounds. Because drug and target median lipophilicity and size are not different overall in the 2010-2020 drugs (Table 3, Figure 5a,b), using the combination of LE and LLE in this set distinguishes target compounds and drugs to the same extent as potency alone.

### Beyond rule of five (bRo5) drugs

A total of 29 drugs in this study have both MW >500 and ALogP >5.0, of which 27 were approved post-1990. For these drugs, potencies at their primary targets are increased versus target medians to the same extent as the full set. Unsurprisingly, MW and ALogP are also increased, resulting in low [drug - target median] differences for both LE and LLE. The median [drug - target median] differences for the 29 drugs are: pChEMBL, +1.2; MW, +83; ALogP, +1.5; LE, 0.0; LLE -0.3. The median pChEMBL value of these drugs is 8.8, higher than for the full drug set (8.1).

### Oral dose

Oral dose has been linked to the AEI (ADME efficiency index, Table 1),^57^ and we therefore evaluated correlations between dose (oral drug maximum daily dose, -p[Dose] = log_10_ (dose in mg/MW/1000), and potency or ligand efficiencies (including [drug - target median] values) for all 562 drugs in this study where a maximum oral daily dose was available.^34^ In brief, all correlations were found to be weak, with none of the efficiency metrics, including AEI, significantly improving on pChEMBL alone.

**Figure 9:**
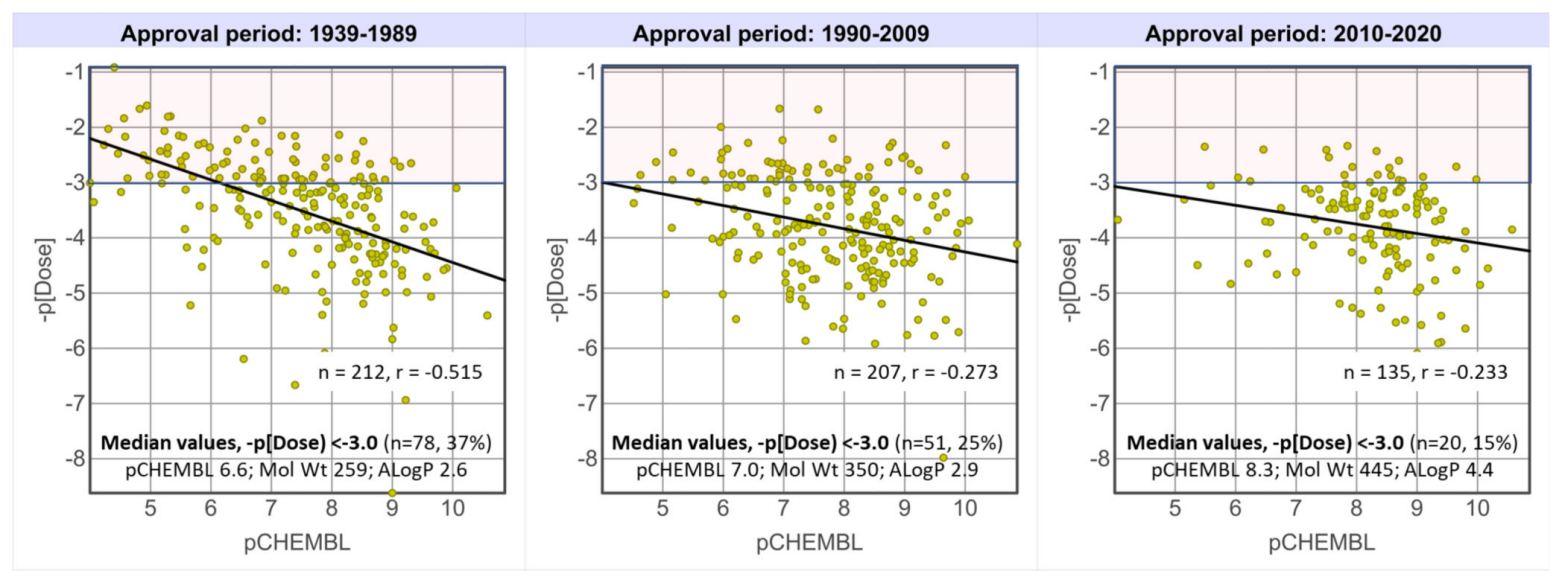
Maximum daily oral dose (-p[Dose]) versus pCHEMBL for 562 oral drugs identified in this study (including those with <100 comparator compounds at their targets of action) by time period of drug approval. –p[Dose] = -3.0 is the upper quartile oral dose value for all 562 drugs. Data used are Supplementary Spreadsheet 2.

The -p[Dose] versus pChEMBL correlation coefficient for all 562 drugs is r = -0.39, lower than that in an earlier study using fewer drugs (n=261, r = -0.51).^5^ However, over time, this correlation is weakening (Figure 9). The trend is exemplified by the upper quartile dose group (-p[Dose] > -3.0 (equivalent to a dose of >~400 mg for a drug with MW = 400), where median potency increases by ~50 fold between the 1939-1989 and 2010-2020 drugs, and is accompanied by increases in MW and ALogP (Figure 9). Increases in physicochemical properties over time may therefore be exerting an increasingly negative impact on ADME properties, for which higher potency does not compensate. Other factors influencing the dose-potency relationship are differing degrees of target occupancy being required for therapeutic efficacy, and lengthy clinical experience with older drugs, which may have led to acceptable dose escalation. The reducing percentages of drugs in upper quartile dose range over time (Figure 9) is consistent with increased MW and ALogP, as observed in studies of the oral doses of a larger set of drugs.^34^

## Perspective

In this study we have assessed ‘drug-like’ parameters in common usage by comparing the properties of small molecule drugs with comparator compounds from the medicinal chemistry literature that act at their annotated therapeutic targets, using binding affinities obtained from ChEMBL version 26. To take into account the variability seen in median physicochemical properties across compounds acting at different biological drug targets, we used the quantitative differences between drugs and their target median properties. The drug approval time period is a key element of this work because drugs are increasing significantly in size and lipophilicity over time, and accordingly they were grouped into three sets for analysis, namely approvals in 1939-1989, 1990-2009, and 2010-2020.

It is the findings from the later time periods, 1990-2009 and especially 2010-2020, that are the most relevant, where the coalescence of many drug and target median properties over time is a striking observation. As discussed earlier, the selection of the target comparators is critical. The earlier 1939-1989 drugs would, with high probability, be better matched to the their contemporaneous discovery molecules than those that necessarily had to be used for this study. In any event it is evident that recent drugs are significantly differentiated from target compounds by a rather short list of parameters: high potency (expressed here as pChEMBL), ligand efficiencies (LE and LLE), and low carboaromaticity. Potency is the most important differentiator of all the measures investigated. The median drug pChEMBL at primary targets (n=643) is 8.1, compared with 7.7 (n=261) in an earlier study.^5^ The corresponding target comparator compounds set a relatively high bar with a median pChEMBL of 6.8, perhaps because of a tendency to focus on more potent compounds in publications. It is highly likely that the true [drug - target median] potency difference would be even greater if all the compounds studied in drug discovery projects were included.

The ‘classical drug-like’ parameters such as MW, lipophilicity, PSA and hydrogen bonding, as well as heteroaromatic ring count and the multiproperty metric QED, show declining differentiation between drugs and target compounds over time. This culminates in no differences being observed in these properties between 2010-2020 drugs and their targets. However, the weakening of the potency versus dose correlations for oral drugs over time (Figure 9) tracks with increasing bulk physicochemical properties, suggesting this creates additional ADME optimisation issues. These issues have obviously been overcome in the discovery of marketed drugs.

While potency is the major drug versus target compound differentiator for 2010-2020 drugs, increased potency does not, on average, come at the expense of increased size or lipophilicity. Combinations of MW, ALogP and pChEMBL, and LE plus LLE, discriminate between drugs and target compounds in all time periods. Thus, 98.6% of all drugs have one or more of higher pChEMBL, lower MW and lower ALogP versus their target medians, and 95.8% have one or both of LE and LLE greater than their target medians. LE and LLE both have similar impact, supporting previous results using smaller sets of targets, and trends seen in optimisation. The LLE results are generally consistent with this parameter frequently increasing in lead optimisation.^11,24,31^ Amongst kinase inhibitors, which dominate the 2010-2020 drugs, the median LLE [drug - target median] difference is smaller (0.6) than all other target classes except for ion channels (0.2) (Supporting Figure S6). We suggest that most drugs have improved LE and LLE values versus their target medians as a result of optimisation leading to specificity for binding to those targets.

The results suggest there is strong case for optimising LE, so that in drug candidates it is above the median for the drug’s known target compounds. The absolute LE value of a drug candidate is less important. LE is widely applied, especially in fragment-based drug discovery,^61^ although its scientific basis and application have provoked a literature debate.^62–64^ There is a high probability that molecular size increases in the optimisation process, which then reduces the maximum LE obtainable.^11,55,56^ In compilations of start-to-finish optimisations of drugs^65^ and literature compounds,^24^ mean MW and potency increase, and LE does not -in other words, LE increases or decreases about equally, in contrast to LLE, which mostly increases. The MW increases during successful optimisation can be substantial: for 60 hit-to-drug^65^ and 66 recent hit-to-candidate pairs^66^ the median change was +93 and +85 Daltons respectively (median lipophilicity did not change significantly in either study). Of the 60 hit-to-drug pairs, 51 drugs are included in this study and despite average LE not changing in their optimisation, their [drug – target] LE and LLE differences mirror the full set of targets shown in Figure 8b. A hit or lead with high LE and low MW can be optimised, ultimately keeping above the target LE median, by increasing MW while keeping LE constant, or even allowing LE to decrease. On the other hand, a hit with low LE below the target median, resulting from high MW and modest potency, will require LE to increase in optimisation.

**Figure 10:**
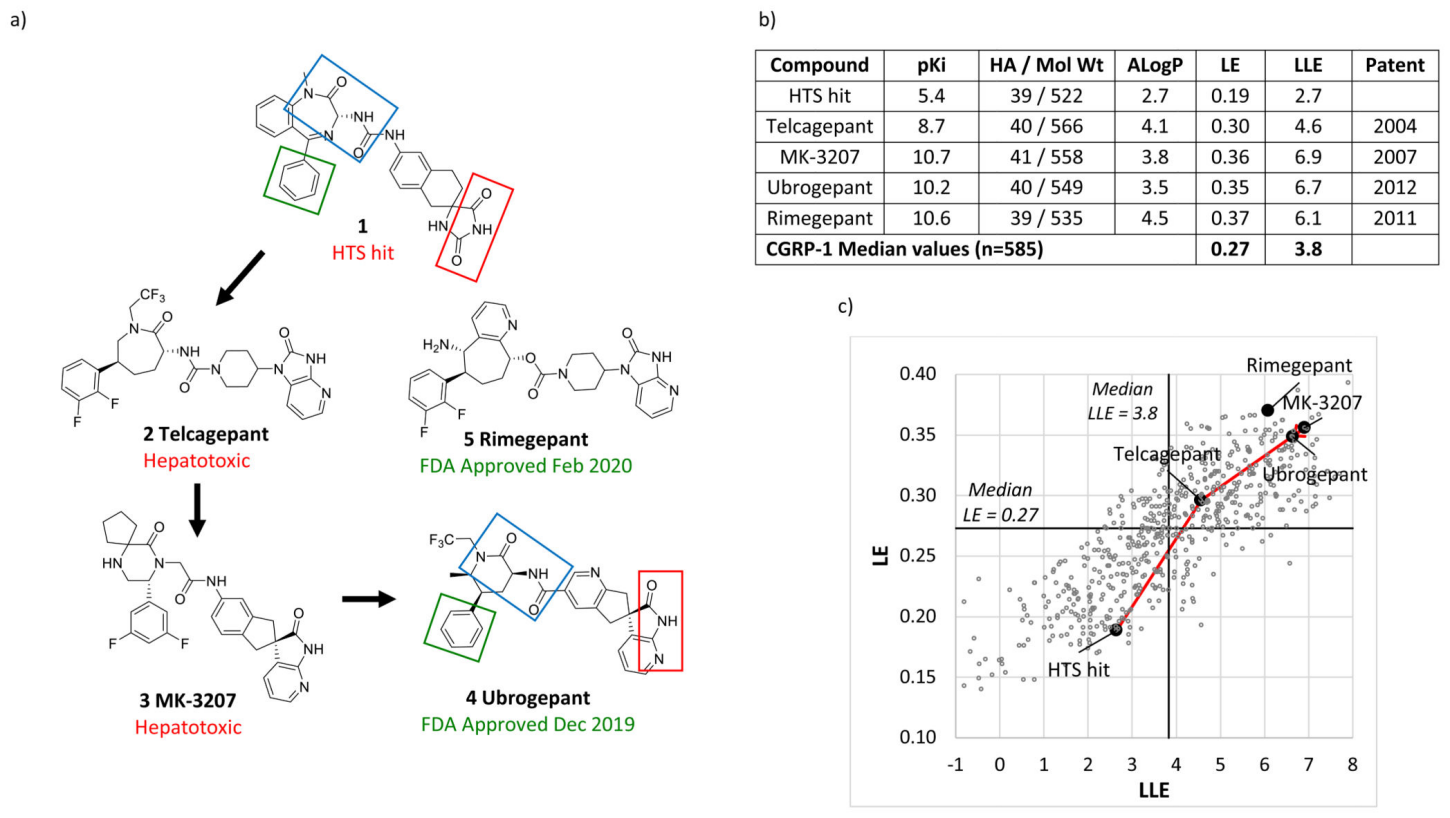
Evolution of oral CGRP antagonists from a high molecular weight HTS hit.^67,68^ a) Initial Merck HTS hit **1**, failed clinical candidates **2** and **3**, and marketed drugs **4** and **5**. Three pharmacophoric elements highlighted are present in hit **1** and retained in the Merck program, shown by the arrows, leading to the marketed drug **4**. In **5**,^70^ discovered by BMS, the azepine ring substituents of **2** are modified. b) Molecular properties and year of 1^st^ patent. c) LE vs LLE plot with CHEMBL comparators showing hit-to-drug trajectory (n=585 Ki values).

The results imply that the increases seen over time in drug properties are to a significant extent a consequence of the requirements of the targets selected. An example of a recent target requiring high MW, illustrating the associated discovery challenges, is the calcitonin gene-related peptide receptor (CGRP or CLR1/RAMP1),^67,68^ where the compounds from the literature used in this study have a median MW of 555 and a median ALogP of 4.2 (n=585). The drug binding site is formed by a protein-protein interaction between the calcitonin-like receptor (CLR1), a 7-transmembrane protein, and the receptor activity modifying protein 1 (RAMP1), a single transmembrane protein. A high throughput screen run at Merck provided hit compound **1**, which despite a modest pKi of 5.4 and high MW of 522 (LE = 0.19) was considered of interest because it is composed of two ‘privileged’ fragments - structures which commonly appear in biologically active molecules - namely a benzodiazepine and a spirohydantoin (Figure 10a). The journey from **1** to the marketed drug **4** increased potency dramatically, by 100,000-fold, yet physical properties were barely changed, with only one heavy atom added and a modest increase in ALogP of 0.8, thereby realising substantial increases in both LE and LLE in **4** versus **1** (Figure 10b). Achieving suitable pharmacokinetic properties was a major challenge, yet notably, most of privileged structure pharmacophoric elements in **1** were ultimately retained (Figure 10a), with the exception of the benzo fusion. In general, retention of pharmacophoric features in hit optimisation is often seen.^31,66^ Extracting higher potency without little change to MW, resulting in increased LE, was also consistently seen in a set of 15 hit-to-lead optimisations derived from DNA-encoded libraries, where the average hit MW was 533.^69^

The first two CGRP antagonist clinical candidates, **2** and **3**, had to be abandoned because of hepatotoxicity in the clinic, and based on the published patents, it took 8 years to move from the first candidate **2** to the successful drug **4**. The time frame reflects the challenge of multiparameter ADMET optimisation for this difficult target, the wealth of options for modification that are presented by the hit structure **1**, and not least, the considerable commitment needed from the project team. All the clinical candidates, including the second marketed drug **5**,^70^ are amongst a handful of the most highly ligand efficient compounds reported at the CGRP receptor (Figure 10c). The determination of ligand-bound CGRP receptor structures has subsequently helped rationalise structure-activity relationships, yet reducing MW and substantially changing the pharmacophore remain challenging.^71^

Approved drug MW and lipophilicity have been increasing over time and it appears likely that the cohort of drugs breaking the Ro5 will continue to grow.^28,72^ In this study, bRo5 drugs having MW >500 and ALogP >5 (29 drugs), in comparison with their corresponding target median compounds, unsurprisingly had higher lipophilicity, size and potency, resulting in [drug - target median] LE and LLE values lower than for other drugs. The nature of the drug binding sites for some targets may necessitate physicochemical properties in bRo5 space.^28,72^ The findings of this paper are consistent with this, in that the nature of the drug’s target has a major impact in influencing the physicochemical properties of drugs and discovery compounds.

It is becoming evident that in vitro ADME assays, such as permeability and plasma protein binding, can be unreliable for highly lipophilic bRo5 compounds because of poor solubility and high levels of non-specific binding to laboratory vessels.^73^ However, it has been observed that such compounds are often bioavailable, which may have been missed in the past because compounds not meeting in vitro criteria would not have progressed to in vivo studies. For example, amongst proteolysis-targeting chimeras (Protacs), bifunctional protein degraders with MW usually >700, pharmacokinetic studies in mice^73^ showed that >30% bioavailability appeared regularly in compounds with the fewest HBA/HBD counts and chromatographic LogD_7.4_ = 5-7 (beyond the reliable range of shake-flask methods). A caveat to this study is the lack of structural variability in the E3 ligase binding moiety of the Protacs reported.^73^ However, in a large diverse set of bRo5 compounds,^53^ 25% of those tested had bioavailability in rats of >27%, leading to the development of the AB-MPS metric discussed above (Table 4), which requires an optimal LogD_7.4_ of 3. The apparent difference in LogD_7.4_ requirements in these two studies is interesting. As in traditional ‘drug-like’ space, small molecular changes in bRo5 compounds, for example aromatic fluorination,^74^ can have a significant impact on potency and oral exposure.

The simple physicochemical properties discussed here are common currency amongst medicinal chemists. They have served the community well, and will likely be useful for some time yet. For example, values of PSA <75 Å^2^ in combination with LogP >3 have been shown in several studies from large pharmaceutical companies to increase the risk of in vivo toxicity.^75–77^ Drug candidates that fail due to clinical toxicity are more lipophilic on average than those that do not.^78^ Lipophilicity^13, 44, 79–81^ will remain a key driver of ADMET properties and strategies to assess^82^ and control^83,84^ it are growing. Measurement of LogD_7.4_ and LogP is essential,^13^ and chromatographic methods^85^ have greater measurement bandwidth than the traditional shake flask method.^8,86^ Lipophilicity associated with high aromatic ring count has been highlighted as significant developability risk,^8,9^ with carboaromatic compounds carrying greater risks than heteroaromatics.^42^ The results in this study are consistent with these observations, showing that in comparison with their target compounds, carboaromatic drugs, but not heteroaromatic drugs, have lower aromatic ring count and lIpophilicity values versus their target medians, independently of the drug approval period. Optimising LLE^44^ is a practical means of keeping lipophilicity under control, and the application of LLE is gaining broad acceptance.^11,31, 87–90^

Taking account of conformational flexibility and intramolecular interactions, by using 3-dimensional shape and polarity descriptors, is providing insights into bRo5 compound properties,^29, 91^ and this approach will also be useful within traditional ‘drug-like’ space. Experimentally estimated exposed polar surface area should supercede 2-D PSA calculations, and can be achieved using supercritical fluid chromatography.^92^ Emphasising predicted solubility and permeability instead of lead-like physical properties is an interesting development in the construction of compound screening collections.^93^ Similarly, keeping measured solubility and lipophilicity under control, together with aiming for a low predicted human dose, are key elements in helping to reduce compound-related attrition in development.^12^

While target compounds and 2010-2020 drugs have similar median physicochemical properties, there is obviously variability as shown by the box plots in Figures 5–7 and Supporting Figures S2-S6, S8 and S9. One source of variability comes from different institutional approaches to drug discovery.^7,16,44^ For example, for targets they had in common, Pfizer’s patented compounds in the period 2000-2010 were consistently smaller and less lipophilic than Merck’s.^16^ It is interesting to note that differences in corporate philosophies reflected by these data continue to be evident in recent publications. Pfizer has emphasised the application of LLE (or LipE) in projects,^90^ while Merck, although acknowledging the importance of balancing lipophilicity, believes that using this property in design limits options excessively.^94^ Nevertheless, the convergence of drug and target compound properties seen in 2010-2020 suggest that overall, the community has responded positively to the call, initiated by the Ro5^6^ and reinforced by subsequent studies,^1–5,8,16,24,44,79,97^ to improve property-based design. While drugs are increasing in size and lipophilicity, low MW drugs can still be found.^95^ Choosing lead-like chemical starting points^96^ that allow compound design within the historical MW/lipophilicity ‘sweet spot,’^97^ will remain a valid strategy for many targets.

**Figure 11:**
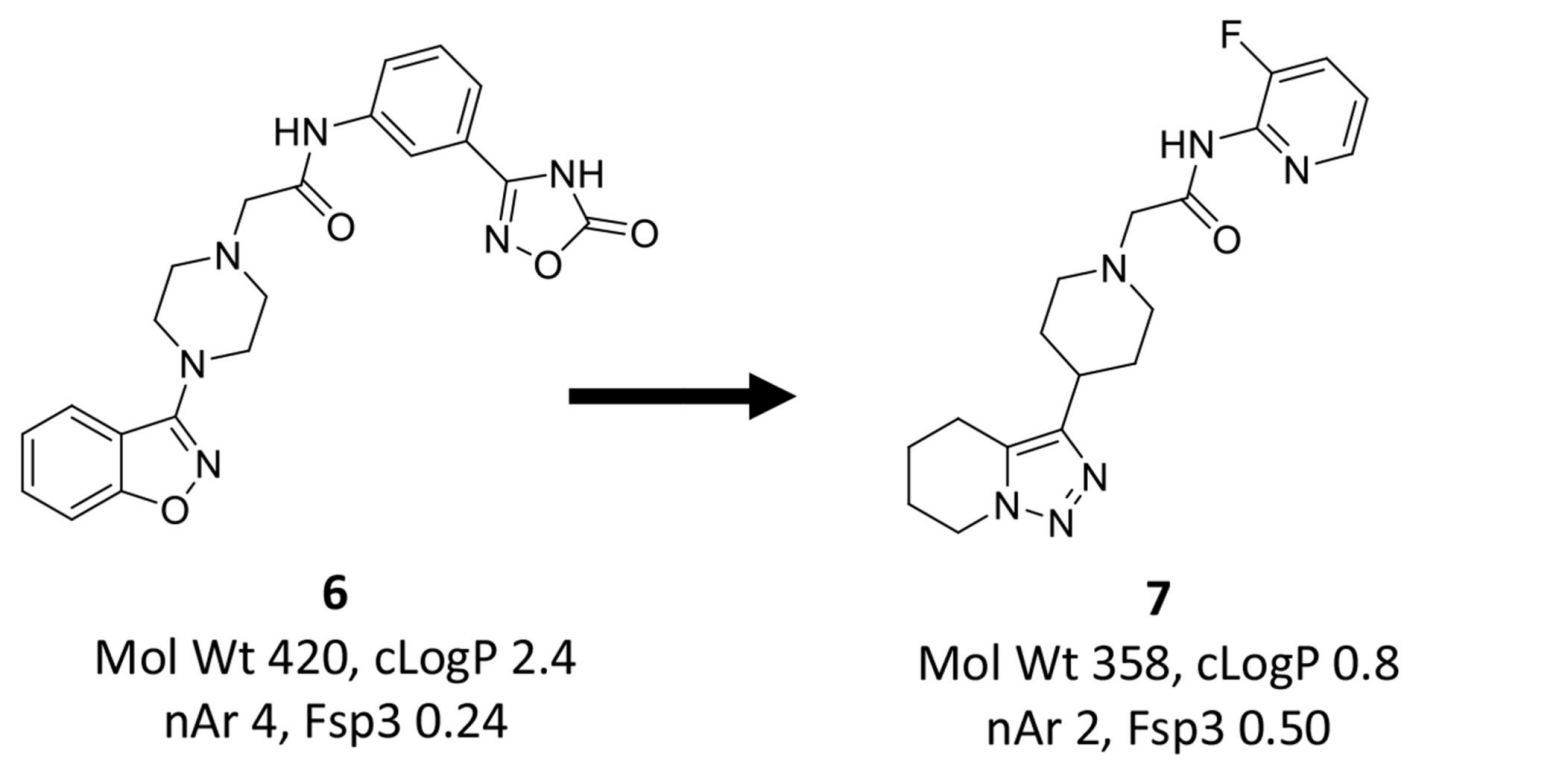
Multiparameter lead optimisation using generative predictive tools. Compounds **6** and **7** act as activators of an undisclosed phenotypic target.^102^ Compound **6** was the best of 881 compounds across 6 off-target and 4 ADME assays. The generative model predicted 150 compounds would improve on **6** of which 11 were made, including **7**, which met all 10 assay objectives. cLogP values from CHEMDRAW (https://www.perkinelmer.com/category/chemdraw).

A significant problem in the application of physicochemical properties in drug design is the cross-correlation and potential redundancy between many of them, as well as amongst ligand efficiency metrics and potency, as observed in this study. It is well established that lipophilicity is a composite property determined by molecular size, polarity, ionisation state and hydrogen bonding.^52^ Even more fundamentally, many experimental properties of organic compounds can be largely predicted by just two principal components, termed ‘bulk’ and ‘cohesiveness’ by Cramer.^98^ Medicinal chemists rarely make use of principal components, probably because, despite Cramer’s helpful terminology, their physical meaning is considered elusive in comparison to LogP, HBA, MW, etc. Medicinal chemists need to become more computationally aware, because predictive computational chemistry methodology is rapidly advancing, with molecule generation using artificial intelligence and machine learning promising to streamline decision-making and shorten discovery timelines.^99^ Overcoming any hurdles preventing close collaboration with computational chemists will be essential for success in these areas.^100,101^ An example, said to be the first report of using a deep learning generative model in multiparameter lead optimisation, is the discovery of **7** from the previous best compound **6** (Figure 11).^102^ Compound **7** was one of only 11 compounds synthesised from a total of 150 predicted to have better overall properties than **6**. The triazine moiety in **7** had been tried earlier and it performed poorly, until the pyridyl amide modification was suggested by the generative tool. This modification had not been considered previously by the chemists. In compound **7**, several changes have been made to the structure of **6** while physiochemical properties are significantly improved (Figure 11). Another team of chemists, employing property-based design instead of generative models, might conceivably have found a similar solution, but perhaps would have needed to synthesise more compounds? Of equal importance to computational chemistry is the application of existing and new synthetic chemistry methodologies, which can open fresh avenues of exploration.^103^ Challenging synthetic chemistry will be needed to realise the opportunities presented by unexplored natural product scaffolds^104^ and expanding 3-dimensional pharmacophore space, which, overall, appears to be relatively unexplored in drugs and research compounds.^105^

## Conclusion

Over time, commonly used physicochemical properties, including MW, lipophilicity (LogP and LogD_7.4_), PSA, hydrogen bond donors and acceptors, heteroaromatic ring count and QED, show declining differentiation between drug values and the median values for published compounds acting at the drug’s biological targets. Drugs approved in 2010-2020 and their corresponding target compound median values show no differences in these properties. This means that the Ro5,^6^ which has become a seemingly indelible reference point in drug discovery,^13,30,106,107^ does not, on average, differentiate recently approved drugs from published discovery compounds. Instead, the physicochemical property space of recent drugs predominantly reflects the constraints imposed by their biological targets.

This study, like all those looking at marketed drug properties, is necessarily retrospective. Nevertheless, those small molecule drug properties that show consistent differentiation from their target compounds over time - namely potency, ligand efficiencies (LE and LLE) and the aromatic ring count and lipophilicity of carboaromatic drugs - are those that are most likely to remain future-proof. Candidate drugs emerging from target-based discovery programs should ideally have one, or preferably both, of their LE and LLE values greater than the median value for all other compounds known to be acting at the target.

## Supplementary Material

Fig 5 values

Fig 6 values

Fig 7 pChEMBL values

Fig 9 doses

Supp drug target and drug_target data

Supp drugs and compounds per target

Supp Fig 8 values AlogP

Supp Fig S2 values

Supp Fig S3 values Mol Wt

Supp Fig S4 values QED

Supp Fig S5 values LE

Supp Fig S6 values LLE

Supp Fig S9 values Fsp3

Supp manual curation approval dates

Supp manual curation_prodrugs

Supp manual curation_route of admin

Supporting figures S1-S10

## Figures and Tables

**Table 1:** Drug and target compound properties and descriptors examined. ^a^ ALogP is from an atom-based calculation and LogD_7.4_ is from ChemAxon.^35^ ^b^ QED = summed, weighted desirability scores using [MW + ALogP + HBD + HBA + PSA + nRotB + nAr + number of alerts].^47^ ^c^ AB-MPS = | LogD_7.4_ – 3| + nAr + nRotB.^53^ ^d^ Experimental LogD_7.4_ determined by chromatography + nAr is known as the Property Forecast Index (PFI).^8^ ^e^ LE = Ligand efficiency = [pChEMBL x 1.37] ÷ HA (units are kcal/mol/HA).^43^ ^f^ BEI = Binding efficiency index = [pChEMBL x 1000] ÷ Mol Wt.^10^ ^g^ SILE = Size-independent LE = pChEMBL ÷ HA^0.3^.^55^ ^h^ FQ = Fit quality = [pChEMBL ÷ HA] ÷ [0.0715 + (7.5328 ÷ HA) + (25.7079 ÷ HA^2^) – (361.4722 ÷ HA^3^)].^56^ ^i^ LLE (or LiPE) = Lipophilic ligand efficiency = pChEMBL – ALogP.^44^ ^j^ LLEAT = LLE adjusted for HA count = 0.111 + [(1.37 x LLE) ÷ HA] (LLE based on ALogP).^58^ ^k^ LELP = LE price paid in lipophilicity = ALogP ÷ LE.^59^ ^l^ SEI = Surface efficiency index = [pChEMBL x 100] ÷ PSA.^10^ ^m^ AEI = ADME efficiency index = [pChEMBL - |ALogP |] x 100 ÷ PSA.^57^

Category	Properties and descriptors
Size	Molecular weight (MW), HA count (HA)
Lipophilicity	Calculated octanol-water partition coefficients ALogP and LogD_7.4_^a^
Polarity	Polar surface area (PSA), hydrogen bond donors (HBD) and acceptors (HBA)
Aromatic and aliphatic descriptors	Aromatic ring count (nAr) of heteroaromatic and carboaromatic drugs, fraction of carbon atoms that are sp3 hybridised (Fsp3) and number of stereocenters (nStereo)
Flexibility	Rotatable bond count (nRotB)
Composite physicochemical descriptors	Quantitative estimate of drug-likeness (QED),^b^ AbbVie multiparameter score (AB-MPS)^c^ and LogD7.4 + nAr^d^
Potency	pChEMBL = -log1o (molar concentration IC50, Ki, etc) in binding assays
Ligand efficiency metrics	LE,^e^ BE I,^f^ SILE,^g^ FQ,^h^ LLE,^i^ LLEAT,^j^ LELP,^k^ SEI,^l^ and AEI^m^

**Table 2:** Median molecular weights of contemporary published compounds from the *Journal of Medicinal Chemistry* ^23^ in comparison with drugs and target medians from this study.

Period	Median Molecular Weight		
	Compounds published in *Journal of Medicinal Chemistry*	This study	
		Drugs approved	Corresponding target medians
1959-69	290	300 (n=43)	386
2000-09	384	389 (n=107)	419

**Table 3:** Primary drug, target median and drug - target median values (n = 643) for molecular weight (MW), heavy atom count (HA), lipophilicity (ALogP and LogD_7.4_), hydrogen bond donors (HBD) and acceptors (HBA) and polar surface area (PSA). Entries in red show no significant difference between drug and target median (*t*-test p >0.05). ^a^ Drug-like ratio = [number of drugs < target median] ÷ [number of drugs > target median].

Property	Drug approval period	Drug			Target median			Drug - Target median			Number of Drugs				r
		Mean	Median	*Std dev*	Mean	Median	*Std dev*	Mean	Median	*Std dev*	> Target median (A)	< Target median (B)	= Target median	Drug-like ratio B/A^a^	Drug vs Target median
**MW**	1939-1989	320	300	*120*	395	394	*54.9*	-75.2	-80.6	*114*	39	220	0	5.64	0.339
	1990-2009	385	373	*131*	414	399	*76.1*	-28.8	-37.1	*107*	75	167	1	2.23	0.572
	2010-2020	452	448	*116*	448	439	*88.5*	4.2	2.0	*70.4*	73	68	0	0.93	0.795
**HA**	1939-1989	22.6	22.0	*8.3*	28.0	28.0	*4.1*	-5.4	-5	*7.85*	31	216	12	6.97	0.349
	1990-2009	27.2	26.0	*9.4*	29.2	29.0	*5.7*	-2.1	-3	*7.58*	72	161	10	2.24	0.595
	2010-2020	31.8	32.0	*8.0*	31.8	31.0	*6.4*	0.1	0	*4.88*	64	67	10	1.05	0.794
**ALogP**	1939-1989	2.7	2.7	*1.7*	3.9	3.9	0.7	-1.3	-1.2	*1.5*	58	199	2	3.43	0.389
	1990-2009	3.0	3.1	*2.0*	3.9	3.9	1.2	-0.8	-0.9	*1.4*	63	180	0	2.86	0.705
	2010-2020	3.6	3.8	*1.7*	3.8	3.9	1.0	-0.2	-0.1	*1.1*	66	74	1	1.12	0.773
**LogD_7.4_**	1939-1989	1.1	1.4	*2.1*	2.7	2.6	1.1	-1.6	-1.4	*2.0*	51	208	0	4.08	0.381
	1990-2009	1.3	1.7	*2.9*	2.5	2.8	1.8	-1.2	-1.0	*2.2*	69	173	1	2.51	0.666
	2010-2020	2.3	2.5	*2.1*	2.7	2.8	1.2	-0.4	-0.3	*1.5*	57	83	1	1.46	0.725
**HBD**	1939-1989	1.62	1	*1.62*	1.33	1	*0.88*	0.29	0	*1.47*	90	72	97	0.80	0.434
	1990-2009	2.02	2	*1.64*	1.65	1	*1.06*	0.38	0	*1.26*	79	41	123	0.52	0.646
	2010-2020	2.08	2	*1.33*	1.94	2	*1.01*	0.13	0	*1.06*	36	32	73	0.89	0.614
**HBA**	1939-1989	3.83	3	*2.35*	4.39	4	*1.07*	-0.56	-1	*2.10*	66	155	38	2.35	0.442
	1990-2009	4.80	4	*2.52*	4.63	4	*1.26*	0.17	0	*2.16*	83	105	55	1.27	0.520
	2010-2020	5.95	6	*2.39*	5.84	6	*1.40*	0.11	0	*1.75*	53	55	33	1.04	0.687
**PSA**	1939-1989	61.5	52.9	*47.6*	64.4	59.1	*22.1*	-2.94	-11.8	*42.2*	95	161	3	1.69	0.465
	1990-2009	81.6	72.7	*48.7*	74.8	68.0	*27.4*	6.76	-0.32	*38.0*	120	122	1	1.02	0.628
	2010-2020	93.8	90.5	*37.4*	91.8	91.4	*28.0*	1.98	0	*24.6*	69	70	2	1.01	0.754

**Table 4:** Primary drug, target median and drug - target median values (n=643) for aromatic, aliphatic and flexibility descriptors described in Table 1. ^a^ nAr carbo and nAr hetero are aromatic ring counts for drugs with zero and ? 1 aromatic heteroatoms respectively; aliphatic drugs are excluded. Entries in red show no significant difference between drug and target median (*t*-test p >0.05). ^b^ Drug-like ratio for nAr, nAr carbo, nAr hetero and nRotB = [number of drugs < target median] ÷ [number of drugs > target median]. Drug-like ratio for Fsp3 and nStereo = [number of drugs > target median] ÷ [number of drugs < target median].

Property	Drug approval period	Drug			Target median			Drug - Target median			Number of Drugs				r
		Mean	Median	*Std dev*	Mean	Median	*Std dev*	Mean	Median	*Std dev*	> Target median (A)	< Target median (B)	= Target median	Drug-like ratio **A**/B or **B**/A^b^	Drug vs Target median
**nAr**	1939-1989	1.40	1	*0.89*	2.42	2	*0.61*	-1.02	-1	*1.03*	11	**176**	72	16.00	0.099
	1990-2009	1.96	2	*1.19*	2.51	3	*0.88*	-0.55	0	*1.06*	35	**115**	93	3.29	0.502
	2010-2020	2.75	3	*1.17*	2.92	3	*0.79*	-0.17	0	*0.92*	26	**42**	73	1.62	0.618
**nAr carbo^a^**	1939-1989	1.49	1	*0.55*	2.40	2	*0.52*	-0.91	-1	*0.73*	2	**108**	41	54.00	0.096
	1990-2009	1.63	2	*0.66*	2.40	2	*0.64*	-0.77	-1	*0.82*	5	**61**	32	12.20	0.183
	2010-2020	1.69	2	*0.66*	2.48	2	*0.74*	-0.79	-1	*0.77*	1	**19**	9	19.00	0.517
**nAr hetero^a^**	1939-1989	2.11	2	*0.81*	2.45	2	*0.64*	-0.34	0	*0.87*	9	**27**	29	3.00	0.298
	1990-2009	2.73	2	*0.96*	2.78	3	*0.88*	-0.04	0	*0.90*	30	**30**	56	1.00	0.534
	2010-2020	3.17	3	*0.92*	3.09	3	*0.70*	0.08	0	*0.77*	25	**19**	63	0.76	0.549
**Fsp3**	1939-1989	0.45	0.43	*0.23*	0.34	0.35	*0.11*	0.11	0.07	*0.21*	**162**	93	4	1.74	0.395
	1990-2009	0.44	0.4	*0.21*	0.35	0.36	*0.12*	0.09	0.07	*0.19*	**147**	87	9	1.69	0.483
	2010-2020	0.37	0.35	*0.18*	0.32	0.31	*0.11*	0.04	0.03	*0.15*	**81**	54	6	1.50	0.556
**nStereo**	1939-1989	2.03	1	*3.11*	0.76	1	*1.10*	1.28	0	*2.71*	**103**	36	120	2.86	0.517
	1990-2009	1.81	1	*2.55*	0.84	0	*1.22*	0.96	0	*2.21*	**90**	16	137	5.63	0.496
	2010-2020	1.45	1	*2.05*	0.90	0	*1.48*	0.55	0	*1.59*	**51**	21	69	2.43	0.640
**nRotB**	1939-1989	4.12	4	*3.10*	5.59	5	*2.05*	-1.47	-2	*2.85*	44	**175**	40	3.98	0.448
	1990-2009	5.70	5	*3.70*	6.07	6	*2.47*	-0.37	0	*2.85*	79	**121**	43	1.53	0.640
	2010-2020	5.98	6	*2.92*	5.87	6	*2.15*	0.11	0	*2.27*	61	**57**	23	0.93	0.635

**Table 5:** Primary drug, target median and drug - target median values (n = 643) for composite descriptors defined in Table 1. Entries in red show no significant difference (*t*-test p >0.05) between drug and target median. ^a^ Drug-like ratio for QED = [number of drugs > target median] ÷ [number of drugs < target median]. Drug-like ratio for LogD_7.4_ + nAr and AB-MPS = [number of drugs < target median] ÷ [number of drugs > target median].

Property	Drug approval period	Drug			Target median			Drug - Target median			Number of Drugs				r
		Mean	Median	*Std dev*	Mean	Median	*Std dev*	Mean	Median	*Std dev*	> Target median (A)	< Target median (B)	= Target median	Drug-like ratio **A**/B or **B**/A^a^	Drug vs Target median
**QED^a^**	1939-1989	0.67	0.70	*0.18*	0.58	0.61	*0.13*	0.09	0.10	*0.18*	**185**	67	7	2.76	0.357
	1990-2009	0.59	0.62	*0.22*	0.53	0.57	*0.16*	0.05	0.07	*0.17*	**168**	71	4	2.37	0.649
	2010-2020	0.51	0.49	*0.20*	0.49	0.50	*0.15*	0.02	0.02	*0.15*	**76**	62	3	1.23	0.673
**LogD_7.4_ + nAr**	1939-1989	2.5	2.6	*2.5*	5.2	5.2	1.3	-2.7	-2.6	*2.5*	40	**219**	0	5.48	0.223
	1990-2009	3.3	3.6	*3.5*	5.0	5.3	2.3	-1.7	-1.6	*2.7*	63	**179**	1	2.84	0.662
	2010-2020	5.0	5.3	*2.8*	5.5	5.8	1.8	-0.5	-0.7	*1.9*	52	**89**	0	1.71	0.723
**AB-MPS**	1939-1989	7.7	7.4	*4.1*	9.6	9.3	2.4	-1.9	-1.9	*3.7*	56	**203**	0	3.63	0.459
	1990-2009	10.1	9.7	*4.8*	10.4	9.8	3.0	-0.3	-0.6	*3.8*	100	**143**	0	1.43	0.595
	2010-2020	10.4	10.1	*3.4*	10.5	10.1	2.4	-0.1	-0.1	*2.7*	66	**74**	1	1.12	0.629

**Table 6:** Primary drug, target median and drug - target median values (n = 643) for potency (pChEMBL) and ligand efficiency metrics, defined in Table 1. All entries show significant differences between drug and target median (*t*-test p <0.05). ^a^ Drug-like ratio for all metrics except LELP = [number of drugs > target median] ÷ [number of drugs < target median]. Drug-like ratio for LELP = [number of drugs < target median] ÷ [number of drugs > target median].

Property	Drug approval period	Drug			Target median			Drug - Target median			Number of Drugs				r
		Mean	Median	*Std dev*	Mean	Median	*Std dev*	Mean	Median	*Std dev*	> Target median (A)	< Target median (B)	= Target median	Drug-like ratio **A**/B or **B**/A^a^	Drug vs Target median
**pChEMBL**	1939-1989	7.48	7.82	*1.46*	6.62	6.73	*0.72*	0.86	1.00	*1.27*	**202**	56	1	3.61	0.448
	1990-2009	7.82	8.00	*1.27*	6.85	6.82	*0.76*	0.97	0.98	*1.12*	**202**	38	3	5.32	0.480
	2010-2020	8.37	8.47	*0.95*	7.08	7.14	*0.61*	1.29	1.31	*0.87*	**132**	8	1	16.50	0.444
**LE**	1939-1989	0.49	0.49	*0.15*	0.33	0.32	*0.06*	0.16	0.16	*0.13*	**236**	23	0	10.26	0.421
	1990-2009	0.43	0.40	*0.13*	0.33	0.32	*0.06*	0.10	0.09	*0.11*	**207**	35	1	5.91	0.597
	2010-2020	0.38	0.37	*0.10*	0.31	0.31	*0.05*	0.07	0.06	*0.08*	**121**	20	0	6.05	0.640
**BEI**	1939-1989	25.5	25.4	*7.70*	17.0	16.5	*2.94*	8.48	8.38	*6.98*	**235**	24	0	9.79	0.424
	1990-2009	22.1	20.8	*6.71*	17.0	16.5	*3.01*	5.12	4.71	*5.58*	**205**	38	0	5.39	0.567
	2010-2020	19.6	18.9	*5.03*	16.2	15.9	*2.43*	3.32	2.77	*3.92*	**121**	20	0	6.05	0.648
**SILE**	1939-1989	4.07	4.16	*0.75*	3.34	3.38	*0.35*	0.73	0.72	*0.66*	**230**	29	0	7.93	0.471
	1990-2009	4.03	4.08	*0.64*	3.42	3.42	*0.33*	0.61	0.61	*0.57*	**206**	37	0	5.57	0.459
	2010-2020	4.08	4.09	*0.46*	3.44	3.45	*0.27*	0.64	0.59	*0.43*	**133**	8	0	16.63	0.394
**FQ**	1939-1989	1.09	1.13	*0.20*	0.91	0.92	*0.09*	0.18	0.18	*0.17*	**224**	34	1	6.59	0.472
	1990-2009	1.08	1.10	*0.17*	0.93	0.93	*0.09*	0.16	0.15	*0.15*	**205**	38	0	5.39	0.447
	2010-2020	1.12	1.12	*0.12*	0.94	0.94	*0.07*	0.18	0.17	*0.11*	**133**	8	0	16.63	0.376
**LLE**	1939-1989	4.8	4.9	*2.0*	2.7	2.8	0.9	2.1	2.0	*1.7*	**233**	26	0	8.96	0.446
	1990-2009	4.8	4.8	*2.0*	3.0	2.8	1.2	1.8	1.8	*1.5*	**216**	26	1	8.31	0.663
	2010-2020	4.8	4.8	*1.8*	3.3	3.2	1.1	1.4	1.4	*1.3*	**119**	22	0	5.41	0.693
**LLEAT**	1939-1989	0.44	0.42	*0.18*	0.25	0.24	*0.06*	0.19	0.16	*0.16*	**241**	17	1	14.18	0.459
	1990-2009	0.38	0.36	*0.16*	0.26	0.25	*0.08*	0.12	0.11	*0.12*	**214**	27	2	7.93	0.709
	2010-2020	0.33	0.31	*0.12*	0.26	0.25	*0.06*	0.07	0.06	*0.09*	**114**	25	2	4.56	0.705
**LELP**	1939-1989	6.2	5.9	*5.1*	12.1	12.1	3.4	-5.9	-5.9	*5.2*	19	**240**	0	12.63	0.299
	1990-2009	8.2	7.5	*6.9*	12.1	11.7	4.9	-3.9	-4.3	*5.6*	37	**206**	0	5.57	0.599
	2010-2020	10.6	10.2	*6.4*	12.4	12.4	4.6	-1.8	-2.1	*3.9*	42	**99**	0	2.36	0.806
**SEI**	1939-1989	25.6	13.4	*39.0*	11.6	11.5	*4.17*	14.0	3	*37.7*	**180**	78	1	2.31	0.358
	1990-2009	14.5	10.3	*15.7*	10.5	9.58	*4.12*	3.98	1.04	*14.1*	**150**	92	1	1.63	0.499
	2010-2020	11.3	8.89	*9.23*	8.51	7.57	*2.99*	2.82	1.37	*7.38*	**107**	34	0	3.15	0.717
**AEI^j^**	1939-1989	14.3	8.15	*20.6*	4.61	4.57	*2.38*	9.68	4.12	*19.9*	**239**	20	0	11.95	0.327
	1990-2009	7.87	6.16	*6.62*	4.31	3.53	*2.37*	3.56	2.23	*5.65*	**204**	39	0	5.23	0.557
	2010-2020	6.26	4.99	*5.61*	3.85	3.46	*1.60*	2.41	1.50	*4.69*	**120**	21	0	5.71	0.669

**Table 7:** Percentage (numbers) of drugs in each time frame (n = 643 with ≥ 100 target comparator compounds) that are aliphatic, carboaromatic (aromatic heteroatoms = 0) and heteroaromatic (aromatic heteroatoms ≥ 1), and percentages of drugs with nAr ≥ 3.

Approval period	Aliphatic	Carboaromatic	Heteroaromatic
1939-1989	17% (43)	58% (151)	25% (65)
1990-2009	12% (29)	40% (98)	48% (116)
2010-2020	4% (5)	21% (29)	76% (107)
All drugs, % nAr ≥ 3		6% (17)	60% (171)

**Table 8:** Percentage of drugs having LE or LLE values < target median, between the target median and 90 percentile and > target 90 percentile, in each time frame.

Drug LE or LLE versus Target percentile	LLE < Median			LLE = Median - 90%			LLE > 90%		
	*1939-1989*	*1990-2009*	*2010-2020*	*1939-1989*	*1990-2009*	*2010-2020*	*1939-1989*	*1990-2009*	*2010-2020*
**LE > 90%**	3.9%	1.6%	0%	22%	13%	11%	32%	21%	14%
**LE = Median - 90%**	4.2%	3.7%	9.9%	18%	31%	34%	10%	16%	17%
**LE < Median**	1.9%	5.8%	5.7%	3.5%	6.2%	7.1%	3.5%	2.9%	1.4%

## Box 1 The three categories of drug and target properties and descriptors examined in this paper. The term **[drug - target median]** difference, used throughout, accounts for the variability between targets and target classes.

1) **Drug** properties.
2) **Target median** properties. Median property values for all compounds, including drugs, which act at each drug target.
3) **[Drug - target median]** property differences. The quantitative differences between the values of properties for each **drug** and the corresponding **target median**, i.e., **1) minus 2)**.

## Abbreviations

AB-MPS: AbbVie multiparameter score
ADMET: absorption, distribution, metabolism, excretion and toxicity
AEI: ADME efficiency index
ALogP: calculated LogP by atomic contribution
BEI: binding efficiency index;bRo5, beyond rule of 5
CGRP: calcitonin gene receptor protein
EGFR: epidermal growth factor receptor
FQ: fit quality
Fsp3: fraction of carbon atoms that are sp3 hybridised
HA: heavy atom
HBA: hydrogen bond acceptor
HBD: hydrogen bond donor
hERG: human ether-à-go-go-related gene
LE: ligand efficiency
LELP: LE adjusted for lipophilicity
LLE (or LipE): lipophilic ligand efficiency
LLEAT: LLE adjusted for heavy atom count
LogD_7.4_: calculated log of the distribution coefficient at pH7.4
nAr: number of aromatic rings
pChEMBL: target binding potency from the ChEMBL database
PSA: polar surface area
QED: quantitative estimate of druglikeness
Ro5: rule of 5
SEI: surface efficiency index
SILE: size-independent ligand efficiency
