# Supplementary material for: Target-based evaluation of ‘drug-like’ properties and ligand efficiencies
Source: J Med Chem. Author manuscript; Available in PMC 2021 Jun 11. (PMC7610969; doi:10.1021/acs.jmedchem.1c00416)
Supplement: Fig 5 values [file EMS123358-supplement-Fig_5_values.pdf]

Property	Approval period	Group	Total Count	Outlier Count	Mean Value	1st Quartile	Median	3rd Quartile	Lower Adjacent Limit	Upper Adjacent Limit	Standard Deviation	Confidence Interval (95%)
Mol Wt	1939-89	Drug	259	11	319.9	251.86	300.4	369.25	111.15	543.53	119.96	305.29-334.51
Mol Wt	1939-89	Target median	259	1	395.11	354.51	394.39	441.5	232.3	571.69	54.851	388.43-401.79
Mol Wt	1939-89	[Drug - target median]	259	16	-75.216	-133.01	-80.595	-35.335	-275.1	96	113.74	-89.068--61.364
Mol Wt	1990-2009	Drug	243	10	384.85	293.37	372.55	443.71	156.27	628.81	130.82	368.41-401.3
Mol Wt	1990-2009	Target median	243	11	413.62	357.67	399.48	451.57	227.27	571.69	76.12	404.05-423.19
Mol Wt	1990-2009	[Drug - target median]	243	9	-28.768	-84.56	-37.06	16.59	-236.24	144.65	107.32	-42.262--15.274
Mol Wt	2010-2020	Drug	141	7	452.14	387.21	447.89	502.79	223.3	631.6	115.82	433.02-471.26
Mol Wt	2010-2020	Target median	141	7	447.94	398.24	439.05	472.55	291.52	575.11	88.455	433.34-462.54
Mol Wt	2010-2020	[Drug - target median]	141	1	4.1956	-46.637	1.98	54.964	-171.23	201.26	70.396	-7.4241-15.815
ALogP	1939-89	Drug	259	3	2.6722	1.5875	2.7	3.8275	-1.42	6.94	1.6601	2.4701-2.8744
ALogP	1939-89	Target median	259	52	3.935	3.73	3.86	4.22	3.12	4.95	0.73445	3.8455-4.0244
ALogP	1939-89	[Drug - target median]	259	2	-1.2627	-2.2975	-1.17	-0.12	-4.78	3.1	1.5319	-1.4493--1.0762
ALogP	1990-2009	Drug	243	10	3.0482	2.0125	3.07	4.2075	-1.27	7.26	1.9544	2.8025-3.2939
ALogP	1990-2009	Target median	243	51	3.8731	3.575	3.93	4.34	2.49	5.39	1.2123	3.7207-4.0255
ALogP	1990-2009	[Drug - target median]	243	3	-0.82492	-1.7475	-0.885	0.02	-4.27	2.16	1.3958	-1.0004--0.64942
ALogP	2010-2020	Drug	141	2	3.6033	2.5725	3.75	4.755	-0.14	7.69	1.6886	3.3245-3.882
ALogP	2010-2020	Target median	141	4	3.7657	3.11	3.92	4.34	1.65	5.755	1.026	3.5964-3.9351
ALogP	2010-2020	[Drug - target median]	141	1	-0.16248	-0.9025	-0.09	0.70875	-2.47	2.64	1.1074	-0.34527-0.020309
PSA	1939-89	Drug	259	14	61.496	34.59	52.93	75.765	3.24	132.96	47.612	55.698-67.295
PSA	1939-89	Target median	259	4	64.434	50.08	59.08	77.009	29.54	116.56	22.084	61.744-67.123
PSA	1939-89	[Drug - target median]	259	13	-2.9376	-27.83	-11.8	10.475	-82.57	67.46	42.15	-8.071-2.1958
PSA	1990-2009	Drug	243	8	81.6	46.972	72.72	104.79	3.24	191.26	48.695	75.477-87.723
PSA	1990-2009	Target median	243	0	74.842	51.37	68.01	94.063	29.54	142.43	27.443	71.391-78.292
PSA	1990-2009	[Drug - target median]	243	12	6.7582	-13.13	-0.32	17.294	-54.9	57.57	38.022	1.9775-11.539
PSA	2010-2020	Drug	141	10	93.791	74.53	90.45	110.24	26.79	156.89	37.406	87.617-99.965
PSA	2010-2020	Target median	141	9	91.807	79.042	91.405	104.05	43.6	137.36	27.995	87.186-96.428
PSA	2010-2020	[Drug - target median]	141	3	1.9842	-15.795	0	14.578	-43.015	52.56	24.561	-2.0699-6.0383
Ar rings, carboaromatics	1939-89	Drug	151	0	1.4901	1	1	2	1	3	0.55218	1.402-1.5781
Ar rings, carboaromatics	1939-89	Target median	151	0	2.404	2	2	3	1	3	0.5187	2.3212-2.4867
Ar rings, carboaromatics	1939-89	[Drug - target median]	151	0	-0.91391	-1	-1	0	-2	1	0.72975	-1.0303--0.79751
Ar rings, carboaromatics	1990-2009	Drug	98	0	1.6327	1	2	2	1	3	0.66407	1.5012-1.7641
Ar rings, carboaromatics	1990-2009	Target median	98	0	2.398	2	2	3	1	4	0.63797	2.2716-2.5243
Ar rings, carboaromatics	1990-2009	[Drug - target median]	98	0	-0.76531	-1	-1	0	-2	1	0.82238	-0.92813--0.60248
Ar rings, carboaromatics	2010-2020	Drug	29	0	1.6897	1	2	2	1	3	0.66027	1.4493-1.93
Ar rings, carboaromatics	2010-2020	Target median	29	1	2.4828	2	2	3	2	4	0.73779	2.2142-2.7513
Ar rings, carboaromatics	2010-2020	[Drug - target median]	29	0	-0.7931	-1	-1	0	-2	1	0.77364	-1.0747--0.51153
Ar rings, heteroaromatics	1939-89	Drug	65	0	2.1077	2	2	3	1	4	0.81246	1.9102-2.3052
Ar rings, heteroaromatics	1939-89	Target median	65	1	2.4462	2	2	3	1	4	0.63813	2.291-2.6013
Ar rings, heteroaromatics	1939-89	[Drug - target median]	65	2	-0.33846	-1	0	0	-2	1	0.87101	-0.55021--0.12671
Ar rings, heteroaromatics	1990-2009	Drug	116	6	2.7328	2	3	3	1	4	0.96331	2.5575-2.9081
Ar rings, heteroaromatics	1990-2009	Target median	116	6	2.7759	2	3	3	1	4	0.87562	2.6165-2.9352
Ar rings, heteroaromatics	1990-2009	[Drug - target median]	116	0	-0.0431	-1	0	1	-2	2	0.89823	-0.20657-0.12036
Ar rings, heteroaromatics	2010-2020	Drug	107	4	3.1682	3	3	4	2	5	0.91616	2.9946-3.3418
Ar rings, heteroaromatics	2010-2020	Target median	107	0	3.0888	3	3	4	2	5	0.6964	2.9568-3.2207
Ar rings, heteroaromatics	2010-2020	[Drug - target median]	107	44	0.079439	0	0	0	0	0	0.77444	-0.067302-0.22618
Fsp3	1939-89	Drug	259	0	0.4532	0.29	0.43	0.61	0	1	0.23095	0.42508-0.48133
Fsp3	1939-89	Target median	259	2	0.34033	0.2625	0.35	0.41	0.1	0.57	0.11015	0.32691-0.35374
Fsp3	1939-89	[Drug - target median]	259	3	0.11288	-0.05	0.07	0.24	-0.37	0.62	0.21297	0.086939-0.13881
Fsp3	1990-2009	Drug	243	10	0.43514	0.29	0.4	0.5475	0	0.89	0.21411	0.40822-0.46207
Fsp3	1990-2009	Target median	243	3	0.34586	0.25	0.36	0.41	0.13	0.64	0.1204	0.33073-0.361
Fsp3	1990-2009	[Drug - target median]	243	7	0.08928	-0.04	0.07	0.19	-0.35	0.52	0.18826	0.065609-0.11295
Fsp3	2010-2020	Drug	141	3	0.36631	0.2475	0.35	0.47	0	0.8	0.17739	0.33703-0.39559
Fsp3	2010-2020	Target median	141	0	0.32323	0.24	0.31	0.4	0.09	0.64	0.11207	0.30473-0.34172
Fsp3	2010-2020	[Drug - target median]	141	6	0.043085	-0.0425	0.03	0.1125	-0.23	0.31	0.14801	0.018654-0.067517
QED	1939-89	Drug	259	10	0.67432	0.59	0.7	0.81	0.26	0.92	0.17684	0.65279-0.69586
QED	1939-89	Target median	259	16	0.57973	0.52	0.61	0.66	0.31	0.82	0.13365	0.56345-0.59601
QED	1939-89	[Drug - target median]	259	8	0.094595	-0.01	0.1	0.2	-0.29	0.5	0.17964	0.072716-0.11647
QED	1990-2009	Drug	243	0	0.58597	0.4325	0.62	0.77	0.07	0.93	0.21928	0.5584-0.61354
QED	1990-2009	Target median	243	0	0.53276	0.385	0.57	0.65	0.18	0.87	0.16187	0.51241-0.55311
QED	1990-2009	[Drug - target median]	243	15	0.05321	-0.02	0.07	0.16	-0.29	0.39	0.16804	0.032081-0.074338
QED	2010-2020	Drug	141	0	0.50603	0.36	0.49	0.6525	0.05	0.93	0.19568	0.47373-0.53833
QED	2010-2020	Target median	141	0	0.49082	0.39	0.5	0.59	0.12	0.81	0.14553	0.46679-0.51484
QED	2010-2020	[Drug - target median]	141	2	0.015213	-0.0725	0.02	0.12	-0.34	0.38	0.14546	0.0087971-0.039223
pCHEMBL	1939-89	Drug	259	0	7.4828	6.4825	7.82	8.5975	4	10.57	1.4566	7.3054-7.6602
pCHEMBL	1939-89	Target median	259	0	6.6232	6.12	6.73	7.09	4.81	8.1	0.71571	6.5361-6.7104
pCHEMBL	1939-89	[Drug - target median]	259	3	0.85954	0.1625	1	1.72	-2.1	3.84	1.2716	0.70468-1.0144
pCHEMBL	1990-2009	Drug	243	0	7.8189	6.99	8	8.6975	4.52	10.86	1.2679	7.6595-7.9784
pCHEMBL	1990-2009	Target median	243	0	6.8476	6.285	6.82	7.37	5.14	8.23	0.75721	6.7524-6.9428
pCHEMBL	1990-2009	[Drug - target median]	243	5	0.97132	0.32	0.98	1.7375	-1.63	3.805	1.1219	0.83026-1.1124
pCHEMBL	2010-2020	Drug	141	4	8.3677	7.85	8.47	9	6.24	10.57	0.94583	8.2116-8.5239
pCHEMBL	2010-2020	Target median	141	9	7.0817	6.8175	7.14	7.49	6.04	8.22	0.61243	6.9806-7.1828
pCHEMBL	2010-2020	[Drug - target median]	141	3	1.286	0.8075	1.31	1.8575	-0.46	3.43	0.86931	1.1425-1.4295
LE	1939-89	Drug	259	11	0.49262	0.404	0.491	0.56775	0.161	0.779	0.14819	0.47457-0.51067
LE	1939-89	Target median	259	4	0.3297	0.2915	0.321	0.355	0.211	0.432	0.057466	0.3227-0.3367
LE	1939-89	[Drug - target median]	259	6	0.16292	0.077	0.158	0.23225	-0.137	0.454	0.13453	0.14653-0.1793
LE	1990-2009	Drug	243	6	0.42886	0.345	0.402	0.5105	0.13	0.703	0.12993	0.41253-0.4452
LE	1990-2009	Target median	243	5	0.32914	0.28825	0.324	0.357	0.223	0.437	0.060604	0.32152-0.33676
LE	1990-2009	[Drug - target median]	243	6	0.099728	0.0275	0.0855	0.1625	-0.156	0.3445	0.10558	0.086453-0.113
LE	2010-2020	Drug	141	5	0.37895	0.31275	0.367	0.43	0.208	0.587	0.098581	0.36268-0.39522
LE	2010-2020	Target median	141	4	0.31251	0.285	0.306	0.347	0.206	0.432	0.046808	0.30479-0.32024
LE	2010-2020	[Drug - target median]	141	6	0.066436	0.019	0.057	0.08925	-0.059	0.191	0.077506	0.053643-0.079229
LLE	1939-89	Drug	259	7	4.8098	3.6312	4.85	5.995	0.2	9.18	1.9755	4.5692-5.0504
LLE	1939-89	Target median	259	19	2.7247	2.31	2.7525	3.205	1.01	4.525	0.92693	2.6118-2.8376
LLE	1939-89	[Drug - target median]	259	8	2.0852	1.0425	2.035	3.1775	-1.72	6.11	1.7479	1.8723-2.298
LLE	1990-2009	Drug	243	4	4.7698	3.4525	4.78	5.9525	0.27	9.03	1.9903	4.5196-5.0201
LLE	1990-2009	Target median	243	7	3.0134	2.315	2.845	3.61	0.45	5.26	1.1587	2.8677-3.1591
LLE	1990-2009	[Drug - target median]	243	5	1.7564	0.7825	1.8	2.7075	-1.73	5.145	1.4989	1.5679-1.9449
LLE	2010-2020	Drug	141	2	4.7635	3.5138	4.84	5.9212	0.625	8.99	1.8364	4.4604-5.0666
LLE	2010-2020	Target median	141	10	3.3422	2.7175	3.23	3.89	1.33	5.25	1.0957	3.1614-3.5231
LLE	2010-2020	[Drug - target median]	141	1	1.4213	0.44375	1.44	2.32	-1.99	4.84	1.3358	1.2008-1.6417
