# Supplementary material for: Target-based evaluation of ‘drug-like’ properties and ligand efficiencies
Source: J Med Chem. Author manuscript; Available in PMC 2021 Jun 11. (PMC7610969; doi:10.1021/acs.jmedchem.1c00416)
Supplement: Fig 6 values [file EMS123358-supplement-Fig_6_values.pdf]

ALogP	Approval period	Target class	Group	Total Count	Outlier Count	Mean Value	1st Quartile	Median	3rd Quartile	Lower Adjacent Limit	Upper Adjacent Limit	Standard Deviation	Confidence Interval (95%)
	1939-89	Enzyme_Hydrolase	Drug	7	0	1.4143	0.3825	1.77	2.145	0.15	2.91	1.0653	0.62507-2.2035
	1939-89	Enzyme_Hydrolase	Target median	7	1	4.2907	4.41	4.41	4.41	4.41	4.41	0.3156	4.0569-4.5245
	1939-89	Enzyme_Hydrolase	[Drug - target median]	7	0	-2.8764	-3.7362	-2.64	-2.265	-4.09	-1.5	0.93951	-3.5724--2.1804
	1939-89	Enzyme_Other	Drug	32	2	0.76938	-0.5	1.34	1.34	-1.42	3.59	1.5342	0.2378-1.3009
	1939-89	Enzyme_Other	Target median	32	0	2.3155	1.67	1.91	2.8875	1.27	4.41	1.0085	1.9661-2.6649
	1939-89	Enzyme_Other	[Drug - target median]	32	0	-1.5461	-2.77	-1.41	-0.33	-4.03	0.62	1.3771	-2.0232--1.0689
	1939-89	Enzyme_Oxidoreductase	Drug	46	0	2.7363	1.58	3.04	3.75	-1.06	6.83	1.4915	2.3053-3.1673
	1939-89	Enzyme_Oxidoreductase	Target median	46	5	3.6902	3.665	3.78	3.93	3.61	4.07	0.37844	3.5809-3.7996
	1939-89	Enzyme_Oxidoreductase	[Drug - target median]	46	1	-0.95391	-2.04	-0.84	-0.03	-3.34	1.42	1.3644	-1.3482--0.55961
	1939-89	Enzyme_Phosphodiesterase	Drug	9	0	1.5244	-0.02	1.62	3.3	-1.04	3.3	1.8149	0.33869-2.7102
	1939-89	Enzyme_Phosphodiesterase	Target median	9	0	3.9689	3.5512	3.8	4.365	3.37	4.98	0.5517	3.6084-4.3293
	1939-89	Enzyme_Phosphodiesterase	[Drug - target median]	9	0	-2.4444	-3.4937	-2.18	-1.245	-4.775	-0.6	1.4209	-3.3728--1.5161
	1939-89	Enzyme_Protease	Drug	7	0	0.53286	-0.115	0.63	1.0575	-0.35	1.24	0.6484	0.052515-1.0132
	1939-89	Enzyme_Protease	Target median	7	0	2.55	2.32	2.4	2.8363	2.32	2.855	0.26462	2.354-2.746
	1939-89	Enzyme_Protease	[Drug - target median]	7	0	-2.0171	-2.6288	-2.015	-1.315	-3.13	-1.08	0.76446	-2.5835--1.4508
	1939-89	Ion channel	Drug	24	1	3.0233	2.03	2.97	3.415	1.85	5.09	1.168	2.5561-3.4906
	1939-89	Ion channel	Target median	24	2	3.651	3.47	3.78	4.065	2.67	4.75	0.74246	3.354-3.9481
	1939-89	Ion channel	[Drug - target median]	24	0	-0.62771	-1.77	-0.67	0.17	-2.57	2.97	1.2492	-1.1275--0.12792
	1939-89	Membrane receptor_GPCR_amine	Drug	180	0	2.6084	1.555	2.425	3.745	-0.77	5.89	1.4877	2.3911-2.8258
	1939-89	Membrane receptor_GPCR_amine	Target median	180	2	3.7798	3.625	3.82	4.01	3.12	4.26	0.33421	3.731-3.8287
	1939-89	Membrane receptor_GPCR_amine	[Drug - target median]	180	0	-1.1714	-2.06	-1.2025	-0.205	-4.52	2.15	1.4109	-1.3775--0.96527
	1939-89	Membrane receptor_GPCR_other	Drug	23	0	0.76565	-1.04	-1.03	3.1975	-1.98	6.26	2.5656	-0.28288-1.8142
	1939-89	Membrane receptor_GPCR_other	Target median	23	0	3.5398	2.78	2.8	4.7487	2.64	5.795	1.1347	3.0761-4.0035
	1939-89	Membrane receptor_GPCR_other	[Drug - target median]	23	0	-2.7741	-3.8375	-3.79	-1.5538	-4.78	0.87	1.665	-3.4546--2.0937
	1939-89	Membrane receptor_GPCR_peptide	Drug	31	2	1.8787	1.3	1.53	3.08	0.75	5.09	1.6859	1.2852-2.4722
	1939-89	Membrane receptor_GPCR_peptide	Target median	31	2	4.2074	4.12	4.22	4.22	4.1	4.22	0.29649	4.103-4.3118
	1939-89	Membrane receptor_GPCR_peptide	[Drug - target median]	31	2	-2.3287	-2.895	-2.59	-1.14	-3.47	0.87	1.7872	-2.9579--1.6996
	1939-89	Other	Drug	7	1	3.1929	2.9975	3.4	3.78	2.87	3.99	0.973	2.4721-3.9137
	1939-89	Other	Target median	7	0	3.4386	2.93	4	4.06	1.79	4.08	0.87492	2.7904-4.0867
	1939-89	Other	[Drug - target median]	7	0	-0.24571	-0.635	-0.6	0.3225	-1.21	0.98	0.75006	-0.80137-0.30994
	1939-89	Transcription factor_NHR	Drug	46	0	3.542	2.56	3.56	4.61	0.62	5.7	1.2635	3.1768-3.9071
	1939-89	Transcription factor_NHR	Target median	46	16	5.0433	4.77	5.06	5.06	4.475	5.06	0.50284	4.8979-5.1886
	1939-89	Transcription factor_NHR	[Drug - target median]	46	0	-1.5013	-2.5	-1.57	-0.365	-4.44	0.93	1.2712	-1.8687--1.1339
	1939-89	Transporter	Drug	35	1	3.2434	2.61	3.43	3.88	1.58	5.46	1.0409	2.8986-3.5883
	1939-89	Transporter	Target median	35	1	3.7866	3.73	3.83	3.99	3.73	4.05	0.44461	3.6393-3.9339
	1939-89	Transporter	[Drug - target median]	35	0	-0.54314	-1.275	-0.43	0.1375	-2.35	1.41	0.87864	-0.83424--0.25205
	1990-2009	Enzyme_Hydrolase	Drug	19	0	0.73789	-0.33	0.91	2.715	-4.49	4.36	2.7062	-0.47895-1.9547
	1990-2009	Enzyme_Hydrolase	Target median	19	0	2.6489	0.42	3.575	4.41	-1.45	4.85	2.1234	1.6941-3.6038
	1990-2009	Enzyme_Hydrolase	[Drug - target median]	19	0	-1.9111	-3.1188	-1.94	-0.8725	-4	0.495	1.5244	-2.5965--1.2256
	1990-2009	Enzyme_Kinase	Drug	51	0	4.0278	3.31	3.33	5.31	3.14	6.36	1.1072	3.724-4.3317
	1990-2009	Enzyme_Kinase	Target median	51	0	4.4032	4.23	4.36	4.5925	3.85	5.09	0.23555	4.3386-4.4679
	1990-2009	Enzyme_Kinase	[Drug - target median]	51	0	-0.37539	-1.1587	-0.93	0.7425	-1.78	2.16	1.126	-0.68442--0.06636
	1990-2009	Enzyme_Other	Drug	12	0	2.2683	0.35	2.505	3.48	-0.4	6.18	2.0606	1.1024-3.4342
	1990-2009	Enzyme_Other	Target median	12	0	2.8667	1.785	2.9475	3.815	1.66	4.045	1.0827	2.2541-3.4793
	1990-2009	Enzyme_Other	[Drug - target median]	12	0	-0.59833	-1.3625	-0.9675	0.2425	-2.31	2.135	1.2556	-1.3087-0.11207
	1990-2009	Enzyme_Oxidoreductase	Drug	32	2	3.2559	2.73	3.07	3.92	1.4	5.14	1.1254	2.866-3.6459
	1990-2009	Enzyme_Oxidoreductase	Target median	32	5	3.8794	3.7225	3.93	4.37	3.61	4.87	0.76682	3.6137-4.1451
	1990-2009	Enzyme_Oxidoreductase	[Drug - target median]	32	0	-0.62344	-1.205	-0.69	0.22	-2.47	1.44	1.0859	-0.99968--0.2472
	1990-2009	Enzyme_Phosphodiesterase	Drug	4	0	1.955	1.77	2	2.14	1.61	2.21	0.25684	1.7033-2.2067
	1990-2009	Enzyme_Phosphodiesterase	Target median	4	0	3.2487	3.1275	3.37	3.37	2.885	3.37	0.2425	3.0111-3.4864
	1990-2009	Enzyme_Phosphodiesterase	[Drug - target median]	4	0	-1.2938	-1.53	-1.23	-1.0575	-1.76	-0.955	0.3416	-1.6285--0.95898
	1990-2009	Enzyme_Protease	Drug	19	0	2.6547	1.355	2.4	3.9425	0.4	5.91	1.5002	1.9802-3.3293
	1990-2009	Enzyme_Protease	Target median	19	0	3.3305	2.32	3.49	4.3	2.25	4.3	0.94802	2.9042-3.7568
	1990-2009	Enzyme_Protease	[Drug - target median]	19	0	-0.67579	-1.7225	-0.91	-0.125	-1.92	1.84	1.1262	-1.1822--0.16937
	1990-2009	Enzyme_Transferase	Drug	9	0	-0.31556	-1.315	-0.37	0.5425	-1.66	1.98	1.2042	-1.1023-0.47116
	1990-2009	Enzyme_Transferase	Target median	9	0	1.7333	1.29	1.29	2.52	1.29	2.82	0.67062	1.2952-2.1715
	1990-2009	Enzyme_Transferase	[Drug - target median]	9	0	-2.0489	-2.6575	-1.85	-1.255	-4.27	-0.54	1.151	-2.8008--1.2969
	1990-2009	Ion channel	Drug	25	5	2.7132	2.01	2.44	2.8	1.77	3.79	1.1881	2.2475-3.1789
	1990-2009	Ion channel	Target median	25	0	3.8024	3.27	3.97	4.37	2.14	5.23	0.81572	3.4826-4.1222
	1990-2009	Ion channel	[Drug - target median]	25	0	-1.0892	-1.87	-1.21	-0.0025	-3.92	0.7	1.1922	-1.5566--0.62184
	1990-2009	Membrane receptor_GPCR_amine	Drug	107	0	2.9658	1.91	2.86	3.81	0.7	5.63	1.1754	2.7431-3.1885
	1990-2009	Membrane receptor_GPCR_amine	Target median	107	6	3.802	3.725	3.84	4.0175	3.29	4.26	0.33499	3.7385-3.8654
	1990-2009	Membrane receptor_GPCR_amine	[Drug - target median]	107	0	-0.83617	-1.6625	-0.86	-0.0725	-3.05	1.81	1.133	-1.0508--0.62149
	1990-2009	Membrane receptor_GPCR_other	Drug	17	2	3.6541	2.56	3.09	4.8975	2.53	6.14	2.3782	2.5236-4.7846
	1990-2009	Membrane receptor_GPCR_other	Target median	17	0	4.5465	3.23	4.6	5.0825	2.64	7.54	1.6781	3.7487-5.3442
	1990-2009	Membrane receptor_GPCR_other	[Drug - target median]	17	1	-0.89235	-1.585	-0.7	-0.1	-2.91	1.47	1.5822	-1.6445--0.14024
	1990-2009	Membrane receptor_GPCR_peptide	Drug	27	1	3.8381	2.735	4.16	5.205	0.42	7.26	2.1074	3.0432-4.6331
	1990-2009	Membrane receptor_GPCR_peptide	Target median	27	0	4.8463	4.22	4.52	5.68	3.71	5.72	0.75631	4.561-5.1316
	1990-2009	Membrane receptor_GPCR_peptide	[Drug - target median]	27	1	-1.0081	-1.59	-1.03	-0.1925	-3.29	1.54	1.6094	-1.6152--0.40108
	1990-2009	Other	Drug	20	0	1.88	0.89	1.13	2.645	0.82	4.75	1.1971	1.3554-2.4046
	1990-2009	Other	Target median	20	3	3.3283	3.08	3.32	3.58	2.8	4.08	0.70146	3.0208-3.6357
	1990-2009	Other	[Drug - target median]	20	0	-1.4483	-2.3925	-1.545	-0.825	-3.59	0.98	1.2077	-1.9775--0.91896
	1990-2009	Transcription factor_NHR	Drug	36	0	5.2861	4.34	5.6	6.1	2.45	8.68	1.5615	4.776-5.7962
	1990-2009	Transcription factor_NHR	Target median	36	0	5.2985	4.77	5.575	5.96	4.085	6.08	0.68929	5.0733-5.5236
	1990-2009	Transcription factor_NHR	[Drug - target median]	36	2	-0.01236	-1.065	-0.36	0.7175	-3.23	2.07	1.6162	-0.54032-0.5156
	1990-2009	Transporter	Drug	24	0	3.3837	2.73	3.425	4.22	1.77	5.18	1.0309	2.9713-3.7962
	1990-2009	Transporter	Target median	24	2	3.8242	3.73	3.93	3.99	3.73	3.99	0.23526	3.73-3.9183
	1990-2009	Transporter	[Drug - target median]	24	0	-0.44042	-1.13	-0.36	0.5	-2.22	1.19	1.0413	-0.85703--0.023803
	2010-2020	Enzyme_Hydrolase	Drug	5	0	0.604	-0.835	-0.14	2.525	-2.92	4.28	2.6844	-1.749-2.957
	2010-2020	Enzyme_Hydrolase	Target median	5	0	1.941	0.40875	1.65	3.24	0.375	4.41	1.7156	0.43724-3.4448
	2010-2020	Enzyme_Hydrolase	[Drug - target median]	5	0	-1.337	-2.995	-0.56	-0.02875	-4.57	1.43	2.2733	-3.3296-0.65564
	2010-2020	Enzyme_Kinase	Drug	116	2	4.3662	3.62	4.77	5.19	1.54	6.36	1.2462	4.1394-4.593
	2010-2020	Enzyme_Kinase	Target	116	0	4.1734	3.885	4.3	4.55	3.01	5.395	0.52477	4.0779-4.2689
	2010-2020	Enzyme_Kinase	[Drug - target median]	116	0	0.1928	-0.58	0.285	0.97	-2.12	2.76	1.0459	0.0024668-0.38314
	2010-2020	Enzyme_Oxidoreductase	Drug	6	0	3.385	2.43	3.34	4.32	2.37	4.51	0.92923	2.6415-4.1285
	2010-2020	Enzyme_Oxidoreductase	Target median	6	0	4.0375	3.68	3.85	4.39	3.665	4.79	0.45611	3.6725-4.4025
	2010-2020	Enzyme_Oxidoreductase	[Drug - target median]	6	0	-0.6525	-1.34	-0.7725	-0.07	-1.79	0.83	0.98777	-1.4429-0.13788
	2010-2020	Enzyme_Phosphodiesterase	Drug	7	0	3.8329	2.43	5.03	5.03	1.85	5.03	1.5056	2.7175-4.9482
	2010-2020	Enzyme_Phosphodiesterase	Target median	7	0	3.9357	3.735	3.9	4.1813	3.37	4.635	0.40973	3.6322-4.2392
	2010-2020	Enzyme_Phosphodiesterase	[Drug - target median]	7	0	-0.10286	-1.4288	0.395	1.0362	-1.52	1.295	1.2767	-1.0487-0.84296
	2010-2020	Enzyme_Protease	Drug	19	0	2.3153	1.1425	1.71	3.7125	0.2	5.25	1.6052	1.5935-3.0371
	2010-2020	Enzyme_Protease	Target median	19	0	2.7279	2.25	2.3	3.63	2.25	3.66	0.65244	2.4345-3.0213
	2010-2020	Enzyme_Protease	[Drug - target median]	19	0	-0.41263	-1.485	-0.74	0.425	-2.37	2.95	1.4271	-1.0543-0.22908
	2010-2020	Enzyme_Transferase	Drug	14	2	2.7086	2.35	2.63	3.43	2.35	4.48	1.0692	2.1485-3.2687
	2010-2020	Enzyme_Transferase	Target median	14	0	2.7207	2.47	2.57	2.98	2.47	3.13	0.25138	2.589-2.8524
	2010-2020	Enzyme_Transferase	[Drug - target median]	14	2	-0.01214	-0.12	0.14	0.61	-0.22	1.35	1.0713	-0.57332-0.54903
	2010-2020	Ion channel	Drug	6	1	5.0583	3.86	4.675	5.08	3.4	5.08	1.8689	3.5629-6.5538
	2010-2020	Ion channel	Target median	6	1	4.4433	3.6	4.34	4.34	2.67	4.34	1.5782	3.1805-5.7061
	2010-2020	Ion channel	[Drug - target median]	6	1	0.615	0.41	0.87	1.19	0.41	1.29	0.82585	-0.045819-1.2758
	2010-2020	Membrane receptor_GPCR_amine	Drug	15	0	3.5567	2.76	3.86	4.2975	2.09	5.14	0.90915	3.0966-4.0168
	2010-2020	Membrane receptor_GPCR_amine	Target median	15	0	3.7713	3.47	3.86	4.07	3.04	4.26	0.36785	3.5852-3.9575
	2010-2020	Membrane receptor_GPCR_amine	[Drug - target median]	15	0	-0.21467	-0.7	-0.09	0.25	-1.15	0.88	0.60127	-0.51895-0.089618
	2010-2020	Membrane receptor_GPCR_other	Drug	9	0	3.7522	2.6675	3.32	4.3875	2.01	6.77	1.5981	2.7081-4.7963
	2010-2020	Membrane receptor_GPCR_other	Target median	9	0	4.0367	3.11	4.13	4.6725	2.78	5.39	0.88942	3.4556-4.6178
	2010-2020	Membrane receptor_GPCR_other	[Drug - target median]	9	1	-0.28444	-1.085	-0.66	-0.08	-1.25	0.46	1.2146	-1.078-0.50907
	2010-2020	Membrane receptor_GPCR_peptide	Drug	14	0	4.5586	3.75	4.325	5.63	1.84	6.79	1.2601	3.8985-5.2186
	2010-2020	Membrane receptor_GPCR_peptide	Target median	14	0	4.5686	4.005	4.285	5.43	3.75	5.69	0.71605	4.1935-4.9437
	2010-2020	Membrane receptor_GPCR_peptide	[Drug - target median]	14	0	-0.01	-0.78	0.19	0.705	-2.13	1.815	1.0648	-0.56779-0.54779
	2010-2020	Other	Drug	26	1	2.9335	2.01	3.33	3.33	2.01	4.5	1.2198	2.4646-3.4023
	2010-2020	Other	Target median	26	1	3.3454	3.09	3.24	3.34	2.87	3.58	0.69804	3.0771-3.6137
	2010-2020	Other	[Drug - target median]	26	0	-0.41192	-1.2	-0.175	0.13	-1.57	1.12	0.80687	-0.72208--0.10177
	2010-2020	Transcription factor_NHR	Drug	4	0	4.545	3.76	4.55	5.33	3.53	5.55	0.94296	3.6209-5.4691
	2010-2020	Transcription factor_NHR	Target median	4	0	4.8688	4.085	4.8175	5.6525	4.085	5.755	0.90886	3.9781-5.7594
	2010-2020	Transcription factor_NHR	[Drug - target median]	4	0	-0.32375	-0.6	-0.325	-0.0475	-0.645	0	0.32343	-0.64071--0.0067897
	2010-2020	Transporter	Drug	11	0	2.4182	1.65	1.84	3.6375	1	4.24	1.1546	1.7359-3.1005
	2010-2020	Transporter	Target median	11	0	2.93	2.08	2.08	3.99	2.08	4.05	0.97981	2.351-3.509
	2010-2020	Transporter	[Drug - target median]	11	0	-0.51182	-0.99	-0.24	0.0625	-2.22	0.89	0.93292	-1.0631-0.039501

Approval period	Therapeutic use	Group	Total Count	Outlier Count	Mean Value	1st Quartile	Median	3rd Quartile	Lower Adjacent Limit	Upper Adjacent Limit	Standard Deviation	Confidence Interval (95%)
1939-1989	Cancer	Drug	11	0	2.5536	0.5375	2.32	4.38	-0.14	5.7	2.136	1.2913-3.8159
1939-1989	Cancer	Target median	11	1	4.0786	3.8338	4.08	4.41	3.815	4.95	0.58586	3.7324-4.4249
1939-1989	Cancer	[Drug - target median]	11	0	-1.525	-2.9225	-1.765	0.0525	-4.03	0.93	1.7758	-2.5744--0.47555
1939-1989	Cardiovascular	Drug	46	3	2.0561	1.15	1.97	2.96	-0.02	5.3	1.679	1.5709-2.5413
1939-1989	Cardiovascular	Target median	46	13	3.6678	3.29	3.645	3.86	2.76	4.475	0.99701	3.3797-3.9559
1939-1989	Cardiovascular	[Drug - target median]	46	4	-1.6117	-2.18	-1.65	-0.9	-3.2	0.375	1.5928	-2.072--1.1514
1939-1989	Central nervous system	Drug	74	0	2.9846	1.86	3.335	4.21	-1.03	5.89	1.4751	2.6485-3.3207
1939-1989	Central nervous system	Target median	74	4	3.8719	3.73	3.99	4.09	3.23	4.41	0.40403	3.7798-3.9639
1939-1989	Central nervous system	[Drug - target median]	74	0	-0.8873	-2.01	-0.505	0.22	-4.47	1.88	1.4658	-1.2213--0.55333
1939-1989	Gastrointestinal & metabolism	Drug	18	0	3.2167	1.56	3.34	4.48	-0.77	6.83	2.0416	2.2735-4.1598
1939-1989	Gastrointestinal & metabolism	Target median	18	4	4.1375	3.75	3.97	4.22	3.73	4.26	0.72182	3.804-4.471
1939-1989	Gastrointestinal & metabolism	[Drug - target median]	18	0	-0.92083	-2.29	-0.94	0.55	-4.52	3.1	1.8275	-1.7651--0.076563
1939-1989	Genito urinary	Drug	16	0	3.5463	3.145	3.55	4.605	1.06	4.83	1.0613	3.0262-4.0663
1939-1989	Genito urinary	Target median	16	0	4.4269	4.085	4.515	4.86	3.31	5.085	0.51976	4.1722-4.6816
1939-1989	Genito urinary	[Drug - target median]	16	0	-0.88063	-1.425	-0.935	-0.2175	-2.66	0.525	0.81705	-1.281--0.48027
1939-1989	Infection	Drug	5	0	1.526	0.025	1.53	2.835	-0.35	3.78	1.6942	0.040964-3.011
1939-1989	Infection	Target median	5	0	2.995	2.73	2.84	3.3837	2.4	3.575	0.45937	2.5923-3.3977
1939-1989	Infection	[Drug - target median]	5	0	-1.469	-2.9188	-1.31	-0.125	-3.425	0.46	1.6228	-2.8914--0.046587
1939-1989	Multiple/other	Drug	32	1	2.2341	1.57	2.005	3.255	-0.09	5.7	1.5737	1.6888-2.7793
1939-1989	Multiple/other	Target median	32	2	4.1262	3.78	3.85	4.49	3.29	5.06	0.70413	3.8823-4.3702
1939-1989	Multiple/other	[Drug - target median]	32	0	-1.8922	-3.16	-1.98	-0.595	-6.23	0.58	1.605	-2.4483--1.3361
1939-1989	Musculo-skeletal	Drug	15	1	3.416	2.93	3.67	4.07	2.19	5.46	1.5454	2.6339-4.1981
1939-1989	Musculo-skeletal	Target median	15	1	3.7887	3.78	3.93	3.93	3.78	4.08	0.43084	3.5706-4.0067
1939-1989	Musculo-skeletal	[Drug - target median]	15	1	-0.37267	-1.075	-0.18	0.175	-1.59	1.42	1.2117	-0.98586-0.24053
1939-1989	Respiratory	Drug	23	1	2.8461	2.1125	2.85	3.715	1.13	5.35	1.3957	2.2757-3.4165
1939-1989	Respiratory	Target median	23	6	3.8663	3.82	3.82	3.86	3.82	3.86	0.38399	3.7094-4.0232
1939-1989	Respiratory	[Drug - target median]	23	0	-1.0202	-1.9125	-0.975	-0.1175	-3.82	1.53	1.3373	-1.5668--0.47366
1939-1989	Skin & eye	Drug	19	0	2.0063	0.53	2.37	2.9175	-1.42	5.6	1.7458	1.2213-2.7913
1939-1989	Skin & eye	Target median	19	3	4.2618	3.86	5.06	5.06	3.34	5.965	1.2396	3.7044-4.8193
1939-1989	Skin & eye	[Drug - target median]	19	0	-2.2555	-2.75	-2.14	-1.5075	-4.44	-0.365	1.0469	-2.7263--1.7848
1990-2009	Cancer	Drug	30	1	3.48	2.45	3.2	4.59	0.42	6.36	1.832	2.8244-4.1356
1990-2009	Cancer	Target median	30	4	3.9125	3.58	4.08	4.34	2.52	4.77	0.88567	3.5956-4.2294
1990-2009	Cancer	[Drug - target median]	30	2	-0.4325	-1.135	-0.8575	0.215	-2.69	2.16	1.4468	-0.95023-0.085229
1990-2009	Cardiovascular	Drug	37	1	3.3254	2.0625	3.74	4.5475	0.4	7.26	1.9396	2.7004-3.9504
1990-2009	Cardiovascular	Target median	37	0	4.2286	3.6962	4.35	5.1225	1.79	5.72	1.221	3.8352-4.6221
1990-2009	Cardiovascular	[Drug - target median]	37	1	-0.90324	-1.76	-0.97	0.015	-3.05	1.84	1.429	-1.3637--0.44277
1990-2009	Central nervous system	Drug	57	0	2.7772	1.9175	2.73	3.56	-0.4	5.18	1.1401	2.4812-3.0732
1990-2009	Central nervous system	Target median	57	9	3.8527	3.78	3.99	4.09	3.34	4.41	0.58903	3.6998-4.0056
1990-2009	Central nervous system	[Drug - target median]	57	0	-1.0755	-1.92	-1.14	-0.1525	-3.92	1.19	1.1376	-1.3709--0.78019
1990-2009	Gastrointestinal & metabolism	Drug	23	4	2.4448	1.7575	2.81	3.48	1.16	5.94	2.6785	1.3501-3.5395
1990-2009	Gastrointestinal & metabolism	Target median	23	2	3.3026	2.67	3.04	5.0975	2.25	5.72	1.9709	2.4971-4.1081
1990-2009	Gastrointestinal & metabolism	[Drug - target median]	23	0	-0.85783	-1.695	-0.35	0.2375	-4.27	2.02	1.4964	-1.4694--0.24625
1990-2009	Genito urinary	Drug	18	1	3.4144	2.21	3.71	5.34	1.35	6.33	2.221	2.3884-4.4405
1990-2009	Genito urinary	Target median	18	0	4.1106	3.67	4.14	4.77	3.37	4.95	0.55857	3.8525-4.3686
1990-2009	Genito urinary	[Drug - target median]	18	1	-0.69611	-1.3	-0.66	0.46	-2.32	2.07	1.9497	-1.5968-0.2046
1990-2009	Infection	Drug	19	0	2.4232	0.835	2.72	4.175	-3.58	5.95	2.3591	1.3624-3.484
1990-2009	Infection	Target median	19	3	3.5563	3.575	3.575	4.3	2.49	4.3	1.2839	2.979-4.1336
1990-2009	Infection	[Drug - target median]	19	0	-1.1332	-1.935	-0.925	0.355	-4	1.61	1.6852	-1.8909--0.37539
1990-2009	Multiple/other	Drug	10	0	3.884	2.73	3.76	5.29	0.81	6.18	1.7179	2.8192-4.9488
1990-2009	Multiple/other	Target median	10	2	4.0285	3.65	3.9875	4.12	3.625	4.67	0.75241	3.5622-4.4948
1990-2009	Multiple/other	[Drug - target median]	10	0	-0.1445	-1.03	-0.39	0.595	-1.92	2.135	1.2569	-0.92355-0.63455
1990-2009	Musculo-skeletal	Drug	18	0	1.9217	-0.12	2.76	3.72	-1.66	4.18	2.049	0.97508-2.8683
1990-2009	Musculo-skeletal	Target median	18	0	2.9467	1.29	3.78	3.93	1.29	4.76	1.2889	2.3512-3.5421
1990-2009	Musculo-skeletal	[Drug - target median]	18	1	-1.025	-1.66	-0.97	-0.55	-2.95	0.25	1.1202	-1.5425--0.50751
1990-2009	Respiratory	Drug	20	1	4.0475	2.91	4.065	4.78	1.31	5.7	1.6734	3.3141-4.7809
1990-2009	Respiratory	Target median	20	3	4.7525	3.82	4.06	5.06	3.82	5.06	1.3053	4.1804-5.3246
1990-2009	Respiratory	[Drug - target median]	20	0	-0.705	-1.875	-0.65	0.225	-2.91	1.81	1.4161	-1.3256--0.084365
1990-2009	Skin & eye	Drug	11	0	3.3509	1.975	2.89	5.1275	0.09	6.68	2.1004	2.1097-4.5922
1990-2009	Skin & eye	Target median	11	0	3.8027	2.1475	3.93	5.2925	1.66	6.04	1.6528	2.826-4.7795
1990-2009	Skin & eye	[Drug - target median]	11	0	-0.45182	-1.0475	-0.45	-0.0275	-1.75	1.35	0.91562	-0.99291-0.089277
2010-2020	Cancer	Drug	61	1	4.1462	3.2275	4.22	5.1075	0.66	6.36	1.41	3.7924-4.5001
2010-2020	Cancer	Target median	61	2	3.9971	3.62	4.23	4.45	2.47	4.69	0.84102	3.7861-4.2082
2010-2020	Cancer	[Drug - target median]	61	0	0.1491	-0.49875	0.12	0.975	-2.32	1.91	1.0323	-0.10996-0.40815
2010-2020	Cardiovascular	Drug	12	0	3.3983	2.61	3.28	4.02	1.29	5.63	1.3001	2.6627-4.134
2010-2020	Cardiovascular	Target median	12	0	3.9008	3.28	3.66	4.62	2.73	5.55	0.85925	3.4147-4.387
2010-2020	Cardiovascular	[Drug - target median]	12	0	-0.5025	-1.09	-0.52	0.22	-2.37	0.74	0.89129	-1.0068-0.0017962
2010-2020	Central nervous system	Drug	13	1	3.9323	3.585	4.11	4.4925	2.37	5.85	1.0052	3.3859-4.4787
2010-2020	Central nervous system	Target median	13	2	3.8358	3.545	3.75	4.03	3.34	4.22	0.60281	3.5081-4.1635
2010-2020	Central nervous system	[Drug - target median]	13	1	0.096538	-0.2425	0.25	0.445	-0.66	1.12	0.66393	-0.26438-0.45745
2010-2020	Gastrointestinal & metabolism	Drug	22	0	2.3855	1.14	1.725	3.43	0.2	6.79	1.8372	1.6177-3.1532
2010-2020	Gastrointestinal & metabolism	Target median	22	0	2.9598	2.08	2.25	3.68	2.08	5.755	1.275	2.427-3.4926
2010-2020	Gastrointestinal & metabolism	[Drug - target median]	22	0	-0.57432	-1.11	-0.7	0.04	-2.05	1.1	0.90115	-0.95089--0.19775
2010-2020	Genito urinary	Drug	4	0	3.4775	2.31	3.26	4.645	1.85	5.54	1.5788	1.9303-5.0247
2010-2020	Genito urinary	Target median	4	0	4.455	3.48	4.51	5.43	3.37	5.43	1.1294	3.3482-5.5618
2010-2020	Genito urinary	[Drug - target median]	4	0	-0.9775	-1.6	-1.17	-0.355	-1.68	0.11	0.81553	-1.7767--0.17828
2010-2020	Infection	Drug	8	0	2.8775	0.785	3.575	4.765	-2.92	7.69	3.2941	0.59478-5.1602
2010-2020	Infection	Target median	8	1	2.9262	1.975	2.575	3.63	0.42	3.63	1.8287	1.659-4.1935
2010-2020	Infection	[Drug - target median]	8	1	-0.04875	-0.575	0.61	1.245	-0.59	1.62	2.0131	-1.4438-1.3463
2010-2020	Multiple/other	Drug	11	1	2.9709	1.865	2.91	3.4775	1.54	3.63	1.4639	2.1058-3.836
2010-2020	Multiple/other	Target median	11	3	3.9091	3.74	3.97	4.13	3.73	4.41	0.518	3.603-4.2152
2010-2020	Multiple/other	[Drug - target median]	11	1	-0.93818	-1.9175	-1.34	-0.3425	-2.47	-0.1	1.4224	-1.7788--0.097606
2010-2020	Musculo-skeletal	Drug	3	0	3.31	2.4275	3.68	4.1	2.01	4.24	1.1601	1.9972-4.6228
2010-2020	Musculo-skeletal	Target median	3	0	3.6967	3.315	3.93	4.02	3.11	4.05	0.5116	3.1177-4.2756
2010-2020	Musculo-skeletal	[Drug - target median]	3	0	-0.38667	-0.8875	-0.25	0.08	-1.1	0.19	0.65577	-1.1287-0.35541
2010-2020	Respiratory	Drug	7	0	4.46	3.7175	4.75	5.0675	3.15	5.14	0.83062	3.8447-5.0753
2010-2020	Respiratory	Target median	7	2	4.2907	4.26	4.34	4.34	4.26	4.34	0.22848	4.1215-4.46
2010-2020	Respiratory	[Drug - target median]	7	0	0.16929	-0.43375	0.41	0.6575	-0.94	0.88	0.70726	-0.35466-0.69323
