# Supplementary material for: Target-based evaluation of ‘drug-like’ properties and ligand efficiencies
Source: J Med Chem. Author manuscript; Available in PMC 2021 Jun 11. (PMC7610969; doi:10.1021/acs.jmedchem.1c00416)
Supplement: Fig 9 doses [file EMS123358-supplement-Fig_9_doses.pdf]

CMPD_PREF_NAME	CANONICAL_SMILES	TARGET_PREF_NAME	TARGET (ChEMBL code_name_species)	Approval Year	Approval period	Max daily oral dose mg	-p[Dose]	pCHEMBL
THEOPHYLLINE	Cn1c(=O)c2nc[nH]c2n(C)c1=O	Adenosine A2a receptor	252_Adenosine A2a receptor_Homo sapiens	1940	1939-1989	600	-2.47753	5.28
MEPERIDINE	CCOC(=O)C1(c2ccccc2)CCN(C)CC1	Mu opioid receptor	129_Mu opioid receptor_Homo sapiens	1942	1939-1989	900	-2.43905	6.27
HYDROCODONE	COc1ccc2c3c1O[C@H]1C(=O)CC[C@H]4[C@@H](C2)N(C)CC[C@]314	Mu opioid receptor	129_Mu opioid receptor_Homo sapiens	1943	1939-1989	60	-3.69806	8.02
METHYLERGONOVINE	CC[C@@H](CO)NC(=O)[C@@H]1C=C2c3cccc4[nH]cc(c34)C[C@H]2N(C)C1	Serotonin 2b (5-HT2b) receptor	227_Serotonin 2b (5-HT2b) receptor_Homo sapiens	1946	1939-1989	0.8	-5.62767	9.02
DIPHENHYDRAMINE	CN(C)CCOC(c1ccccc1)c1ccccc1	Histamine H1 receptor	127_Histamine H1 receptor_Homo sapiens	1946	1939-1989	300	-2.93003	7.23
METHADONE	CCC(=O)C(CC(C)N(C)C)(c1ccccc1)c1ccccc1	Mu opioid receptor	129_Mu opioid receptor_Homo sapiens	1947	1939-1989	120	-3.41141	8.39
PROPYLTHIOURACIL	CCCc1cc(=O)[nH]c(=S)[nH]1	Thyroid peroxidase	40_Thyroid peroxidase_Homo sapiens	1947	1939-1989	1200	-2.15188	5.47
CAFFEINE	Cn1c(=O)c2c(ncn2C)n(C)c1=O	Adenosine A3 receptor	280_Adenosine A3 receptor_Homo sapiens	1948	1939-1989	600	-2.51008	4.88
ERGOTAMINE	CN1C[C@H](C(=O)N[C@]2(C)O[C@@]3(O)[C@@H]4CCCN4C(=O)[C@H](Cc4ccccc4)N3C2=O)C=C2c3cccc4[nH]cc(c34)C[C@H]21	Alpha-2a adrenergic receptor	52_Alpha-2a adrenergic receptor_Homo sapiens	1948	1939-1989	6	-4.98653	8.89
LIDOCAINE	CCN(CC)CC(=O)Nc1c(C)cccc1C	Sodium channel protein type IV alpha subunit	18043_Sodium channel protein type IV alpha subunit_Homo sapiens	1948	1939-1989	300	-2.89273	5.7
TRIPELENNAMINE	CN(C)CCN(Cc1ccccc1)c1ccccn1	Histamine H1 receptor	127_Histamine H1 receptor_Homo sapiens	1948	1939-1989	600	-2.62902	7.4
TRIHEXYPHENIDYL	OC(CCN1CCCCC1)(c1ccccc1)C1CCCCC1	Muscarinic acetylcholine receptor M1	61_Muscarinic acetylcholine receptor M1_Homo sapiens	1949	1939-1989	15	-4.30315	8.87
ASPIRIN	CC(=O)Oc1ccccc1C(=O)O	Cyclooxygenase	104725_Cyclooxygenase_Homo sapiens	1950	1939-1989	3900	-1.66459	4.82
QUINIDINE	C=C[C@H]1CN2CC[C@H]1C[C@@H]2[C@@H](O)c1ccnc2ccc(OC)cc12	Sodium channel protein type V alpha subunit	11480_Sodium channel protein type V alpha subunit_Homo sapiens	1950	1939-1989	1200	-2.43193	5.16
OXYCODONE	COc1ccc2c3c1O[C@H]1C(=O)CC[C@@]4(O)[C@@H](C2)N(C)CC[C@]314	Mu opioid receptor	129_Mu opioid receptor_Homo sapiens	1950	1939-1989	75	-3.62376	6.6
DICYCLOMINE	CCN(CC)CCOC(=O)C1(C2CCCCC2)CCCCC1	Muscarinic acetylcholine receptor M1	61_Muscarinic acetylcholine receptor M1_Homo sapiens	1950	1939-1989	160	-3.28653	8.77
CORTISONE ACETATE	CC(=O)OCC(=O)[C@@]1(O)CC[C@H]2[C@@H]3CCC4=CC(=O)CC[C@]4(C)[C@H]3C(=O)C[C@@]21C	Glucocorticoid receptor	25_Glucocorticoid receptor_Homo sapiens	1950	1939-1989	300	-3.12763	5.58
CHLORPHENIRAMINE	CN(C)CCC(c1ccc(Cl)cc1)c1ccccn1	Histamine H1 receptor	127_Histamine H1 receptor_Homo sapiens	1950	1939-1989	24	-4.05881	8.25
PROMETHAZINE	CC(CN1c2ccccc2Sc2ccccc21)N(C)C	Histamine H1 receptor	127_Histamine H1 receptor_Homo sapiens	1951	1939-1989	100	-3.45398	8.54
CODEINE	COc1ccc2c3c1O[C@H]1[C@@H](O)C=C[C@H]4[C@@H](C2)N(C)CC[C@]314	Mu opioid receptor	129_Mu opioid receptor_Homo sapiens	1952	1939-1989	360	-2.91991	6.14
HYDROCORTISONE	C[C@]12CC[C@H]3[C@@H](CCC4=CC(=O)CC[C@@]43C)[C@@H]1CC[C@]2(O)C(=O)CO	Glucocorticoid receptor	25_Glucocorticoid receptor_Homo sapiens	1952	1939-1989	240	-3.17906	7.75
PHENYLEPHRINE	CNC[C@H](O)c1cccc(O)c1	Alpha-1b adrenergic receptor	128_Alpha-1b adrenergic receptor_Homo sapiens	1952	1939-1989	40	-3.6212	8.65
METHOTREXATE	CN(Cc1cnc2nc(N)nc(N)c2n1)c1ccc(C(=O)N[C@@H](CCC(=O)O)C(=O)O)cc1	Dihydrofolate reductase	6_Dihydrofolate reductase_Homo sapiens	1953	1939-1989	30	-4.18036	8.17
PHENYTOIN	O=C1NC(=O)C(c2ccccc2)(c2ccccc2)N1	Sodium channel protein type IV alpha subunit	18043_Sodium channel protein type IV alpha subunit_Homo sapiens	1953	1939-1989	300	-2.92474	4.62
METHSCOPOLAMINE	C[N+]1(C)[C@H]2C[C@@H](OC(=O)[C@H](CO)c3ccccc3)C[C@@H]1[C@H]1O[C@@H]21	Muscarinic acetylcholine receptor M1	61_Muscarinic acetylcholine receptor M1_Homo sapiens	1953	1939-1989	20	-4.20193	9.63
PYRIMETHAMINE	CCc1nc(N)nc(N)c1-c1ccc(Cl)cc1	Dihydrofolate reductase	70_Dihydrofolate reductase_Plasmodium falciparum K1	1953	1939-1989	75	-3.52065	7.54
ACETAZOLAMIDE	CC(=O)Nc1nnc(S(N)(=O)=O)s1	Carbonic anhydrase XII	12209_Carbonic anhydrase XII_Homo sapiens	1953	1939-1989	1000	-2.34684	7.41
BENZTROPINE	CN1[C@H]2CC[C@@H]1C[C@H](OC(c1ccccc1)c1ccccc1)C2	Muscarinic acetylcholine receptor M1	61_Muscarinic acetylcholine receptor M1_Homo sapiens	1954	1939-1989	6	-4.70961	9.57
DEXTROAMPHETAMINE	C[C@H](N)Cc1ccccc1	Norepinephrine transporter	100_Norepinephrine transporter_Homo sapiens	1955	1939-1989	60	-3.35286	7
METHYLPHENIDATE	COC(=O)C(c1ccccc1)C1CCCCN1	Dopamine transporter	155_Dopamine transporter_Homo sapiens	1955	1939-1989	60	-3.58978	7.1
PYRIDOSTIGMINE	CN(C)C(=O)Oc1ccc[n+](C)c1	Acetylcholinesterase	93_Acetylcholinesterase_Homo sapiens	1955	1939-1989	1080	-2.22478	6.44
HYDROXYCHLOROQUINE	CCN(CCO)CCCC(C)Nc1ccnc2cc(Cl)ccc12	Toll-like receptor 9	101567_Toll-like receptor 9_Homo sapiens	1955	1939-1989	1200	-2.447	6.96
PREDNISOLONE	C[C@]12C[C@H](O)[C@H]3[C@@H](CCC4=CC(=O)C=C[C@@]43C)[C@@H]1CC[C@]2(O)C(=O)CO	Glucocorticoid receptor	25_Glucocorticoid receptor_Homo sapiens	1955	1939-1989	60	-3.77869	7.87
MECAMYLAMINE	CNC1(C)C2CCC(C2)C1(C)C	Neuronal acetylcholine receptor; alpha3/beta4	104288_Neuronal acetylcholine receptor; alpha3/beta4_Homo sapiens	1956	1939-1989	5	-4.52453	5.85
HYDROXYZINE	OCCOCCN1CCN(C(c2ccccc2)c2ccc(Cl)cc2)CC1	Histamine H1 receptor	127_Histamine H1 receptor_Homo sapiens	1956	1939-1989	400	-2.97187	8.7
PROCHLORPERAZINE	CN1CCN(CCCN2c3ccccc3Sc3ccc(Cl)cc32)CC1	Dopamine D2 receptor	72_Dopamine D2 receptor_Homo sapiens	1956	1939-1989	150	-3.39672	8.2
PROMAZINE	CN(C)CCCN1c2ccccc2Sc2ccccc21	Serotonin 2a (5-HT2a) receptor	107_Serotonin 2a (5-HT2a) receptor_Homo sapiens	1956	1939-1989	800	-2.55089	7.9
MEPENZOLATE	C[N+]1(C)CCCC(OC(=O)C(O)(c2ccccc2)c2ccccc2)C1	Muscarinic acetylcholine receptor M3	219_Muscarinic acetylcholine receptor M3_Homo sapiens	1956	1939-1989	200	-3.23101	8.59
FLUOXYMESTERONE	C[C@]1(O)CC[C@H]2[C@@H]3CCC4=CC(=O)CC[C@]4(C)[C@@]3(F)[C@@H](O)C[C@@]21C	Androgen Receptor	56_Androgen Receptor_Homo sapiens	1956	1939-1989	40	-3.92486	8.24
LIOTHYRONINE	N[C@@H](Cc1cc(I)c(Oc2ccc(O)c(I)c2)c(I)c1)C(=O)O	Thyroid hormone receptor beta-1	271_Thyroid hormone receptor beta-1_Homo sapiens	1956	1939-1989	0.075	-6.93851	9.22
CHLORPROMAZINE	CN(C)CCCN1c2ccccc2Sc2ccc(Cl)cc21	Dopamine D3 receptor	130_Dopamine D3 receptor_Homo sapiens	1957	1939-1989	800	-2.60052	8.38
PERPHENAZINE	OCCN1CCN(CCCN2c3ccccc3Sc3ccc(Cl)cc32)CC1	Dopamine D2 receptor	72_Dopamine D2 receptor_Homo sapiens	1957	1939-1989	64	-3.80018	9.52
METHYLPREDNISOLONE	C[C@H]1C[C@H]2[C@@H]3CC[C@](O)(C(=O)CO)[C@@]3(C)C[C@H](O)[C@@H]2[C@@]2(C)C=CC(=O)C=C12	Glucocorticoid receptor	25_Glucocorticoid receptor_Homo sapiens	1957	1939-1989	48	-3.89219	8.13
ORPHENADRINE	Cc1ccccc1C(OCCN(C)C)c1ccccc1	Histamine H1 receptor	127_Histamine H1 receptor_Homo sapiens	1957	1939-1989	200	-3.12935	6.38
TRIAMCINOLONE	C[C@]12C[C@H](O)[C@@]3(F)[C@@H](CCC4=CC(=O)C=C[C@@]43C)[C@@H]1C[C@H](O)[C@]2(O)C(=O)CO	Glucocorticoid receptor	25_Glucocorticoid receptor_Homo sapiens	1957	1939-1989	60	-3.81783	7.41
DEXAMETHASONE	C[C@@H]1C[C@H]2[C@@H]3CCC4=CC(=O)C=C[C@]4(C)[C@@]3(F)[C@@H](O)C[C@]2(C)[C@@]1(O)C(=O)CO	Glucocorticoid receptor	25_Glucocorticoid receptor_Homo sapiens	1958	1939-1989	9	-4.63956	8.46
DICHLORPHENAMIDE	NS(=O)(=O)c1cc(Cl)c(Cl)c(S(N)(=O)=O)c1	Carbonic anhydrase II	15_Carbonic anhydrase II_Homo sapiens	1958	1939-1989	200	-3.1835	7.42
BIPERIDEN	OC(CCN1CCCCC1)(c1ccccc1)C1CC2C=CC1C2	Muscarinic acetylcholine receptor M1	61_Muscarinic acetylcholine receptor M1_Homo sapiens	1959	1939-1989	16	-4.2893	8.63
FLUPHENAZINE	OCCN1CCN(CCCN2c3ccccc3Sc3ccc(C(F)(F)F)cc32)CC1	Dopamine D2 receptor	72_Dopamine D2 receptor_Homo sapiens	1959	1939-1989	20	-4.33998	8.84
IMIPRAMINE	CN(C)CCCN1c2ccccc2CCc2ccccc21	Serotonin transporter	121_Serotonin transporter_Homo sapiens	1959	1939-1989	300	-2.97069	8.36
OXYMORPHONE	CN1CC[C@]23c4c5ccc(O)c4O[C@H]2C(=O)CC[C@@]3(O)[C@H]1C5	Mu opioid receptor	129_Mu opioid receptor_Homo sapiens	1959	1939-1989	40	-3.877	8.43
MEDROXYPROGESTERONE ACETATE	CC(=O)O[C@]1(C(C)=O)CC[C@H]2[C@@H]3C[C@H](C)C4=CC(=O)CC[C@]4(C)[C@H]3CC[C@@]21C	Progesterone receptor	36_Progesterone receptor_Homo sapiens	1959	1939-1989	10	-4.58718	9.16
SULFINPYRAZONE	O=C1C(CC[S+]([O-])c2ccccc2)C(=O)N(c2ccccc2)N1c1ccccc1	Solute carrier family 22 member 12	102767_Solute carrier family 22 member 12_Homo sapiens	1959	1939-1989	400	-3.00485	4
METHAZOLAMIDE	CC(=O)/N=c1/sc(S(N)(=O)=O)nn1C	Carbonic anhydrase VII	11060_Carbonic anhydrase VII_Homo sapiens	1959	1939-1989	300	-2.89631	8.68
RESERPINE	COC(=O)[C@H]1[C@H]2C[C@@H]3c4[nH]c5cc(OC)ccc5c4CCN3C[C@H]2C[C@@H](OC(=O)c2cc(OC)c(OC)c(OC)c2)[C@@H]1OC	Synaptic vesicular amine transporter	162_Synaptic vesicular amine transporter_Homo sapiens	1960	1939-1989	0.5	-6.08543	7.88
SPIRONOLACTONE	CC(=O)S[C@@H]1CC2=CC(=O)CC[C@]2(C)[C@H]2CC[C@@]3(C)[C@@H](CC[C@]34CCC(=O)O4)[C@@H]21	Mineralocorticoid receptor	48_Mineralocorticoid receptor_Homo sapiens	1960	1939-1989	400	-3.01764	7.96
ATROPINE	CN1[C@H]2CC[C@@H]1CC(OC(=O)C(CO)c1ccccc1)C2	Muscarinic acetylcholine receptor M1	61_Muscarinic acetylcholine receptor M1_Homo sapiens	1960	1939-1989	3	-4.98433	9.24
DIPYRIDAMOLE	OCCN(CCO)c1nc(N2CCCCC2)c2nc(N(CCO)CCO)nc(N3CCCCC3)c2n1	Equilibrative nucleoside transporter 1	175_Equilibrative nucleoside transporter 1_Homo sapiens	1961	1939-1989	400	-3.10092	8.06
AMITRIPTYLINE	CN(C)CCC=C1c2ccccc2CCc2ccccc21	Serotonin transporter	121_Serotonin transporter_Homo sapiens	1961	1939-1989	150	-3.26703	8.2
PHENELZINE	NNCCc1ccccc1	Monoamine oxidase B	104_Monoamine oxidase B_Homo sapiens	1961	1939-1989	90	-3.17993	6.91
BETAMETHASONE	C[C@H]1C[C@H]2[C@@H]3CCC4=CC(=O)C=C[C@]4(C)[C@@]3(F)[C@@H](O)C[C@]2(C)[C@@]1(O)C(=O)CO	Glucocorticoid receptor	25_Glucocorticoid receptor_Homo sapiens	1961	1939-1989	30	-4.11669	8.28
COLCHICINE	COc1cc2c(c(OC)c1OC)-c1ccc(OC)c(=O)cc1[C@@H](NC(C)=O)CC2	Tubulin	104766_Tubulin_Homo sapiens	1961	1939-1989	2.4	-5.22124	5.66
CYPROHEPTADINE	CN1CCC(=C2c3ccccc3C=Cc3ccccc32)CC1	Histamine H1 receptor	127_Histamine H1 receptor_Homo sapiens	1961	1939-1989	20	-4.15747	8.43
[illegible]	[illegible]	[illegible]	[illegible]	[illegible]	[illegible]	[illegible]	[illegible]	[illegible]

OZANIMOD	CC(C)Oc1ccc(-c2nc(-c3cccc4c3CC[C@@H]4NCCO)no2)cc1C#N	Sphingosine 1-phosphate receptor Edg-1	11631_Sphingosine 1-phosphate receptor Edg-1_Homo sapiens	2020	2010-2020	0.92	-5.6431	9.8
SELUMETINIB	Cn1cnc2c(F)c(Nc3ccc(Br)cc3Cl)c(C(=O)NOCCO)cc21	Dual specificity mitogen-activated protein kinase kinase 1	11409-WT_Dual specificity mitogen-activated protein kinase kinase 1_Homo sapiens	2020	2010-2020	100	-3.66057	7.31
FADROZOLE	N#Cc1ccc(C2CCCc3cncn32)cc1	Cytochrome P450 19A1	65_Cytochrome P450 19A1_Homo sapiens	1995	1990-2009	4	-4.74679	7.53
MOXONIDINE	COc1nc(C)nc(Cl)c1NC1=NCCN1	Nischarin	11684_Nischarin_Homo sapiens	1991	1990-2009	0.6	-5.60509	7.82
CIPROFIBRATE	CC(C)(Oc1ccc(C2CC2(Cl)Cl)cc1)C(=O)O	Peroxisome proliferator-activated receptor alpha	163_Peroxisome proliferator-activated receptor alpha_Homo sapiens	1985	1939-1989	100	-3.46114	6.05
BEZAFIBRATE	CC(C)(Oc1ccc(CCNC(=O)c2ccc(Cl)cc2)cc1)C(=O)O	Peroxisome proliferator-activated receptor	109738_Peroxisome proliferator-activated receptor_Homo sapiens		1939-1989	600	-2.78035	5.98
MOCLOBEMIDE	O=C(NCCN1CCOCC1)c1ccc(Cl)cc1	Monoamine oxidase A	86_Monoamine oxidase A_Homo sapiens	1990	1990-2009	600	-2.65118	6.98
MOFEZOLAC	COc1ccc(-c2noc(CC(=O)O)c2-c2ccc(OC)cc2)cc1	Cyclooxygenase-1	96_Cyclooxygenase-1_Homo sapiens	1994	1990-2009	225	-3.17847	8.48
SERTINDOLE	O=C1NCCN1CCN1CCC(c2cn(-c3ccc(F)cc3)c3ccc(Cl)cc23)CC1	Serotonin 2a (5-HT2a) receptor	107_Serotonin 2a (5-HT2a) receptor_Homo sapiens	1996	1990-2009	24	-4.26418	9.15
REBOXETINE	CCOc1ccccc1OC(c1ccccc1)C1CNCCO1	Norepinephrine transporter	100_Norepinephrine transporter_Homo sapiens	1997	1990-2009	12	-4.41692	8.49
AGOMELATINE	COc1ccc2cccc(CCNC(C)=O)c2c1	Melatonin receptor	104733_Melatonin receptor_Homo sapiens	2009	1990-2009	50	-3.68719	10.06
TOLOXATONE	Cc1cccc(N2CC(CO)OC2=O)c1	Monoamine oxidase A	86_Monoamine oxidase A_Homo sapiens	1984	1939-1989	1000	-2.31645	5.79
AMISULPRIDE	CCN1CCCC1CNC(=O)c1cc(S(=O)(=O)CC)c(N)cc1OC	Dopamine D3 receptor	130_Dopamine D3 receptor_Homo sapiens	1986	1939-1989	1200	-2.48842	8.4
BENPERIDOL	O=C(CCCN1CCC(n2c(O)nc3ccccc32)CC1)c1ccc(F)cc1	Dopamine D2 receptor	72_Dopamine D2 receptor_Homo sapiens		1939-1989	1.5	-5.40535	10.57
CINNARIZINE	C(=C/c1ccccc1)\CN1CCN(C(c2ccccc2)c2ccccc2)CC1	Histamine H1 receptor	127_Histamine H1 receptor_Homo sapiens		1939-1989	90	-3.61222	7.6
FLUNITRAZEPAM	CN1C(=O)CN=C(c2ccccc2F)c2cc([N+](=O)[O-])ccc21	GABA receptor alpha-5 subunit	11440_GABA receptor alpha-5 subunit_Homo sapiens		1939-1989	2	-5.19492	8.52
IPRONIAZID	CC(C)NNC(=O)c1ccncc1	Monoamine oxidase A	86_Monoamine oxidase A_Homo sapiens		1939-1989	250	-2.85545	5.18
MELATONIN	COc1ccc2[nH]cc(CCNC(C)=O)c2c1	Melatonin receptor 1A	11084_Melatonin receptor 1A_Homo sapiens		1939-1989	2	-5.06498	9.64
SULPIRIDE	CCN1CCCC1CNC(=O)c1cc(S(N)(=O)=O)ccc1OC	Dopamine D2 receptor	72_Dopamine D2 receptor_Homo sapiens		1939-1989	2400	-2.15309	7.17
GOSOGLIPTIN	O=C([C@@H]1C[C@H](N2CCN(c3ncccn3)CC2)CN1)N1CCC(F)(F)C1	Dipeptidyl peptidase IV	11140_Dipeptidyl peptidase IV_Homo sapiens	2016	2010-2020	20	-4.26295	7.92
VOGLIBOSE	OCC(CO)N[C@H]1C[C@](O)(CO)[C@@H](O)[C@H](O)[C@H]1O	Lysosomal alpha-glucosidase	10977_Lysosomal alpha-glucosidase_Homo sapiens	1994	1990-2009	0.9	-5.47272	6.21
TOLRESTAT	COc1ccc2c(C(=S)N(C)CC(=O)O)cccc2c1C(F)(F)F	Aldose reductase	242_Aldose reductase_Homo sapiens	1989	1939-1989	400	-2.95103	7.46
CARBENOXOLONE	CC1(C)[C@@H](OC(=O)CCC(=O)O)CC[C@@]2(C)[C@H]1CC[C@]1(C)[C@@H]2C(=O)C=C2[C@@H]3C[C@@](C)(C(=O)O)CC[C@]3(C)CC[C@]21C	11-beta-hydroxysteroid dehydrogenase 1	11489_11-beta-hydroxysteroid dehydrogenase 1_Homo sapiens		1939-1989	300	-3.27934	6.48
CLEBOPRIDE	COc1cc(N)c(Cl)cc1C(=O)NC1CCN(Cc2ccccc2)CC1	Dopamine D2 receptor	72_Dopamine D2 receptor_Homo sapiens		1939-1989	1.5	-5.39664	7.84
DOMPERIDONE	O=c1[nH]c2ccccc2n1CCCN1CCC(n2c(=O)[nH]c3cc(Cl)ccc32)CC1	Dopamine D2 receptor	72_Dopamine D2 receptor_Homo sapiens		1939-1989	30	-4.15221	8.89
PIRENZEPINE	CN1CCN(CC(=O)N2c3ccccc3C(=O)Nc3cccnc32)CC1	Muscarinic acetylcholine receptor M1	61_Muscarinic acetylcholine receptor M1_Homo sapiens		1939-1989	150	-3.36972	7.7
LASOFOXIFENE	Oc1ccc2c(c1)CC[C@H](c1ccccc1)[C@@H]2c1ccc(OCCN2CCCC2)cc1	Estrogen receptor alpha	19_Estrogen receptor alpha_Homo sapiens	2009	1990-2009	0.5	-5.91757	8.51
CYPROTERONE ACETATE	CC(=O)O[C@]1(C(C)=O)CC[C@H]2[C@@H]3C=C(Cl)C4=CC(=O)[C@@H]5C[C@@H]5[C@]4(C)[C@H]3CC[C@@]21C	Androgen Receptor	56_Androgen Receptor_Homo sapiens		1939-1989	300	-3.14296	7.85
CYCLOGUANIL	CC1(C)N=C(N)N=C(N)N1c1ccc(Cl)cc1	Dihydrofolate reductase	70_Dihydrofolate reductase_Plasmodium falciparum K1		1939-1989	400	-2.79886	6.82
MELAGATRAN	N=C(N)c1ccc(CNC(=O)[C@@H]2CCN2C(=O)[C@H](NCC(=O)O)C2CCCCC2)cc1	Thrombin	11_Thrombin_Homo sapiens	2004	1990-2009	72	-3.77565	8.37
OZAGREL	O=C(O)/C=C/c1ccc(Cn2ccnc2)cc1	Thromboxane-A synthase	248_Thromboxane-A synthase_Homo sapiens	1988	1939-1989	400	-2.75635	6.52
DIMETHINDENE	CC(C1=C(CCN(C)C)Cc2ccccc21)c1ccccn1	Histamine H1 receptor	127_Histamine H1 receptor_Homo sapiens		1939-1989	6	-4.68787	9.16
MOXISYLYTE	CC(=O)Oc1cc(C(C)C)c(OCCN(C)C)cc1C	Adrenergic receptor alpha	104787_Adrenergic receptor alpha_Homo sapiens		1939-1989	320	-2.94105	7.5
DEXIBUPROFEN	CC(C)Cc1ccc([C@H](C)C(=O)O)cc1	Cyclooxygenase-1	96_Cyclooxygenase-1_Homo sapiens	1994	1990-2009	1200	-2.23528	6.98
DEXKETOPROFEN	C[C@H](C(=O)O)c1cccc(C(=O)c2ccccc2)c1	Cyclooxygenase-1	96_Cyclooxygenase-1_Homo sapiens	1994	1990-2009	75	-3.53027	8.54
BENZBROMARONE	CCc1oc2ccccc2c1C(=O)c1cc(Br)c(O)c(Br)c1	Solute carrier family 22 member 12	102767_Solute carrier family 22 member 12_Homo sapiens		1939-1989	300	-3.15034	7.19
OXYPURINOL	O=c1[nH]c(=O)c2c[nH]nc2[nH]1	Xanthine dehydrogenase	149_Xanthine dehydrogenase_Homo sapiens		1939-1989	800	-2.27907	6.73
SERATRODAST	CC1=C(C)C(=O)C(C(CCCCCC(=O)O)c2ccccc2)=C(C)C1=O	Thromboxane A2 receptor	246_Thromboxane A2 receptor_Homo sapiens	1995	1990-2009	320	-3.04441	8.13
MIZOLASTINE	CN(c1nccc(=O)[nH]1)C1CCN(c2nc3ccccc3n2Cc2ccc(F)cc2)CC1	Histamine H1 receptor	127_Histamine H1 receptor_Homo sapiens	1998	1990-2009	10	-4.63599	8.57
IBUDILAST	CC(C)C(=O)c1c(C(C)C)nn2ccccc12	Phosphodiesterase 4D	11359_Phosphodiesterase 4D_Homo sapiens	1989	1939-1989	60	-3.58416	6.78
CARBETAPENTANE	CCN(CC)CCOCCOC(=O)C1(c2ccccc2)CCCC1	Sigma opioid receptor	11272_Sigma opioid receptor_Homo sapiens		1939-1989	120	-3.44388	7.71
CLENBUTEROL	CC(C)(C)NCC(O)c1cc(Cl)c(N)c(Cl)c1	Beta-2 adrenergic receptor	43_Beta-2 adrenergic receptor_Homo sapiens		1939-1989	0.06	-6.66464	7.39
FENOTEROL	CC(Cc1ccc(O)cc1)NCC(O)c1cc(O)cc(O)c1	Beta-2 adrenergic receptor	43_Beta-2 adrenergic receptor_Homo sapiens		1939-1989	10	-4.48196	6.9
