# Supplementary material for: Target-based evaluation of ‘drug-like’ properties and ligand efficiencies
Source: J Med Chem. Author manuscript; Available in PMC 2021 Jun 11. (PMC7610969; doi:10.1021/acs.jmedchem.1c00416)

DRUG	CMPD_PREF_NAME	CANONICAL_SMILES	TARGET_PREF_NAME
	2',3'-DIDEOXYADENOSINE TRIPHOSPHATE	Nc1ncnc2c1ncn2[C@H]1CC[C@@H](COP(=O)(O)OP(=O)(O)OP(=O)(O)O)O1	Human immunodeficiency virus [illegible]
	7-ETHYL-10-HYDROXYCAMPTOTHECIN	CCc1c2c(nc3ccc(O)cc13)-c1cc3c(c(=O)n1C2)COC(=O)[C@]3(O)CC	DNA topoisomerase I
	ABEMACICLIB	CCN1CCN(Cc2ccc(Nc3ncc(F)c(-c4cc(F)c5nc(C)n(C(C)C)c5c4)n3)nc2)CC1	Cyclin-dependent kinase 6
	ABEMACICLIB	CCN1CCN(Cc2ccc(Nc3ncc(F)c(-c4cc(F)c5nc(C)n(C(C)C)c5c4)n3)nc2)CC1	Cyclin-dependent kinase 4
	ABIRATERONE	C[C@]12CC[C@H]3[C@@H](CC=C4C[C@@H](O)CC[C@@]43C)[C@@H]1CC=C2c1cccnc1	Cytochrome P450 17A1
	ACALABRUTINIB	CC#CC(=O)N1CCC[C@H]1c1nc(-c2ccc(C(=O)Nc3ccccn3)cc2)c2c(N)nccn12	Tyrosine-protein kinase BTK
	ACEBUTOLOL	CCCC(=O)Nc1ccc(OCC(O)CNC(C)C)c(C(C)=O)c1	Beta-1 adrenergic receptor
	ACETAZOLAMIDE	CC(=O)Nc1nnc(S(N)(=O)=O)s1	Carbonic anhydrase I
	ACETAZOLAMIDE	CC(=O)Nc1nnc(S(N)(=O)=O)s1	Carbonic anhydrase XII
	ACETAZOLAMIDE	CC(=O)Nc1nnc(S(N)(=O)=O)s1	Carbonic anhydrase IV
	ACETAZOLAMIDE	CC(=O)Nc1nnc(S(N)(=O)=O)s1	Carbonic anhydrase II
	ACETYLCHOLINE	CC(=O)OCC[N+](C)(C)C	Muscarinic acetylcholine recep[illegible]
	ACLIDINIUM	O=C(O[C@H]1C[N+]2(CCCOc3ccccc3)CCC1CC2)C(O)(c1cccs1)c1cccs1	Muscarinic acetylcholine recep[illegible]
	ADAPALENE	COc1ccc(-c2ccc3cc(C(=O)O)ccc3c2)cc1C12CC3CC(CC(C3)C1)C2	Retinoic acid receptor gamma
	ADAPALENE	COc1ccc(-c2ccc3cc(C(=O)O)ccc3c2)cc1C12CC3CC(CC(C3)C1)C2	Retinoic acid receptor beta
	ADAPALENE	COc1ccc(-c2ccc3cc(C(=O)O)ccc3c2)cc1C12CC3CC(CC(C3)C1)C2	Retinoic acid receptor alpha
	ADENOSINE	Nc1ncnc2c1ncn2[C@@H]1O[C@H](CO)[C@@H](O)[C@H]1O	Adenosine A1 receptor
	ADENOSINE	Nc1ncnc2c1ncn2[C@@H]1O[C@H](CO)[C@@H](O)[C@H]1O	Adenosine A2a receptor
	ADENOSINE	Nc1ncnc2c1ncn2[C@@H]1O[C@H](CO)[C@@H](O)[C@H]1O	Adenosine A2b receptor
	ADENOSINE	Nc1ncnc2c1ncn2[C@@H]1O[C@H](CO)[C@@H](O)[C@H]1O	Adenosine A3 receptor
	AFATINIB	CN(C)C/C=C/C(=O)Nc1cc2c(Nc3ccc(F)c(Cl)c3)ncnc2cc1O[C@H]1CCOC1	Receptor protein-tyrosine kina[illegible]
	AFATINIB	CN(C)C/C=C/C(=O)Nc1cc2c(Nc3ccc(F)c(Cl)c3)ncnc2cc1O[C@H]1CCOC1	Receptor protein-tyrosine kina[illegible]
	AFATINIB	CN(C)C/C=C/C(=O)Nc1cc2c(Nc3ccc(F)c(Cl)c3)ncnc2cc1O[C@H]1CCOC1	Epidermal growth factor recept[illegible]
	AFIMOXIFENE	CCC(=C(c1ccc(O)cc1)c1ccc(OCCN(C)C)cc1)c1ccccc1	Estrogen receptor alpha
	AGOMELATINE	COc1ccc2cccc(CCNC(C)=O)c2c1	Melatonin receptor
	AGOMELATINE	COc1ccc2cccc(CCNC(C)=O)c2c1	Serotonin 2c (5-HT2c) receptor
	AGOMELATINE	COc1ccc2cccc(CCNC(C)=O)c2c1	Melatonin receptor 1A
	AGOMELATINE	COc1ccc2cccc(CCNC(C)=O)c2c1	Melatonin receptor 1B
	ALBUTEROL	CC(C)(C)NCC(O)c1ccc(O)c(CO)c1	Beta-2 adrenergic receptor
	ALECTINIB	CCc1cc2c(cc1N1CCC(N3CCOCC3)CC1)C(C)(C)c1[nH]c3cc(C#N)ccc3c1C2=O	Tyrosine-protein kinase recepto[illegible]
	ALECTINIB	CCc1cc2c(cc1N1CCC(N3CCOCC3)CC1)C(C)(C)c1[nH]c3cc(C#N)ccc3c1C2=O	ALK tyrosine kinase receptor
	ALENDRONIC ACID	NCCCC(O)(P(=O)(O)O)P(=O)(O)O	Farnesyl diphosphate synthase
	ALFUZOSIN	COc1cc2nc(N(C)CCCNC(=O)C3CCCO3)nc(N)c2cc1OC	Alpha-1d adrenergic receptor
	ALFUZOSIN	COc1cc2nc(N(C)CCCNC(=O)C3CCCO3)nc(N)c2cc1OC	Alpha-1a adrenergic receptor
	ALFUZOSIN	COc1cc2nc(N(C)CCCNC(=O)C3CCCO3)nc(N)c2cc1OC	Alpha-1b adrenergic receptor
	ALISKIREN	COCCCOc1cc(C[C@@H](C[C@H](N)[C@@H](O)C[C@H](C(=O)NCC(C)(C)C(N)=O)C(C)C)C(C)C)ccc1OC	Renin
	ALITRETINOIN	CC1=C(/C=C/C(C)=C\C=C\C(C)=C\C(=O)O)C(C)(C)CCC1	Retinoic acid receptor gamma
	ALITRETINOIN	CC1=C(/C=C/C(C)=C\C=C\C(C)=C\C(=O)O)C(C)(C)CCC1	Retinoid X receptor gamma
	ALITRETINOIN	CC1=C(/C=C/C(C)=C\C=C\C(C)=C\C(=O)O)C(C)(C)CCC1	Retinoic acid receptor beta
	ALITRETINOIN	CC1=C(/C=C/C(C)=C\C=C\C(C)=C\C(=O)O)C(C)(C)CCC1	Retinoid X receptor alpha
	ALITRETINOIN	CC1=C(/C=C/C(C)=C\C=C\C(C)=C\C(=O)O)C(C)(C)CCC1	Retinoid X receptor beta
	ALITRETINOIN	CC1=C(/C=C/C(C)=C\C=C\C(C)=C\C(=O)O)C(C)(C)CCC1	Retinoic acid receptor alpha
	ALOGLIPTIN	Cn1c(=O)cc(N2CCC[C@@H](N)C2)n(Cc2ccccc2C#N)c1=O	Dipeptidyl peptidase IV
	ALOSETRON	Cc1[nH]cnc1CN1CCc2c(c3ccccc3n2C)C1=O	Serotonin 3a (5-HT3a) receptor
	ALPELISIB	Cc1nc(NC(=O)N2CCC[C@H]2C(N)=O)sc1-c1ccnc(C(C)(C)C(F)(F)F)c1	[illegible]
	ALPRAZOLAM	Cc1nnc2n1-c1ccc(Cl)cc1C(c1ccccc1)=NC2	[illegible]
	ALVIMOPAN	C[C@H](CN(C[C@H](Cc2ccccc2)C(=O)NCC(=O)O)CC[C@@]1(C)c1cccc(O)c1	[illegible]
	AMANTADINE	NC12CC3CC(CC(C3)C1)C2	[illegible]
	AMINOCAPROIC ACID	NCCCCCC(=O)O	[illegible]
	AMINOGLUTETHIMIDE	CCC1(c2ccc(N)cc2)CCC(=O)NC1=O	[illegible]
	AMIODARONE	CCCCc1oc2ccccc2c1C(=O)c1cc(I)c(OCCN(CC)CC)c(I)c1	[illegible]
	AMISULPRIDE	CCN1CCCC1CNC(=O)c1cc(S(=O)(=O)CC)c(N)cc1OC	[illegible]
	AMISULPRIDE	CCN1CCCC1CNC(=O)c1cc(S(=O)(=O)CC)c(N)cc1OC	[illegible]
	AMISULPRIDE	CCN1CCCC1CNC(=O)c1cc(S(=O)(=O)CC)c(N)cc1OC	[illegible]
	AMISULPRIDE	CCN1CCCC1CNC(=O)c1cc(S(=O)(=O)CC)c(N)cc1OC	[illegible]
	AMISULPRIDE	CCN1CCCC1CNC(=O)c1cc(S(=O)(=O)CC)c(N)cc1OC	[illegible]
	AMITRIPTYLINE	CN(C)CCC=C1c2ccccc2CCc2ccccc21	[illegible]
	AMITRIPTYLINE	CN(C)CCC=C1c2ccccc2CCc2ccccc21	[illegible]
	AMOXAPINE	Clc1ccc2c(c1)C(N1CCNCC1)=Nc1ccccc1O2	[illegible]
	AMOXAPINE	Clc1ccc2c(c1)C(N1CCNCC1)=Nc1ccccc1O2	[illegible]
	AMOXAPINE	Clc1ccc2c(c1)C(N1CCNCC1)=Nc1ccccc1O2	[illegible]
	AMOXAPINE	Clc1ccc2c(c1)C(N1CCNCC1)=Nc1ccccc1O2	[illegible]
	AMOXAPINE	Clc1ccc2c(c1)C(N1CCNCC1)=Nc1ccccc1O2	[illegible]
	AMOXAPINE	Clc1ccc2c(c1)C(N1CCNCC1)=Nc1ccccc1O2	[illegible]
	AMPRENAVIR	CC(C)CN(C[C@@H](O)[C@H](Cc1ccccc1)NC(=O)O[C@H]1CCOC1)S(=O)(=O)c1ccc(N)cc1	[illegible]
	AMSACRINE	COc1cc(NS(C)(=O)=O)ccc1Nc1c2ccccc2nc2ccccc12	[illegible]
	AMSACRINE	COc1cc(NS(C)(=O)=O)ccc1Nc1c2ccccc2nc2ccccc12	[illegible]
	ANAGLIPTIN	Cc1cc2ncc(C(=O)NCC(C)(C)NCC(=O)N3CCC[C@H]3C#N)cn2n1	[illegible]
	ANAGRELIDE	O=C1CN2Cc3c(ccc(Cl)c3Cl)N=C2N1	[illegible]
	ANASTROZOLE	CC(C)(C#N)c1cc(Cn2cncn2)cc(C(C)(C)C#N)c1	[illegible]
	APALUTAMIDE	CNC(=O)c1ccc(N2C(=S)N(c3cnc(C#N)c(C(F)(F)F)c3)C(=O)C23CCC3)cc1F	[illegible]
	APIXABAN	COc1ccc(-n2nc(C(N)=O)c3c2C(=O)N(c2ccc(N4CCCCC4=O)cc2)CC3)cc1	[illegible]
	APOMORPHINE	CN1CCc2cccc3c2[C@H]1Cc1ccc(O)c(O)c1-3	[illegible]
	APOMORPHINE	CN1CCc2cccc3c2[C@H]1Cc1ccc(O)c(O)c1-3	[illegible]
	APOMORPHINE	CN1CCc2cccc3c2[C@H]1Cc1ccc(O)c(O)c1-3	[illegible]
	APRACLONIDINE	Nc1cc(Cl)c(N=C2NCCN2)c(Cl)c1	[illegible]
	APRACLONIDINE	Nc1cc(Cl)c(N=C2NCCN2)c(Cl)c1	[illegible]
	APRACLONIDINE	Nc1cc(Cl)c(N=C2NCCN2)c(Cl)c1	[illegible]
	APREMILAST	CCOc1cc([C@@H](CS(C)(=O)=O)N2C(=O)c3cccc(NC(C)=O)c3C2=O)ccc1OC	[illegible]
	APREMILAST	CCOc1cc([C@@H](CS(C)(=O)=O)N2C(=O)c3cccc(NC(C)=O)c3C2=O)ccc1OC	[illegible]
	APREPITANT	C[C@@H](O[C@H]1OCCN(Cc2n[nH]c(=O)[nH]2)[C@H]1c1ccc(F)cc1)c1cc(C(F)(F)F)cc(C(F)(F)F)c1	[illegible]
	ARGATROBAN	CC1CNc2c(cccc2S(=O)(=O)N[C@@H](CCCNC(=N)N)C(=O)N2CC[C@@H](C)C[C@@H]2C(=O)O)C1	[illegible]
	ARIPIPRAZOLE	O=C1CCc2ccc(OCCCCN3CCN(c4cccc(Cl)c4Cl)CC3)cc2N1	[illegible]
[illegible]	[illegible]	[illegible]	[illegible]

ENTRECTINIB	CN1CCN(c2ccc(C(=O)Nc3n[nH]c4ccc(Cc5cc(F)cc(F)c5)cc34)c(NC3CCOCC3)c2)CC1	ALK tyrosine kinase receptor
ENTRECTINIB	CN1CCN(c2ccc(C(=O)Nc3n[nH]c4ccc(Cc5cc(F)cc(F)c5)cc34)c(NC3CCOCC3)c2)CC1	[illegible]
ENTRECTINIB	CN1CCN(c2ccc(C(=O)Nc3n[nH]c4ccc(Cc5cc(F)cc(F)c5)cc34)c(NC3CCOCC3)c2)CC1	[illegible]
ENZALUTAMIDE	CNC(=O)c1ccc(N2C(=S)N(c3ccc(C#N)c(C(F)(F)F)c3)C(=O)C2(C)C)cc1F	Androgen Receptor
EPALRESTAT	CC(/C=C1\SC(=S)N(CC(=O)O)C1=O)=C\c1ccccc1	Aldose reductase
EPINEPHRINE	CNC[C@H](O)c1ccc(O)c(O)c1	Alpha-1d adrenergic receptor
EPINEPHRINE	CNC[C@H](O)c1ccc(O)c(O)c1	Alpha-1a adrenergic receptor
EPINEPHRINE	CNC[C@H](O)c1ccc(O)c(O)c1	Alpha-2b adrenergic receptor
EPINEPHRINE	CNC[C@H](O)c1ccc(O)c(O)c1	Alpha-2c adrenergic receptor
EPINEPHRINE	CNC[C@H](O)c1ccc(O)c(O)c1	Beta-2 adrenergic receptor
EPINEPHRINE	CNC[C@H](O)c1ccc(O)c(O)c1	Alpha-2a adrenergic receptor
EPLERENONE	[illegible]	Mineralocorticoid receptor
EPROSARTAN	CCCCc1ncc(/C=C(\Cc2cccs2)C(=O)O)n1Cc1ccc(C(=O)O)cc1	Type-1 angiotensin II receptor
ERDAFITINIB	COc1cc(OC)cc(N(CCNC(C)C)c2ccc3ncc(-c4cnn(C)c4)nc3c2)c1	[illegible]
ERDAFITINIB	COc1cc(OC)cc(N(CCNC(C)C)c2ccc3ncc(-c4cnn(C)c4)nc3c2)c1	[illegible]
ERDAFITINIB	COc1cc(OC)cc(N(CCNC(C)C)c2ccc3ncc(-c4cnn(C)c4)nc3c2)c1	[illegible]
ERDAFITINIB	COc1cc(OC)cc(N(CCNC(C)C)c2ccc3ncc(-c4cnn(C)c4)nc3c2)c1	[illegible]
ERGOTAMINE	[illegible]	Alpha-1d adrenergic receptor
ERGOTAMINE	[illegible]	Serotonin 1d (5-HT1d) receptor
ERGOTAMINE	[illegible]	Alpha-2b adrenergic receptor
ERGOTAMINE	[illegible]	Alpha-2c adrenergic receptor
ERGOTAMINE	[illegible]	Alpha-2a adrenergic receptor
ERLOSAMIDE	COC[C@@H](NC(C)=O)C(=O)NCc1ccccc1	[illegible]
ERLOTINIB	C#Cc1cccc(Nc2ncnc3cc(OCCOC)c(OCCOC)cc23)c1	[illegible]
ERTUGLIFLOZIN	[illegible]	[illegible]
ESCITALOPRAM	CN(C)CCC[C@@]1(c2ccc(F)cc2)OCc2cc(C#N)ccc21	Serotonin transporter
ESFLURBIPROFEN	C[C@H](C(=O)O)c1ccc(-c2ccccc2)c(F)c1	Cyclooxygenase-2
ESMOLOL	COC(=O)CCc1ccc(OCC(O)CNC(C)C)cc1	Beta-1 adrenergic receptor
ESTRADIOL	[illegible]	Estrogen receptor alpha
ESTRIOL	[illegible]	Estrogen receptor beta
ESTRIOL	[illegible]	Estrogen receptor alpha
ESTRONE	[illegible]	Estrogen receptor alpha
ETHINYL ESTRADIOL	[illegible]	Estrogen receptor alpha
ETHOXZOLAMIDE	CCOc1ccc2nc(S(N)(=O)=O)sc2c1	Carbonic anhydrase III
ETHOXZOLAMIDE	CCOc1ccc2nc(S(N)(=O)=O)sc2c1	Carbonic anhydrase VI
ETHOXZOLAMIDE	CCOc1ccc2nc(S(N)(=O)=O)sc2c1	[illegible]
ETHOXZOLAMIDE	CCOc1ccc2nc(S(N)(=O)=O)sc2c1	[illegible]
ETHOXZOLAMIDE	CCOc1ccc2nc(S(N)(=O)=O)sc2c1	[illegible]
ETHOXZOLAMIDE	CCOc1ccc2nc(S(N)(=O)=O)sc2c1	[illegible]
ETHOXZOLAMIDE	CCOc1ccc2nc(S(N)(=O)=O)sc2c1	[illegible]
ETHOXZOLAMIDE	CCOc1ccc2nc(S(N)(=O)=O)sc2c1	[illegible]
ETHOXZOLAMIDE	CCOc1ccc2nc(S(N)(=O)=O)sc2c1	[illegible]
ETHOXZOLAMIDE	CCOc1ccc2nc(S(N)(=O)=O)sc2c1	[illegible]
ETHOXZOLAMIDE	CCOc1ccc2nc(S(N)(=O)=O)sc2c1	[illegible]
ETHOXZOLAMIDE	CCOc1ccc2nc(S(N)(=O)=O)sc2c1	[illegible]
ETODOLAC	CCc1cccc2c3c([nH]c12)C(CC)(CC(=O)O)OCC3	[illegible]
ETOPOSIDE	[illegible]	[illegible]
ETOPOSIDE	[illegible]	[illegible]
ETORICOXIB	Cc1ccc(-c2ncc(Cl)cc2-c2ccc(S(C)(=O)=O)cc2)cn1	[illegible]
EVOGLIPTIN	[illegible]	[illegible]
EXEMESTANE	[illegible]	[illegible]
FADROZOLE	N#Cc1ccc(C2CCCc3cncn32)cc1	[illegible]
FAMOTIDINE	NC(N)=Nc1nc(CSCC/C(N)=N/S(N)(=O)=O)cs1	[illegible]
FEBUXOSTAT	Cc1nc(-c2ccc(OCC(C)C)c(C#N)c2)sc1C(=O)O	[illegible]
FEDRATINIB	Cc1cnc(Nc2ccc(OCCN3CCCC3)cc2)nc1Nc1cccc(S(=O)(=O)NC(C)(C)C)c1	[illegible]
FEDRATINIB	Cc1cnc(Nc2ccc(OCCN3CCCC3)cc2)nc1Nc1cccc(S(=O)(=O)NC(C)(C)C)c1	[illegible]
FENOFIBRIC ACID	CC(C)(Oc1ccc(C(=O)c2ccc(Cl)cc2)cc1)C(=O)O	[illegible]
FENOLDOPAM	Oc1ccc(C2CNCCc3c2cc(O)c(O)c3Cl)cc1	[illegible]
FENOPROFEN	CC(C(=O)O)c1cccc(Oc2ccccc2)c1	[illegible]
FENOTEROL	CC(Cc1ccc(O)cc1)NCC(O)c1cc(O)cc(O)c1	[illegible]
FENTANYL	CCC(=O)N(c1ccccc1)C1CCN(CCc2ccccc2)CC1	[illegible]
FEXOFENADINE	CC(C)(C(=O)O)c1ccc(C(O)CCCN2CCC(C(O)(c3ccccc3)c3ccccc3)CC2)cc1	[illegible]
FINASTERIDE	[illegible]	[illegible]
FINGOLIMOD PHOSPHATE	CCCCCCCCc1ccc(CC[C@](N)(CO)COP(=O)(O)O)cc1	[illegible]
FINGOLIMOD PHOSPHATE	CCCCCCCCc1ccc(CC[C@](N)(CO)COP(=O)(O)O)cc1	[illegible]
FLECAINIDE	O=C(NCC1CCCCN1)c1cc(OCC(F)(F)F)ccc1OCC(F)(F)F	[illegible]
FLUMAZENIL	CCOC(=O)c1ncn2c1CN(C)C(=O)c1cc(F)ccc1-2	[illegible]
FLUMAZENIL	CCOC(=O)c1ncn2c1CN(C)C(=O)c1cc(F)ccc1-2	[illegible]
FLUNISOLIDE	[illegible]	[illegible]
[illegible]	[illegible]	[illegible]

NOREPINEPHRINE	NC[C@H](O)c1ccc(O)c(O)c1	Alpha-2c adrenergic receptor	218_Alpha-2c adrenergic receptor_Homo [illegible]	[illegible]
NOREPINEPHRINE	NC[C@H](O)c1ccc(O)c(O)c1	Beta-2 adrenergic receptor	43_Beta-2 adrenergic receptor_Homo sa[illegible]	[illegible]
NOREPINEPHRINE	NC[C@H](O)c1ccc(O)c(O)c1	Alpha-2a adrenergic receptor	52_Alpha-2a adrenergic receptor_Homo s[illegible]	[illegible]
NORETHINDRONE	C#C[C@]1(O)CC[C@H]2[C@@H]3CCC4=CC(=O)CC[C@@H]4[C@H]3CC[C@@]21C	Progesterone receptor	36_Progesterone receptor_Homo sapiens	[illegible]
NORTRIPTYLINE	CNCCC=C1c2ccccc2CCc2ccccc21	Norepinephrine transporter	100_Norepinephrine transporter_Homo [illegible]	[illegible]
NORTRIPTYLINE	CNCCC=C1c2ccccc2CCc2ccccc21	Serotonin transporter	121_Serotonin transporter_Homo sapiens	[illegible]
OBETICHOLIC ACID	CC[C@@H]1[C@@H]2C[C@H](O)CC[C@]2(C)[C@H]2CC[C@@]3(C)[C@@H](CC[C@@H]3[C@H](C)CCC(=O)O)[C@@H]2[C@@H]1O	Bile acid receptor FXR	11180_Bile acid receptor FXR_Homo sapi[illegible]	[illegible]
OLANZAPINE	Cc1cc2c(s1)Nc1ccccc1N=C2N1CCN(C)CC1	Serotonin 2a (5-HT2a) receptor	107_Serotonin 2a (5-HT2a) receptor_Hom[illegible]	[illegible]
OLANZAPINE	Cc1cc2c(s1)Nc1ccccc1N=C2N1CCN(C)CC1	Serotonin 2c (5-HT2c) receptor	108_Serotonin 2c (5-HT2c) receptor_Hom[illegible]	[illegible]
OLANZAPINE	Cc1cc2c(s1)Nc1ccccc1N=C2N1CCN(C)CC1	Dopamine D3 receptor	130_Dopamine D3 receptor_Homo sapien[illegible]	[illegible]
OLANZAPINE	Cc1cc2c(s1)Nc1ccccc1N=C2N1CCN(C)CC1	Dopamine D2 receptor	72_Dopamine D2 receptor_Homo sapiens	[illegible]
OLANZAPINE	Cc1cc2c(s1)Nc1ccccc1N=C2N1CCN(C)CC1	Dopamine D4 receptor	90_Dopamine D4 receptor_Homo sapiens	[illegible]
OLAPARIB	O=C(c1cc(Cc2n[nH]c(=O)c3ccccc23)ccc1F)N1CCN(C(=O)C2CC2)CC1	Poly [ADP-ribose] polymerase 2	100933_Poly [ADP-ribose] polymerase 2_[illegible]	[illegible]
OLAPARIB	O=C(c1cc(Cc2n[nH]c(=O)c3ccccc23)ccc1F)N1CCN(C(=O)C2CC2)CC1	Poly [ADP-ribose] polymerase-1	11663_Poly [ADP-ribose] polymerase-1_H[illegible]	[illegible]
OMARIGLIPTIN	CS(=O)(=O)n1cc2c(n1)CN([C@H]1CO[C@H](c3cc(F)ccc3F)[C@@H](N)C1)C2	Dipeptidyl peptidase IV	11140_Dipeptidyl peptidase IV_Homo sap[illegible]	[illegible]
OMBITASVIR	COC(=O)N[C@H](C(=O)N1CCC[C@H]1C(=O)Nc1ccc([C@@H]2CC[C@@H](c3ccc(NC(=O)[C@@H]4CCCN4C(=O)[C@@H](NC(=O)OC)C(C)C)cc3)N2c2ccc(C(C)(C)C)cc2)cc1)C(C)C	Nonstructural protein 5A	108438_Nonstructural protein 5A_Hepati[illegible]	[illegible]
ONAPRISTONE	CN(C)c1ccc([C@H]2C[C@]3(C)[C@@H](CC[C@]3(O)CCCO)[C@@H]3CCC4=CC(=O)CCC4=C32)cc1	Progesterone receptor	36_Progesterone receptor_Homo sapiens	[illegible]
ONDANSETRON	Cc1nccn1CC1CCc2c(c3ccccc3n2C)C1=O	Serotonin 3a (5-HT3a) receptor	144_Serotonin 3a (5-HT3a) receptor_Hom[illegible]	[illegible]
ORPHENADRINE	Cc1ccccc1C(OCCN(C)C)c1ccccc1	Norepinephrine transporter	100_Norepinephrine transporter_Homo s[illegible]	[illegible]
ORPHENADRINE	Cc1ccccc1C(OCCN(C)C)c1ccccc1	Glutamate [NMDA] receptor	104706_Glutamate [NMDA] receptor_Ho[illegible]	[illegible]
ORPHENADRINE	Cc1ccccc1C(OCCN(C)C)c1ccccc1	Histamine H1 receptor	127_Histamine H1 receptor_Homo sapien[illegible]	[illegible]
OSELTAMIVIR CARBOXYLIC ACID	CCC(CC)O[C@@H]1C=C(C(=O)O)C[C@H](N)[C@H]1NC(C)=O	Neuraminidase	10738_Neuraminidase_Influenza B virus ([illegible]	[illegible]
OSELTAMIVIR CARBOXYLIC ACID	CCC(CC)O[C@@H]1C=C(C(=O)O)C[C@H](N)[C@H]1NC(C)=O	Neuraminidase	21_Neuraminidase_Influenza A virus (A/P[illegible]	[illegible]
OSILODROSTAT	N#Cc1ccc([C@H]2CCc3cncn32)c(F)c1	Cytochrome P450 11B1	76_Cytochrome P450 11B1_Homo sapiens	[illegible]
OSIMERTINIB	C=CC(=O)Nc1cc(Nc2nccc(-c3cn(C)c4ccccc34)n2)c(OC)cc1N(C)CCN(C)C	Epidermal growth factor receptor erbB1	9-T790M_Epidermal growth factor recept[illegible]	[illegible]
OXAPROZIN	O=C(O)CCc1nc(-c2ccccc2)c(-c2ccccc2)o1	Cyclooxygenase-1	96_Cyclooxygenase-1_Homo sapiens	[illegible]
OXYBUTYNIN	CCN(CC)CC#CCOC(=O)C(O)(c1ccccc1)C1CCCCC1	Muscarinic acetylcholine receptor M3	219_Muscarinic acetylcholine receptor M[illegible]	[illegible]
OXYBUTYNIN	CCN(CC)CC#CCOC(=O)C(O)(c1ccccc1)C1CCCCC1	Muscarinic acetylcholine receptor M2	47_Muscarinic acetylcholine receptor M2[illegible]	[illegible]
OXYCODONE	COc1ccc2c3c1O[C@H]1C(=O)CC[C@@]4(O)[C@@H](C2)N(C)CC[C@]314	Mu opioid receptor	129_Mu opioid receptor_Homo sapiens	[illegible]
OXYMETAZOLINE	Cc1cc(C(C)(C)C)c(O)c(C)c1CC1=NCCN1	Alpha-1d adrenergic receptor	103_Alpha-1d adrenergic receptor_Homo[illegible]	[illegible]
OXYMETAZOLINE	Cc1cc(C(C)(C)C)c(O)c(C)c1CC1=NCCN1	Alpha-1a adrenergic receptor	125_Alpha-1a adrenergic receptor_Homo[illegible]	[illegible]
OXYMETAZOLINE	Cc1cc(C(C)(C)C)c(O)c(C)c1CC1=NCCN1	Alpha-1b adrenergic receptor	128_Alpha-1b adrenergic receptor_Homo[illegible]	[illegible]
OXYMETAZOLINE	Cc1cc(C(C)(C)C)c(O)c(C)c1CC1=NCCN1	Alpha-2b adrenergic receptor	216_Alpha-2b adrenergic receptor_Homo[illegible]	[illegible]
OXYMETAZOLINE	Cc1cc(C(C)(C)C)c(O)c(C)c1CC1=NCCN1	Alpha-2c adrenergic receptor	218_Alpha-2c adrenergic receptor_Homo[illegible]	[illegible]
OXYMETAZOLINE	Cc1cc(C(C)(C)C)c(O)c(C)c1CC1=NCCN1	Alpha-2a adrenergic receptor	52_Alpha-2a adrenergic receptor_Homo s[illegible]	[illegible]
[illegible]	[illegible]	[illegible]	[illegible]	[illegible]

TRELAGLIPTIN	Cn1c(=O)cc(N2CCC[C@@H](N)C2)n(Cc2cc(F)ccc2C#N)c1=O	Dipeptidyl peptidase IV	11140_Dipeptidyl peptidase IV_Homo sapiens	3731	[illegible]
TRETINOIN	CC1=C(/C=C/C(C)=C/C=C/C(C)=C/C(=O)O)C(C)(C)CCC1	Retinoic acid receptor gamma	240_Retinoic acid receptor gamma_Homo sapiens	242	[illegible]
TRETINOIN	CC1=C(/C=C/C(C)=C/C=C/C(C)=C/C(=O)O)C(C)(C)CCC1	Retinoic acid receptor beta	270_Retinoic acid receptor beta_Homo sapiens	225	[illegible]
TRETINOIN	CC1=C(/C=C/C(C)=C/C=C/C(C)=C/C(=O)O)C(C)(C)CCC1	Retinoic acid receptor alpha	57_Retinoic acid receptor alpha_Homo sapiens	195	[illegible]
TRIAMCINOLONE	C[C@]12C[C@H](O)[C@@]3(F)[C@@H](CCC4=CC(=O)C=C[C@@]43C)[C@@H]1C[C@@H](O)[C@]2(O)C(=O)CO	Glucocorticoid receptor	25_Glucocorticoid receptor_Homo sapiens	2113	[illegible]
TRIAMCINOLONE ACETONIDE	CC1(C)O[C@@H]2C[C@H]3[C@@H]4CCC5=CC(=O)C=C[C@]5(C)[C@@]4(F)[C@@H](O)C[C@]3(C)[C@]2(C(=O)CO)O1	Glucocorticoid receptor	25_Glucocorticoid receptor_Homo sapiens	2113	[illegible]
TRIAZOLAM	Cc1nnc2n1-c1ccc(Cl)cc1C(c1ccccc1Cl)=NC2	GABA receptor alpha-5 subunit	11440_GABA receptor alpha-5 subunit_Homo sapiens	438	[illegible]
TRICLOSAN	Oc1cc(Cl)ccc1Oc1ccc(Cl)cc1Cl	Enoyl-[acyl-carrier-protein] reductase	110_Enoyl-[acyl-carrier-protein] reductase_Escherichia coli K-12	103	[illegible]
TRIHEXYPHENIDYL	OC(CCN1CCCCC1)(c1ccccc1)C1CCCCC1	Muscarinic acetylcholine receptor M1	61_Muscarinic acetylcholine receptor M1_Homo sapiens	1329	[illegible]
TRIMETREXATE	COc1cc(NCc2ccc3nc(N)nc(N)c3c2C)cc(OC)c1OC	Dihydrofolate reductase	10460_Dihydrofolate reductase_Pneumocystis carinii	584	[illegible]
TRIPELENNAMINE	CN(C)CCN(Cc1ccccc1)c1ccccn1	Histamine H1 receptor	127_Histamine H1 receptor_Homo sapiens	1072	[illegible]
TRIPROLIDINE	Cc1ccc(/C(=C\CN2CCCC2)c2ccccn2)cc1	Histamine H1 receptor	127_Histamine H1 receptor_Homo sapiens	1072	[illegible]
TROGLITAZONE	Cc1c(C)c2c(c(C)c1O)CCC(C)(COc1ccc(CC3SC(=O)NC3=O)cc1)O2	Peroxisome proliferator-activated receptor gamma	133_Peroxisome proliferator-activated receptor gamma_Homo sapiens	2617	[illegible]
TROPICAMIDE	CCN(Cc1ccncc1)C(=O)C(CO)c1ccccc1	Muscarinic acetylcholine receptor M3	219_Muscarinic acetylcholine receptor M3_Homo sapiens	1500	[illegible]
UMECLIDINIUM	OC(c1ccccc1)(c1ccccc1)C12CC[N+](CCOCc3ccccc3)(CC1)CC2	Muscarinic acetylcholine receptor M3	219_Muscarinic acetylcholine receptor M3_Homo sapiens	1500	[illegible]
UPADACITINIB	CC[C@@H]1CN(C(=O)NCC(F)(F)F)C[C@@H]1c1cnc2cnc3[nH]ccc3n12	Tyrosine-protein kinase JAK1	10919-WT_Tyrosine-protein kinase JAK1_Homo sapiens	3955	[illegible]
VALDECOXIB	Cc1onc(-c2ccccc2)c1-c1ccc(S(N)(=O)=O)cc1	Cyclooxygenase-2	126_Cyclooxygenase-2_Homo sapiens	3122	[illegible]
VALSARTAN	CCCCC(=O)N(Cc1ccc(-c2ccccc2-c2nnn[nH]2)cc1)[C@H](C(=O)O)C(C)C	Type-1 angiotensin II receptor	115_Type-1 angiotensin II receptor_Homo sapiens	659	[illegible]
VANDETANIB	COc1cc2c(Nc3ccc(Br)cc3F)ncnc2cc1OCC1CCN(C)CC1	Tyrosine-protein kinase receptor RET	100079-WT_Tyrosine-protein kinase receptor RET_Homo sapiens	571	[illegible]
VANDETANIB	COc1cc2c(Nc3ccc(Br)cc3F)ncnc2cc1OCC1CCN(C)CC1	Ephrin type-B receptor 4	100129-WT_Ephrin type-B receptor 4_Homo sapiens	717	[illegible]
VANDETANIB	COc1cc2c(Nc3ccc(Br)cc3F)ncnc2cc1OCC1CCN(C)CC1	Tyrosine-protein kinase SRC	10434-WT_Tyrosine-protein kinase SRC_Homo sapiens	2944	[illegible]
VANDETANIB	COc1cc2c(Nc3ccc(Br)cc3F)ncnc2cc1OCC1CCN(C)CC1	Vascular endothelial growth factor receptor 1	10979-WT_Vascular endothelial growth factor receptor 1_Homo sapiens	837	[illegible]
VANDETANIB	COc1cc2c(Nc3ccc(Br)cc3F)ncnc2cc1OCC1CCN(C)CC1	Vascular endothelial growth factor receptor 2	10980-WT_Vascular endothelial growth factor receptor 2_Homo sapiens	7468	[illegible]
VANDETANIB	COc1cc2c(Nc3ccc(Br)cc3F)ncnc2cc1OCC1CCN(C)CC1	Vascular endothelial growth factor receptor 3	11752-WT_Vascular endothelial growth factor receptor 3_Homo sapiens	382	[illegible]
VANDETANIB	COc1cc2c(Nc3ccc(Br)cc3F)ncnc2cc1OCC1CCN(C)CC1	Receptor protein-tyrosine kinase erbB-4	12699-WT_Receptor protein-tyrosine kinase erbB-4_Homo sapiens	176	[illegible]
VANDETANIB	COc1cc2c(Nc3ccc(Br)cc3F)ncnc2cc1OCC1CCN(C)CC1	Tyrosine-protein kinase TIE-2	12913-WT_Tyrosine-protein kinase TIE-2_Homo sapiens	790	[illegible]
VANDETANIB	COc1cc2c(Nc3ccc(Br)cc3F)ncnc2cc1OCC1CCN(C)CC1	Receptor protein-tyrosine kinase erbB-2	188-WT_Receptor protein-tyrosine kinase erbB-2_Homo sapiens	1687	[illegible]
VANDETANIB	COc1cc2c(Nc3ccc(Br)cc3F)ncnc2cc1OCC1CCN(C)CC1	Ephrin type-A receptor 2	30008-WT_Ephrin type-A receptor 2_Homo sapiens	373	[illegible]
VANDETANIB	COc1cc2c(Nc3ccc(Br)cc3F)ncnc2cc1OCC1CCN(C)CC1	Tyrosine-protein kinase BRK	30026-WT_Tyrosine-protein kinase BRK_Homo sapiens	157	[illegible]
VANDETANIB	COc1cc2c(Nc3ccc(Br)cc3F)ncnc2cc1OCC1CCN(C)CC1	Epidermal growth factor receptor erbB1	9-WT_Epidermal growth factor receptor erbB1_Homo sapiens	5409	[illegible]
VARDENAFIL	CCCc1nc(C)c2c(=O)nc(-c3cc(S(=O)(=O)N4CCN(CC)CC4)ccc3OCC)[nH]n12	Phosphodiesterase 5A	3_Phosphodiesterase 5A_Homo sapiens	1769	[illegible]
VARENICLINE	c1cnc2cc3c(cc2n1)C1CNCC3C1	Neuronal acetylcholine receptor; alpha4/beta2	104283_Neuronal acetylcholine receptor; alpha4/beta2_Homo sapiens	702	[illegible]
VEMURAFENIB	CCCS(=O)(=O)Nc1ccc(F)c(C(=O)c2c[nH]c3ncc(-c4ccc(Cl)cc4)cc23)c1F	Serine/threonine-protein kinase B-raf	100126-V600E_Serine/threonine-protein kinase B-raf_Homo sapiens	1471	[illegible]
VENETOCLAX	CC1(C)CCC(CN2CCN(c3ccc(C(=O)NS(=O)(=O)c4ccc(NCC5CCOCC5)c([N+](=O)[O-])c4)c(Oc4cnc5[nH]ccc5c4)c3)CC2)=C(c2ccc(Cl)cc2)C1	Apoptosis regulator Bcl-2	100304_Apoptosis regulator Bcl-2_Homo sapiens	1591	[illegible]
VENLAFAXINE	COc1ccc(C(CN(C)C)C2(O)CCCCC2)cc1	Norepinephrine transporter	100_Norepinephrine transporter_Homo sapiens	2947	[illegible]
VENLAFAXINE	COc1ccc(C(CN(C)C)C2(O)CCCCC2)cc1	Serotonin transporter	121_Serotonin transporter_Homo sapiens	4301	[illegible]
VERAPAMIL	COc1ccc(CCN(C)CCCC(C#N)(c2ccc(OC)c(OC)c2)C(C)C)cc1OC	Voltage-gated L-type calcium channel alpha-1C subunit	169_Voltage-gated L-type calcium channel alpha-1C subunit_Homo sapiens	137	[illegible]
VIBEGRON	O=C(Nc1ccc(C[C@@H]2CC[C@H]([C@H](O)c3ccccc3)N2)cc1)[C@@H]1CCc2nccc(=O)n21	Beta-3 adrenergic receptor	226_Beta-3 adrenergic receptor_Homo sapiens	452	[illegible]
VILAZODONE	N#Cc1ccc2[nH]cc(CCCCN3CCN(c4ccc5oc(C(N)=O)cc5c4)CC3)c2c1	Serotonin transporter	121_Serotonin transporter_Homo sapiens	4301	[illegible]
VILAZODONE	N#Cc1ccc2[nH]cc(CCCCN3CCN(c4ccc5oc(C(N)=O)cc5c4)CC3)c2c1	Serotonin 1a (5-HT1a) receptor	51_Serotonin 1a (5-HT1a) receptor_Homo sapiens	3774	[illegible]
VILDAGLIPTIN	N#C[C@@H]1CCCN1C(=O)CNC12CC3CC(CC(O)(C3)C1)C2	Dipeptidyl peptidase IV	11140_Dipeptidyl peptidase IV_Homo sapiens	3731	[illegible]
VINBLASTINE	CC[C@]1(O)C[C@@H]2CN(CCc3c([nH]c4ccccc34)[C@@](C(=O)OC)(c3cc4c(cc3OC)N(C)[C@H]3[C@@](O)(C(=O)OC)[C@H](OC(C)=O)[C@]5(CC)C=CCN6CC[C@]43[C@@H]65)C2)C1	Tubulin	104766_Tubulin_Homo sapiens	517	[illegible]
VINORELBINE	CCC1=C[C@@H]2CN(C1)Cc1c([nH]c3ccccc13)[C@@](C(=O)OC)(c1cc3c(cc1OC)N(C)[C@H]1[C@@](O)(C(=O)OC)[C@H](OC(C)=O)[C@]4(CC)C=CCN5CC[C@]31[C@@H]54)C2	Tubulin	104766_Tubulin_Homo sapiens	517	[illegible]
VISMODEGIB	CS(=O)(=O)c1ccc(C(=O)Nc2ccc(Cl)c(-c3ccccn3)c2)c(Cl)c1	Smoothened homolog	101400_Smoothened homolog_Homo sapiens	568	[illegible]
VOGLIBOSE	OCC(CO)N[C@H]1C[C@](O)(CO)[C@@H](O)[C@H](O)[C@H]1O	Maltase-glucoamylase	1_Maltase-glucoamylase_Homo sapiens	127	[illegible]
VORAPAXAR	CCOC(=O)N[C@@H]1CC[C@@H]2[C@@H](C1)C[C@H]1C(=O)O[C@H](C)[C@H]1[C@H]2/C=C/c1ccc(-c2cccc(F)c2)cn1	Proteinase-activated receptor 1	10653_Proteinase-activated receptor 1_Homo sapiens	771	[illegible]
VORINOSTAT	O=C(CCCCCCC(=O)Nc1ccccc1)NO	Histone deacetylase 2	11206_Histone deacetylase 2_Homo sapiens	1172	[illegible]
VORINOSTAT	O=C(CCCCCCC(=O)Nc1ccccc1)NO	Histone deacetylase 3	11208_Histone deacetylase 3_Homo sapiens	1287	[illegible]
VORINOSTAT	O=C(CCCCCCC(=O)Nc1ccccc1)NO	Histone deacetylase 6	11307_Histone deacetylase 6_Homo sapiens	2473	[illegible]
VORINOSTAT	O=C(CCCCCCC(=O)Nc1ccccc1)NO	Histone deacetylase 1	12697_Histone deacetylase 1_Homo sapiens	3935	[illegible]
VORTIOXETINE	Cc1ccc(Sc2ccccc2N2CCNCC2)c(C)c1	Serotonin transporter	121_Serotonin transporter_Homo sapiens	4301	[illegible]
VORTIOXETINE	Cc1ccc(Sc2ccccc2N2CCNCC2)c(C)c1	Serotonin 3a (5-HT3a) receptor	144_Serotonin 3a (5-HT3a) receptor_Homo sapiens	669	[illegible]
VORTIOXETINE	Cc1ccc(Sc2ccccc2N2CCNCC2)c(C)c1	Serotonin 1a (5-HT1a) receptor	51_Serotonin 1a (5-HT1a) receptor_Homo sapiens	3774	[illegible]
ZAFIRLUKAST	COc1cc(C(=O)NS(=O)(=O)c2ccccc2C)ccc1Cc1cn(C)c2ccc(NC(=O)OC3CCCC3)cc12	Cysteinyl leukotriene receptor 1	179_Cysteinyl leukotriene receptor 1_Homo sapiens	111	[illegible]
ZANAMIVIR	CC(=O)N[C@@H]1[C@@H](NC(=N)N)C=C(C(=O)O)O[C@H]1[C@H](O)[C@H](O)CO	Neuraminidase	10738_Neuraminidase_Influenza B virus (strain B/Lee/1940)	194	[illegible]
ZANAMIVIR	CC(=O)N[C@@H]1[C@@H](NC(=N)N)C=C(C(=O)O)O[C@H]1[C@H](O)[C@H](O)CO	Neuraminidase	21_Neuraminidase_Influenza A virus (A/Puerto Rico/8/1934(H1N1))	320	[illegible]
ZANUBRUTINIB	C=CC(=O)N1CCC([C@@H]2CCNc3c(C(N)=O)c(-c4ccc(Oc5ccccc5)cc4)nn32)CC1	Tyrosine-protein kinase BTK	100097-WT_Tyrosine-protein kinase BTK_Homo sapiens	2128	[illegible]
ZIDOVUDINE TRIPHOSPHATE	Cc1cn([C@H]2C[C@H](N=[N+]=[N-])[C@@H](COP(=O)(O)OP(=O)(O)OP(=O)(O)O)O2)c(=O)[nH]c1=O	Human immunodeficiency virus type 1 reverse transcriptase	228_Human immunodeficiency virus type 1 reverse transcriptase_Human immunodeficiency virus 1	2036	[illegible]
ZILEUTON	CC(c1cc2ccccc2s1)N(O)C(N)=O	Arachidonate 5-lipoxygenase	55_Arachidonate 5-lipoxygenase_Homo sapiens	1428	[illegible]
ZIPRASIDONE	O=C1Cc2cc(CCN3CCN(c4nsc5ccccc45)CC3)c(Cl)cc2N1	Serotonin 2a (5-HT2a) receptor	107_Serotonin 2a (5-HT2a) receptor_Homo sapiens	3893	[illegible]
ZIPRASIDONE	O=C1Cc2cc(CCN3CCN(c4nsc5ccccc45)CC3)c(Cl)cc2N1	Serotonin 2c (5-HT2c) receptor	108_Serotonin 2c (5-HT2c) receptor_Homo sapiens	2763	[illegible]
ZIPRASIDONE	O=C1Cc2cc(CCN3CCN(c4nsc5ccccc45)CC3)c(Cl)cc2N1	Dopamine D2 receptor	72_Dopamine D2 receptor_Homo sapiens	6009	[illegible]
ZOLEDRONIC ACID	O=P(O)(O)C(O)(Cn1ccnc1)P(=O)(O)O	Farnesyl diphosphate synthase	71_Farnesyl diphosphate synthase_Homo sapiens	217	[illegible]
ZOLMITRIPTAN	CN(C)CCc1c[nH]c2ccc(C[C@H]3COC(=O)N3)cc12	Serotonin 1d (5-HT1d) receptor	105_Serotonin 1d (5-HT1d) receptor_Homo sapiens	1136	[illegible]
ZOLMITRIPTAN	CN(C)CCc1c[nH]c2ccc(C[C@H]3COC(=O)N3)cc12	Serotonin 1b (5-HT1b) receptor	106_Serotonin 1b (5-HT1b) receptor_Homo sapiens	942	[illegible]

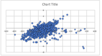

Supplement: Supp drug target and drug_target data [file EMS123358-supplement-Supp_drug_target_and_drug_target_data.pdf]
