# Supplementary material for: Target-based evaluation of ‘drug-like’ properties and ligand efficiencies
Source: J Med Chem. Author manuscript; Available in PMC 2021 Jun 11. (PMC7610969; doi:10.1021/acs.jmedchem.1c00416)
Supplement: Supp Fig 8 values AlogP [file EMS123358-supplement-Supp_Fig_8_values_AlogP.pdf]

ALogP	Approval period	Group	Total Count	Outlier Count	Mean Value	1st Quartile	Median	3rd Quartile	Lower Adjacent Limit	Upper Adjacent Limit	Standard Deviation	Confidence Interval (95%)
Carboaromatic drugs	1939-1989	Drug	151	1	2.8513	1.7525	2.96	3.8675	-0.35	6.26	1.468	2.6171-3.0854
Carboaromatic drugs	1939-1989	Target median	151	13	3.9462	3.73	3.86	4.22	3.29	4.95	0.56645	3.8558-4.0365
Carboaromatic drugs	1939-1989	[Drug - target median]	151	1	-1.0949	-2.0138	-0.92	-0.085	-4.03	1.88	1.353	-1.3107--0.87913
Carboaromatic drugs	1990-2009	Drug	98	3	3.1692	2.38	3.045	4.07	0.4	6.14	1.5776	2.8568-3.4815
Carboaromatic drugs	1990-2009	Target median	98	19	3.9778	3.75	3.985	4.26	3.04	4.95	0.78339	3.8227-4.1329
Carboaromatic drugs	1990-2009	[Drug - target median]	98	2	-0.80857	-1.64	-0.8625	-0.01	-3.92	1.84	1.3655	-1.0789--0.53822
Carboaromatic drugs	2010-2020	Drug	29	1	3.3645	1.9925	3.32	4.035	1	6.77	1.6644	2.7587-3.9703
Carboaromatic drugs	2010-2020	Target median	29	2	3.7731	3.235	3.9	4.155	2.08	5.39	1.0789	3.3804-4.1658
Carboaromatic drugs	2010-2020	[Drug - target median]	29	1	-0.40862	-1.0975	-0.47	0.0925	-2.47	1.06	1.0302	-0.78356--0.033683
Heteroaromatic drugs	1939-1989	Drug	65	0	2.0855	0.5925	2.09	3.705	-2.3	6.94	1.9767	1.605-2.5661
Heteroaromatic drugs	1939-1989	Target median	65	7	3.5995	3.335	3.82	4.01	2.75	4.41	0.72129	3.4242-3.7749
Heteroaromatic drugs	1939-1989	[Drug - target median]	65	1	-1.514	-2.6675	-1.37	-0.0675	-4.78	2.97	1.8254	-1.9578--1.0702
Heteroaromatic drugs	1990-2009	Drug	116	2	3.2424	2.045	3.15	4.41	-1.12	7.26	1.83	2.9094-3.5754
Heteroaromatic drugs	1990-2009	Target median	116	10	3.949	3.42	3.93	4.345	2.08	5.72	1.1851	3.7333-4.1647
Heteroaromatic drugs	1990-2009	[Drug - target median]	116	1	-0.70659	-1.675	-0.56	0.195	-3.935	2.16	1.3485	-0.952--0.46119
Heteroaromatic drugs	2010-2020	Drug	107	1	3.7582	2.7775	3.91	4.91	0.2	6.79	1.5479	3.4649-4.0515
Heteroaromatic drugs	2010-2020	Target median	107	2	3.8114	3.115	4.005	4.3475	2.08	5.69	0.93535	3.6342-3.9887
Heteroaromatic drugs	2010-2020	[Drug - target median]	107	0	-0.05322	-0.82	0.09	0.8	-2.37	1.91	1.0533	-0.2528-0.14635
