# Supplementary material for: Target-based evaluation of ‘drug-like’ properties and ligand efficiencies
Source: J Med Chem. Author manuscript; Available in PMC 2021 Jun 11. (PMC7610969; doi:10.1021/acs.jmedchem.1c00416)
Supplement: Supp Fig S2 values [file EMS123358-supplement-Supp_Fig_S2_values.pdf]

Property	Approval period	Group	Total Count	Outlier Count	Mean Value	1st Quartile	Median	3rd Quartile	Lower Adjacent Limit	Upper Adjacent Limit	Standard Deviation	Confidence Interval (95%)
HA	1939-1989	Drug	259	10	22.579	18	22	26	8	36	8.2765	21.571-23.587
HA	1939-1989	Target	259	0	28.019	24	28	32	15	42	4.1217	27.517-28.521
HA	1939-1989	[Drug - target median]	259	15	-5.4402	-9	-5	-2	-19	7	7.8547	-6.3968--4.4835
HA	1990-2009	Drug	243	9	27.169	20	26	31.75	11	49	9.4358	25.982-28.355
HA	1990-2009	Target	243	9	29.235	25	29	32	15	42	5.696	28.518-29.951
HA	1990-2009	[Drug - target median]	243	11	-2.0658	-6	-3	1	-16	11	7.5829	-3.0193--1.1124
HA	2010-2020	Drug	141	6	31.844	27	32	36	16	45	8.0163	30.521-33.167
HA	2010-2020	Target	141	8	31.752	28.75	31	34	21	41	6.3843	30.698-32.806
HA	2010-2020	[Drug - target median]	141	1	0.092199	-3	0	4	-13	11	4.8784	-0.71304-0.89743
LogD7.4	1939-1989	Drug	259	8	1.1249	0.03	1.37	2.5325	-3.58	5.94	2.1221	0.86649-1.3834
LogD7.4	1939-1989	Target	259	9	2.7056	2.05	2.59	3.325	0.2	4.865	1.0557	2.577-2.8342
LogD7.4	1939-1989	[Drug - target median]	259	5	-1.5807	-2.7275	-1.4	-0.2425	-6.355	2.72	1.9781	-1.8216--1.3398
LogD7.4	1990-2009	Drug	243	16	1.2914	0.0725	1.68	3.03	-4.21	7.45	2.9349	0.92238-1.6604
LogD7.4	1990-2009	Target	243	17	2.4597	1.97	2.75	3.48	0.2	5.38	1.7786	2.2361-2.6833
LogD7.4	1990-2009	[Drug - target median]	243	10	-1.1683	-1.91	-1.03	0.17	-4.99	2.78	2.1958	-1.4444--0.89222
LogD7.4	2010-2020	Drug	141	3	2.2873	1.03	2.48	3.565	-2.25	6.88	2.0835	1.9434-2.6312
LogD7.4	2010-2020	Target	141	3	2.6637	2.0075	2.84	3.53	0.31	5.38	1.2375	2.4594-2.8679
LogD7.4	2010-2020	[Drug - target median]	141	3	-0.37638	-1.2375	-0.28	0.6525	-3.44	3.315	1.4615	-0.61762--0.13515
HBA	1939-1989	Drug	259	11	3.8263	2	3	5	1	9	2.3454	3.5406-4.1119
HBA	1939-1989	Target	259	7	4.39	4	4	5	3	6	1.074	4.2592-4.5208
HBA	1939-1989	[Drug - target median]	259	7	-0.56371	-2	-1	1	-5	5	2.1036	-0.8199--0.30751
HBA	1990-2009	Drug	243	9	4.8025	3	4	6	1	10	2.5247	4.485-5.1199
HBA	1990-2009	Target	243	0	4.6276	4	4	6	2	8	1.2589	4.4693-4.7859
HBA	1990-2009	[Drug - target median]	243	10	0.1749	-1	0	1	-4	3	2.1569	-0.096296-0.44609
HBA	2010-2020	Drug	141	1	5.9504	4	6	7	1	11	2.3884	5.5561-6.3446
HBA	2010-2020	Target	141	0	5.844	5	6	7	3	9	1.4042	5.6122-6.0758
HBA	2010-2020	[Drug - target median]	141	2	0.10638	-1	0	1	-4	4	1.7524	-0.18286-0.39563
HBD	1939-1989	Drug	259	25	1.6178	1	1	2	0	3	1.6201	1.4205-1.8151
HBD	1939-1989	Target	259	68	1.3282	1	1	1	1	1	0.87848	1.2212-1.4352
HBD	1939-1989	[Drug - target median]	259	3	0.28958	-1	0	1	-2	4	1.4697	0.11058-0.46857
HBD	1990-2009	Drug	243	3	2.0247	1	2	3	0	6	1.6437	1.818-2.2314
HBD	1990-2009	Target	243	26	1.6461	1	1	2	0	3	1.0554	1.5134-1.7788
HBD	1990-2009	[Drug - target median]	243	13	0.3786	0	0	1	-1	2	1.255	0.2208-0.5364
HBD	2010-2020	Drug	141	2	2.078	1	2	3	0	6	1.326	1.8591-2.2969
HBD	2010-2020	Target	141	16	1.9433	1	2	2	0	3	1.0055	1.7773-2.1092
HBD	2010-2020	[Drug - target median]	141	9	0.13475	0	0	1	-1	2	1.0638	-0.040844-0.31035
