# Supplementary material for: Target-based evaluation of ‘drug-like’ properties and ligand efficiencies
Source: J Med Chem. Author manuscript; Available in PMC 2021 Jun 11. (PMC7610969; doi:10.1021/acs.jmedchem.1c00416)
Supplement: Supp Fig S3 values Mol Wt [file EMS123358-supplement-Supp_Fig_S3_values_Mol_Wt.pdf]

Mol Wt

Approval period	Target class	Group	Total Count	Outlier Count	Mean Value	1st Quartile	Median	3rd Quartile	Lower Adjacent Limit	Upper Adjacent Limit	Standard Deviation	Confidence Interval (95%)
1939-89	Enzyme_Hydrolase	Drug	7	1	249.96	169.99	184.15	292.63	137.16	298.39	127.76	155.31-344.6
1939-89	Enzyme_Hydrolase	Target median	7	1	387	394.52	394.52	394.52	394.52	394.52	19.904	372.26-401.75
1939-89	Enzyme_Hydrolase	[Drug - target median]	7	1	-137.05	-224.54	-210.38	-101.9	-257.36	-96.135	145.72	-245--29.094
1939-89	Enzyme_Other	Drug	32	4	301.23	236.28	258.32	305.16	222.25	393.47	103.19	265.48-336.98
1939-89	Enzyme_Other	Target median	32	0	367.85	349.8	376.43	393.48	303.32	416.47	30.335	357.34-378.36
1939-89	Enzyme_Other	[Drug - target median]	32	3	-66.62	-128.31	-87.12	-49.323	-171.23	47.13	91.602	-98.358--34.881
1939-89	Enzyme_Oxidoreductase	Drug	46	2	257.24	206.28	248.52	296.15	136.2	357.79	80.852	233.87-280.6
1939-89	Enzyme_Oxidoreductase	Target median	46	1	355.92	350.44	357.42	381.38	310.43	398.4	27.831	347.88-363.97
1939-89	Enzyme_Oxidoreductase	[Drug - target median]	46	2	-98.685	-140.09	-114.15	-70.14	-201.22	6.91	70.192	-118.97--78.4
1939-89	Enzyme_Phosphodiesterase	Drug	9	0	314.06	225.54	230.31	504.64	180.17	504.64	143.84	220.09-408.04
1939-89	Enzyme_Phosphodiesterase	Target median	9	1	409.18	393.8	403.51	417.46	387.48	430.9	21.749	394.97-423.39
1939-89	Enzyme_Phosphodiesterase	[Drug - target median]	9	0	-95.121	-190.18	-175.18	80.587	-225.68	110.53	144.18	-189.32--0.92304
1939-89	Enzyme_Protease	Drug	7	0	306.92	172.23	348.4	434.71	131.18	444.44	135.77	206.35-407.5
1939-89	Enzyme_Protease	Target median	7	0	447.88	397.61	449.53	501.86	397.61	514.63	52.821	408.75-487.01
1939-89	Enzyme_Protease	[Drug - target median]	7	0	-140.96	-313.15	-49.21	-8.5925	-383.45	7.89	168.86	-266.05--15.861
1939-89	Ion channel	Drug	24	1	283.53	179.26	277.07	332.93	151.25	454.61	112.19	238.65-328.42
1939-89	Ion channel	Target median	24	0	375.6	308.3	387.82	444.77	232.3	467.42	76.632	344.94-406.26
1939-89	Ion channel	[Drug - target median]	24	1	-92.068	-141.18	-79.833	-33.87	-288.16	29.2	107.59	-135.11--49.022
1939-89	Membrane receptor_GPCR_amine	Drug	180	7	309.78	259.86	309.47	363.01	111.15	461.56	88.278	296.88-322.68
1939-89	Membrane receptor_GPCR_amine	Target median	180	2	405.42	372.99	414.53	451.57	329.44	484.08	50.379	398.06-412.78
1939-89	Membrane receptor_GPCR_amine	[Drug - target median]	180	8	-95.635	-151.7	-94.09	-46.595	-295.38	72.33	100.03	-110.25--81.022
1939-89	Membrane receptor_GPCR_other	Drug	23	0	260.56	194.19	267.25	342.97	180.17	372.55	68.895	232.41-288.72
1939-89	Membrane receptor_GPCR_other	Target median	23	3	400.32	381.46	393.88	432.66	308.38	451.92	53.66	378.39-422.25
1939-89	Membrane receptor_GPCR_other	[Drug - target median]	23	0	-139.76	-197.34	-127.11	-84.415	-244.32	-41.1	60.443	-164.46--115.06
1939-89	Membrane receptor_GPCR_peptide	Drug	31	5	371.29	299.37	327.38	353.44	247.34	357.45	163.52	313.72-428.85
1939-89	Membrane receptor_GPCR_peptide	Target median	31	7	450.77	441.44	443.98	443.98	440.59	443.98	27.423	441.11-460.42
1939-89	Membrane receptor_GPCR_peptide	[Drug - target median]	31	5	-79.482	-144.12	-116.6	-93.195	-196.64	-83.14	139.09	-128.45--30.519
1939-89	Other	Drug	7	1	381.04	286.92	335.88	383.55	180.25	399.44	201.61	231.69-530.4
1939-89	Other	Target median	7	1	341.75	338.41	366.38	376.46	338.41	391.88	63.839	294.46-389.05
1939-89	Other	[Drug - target median]	7	1	39.291	-49.625	-24.02	16	-67.01	22.98	176.87	-91.735-170.32
1939-89	Transcription factor_NHR	Drug	46	2	372.11	298.43	375.48	416.95	214.65	494.53	89.947	346.12-398.11
1939-89	Transcription factor_NHR	Target median	46	0	418.23	389.48	441.5	441.5	344.48	481.01	37.721	407.33-429.13
1939-89	Transcription factor_NHR	[Drug - target median]	46	3	-46.113	-89.035	-58.52	-6.95	-207.19	68.11	90.029	-72.13--20.096
1939-89	Transporter	Drug	35	4	307.93	241.15	277.41	312.67	135.21	404.49	134.68	263.31-352.55
1939-89	Transporter	Target median	35	5	334.21	314.4	319.41	336.48	314.4	336.48	32.093	323.57-344.84
1939-89	Transporter	[Drug - target median]	35	5	-26.273	-74.497	-48.01	-23.805	-98.15	35.39	109.66	-62.603-10.056
1990-2009	Enzyme_Hydrolase	Drug	19	0	324.39	254.33	287.36	428.19	198.27	475.18	96.054	281.2-367.58
1990-2009	Enzyme_Hydrolase	Target median	19	0	363.06	341.86	341.86	394.52	325.43	418.54	31.004	349.12-377.01
1990-2009	Enzyme_Hydrolase	[Drug - target median]	19	0	-38.674	-134.93	-41.07	46.03	-220.27	133.32	112.39	-89.211-11.864
1990-2009	Enzyme_Kinase	Drug	51	2	459.53	437.53	464.83	488.02	393.44	529.53	44.861	447.21-471.84
1990-2009	Enzyme_Kinase	Target median	51	5	448.21	437.43	447.2	456.68	424.52	478.35	22.982	441.9-454.51
1990-2009	Enzyme_Kinase	[Drug - target median]	51	1	11.32	-22.673	16.13	34.616	-62.15	105.01	44.63	-0.92859-23.569
1990-2009	Enzyme_Other	Drug	12	2	418.1	294.89	339.37	406.93	223.28	421.45	214.06	296.98-539.21
1990-2009	Enzyme_Other	Target median	12	0	392.28	318.2	376.43	442.27	318.2	513.6	72.076	351.5-433.06
1990-2009	Enzyme_Other	[Drug - target median]	12	2	25.82	-50.92	-28.86	-6.865	-94.92	7.09	153.14	-60.827-112.47
1990-2009	Enzyme_Oxidoreductase	Drug	32	1	334.14	288.45	320.34	376.96	171.24	481.55	84.096	305-363.28
1990-2009	Enzyme_Oxidoreductase	Target median	32	8	388.67	357.42	381.38	392.4	310.43	401.38	53.943	369.97-407.36
1990-2009	Enzyme_Oxidoreductase	[Drug - target median]	32	0	-54.524	-90.835	-53.005	-21.755	-175.1	78.11	61.837	-75.949--33.098
1990-2009	Enzyme_Phosphodiesterase	Drug	4	0	402.17	322.75	432	481.6	256.09	488.61	106.8	297.51-506.84
1990-2009	Enzyme_Phosphodiesterase	Target median	4	0	415.04	399.18	430.9	430.9	367.45	430.9	31.723	383.95-446.13
1990-2009	Enzyme_Phosphodiesterase	[Drug - target median]	4	0	-12.864	-76.427	1.1	50.7	-111.36	57.71	78.953	-90.238-64.511
1990-2009	Enzyme_Protease	Drug	19	0	518.86	415.23	508.65	625.06	303.41	720.96	132.93	459.09-578.63
1990-2009	Enzyme_Protease	Target median	19	0	515.39	397.61	593.2	606.72	386.42	606.72	101.98	469.54-561.24
1990-2009	Enzyme_Protease	[Drug - target median]	19	0	3.4703	-57.802	12.86	40.192	-101.08	126.72	67.427	-26.848-33.789
1990-2009	Enzyme_Transferase	Drug	9	0	309.55	245.59	283.11	348.47	219.28	458.5	83.505	254.99-364.11
1990-2009	Enzyme_Transferase	Target median	9	0	370.57	342.57	342.57	412.08	342.57	455.52	43.829	341.93-399.2
1990-2009	Enzyme_Transferase	[Drug - target median]	9	1	-61.017	-96.978	-59.46	-11.48	-107.5	46.42	82.501	-114.92--7.1165
1990-2009	Ion channel	Drug	25	3	304.81	256.1	293.37	330.46	156.27	441.58	99.112	265.95-343.66
1990-2009	Ion channel	Target median	25	0	394.29	356.47	399.54	444.77	242.84	467.42	62.778	369.68-418.9
1990-2009	Ion channel	[Drug - target median]	25	0	-89.488	-182.47	-73.44	-27.979	-298.83	96.1	101.46	-129.26--49.717
1990-2009	Membrane receptor_GPCR_amine	Drug	107	0	344.92	270.11	344.41	410.49	197.23	501.67	79.646	329.83-360.02
1990-2009	Membrane receptor_GPCR_amine	Target median	107	1	406.35	376.54	414.53	428.58	329.44	484.08	42.188	398.35-414.34
1990-2009	Membrane receptor_GPCR_amine	[Drug - target median]	107	0	-61.423	-120.65	-62.515	-0.03	-254.34	115.42	78.296	-76.259--46.588
1990-2009	Membrane receptor_GPCR_other	Drug	17	0	374.67	259.35	360.49	459.8	243.31	586.2	113.98	320.48-428.85
1990-2009	Membrane receptor_GPCR_other	Target median	17	0	405.46	305.64	438.94	442.47	273.38	558.07	98.733	358.52-452.39
1990-2009	Membrane receptor_GPCR_other	[Drug - target median]	17	0	-30.792	-67.942	-30.07	11.834	-84.57	28.13	40.157	-49.882--11.703
1990-2009	Membrane receptor_GPCR_peptide	Drug	27	1	447.32	371.32	435.53	501.75	221.34	551.63	140.65	394.26-500.37
1990-2009	Membrane receptor_GPCR_peptide	Target median	27	0	503.36	443.98	514.58	538.93	440.59	571.69	50.952	484.14-522.58
1990-2009	Membrane receptor_GPCR_peptide	[Drug - target median]	27	1	-56.044	-134.93	-70.72	-19.675	-222.64	43.03	126.48	-103.75--8.3373
1990-2009	Other	Drug	20	0	473.82	264.32	542.72	542.72	171.24	853.92	201.12	385.67-561.96
1990-2009	Other	Target median	20	2	382.13	376.46	388.96	399.48	363.87	403.52	46.843	361.6-402.66
1990-2009	Other	[Drug - target median]	20	0	91.689	-126.1	143.24	163.29	-216.25	477.46	199.59	4.2132-179.17
1990-2009	Transcription factor_NHR	Drug	36	2	397.72	333.63	412.53	430.46	296.41	538.58	82.049	370.92-424.52
1990-2009	Transcription factor_NHR	Target median	36	0	399.1	372.32	388.51	441.5	344.48	478.55	40.952	385.72-412.48
1990-2009	Transcription factor_NHR	[Drug - target median]	36	1	-1.3781	-72.935	-19.548	53.27	-138.77	212.31	91.58	-31.294-28.538
1990-2009	Transporter	Drug	24	2	305.61	268.37	279.86	324.4	233.31	383.38	62.43	280.63-330.59
1990-2009	Transporter	Target median	24	0	324.64	314.4	319.41	336.48	314.4	336.48	10.364	320.5-328.79
1990-2009	Transporter	[Drug - target median]	24	3	-19.03	-57.83	-38.025	-9.595	-90.13	52.48	62.647	-44.094-6.0337
2010-2020	Enzyme_Hydrolase	Drug	5	0	334.87	302.13	328.41	371.73	223.3	447.89	79.744	264.97-404.77
2010-2020	Enzyme_Hydrolase	Target median	5	0	360.63	335.91	358.44	387.64	325.43	394.52	29.385	334.87-386.38
2010-2020	Enzyme_Hydrolase	[Drug - target median]	5	1	-25.757	-51.881	-10.99	17.872	-12.1	62.55	86.876	-101.91-50.393
2010-2020	Enzyme_Kinase	Drug	116	2	478.85	446.56	475.36	539.64	312.38	615.4	72.988	465.56-492.13
2010-2020	Enzyme_Kinase	Target	116	2	456.39	436.42	450.22	474.55	400.45	531.59	29.684	450.99-461.79
2010-2020	Enzyme_Kinase	[Drug - target median]	116	0	22.454	-28.355	29.33	77.327	-124.16	159.16	69.724	9.7656-35.143
2010-2020	Enzyme_Oxidoreductase	Drug	6	0	362.33	244.26	286.28	546.94	227.24	582.97	159.4	234.78-489.87
2010-2020	Enzyme_Oxidoreductase	Target median	6	0	387.83	310.43	375.41	460.57	305.55	499.62	78.648	324.9-450.76
2010-2020	Enzyme_Oxidoreductase	[Drug - target median]	6	0	-25.503	-99.235	-43.195	47.32	-137.12	122.4	98.15	-104.04-53.033
2010-2020	Enzyme_Phosphodiesterase	Drug	7	0	431.12	403.21	403.21	460.51	403.21	483.96	35.673	404.69-457.54
2010-2020	Enzyme_Phosphodiesterase	Target median	7	0	413.8	396.02	405.49	426.42	392.86	455.98	22.684	396.99-430.6
2010-2020	Enzyme_Phosphodiesterase	[Drug - target median]	7	0	17.321	-7.8975	10.345	54.53	-52.775	67.645	43.419	-14.844-49.486
2010-2020	Enzyme_Protease	Drug	19	0	515.56	399.19	459.51	698.24	339.4	766.92	154.6	446.05-585.08
2010-2020	Enzyme_Protease	Target median	19	0	527.44	386.42	499.93	723.35	386.42	770.91	155.5	457.51-597.36
2010-2020	Enzyme_Protease	[Drug - target median]	19	1	-11.873	-37.572	-20	26.19	-64.04	86.13	60.897	-39.255-15.51
2010-2020	Enzyme_Transferase	Drug	14	0	396.9	323.37	409.99	434.47	320.4	480.53	57.287	366.89-426.91
2010-2020	Enzyme_Transferase	Target median	14	0	388.35	340.38	366.47	432.49	340.38	455.52	43.926	365.34-411.36
2010-2020	Enzyme_Transferase	[Drug - target median]	14	0	8.5539	-22.775	6.67	42.325	-50.975	94.09	44.918	-14.975-32.083
2010-2020	Ion channel	Drug	6	1	475.14	318.5	422.46	520.5	298.46	520.5	209.69	307.35-642.93
2010-2020	Ion channel	Target median	6	1	450.62	356.47	417.37	417.37	308.3	417.37	170.57	314.13-587.1
2010-2020	Ion channel	[Drug - target median]	6	0	24.52	-24.87	22.62	81.63	-58.01	103.13	61.656	-24.815-73.855
2010-2020	Membrane receptor_GPCR_amine	Drug	15	0	358.37	268.32	392.5	428.3	195.69	484.66	88.943	313.35-403.38
2010-2020	Membrane receptor_GPCR_amine	Target median	15	0	400.73	365.48	414.53	422.8	329.44	484.08	46.79	377.05-424.41
2010-2020	Membrane receptor_GPCR_amine	[Drug - target median]	15	0	-42.367	-81.173	-66.08	6.1475	-169.76	92.13	66.945	-76.246--8.4878
2010-2020	Membrane receptor_GPCR_other	Drug	9	1	465.61	386.7	404.47	518.09	314.47	522.58	136.37	376.52-554.7
2010-2020	Membrane receptor_GPCR_other	Target median	9	2	463.42	433.97	439.05	486.98	380.45	457.6	66.652	419.88-506.97
2010-2020	Membrane receptor_GPCR_other	[Drug - target median]	9	0	2.1856	-56.932	-29.5	63.846	-127.11	201.26	98.42	-62.115-66.486
2010-2020	Membrane receptor_GPCR_peptide	Drug	14	0	527.93	474.57	517.53	588.28	421.31	631.6	68.736	491.92-563.93
2010-2020	Membrane receptor_GPCR_peptide	Target median	14	0	494.6	434.89	511.59	525.65	430.43	559.66	50.678	468.05-521.14
2010-2020	Membrane receptor_GPCR_peptide	[Drug - target median]	14	0	33.328	-13.58	35.557	71.94	-51.08	125.68	49.31	7.4979-59.159
2010-2020	Other	Drug	26	1	354.45	318.35	333.89	349.43	308.12	349.43	111.24	311.69-397.2
2010-2020	Other	Target median	26	2	397.98	375.47	388.94	399.48	363.87	403.52	83.267	365.97-429.99
2010-2020	Other	[Drug - target median]	26	1	-43.535	-69.09	-49.565	-32.96	-85.17	16.6	38.394	-58.293--28.776
2010-2020	Transcription factor_NHR	Drug	4	0	463.27	442.54	470.94	484	420.63	490.56	30.359	433.52-493.02
2010-2020	Transcription factor_NHR	Target median	4	0	406.56	348.84	403.18	464.28	348.84	471.04	66.878	341.02-472.1
2010-2020	Transcription factor_NHR	[Drug - target median]	4	0	56.706	-8.6875	74.315	122.1	-50.405	128.6	83.007	-24.641-138.05
2010-2020	Transporter	Drug	11	0	379.01	320.45	404.46	440.38	246.35	450.92	77.873	332.99-425.03
2010-2020	Transporter	Target median	11	0	401.08	336.48	448.94	448.94	314.4	448.94	58.156	366.71-435.45
2010-2020	Transporter	[Drug - target median]	11	1	-22.07	-57.995	-38.02	0.38	-90.13	9.9	52.57	-53.137-8.9966

Approval period	Therapeutic use	Group	Total Count	Outlier Count	Mean Value	1st Quartile	Median	3rd Quartile	Lower Adjacent Limit	Upper Adjacent Limit	Standard Deviation	Confidence Interval (95%)
1939-1989	Cancer	Drug	11	0	445.23	322.18	444.49	521.26	232.28	810.99	161.2	349.97-540.49
1939-1989	Cancer	Target median	11	0	374.95	351.24	382.14	397.36	318.2	416.47	35.046	354.24-395.66
1939-1989	Cancer	[Drug - target median]	11	1	70.284	-21.7	47.13	133.63	-85.92	172.09	145.92	-15.952-156.52
1939-1989	Cardiovascular	Drug	46	4	322.54	217.29	279.83	348.4	131.18	504.64	166.14	274.53-370.55
1939-1989	Cardiovascular	Target median	46	2	420.4	393.53	437.2	469.64	310.43	571.69	71.288	399.8-441.01
1939-1989	Cardiovascular	[Drug - target median]	46	5	-97.861	-177.26	-139.19	-49.21	-357.42	70.21	159.29	-143.89--51.827
1939-1989	Central nervous system	Drug	74	6	305.19	263.38	309.05	341.43	147.2	437.53	88.798	284.95-325.42
1939-1989	Central nervous system	Target median	74	0	378.39	319.41	390.17	423.56	242.84	467.42	56.038	365.62-391.16
1939-1989	Central nervous system	[Drug - target median]	74	3	-73.207	-116.6	-70.77	-39.06	-231.15	41.05	79.049	-91.218--55.196
1939-1989	Gastrointestinal & metabolism	Drug	18	2	354.25	309.49	338.95	373.88	252.35	425.92	76.479	318.92-389.58
1939-1989	Gastrointestinal & metabolism	Target median	18	0	401.34	354.51	401.55	441.58	314.4	471.04	41.786	382.04-420.64
1939-1989	Gastrointestinal & metabolism	[Drug - target median]	18	1	-47.092	-87.14	-49.36	-3.1	-160.57	33.07	77.72	-82.996--11.187
1939-1989	Genito urinary	Drug	16	0	324.9	280.39	325.46	355.99	266.34	416.95	48.342	301.21-348.59
1939-1989	Genito urinary	Target median	16	0	386.09	348.84	387.34	394.47	333.19	453.61	36.347	368.28-403.9
1939-1989	Genito urinary	[Drug - target median]	16	0	-61.189	-108.54	-66.92	-23.715	-128.13	68.11	56.05	-88.654--33.725
1939-1989	Infection	Drug	5	0	357.59	250.97	335.88	460.13	248.72	507.18	115.6	256.26-458.92
1939-1989	Infection	Target median	5	0	365.4	321.86	341.86	406.29	321.86	449.53	55.055	317.14-413.66
1939-1989	Infection	[Drug - target median]	5	0	-7.811	-70.89	-56	37.511	-73.14	165.32	100.57	-95.962-80.34
1939-1989	Multiple/other	Drug	32	2	307.46	219.26	279.89	361.46	111.15	416.65	162.21	251.26-363.66
1939-1989	Multiple/other	Target median	32	0	399.35	357.42	390.39	441.5	319.41	530.46	48.009	382.72-415.99
1939-1989	Multiple/other	[Drug - target median]	32	2	-91.892	-158.2	-97.34	-55.15	-286.4	0.37	142.59	-141.3--42.486
1939-1989	Musculo-skeletal	Drug	15	0	291.46	249.81	269.39	332.41	152.11	424.09	73.324	254.36-328.57
1939-1989	Musculo-skeletal	Target median	15	1	369.51	357.42	381.38	381.38	357.42	394.39	24.24	357.25-381.78
1939-1989	Musculo-skeletal	[Drug - target median]	15	0	-78.051	-116.43	-100.13	-42.98	-145.12	29.7	58.839	-107.83--48.274
1939-1989	Respiratory	Drug	23	2	284.43	243.32	274.8	314.67	180.17	343.9	64.927	257.89-310.96
1939-1989	Respiratory	Target median	23	0	408.45	386.25	386.25	469.32	338.41	478.59	47.244	389.14-427.76
1939-1989	Respiratory	[Drug - target median]	23	0	-124.02	-195.88	-119.86	-64.944	-267.33	72.33	87.881	-159.94--88.106
1939-1989	Skin & eye	Drug	19	0	337.85	254.92	316.43	436.01	146.21	494.53	103.8	291.18-384.52
1939-1989	Skin & eye	Target median	19	0	417.09	390.24	441.5	441.5	339.95	478.59	41.456	398.45-435.73
1939-1989	Skin & eye	[Drug - target median]	19	0	-79.241	-130.8	-71.27	-5.485	-246.32	53.03	83.903	-116.97--41.513
1990-2009	Cancer	Drug	30	2	460.42	348.49	433.96	529.53	223.28	778.95	163.96	401.75-519.09
1990-2009	Cancer	Target median	30	1	404.06	367.45	399.48	436.42	318.2	510.72	60.575	382.38-425.74
1990-2009	Cancer	[Drug - target median]	30	3	56.358	-31.62	1.285	105.01	-135.16	212.31	151.41	2.1761-110.54
1990-2009	Cardiovascular	Drug	37	8	422.96	402.45	428.54	456.07	360.49	514.63	79.478	397.35-448.57
1990-2009	Cardiovascular	Target median	37	1	469.64	430.66	478.59	515.85	373.45	605.83	76.917	444.85-494.42
1990-2009	Cardiovascular	[Drug - target median]	37	2	-46.681	-72.679	-54.06	9.2338	-148.77	96.1	77.066	-71.513--21.848
1990-2009	Central nervous system	Drug	57	1	295.58	250.33	277.41	336.44	171.24	448.39	72.631	276.72-314.43
1990-2009	Central nervous system	Target median	57	0	376.25	336.48	392.53	417.8	242.84	467.42	52.765	362.55-389.95
1990-2009	Central nervous system	[Drug - target median]	57	0	-80.67	-132.44	-73.1	-27.07	-222.64	55.49	72.75	-99.557--61.784
1990-2009	Gastrointestinal & metabolism	Drug	23	0	349.38	294.87	356.44	401.65	207.23	534.43	81.464	316.09-382.67
1990-2009	Gastrointestinal & metabolism	Target median	23	0	405.48	356.47	386.42	452.64	316.89	523.66	60.088	380.93-430.04
1990-2009	Gastrointestinal & metabolism	[Drug - target median]	23	1	-56.103	-90.247	-62.11	3.2212	-151.2	66.49	71.589	-85.361--26.845
1990-2009	Genito urinary	Drug	18	1	442.79	372.55	411.04	473.59	296.41	495.54	148.47	374.2-511.38
1990-2009	Genito urinary	Target median	18	0	413.19	382.14	397.92	451.57	344.48	530.46	45.925	391.98-434.41
1990-2009	Genito urinary	[Drug - target median]	18	1	29.595	-43.05	1.725	47.46	-98.06	129.11	125.65	-28.454-87.644
1990-2009	Infection	Drug	19	0	492.19	388.18	475.18	602.3	266.3	720.96	138.15	430.07-554.31
1990-2009	Infection	Target median	19	0	465.81	341.86	385.34	606.72	339.4	606.72	131.48	406.68-524.93
1990-2009	Infection	[Drug - target median]	19	0	26.388	-36.207	22.09	110.22	-101.08	133.32	76.766	-8.1297-60.907
1990-2009	Multiple/other	Drug	10	2	457.67	320.34	404.46	476.57	156.27	476.57	230.45	314.83-600.51
1990-2009	Multiple/other	Target median	10	0	443.32	381.38	441.29	513.6	370.34	519.67	63.303	404.08-482.56
1990-2009	Multiple/other	[Drug - target median]	10	2	14.35	-84.57	-57.725	35.98	-214.53	35.98	188.09	-102.23-130.93
1990-2009	Musculo-skeletal	Drug	18	0	290.57	254.29	293.53	316.38	206.28	358.85	40.004	272.09-309.05
1990-2009	Musculo-skeletal	Target median	18	1	361.83	342.57	357.42	381.38	297.23	381.38	33.284	346.45-377.21
1990-2009	Musculo-skeletal	[Drug - target median]	18	0	-71.263	-94.02	-68.75	-29.97	-154.41	19.15	45.042	-92.071--50.454
1990-2009	Respiratory	Drug	20	0	416.64	368.18	415.77	491.04	236.3	586.2	96.087	374.53-458.75
1990-2009	Respiratory	Target median	20	0	439.6	386.25	438.94	478.59	383.42	558.07	61.547	412.63-466.58
1990-2009	Respiratory	[Drug - target median]	20	1	-22.963	-75.99	-2.175	28.925	-147.13	115.42	85.851	-60.589-14.663
1990-2009	Skin & eye	Drug	11	0	337.51	290.2	324.45	405.28	240.31	458.47	69.805	296.25-378.76
1990-2009	Skin & eye	Target median	11	1	383.07	368.89	381.38	422.67	355.39	467.42	64.012	345.24-420.9
1990-2009	Skin & eye	[Drug - target median]	11	0	-45.564	-79.52	-47.21	19.09	-203.05	64.87	76.365	-90.693--0.43471
2010-2020	Cancer	Drug	61	2	469.19	420.44	469.95	526.13	306.37	615.4	92.492	445.98-492.4
2010-2020	Cancer	Target median	61	12	444.62	428.24	446.44	469.52	366.47	507.48	67.814	427.61-461.64
2010-2020	Cancer	[Drug - target median]	61	0	24.563	-29.89	33.53	81.768	-124.16	159.16	67.857	7.5341-41.592
2010-2020	Cardiovascular	Drug	12	1	517.03	464.98	491.58	535.33	435.89	588.28	92.154	464.89-569.17
2010-2020	Cardiovascular	Target median	12	2	496.84	468.04	499.93	507.26	427.47	514.58	45.368	471.17-522.51
2010-2020	Cardiovascular	[Drug - target median]	12	1	20.19	-44.22	23.53	52.862	-64.04	73.7	74.569	-22.002-62.381
2010-2020	Central nervous system	Drug	13	0	363.79	301.38	318.5	430.95	259.14	534.57	81.32	319.59-408
2010-2020	Central nervous system	Target median	13	0	385.65	329.97	380.45	425.52	291.52	554.72	72.097	346.46-424.84
2010-2020	Central nervous system	[Drug - target median]	13	0	-21.856	-45.94	-8.08	11.8	-127.11	26.6	45.818	-46.763-3.0509
2010-2020	Gastrointestinal & metabolism	Drug	22	3	421.01	383.46	406.71	450.92	339.4	546.94	83.084	386.29-455.73
2010-2020	Gastrointestinal & metabolism	Target median	22	0	431.9	386.42	446.46	448.94	365.45	523.66	46.99	412.27-451.54
2010-2020	Gastrointestinal & metabolism	[Drug - target median]	22	2	-10.894	-47.02	-16.025	15.01	-66.08	86.13	60.877	-36.333-14.545
2010-2020	Genito urinary	Drug	4	0	545.93	464.25	553.8	627.62	444.54	631.6	95.74	452.11-639.76
2010-2020	Genito urinary	Target median	4	0	508.57	457.49	521.87	559.66	430.9	559.66	62.856	446.98-570.17
2010-2020	Genito urinary	[Drug - target median]	4	0	37.36	6.76	58.52	67.96	-39.54	71.94	51.848	-13.451-88.171
2010-2020	Infection	Drug	8	0	600.2	397.11	633.99	758.44	328.41	894.13	215.99	450.53-749.88
2010-2020	Infection	Target median	8	0	607.83	371.89	723.35	770.91	339.4	790.97	206.04	465.05-750.62
2010-2020	Infection	[Drug - target median]	8	1	-7.63	-21.78	-11.545	53.057	-22.61	103.16	91.561	-71.078-55.818
2010-2020	Multiple/other	Drug	11	0	354.6	252.32	380.37	444.39	223.3	516.6	104.68	292.74-416.47
2010-2020	Multiple/other	Target median	11	1	409.7	375.71	429.48	433.97	305.55	456.24	62.888	372.53-446.86
2010-2020	Multiple/other	[Drug - target median]	11	1	-55.092	-92.38	-51.08	-33.752	-171.23	1.45	63.914	-92.862--17.321
2010-2020	Musculo-skeletal	Drug	3	0	324.98	264.79	326.4	384.82	244.26	404.29	80.024	234.43-415.54
2010-2020	Musculo-skeletal	Target median	3	0	395.07	384.63	394.39	405.68	381.38	409.45	14.047	379.18-410.97
2010-2020	Musculo-skeletal	[Drug - target median]	3	0	-70.09	-123.6	-83.05	-13.338	-137.12	9.9	74.362	-154.24-14.058
2010-2020	Respiratory	Drug	7	0	439.2	395.18	428.6	476.6	392.5	520.5	49.363	402.63-475.77
2010-2020	Respiratory	Target median	7	0	424.54	398.74	417.37	446.33	392.53	478.59	31.921	400.89-448.18
2010-2020	Respiratory	[Drug - target median]	7	0	14.662	-45.799	35.04	78.115	-86.09	103.13	71.837	-38.555-67.879
