# Supplementary material for: Target-based evaluation of ‘drug-like’ properties and ligand efficiencies
Source: J Med Chem. Author manuscript; Available in PMC 2021 Jun 11. (PMC7610969; doi:10.1021/acs.jmedchem.1c00416)
Supplement: Supp Fig S5 values LE [file EMS123358-supplement-Supp_Fig_S5_values_LE.pdf]

LE

Approval period	Target class	Group	Total Count	Outlier Count	Mean Value	1st Quartile	Median	3rd Quartile	Lower Adjacent Limit	Upper Adjacent Limit	Standard Deviation	Confidence Interval (95%)
1939-89	Enzyme_Hydrolase	Drug	7	0	0.53071	0.3635	0.622	0.66975	0.253	0.695	0.17984	0.39749-0.66394
1939-89	Enzyme_Hydrolase	Target median	7	1	0.29929	0.29	0.29	0.29	0.29	0.29	0.024568	0.28109-0.31749
1939-89	Enzyme_Hydrolase	[Drug - target median]	7	0	0.23143	0.0255	0.332	0.37975	-0.038	0.405	0.19384	0.087828-0.37503
1939-89	Enzyme_Other	Drug	32	0	0.55713	0.4155	0.635	0.6985	0.139	0.847	0.20757	0.48521-0.62904
1939-89	Enzyme_Other	Target median	32	4	0.365	0.345	0.38925	0.4175	0.25	0.438	0.06778	0.34152-0.38848
1939-89	Enzyme_Other	[Drug - target median]	32	0	0.19213	0.05075	0.226	0.325	-0.108	0.424	0.15684	0.13778-0.24647
1939-89	Enzyme_Oxidoreductase	Drug	46	7	0.50498	0.438	0.4815	0.544	0.326	0.62	0.14835	0.46211-0.54785
1939-89	Enzyme_Oxidoreductase	Target median	46	2	0.32572	0.2915	0.324	0.333	0.2915	0.382	0.034555	0.31573-0.3357
1939-89	Enzyme_Oxidoreductase	[Drug - target median]	46	6	0.17926	0.117	0.17575	0.2235	0.002	0.347	0.13965	0.1389-0.21962
1939-89	Enzyme_Phosphodiesterase	Drug	9	0	0.40422	0.23475	0.464	0.5095	0.206	0.545	0.13938	0.31316-0.49528
1939-89	Enzyme_Phosphodiesterase	Target median	9	1	0.32433	0.312	0.319	0.3335	0.298	0.353	0.02265	0.30954-0.33913
1939-89	Enzyme_Phosphodiesterase	[Drug - target median]	9	0	0.079889	-0.09925	0.166	0.18525	-0.147	0.245	0.15753	-0.023029-0.18281
1939-89	Enzyme_Protease	Drug	7	0	0.46657	0.24075	0.467	0.65425	0.188	0.751	0.22444	0.3003-0.63284
1939-89	Enzyme_Protease	Target median	7	0	0.2995	0.24113	0.323	0.341	0.214	0.341	0.058975	0.25581-0.34319
1939-89	Enzyme_Protease	[Drug - target median]	7	0	0.16707	-0.08638	0.126	0.40725	-0.135	0.454	0.25537	-0.022107-0.35625
1939-89	Ion channel	Drug	24	1	0.46612	0.327	0.501	0.543	0.242	0.666	0.15759	0.40307-0.52918
1939-89	Ion channel	Target median	24	0	0.34469	0.255	0.325	0.4215	0.237	0.5815	0.094098	0.30704-0.38233
1939-89	Ion channel	[Drug - target median]	24	0	0.12144	0.0475	0.10925	0.18975	-0.0655	0.3445	0.10126	0.080923-0.16195
1939-89	Membrane receptor_GPCR_amine	Drug	180	6	0.51221	0.4285	0.503	0.5775	0.237	0.778	0.1284	0.49345-0.53097
1939-89	Membrane receptor_GPCR_amine	Target median	180	2	0.32871	0.314	0.325	0.357	0.252	0.3885	0.045335	0.32209-0.33533
1939-89	Membrane receptor_GPCR_amine	[Drug - target median]	180	5	0.1835	0.1025	0.1845	0.252	-0.0975	0.447	0.13002	0.1645-0.2025
1939-89	Membrane receptor_GPCR_other	Drug	23	3	0.50304	0.4455	0.466	0.536	0.332	0.556	0.11603	0.45562-0.55046
1939-89	Membrane receptor_GPCR_other	Target median	23	3	0.3435	0.311	0.328	0.33725	0.273	0.338	0.068687	0.31543-0.37157
1939-89	Membrane receptor_GPCR_other	[Drug - target median]	23	0	0.15954	0.1255	0.152	0.202	0.014	0.311	0.066717	0.13228-0.18681
1939-89	Membrane receptor_GPCR_peptide	Drug	31	1	0.43219	0.37725	0.449	0.498	0.198	0.621	0.10528	0.39513-0.46926
1939-89	Membrane receptor_GPCR_peptide	Target median	31	7	0.30081	0.304	0.304	0.30775	0.304	0.309	0.011202	0.29686-0.30475
1939-89	Membrane receptor_GPCR_peptide	[Drug - target median]	31	0	0.13139	0.07325	0.149	0.194	-0.088	0.317	0.098514	0.096708-0.16607
1939-89	Other	Drug	7	1	0.405	0.29075	0.413	0.4405	0.153	0.441	0.18839	0.26544-0.54456
1939-89	Other	Target median	7	1	0.378	0.286	0.313	0.417	0.283	0.417	0.13164	0.28048-0.47552
1939-89	Other	[Drug - target median]	7	0	0.027	-0.00875	0.024	0.09925	-0.133	0.13	0.088284	-0.038401-0.092401
1939-89	Transcription factor_NHR	Drug	46	0	0.42122	0.37	0.4105	0.492	0.231	0.644	0.10238	0.39163-0.4508
1939-89	Transcription factor_NHR	Target median	46	12	0.32208	0.321	0.321	0.333	0.3115	0.351	0.031528	0.31296-0.33119
1939-89	Transcription factor_NHR	[Drug - target median]	46	0	0.099141	0.049	0.088	0.163	-0.058	0.304	0.089207	0.073362-0.12492
1939-89	Transporter	Drug	35	6	0.50503	0.46225	0.509	0.566	0.36	0.625	0.15923	0.45228-0.55778
1939-89	Transporter	Target median	35	5	0.40123	0.401	0.404	0.432	0.401	0.432	0.044381	0.38653-0.41593
1939-89	Transporter	[Drug - target median]	35	4	0.1038	0.054	0.096	0.148	-0.063	0.224	0.13686	0.058458-0.14914
1990-2009	Enzyme_Hydrolase	Drug	19	0	0.45137	0.35625	0.447	0.52675	0.254	0.692	0.11727	0.39864-0.5041
1990-2009	Enzyme_Hydrolase	Target median	19	0	0.32305	0.29	0.329	0.355	0.27	0.3615	0.034625	0.30748-0.33862
1990-2009	Enzyme_Hydrolase	[Drug - target median]	19	0	0.12832	0.039	0.126	0.21637	-0.101	0.422	0.12971	0.069993-0.18664
1990-2009	Enzyme_Kinase	Drug	51	0	0.33684	0.31475	0.341	0.3725	0.247	0.412	0.044601	0.3246-0.34908
1990-2009	Enzyme_Kinase	Target median	51	3	0.29443	0.2785	0.294	0.3	0.252	0.315	0.01872	0.28929-0.29957
1990-2009	Enzyme_Kinase	[Drug - target median]	51	2	0.042412	0.0215	0.05	0.074875	-0.042	0.133	0.050455	0.028564-0.056259
1990-2009	Enzyme_Other	Drug	12	0	0.40867	0.285	0.429	0.509	0.194	0.625	0.14762	0.32514-0.49219
1990-2009	Enzyme_Other	Target median	12	0	0.33558	0.238	0.382	0.3925	0.2345	0.403	0.074968	0.29317-0.378
1990-2009	Enzyme_Other	[Drug - target median]	12	0	0.073083	-0.0065	0.0695	0.1235	-0.0405	0.223	0.089816	0.022265-0.1239
1990-2009	Enzyme_Oxidoreductase	Drug	32	1	0.4455	0.372	0.44	0.5165	0.222	0.636	0.11671	0.40506-0.48594
1990-2009	Enzyme_Oxidoreductase	Target median	32	0	0.31702	0.293	0.324	0.329	0.24	0.382	0.029667	0.30674-0.32729
1990-2009	Enzyme_Oxidoreductase	[Drug - target median]	32	0	0.12848	0.0495	0.103	0.2	-0.079	0.403	0.11332	0.089221-0.16775
1990-2009	Enzyme_Phosphodiesterase	Drug	4	0	0.4275	0.356	0.38	0.499	0.344	0.606	0.1206	0.30931-0.54569
1990-2009	Enzyme_Phosphodiesterase	Target median	4	0	0.324	0.323	0.323	0.325	0.323	0.327	0.002	0.32204-0.32596
1990-2009	Enzyme_Phosphodiesterase	[Drug - target median]	4	0	0.1035	0.033	0.057	0.174	0.021	0.279	0.11863	-0.012757-0.21976
1990-2009	Enzyme_Protease	Drug	19	0	0.34258	0.2745	0.327	0.396	0.211	0.504	0.082957	0.30528-0.37988
1990-2009	Enzyme_Protease	Target median	19	0	0.28721	0.253	0.253	0.341	0.223	0.363	0.050564	0.26447-0.30995
1990-2009	Enzyme_Protease	[Drug - target median]	19	0	0.055368	0.00475	0.046	0.09925	-0.012	0.141	0.049675	0.033032-0.077705
1990-2009	Enzyme_Transferase	Drug	9	0	0.50211	0.38225	0.589	0.60925	0.226	0.679	0.1641	0.3949-0.60932
1990-2009	Enzyme_Transferase	Target median	9	0	0.376	0.30775	0.412	0.412	0.301	0.412	0.054062	0.34068-0.41132
1990-2009	Enzyme_Transferase	[Drug - target median]	9	0	0.12611	0.06025	0.177	0.19725	-0.075	0.267	0.11624	0.050168-0.20205
1990-2009	Ion channel	Drug	25	0	0.44852	0.37175	0.448	0.56275	0.228	0.64	0.12337	0.40016-0.49688
1990-2009	Ion channel	Target median	25	0	0.3383	0.27	0.316	0.42413	0.237	0.5815	0.087753	0.3039-0.3727
1990-2009	Ion channel	[Drug - target median]	25	0	0.11022	0.062625	0.13	0.18288	-0.103	0.253	0.096906	0.072233-0.14821
1990-2009	Membrane receptor_GPCR_amine	Drug	107	4	0.46997	0.38325	0.467	0.524	0.279	0.73	0.11164	0.44882-0.49113
1990-2009	Membrane receptor_GPCR_amine	Target median	107	1	0.33796	0.316	0.345	0.358	0.258	0.3885	0.036695	0.331-0.34491
1990-2009	Membrane receptor_GPCR_amine	[Drug - target median]	107	4	0.13201	0.0605	0.127	0.19025	-0.078	0.3775	0.10337	0.11243-0.1516
1990-2009	Membrane receptor_GPCR_other	Drug	17	0	0.49706	0.3265	0.393	0.74625	0.288	0.781	0.20399	0.40009-0.59403
1990-2009	Membrane receptor_GPCR_other	Target median	17	0	0.37326	0.288	0.309	0.472	0.273	0.561	0.10652	0.32263-0.4239
1990-2009	Membrane receptor_GPCR_other	[Drug - target median]	17	0	0.12379	0.012	0.1045	0.22375	-0.016	0.318	0.10992	0.071542-0.17605
1990-2009	Membrane receptor_GPCR_peptide	Drug	27	3	0.37456	0.30925	0.369	0.41275	0.223	0.542	0.11479	0.33126-0.41785
1990-2009	Membrane receptor_GPCR_peptide	Target median	27	0	0.28081	0.26	0.276	0.304	0.231	0.309	0.021394	0.27275-0.28888
1990-2009	Membrane receptor_GPCR_peptide	[Drug - target median]	27	3	0.093741	0.04575	0.088	0.1285	-0.05	0.233	0.10348	0.054707-0.13277
1990-2009	Other	Drug	20	0	0.3632	0.216	0.3275	0.5075	0.13	0.862	0.18915	0.2803-0.4461
1990-2009	Other	Target median	20	1	0.32608	0.29775	0.313	0.318	0.28	0.343	0.076763	0.29243-0.35972
1990-2009	Other	[Drug - target median]	20	1	0.037125	-0.0875	0.0125	0.136	-0.156	0.206	0.1702	-0.037469-0.11172
1990-2009	Transcription factor_NHR	Drug	36	0	0.38111	0.3155	0.374	0.449	0.263	0.56	0.078278	0.35554-0.40668
1990-2009	Transcription factor_NHR	Target median	36	8	0.32465	0.321	0.3315	0.341	0.3115	0.351	0.030494	0.31469-0.33461
1990-2009	Transcription factor_NHR	[Drug - target median]	36	0	0.056458	0.002	0.05	0.1325	-0.081	0.228	0.080825	0.030055-0.082861
1990-2009	Transporter	Drug	24	0	0.46858	0.406	0.4775	0.543	0.267	0.662	0.10414	0.42692-0.51025
1990-2009	Transporter	Target median	24	2	0.40542	0.401	0.4025	0.432	0.401	0.432	0.028828	0.39388-0.41695
1990-2009	Transporter	[Drug - target median]	24	0	0.063167	0.0045	0.0685	0.142	-0.165	0.261	0.10042	0.022989-0.10334
2010-2020	Enzyme_Hydrolase	Drug	5	0	0.4852	0.4245	0.483	0.55175	0.333	0.629	0.10773	0.39077-0.57963
2010-2020	Enzyme_Hydrolase	Target median	5	0	0.3116	0.286	0.306	0.33525	0.274	0.3615	0.033999	0.2818-0.3414
2010-2020	Enzyme_Hydrolase	[Drug - target median]	5	1	0.1736	0.1265	0.1565	0.20812	0.059	0.1645	0.10182	0.084349-0.26285
2010-2020	Enzyme_Kinase	Drug	116	9	0.32822	0.288	0.3215	0.3595	0.192	0.464	0.074174	0.31472-0.34171
2010-2020	Enzyme_Kinase	Target	116	5	0.30085	0.286	0.294	0.3115	0.252	0.347	0.02256	0.29675-0.30496
2010-2020	Enzyme_Kinase	[Drug - target median]	116	5	0.027362	-0.0105	0.0225	0.0605	-0.108	0.151	0.064864	0.015558-0.039166
2010-2020	Enzyme_Oxidoreductase	Drug	6	0	0.43833	0.28	0.463	0.474	0.264	0.686	0.15449	0.31471-0.56195
2010-2020	Enzyme_Oxidoreductase	Target median	6	0	0.33175	0.29	0.33225	0.37	0.265	0.401	0.050187	0.29159-0.37191
2010-2020	Enzyme_Oxidoreductase	[Drug - target median]	6	0	0.10658	0.015	0.11575	0.134	-0.026	0.285	0.10862	0.019667-0.1935
2010-2020	Enzyme_Phosphodiesterase	Drug	7	0	0.41	0.31425	0.476	0.486	0.304	0.491	0.091659	0.3421-0.4779
2010-2020	Enzyme_Phosphodiesterase	Target median	7	1	0.31829	0.316	0.319	0.326	0.316	0.327	0.009304	0.31139-0.32518
2010-2020	Enzyme_Phosphodiesterase	[Drug - target median]	7	0	0.091714	-0.00975	0.15	0.17275	-0.012	0.176	0.095227	0.021169-0.16226
2010-2020	Enzyme_Protease	Drug	19	0	0.34695	0.253	0.351	0.42575	0.232	0.453	0.083494	0.3094-0.38449
2010-2020	Enzyme_Protease	Target median	19	0	0.28284	0.191	0.285	0.363	0.173	0.363	0.080716	0.24655-0.31914
2010-2020	Enzyme_Protease	[Drug - target median]	19	2	0.064105	0.05075	0.067	0.08625	0.042	0.123	0.035245	0.048257-0.079953
2010-2020	Enzyme_Transferase	Drug	14	0	0.39643	0.364	0.3765	0.442	0.351	0.457	0.03858	0.37622-0.41664
2010-2020	Enzyme_Transferase	Target median	14	0	0.34521	0.31	0.368	0.374	0.301	0.374	0.031421	0.32876-0.36167
2010-2020	Enzyme_Transferase	[Drug - target median]	14	0	0.051214	0.036	0.058	0.072	-0.017	0.089	0.031086	0.03493-0.067498
2010-2020	Ion channel	Drug	6	0	0.33267	0.231	0.2895	0.455	0.208	0.523	0.13048	0.22826-0.43707
2010-2020	Ion channel	Target median	6	0	0.31117	0.264	0.264	0.432	0.206	0.437	0.098152	0.23263-0.3897
2010-2020	Ion channel	[Drug - target median]	6	0	0.0215	-0.021	0.0125	0.072	-0.033	0.086	0.048723	-0.017486-0.060486
2010-2020	Membrane receptor_GPCR_amine	Drug	15	0	0.5058	0.40775	0.436	0.605	0.349	0.834	0.13904	0.43544-0.57616
2010-2020	Membrane receptor_GPCR_amine	Target median	15	3	0.3478	0.34525	0.354	0.3655	0.316	0.3885	0.037803	0.32867-0.36693
2010-2020	Membrane receptor_GPCR_amine	[Drug - target median]	15	1	0.158	0.079	0.118	0.21113	0.047	0.3345	0.11865	0.097954-0.21805
2010-2020	Membrane receptor_GPCR_other	Drug	9	0	0.36256	0.30025	0.347	0.43625	0.27	0.494	0.077068	0.3122-0.41291
2010-2020	Membrane receptor_GPCR_other	Target median	9	0	0.30578	0.2695	0.309	0.351	0.244	0.351	0.04428	0.27685-0.33471
2010-2020	Membrane receptor_GPCR_other	[Drug - target median]	9	0	0.056778	0.01275	0.026	0.107	-0.004	0.155	0.059891	0.017649-0.095907
2010-2020	Membrane receptor_GPCR_peptide	Drug	14	0	0.332	0.293	0.3135	0.37	0.274	0.403	0.047712	0.30701-0.35699
2010-2020	Membrane receptor_GPCR_peptide	Target median	14	0	0.28014	0.263	0.2895	0.297	0.231	0.304	0.02169	0.26878-0.2915
2010-2020	Membrane receptor_GPCR_peptide	[Drug - target median]	14	0	0.051857	0.021	0.046	0.097	-0.011	0.11	0.040453	0.030667-0.073048
2010-2020	Other	Drug	26	1	0.41908	0.371	0.445	0.469	0.292	0.497	0.070945	0.39181-0.44635
2010-2020	Other	Target median	26	2	0.31462	0.306	0.3135	0.33	0.2915	0.343	0.025237	0.30491-0.32432
2010-2020	Other	[Drug - target median]	26	0	0.10446	0.062	0.1335	0.153	-0.018	0.172	0.061711	0.08074-0.12818
2010-2020	Transcription factor_NHR	Drug	4	0	0.3175	0.3035	0.3205	0.3315	0.288	0.341	0.021947	0.29599-0.33901
2010-2020	Transcription factor_NHR	Target median	4	0	0.31975	0.2605	0.322	0.379	0.256	0.379	0.068515	0.25261-0.38689
2010-2020	Transcription factor_NHR	[Drug - target median]	4	0	-0.00225	-0.074	0.003	0.0695	-0.091	0.076	0.084172	-0.084739-0.080239
2010-2020	Transporter	Drug	11	0	0.42473	0.38325	0.402	0.47375	0.293	0.561	0.080554	0.37712-0.47233
2010-2020	Transporter	Target median	11	0	0.36555	0.339	0.339	0.401	0.339	0.432	0.035495	0.34457-0.38652
2010-2020	Transporter	[Drug - target median]	11	1	0.059182	0.03775	0.063	0.08825	-0.014	0.154	0.059687	0.023909-0.094455

Approval period	Therapeutic use	Group	Total Count	Outlier Count	Mean Value	1st Quartile	Median	3rd Quartile	Lower Adjacent Limit	Upper Adjacent Limit	Standard Deviation	Confidence Interval (95%)
1939-1989	Cancer	Drug	11	0	0.29618	0.21375	0.296	0.378	0.142	0.459	0.10582	0.23365-0.35872
1939-1989	Cancer	Target median	11	0	0.31018	0.25	0.331	0.37125	0.236	0.382	0.060188	0.27461-0.34575
1939-1989	Cancer	[Drug - target median]	11	0	-0.014	-0.08525	0.003	0.044	-0.133	0.08	0.072154	-0.05664-0.02864
1939-1989	Cardiovascular	Drug	46	0	0.48043	0.356	0.4655	0.614	0.161	0.819	0.17668	0.42938-0.53149
1939-1989	Cardiovascular	Target median	46	2	0.30027	0.258	0.265	0.341	0.211	0.3885	0.076697	0.27811-0.32244
1939-1989	Cardiovascular	[Drug - target median]	46	0	0.18016	0.056	0.18125	0.2655	-0.107	0.454	0.15091	0.13655-0.22377
1939-1989	Central nervous system	Drug	74	6	0.51482	0.448	0.5045	0.555	0.293	0.699	0.13031	0.48513-0.54451
1939-1989	Central nervous system	Target median	74	1	0.35929	0.316	0.346	0.401	0.27	0.463	0.054784	0.34681-0.37177
1939-1989	Central nervous system	[Drug - target median]	74	4	0.15553	0.088	0.1415	0.1965	-0.059	0.347	0.11332	0.12971-0.18135
1939-1989	Gastrointestinal & metabolism	Drug	18	1	0.44706	0.405	0.4645	0.528	0.242	0.578	0.10057	0.40059-0.49352
1939-1989	Gastrointestinal & metabolism	Target median	18	1	0.32067	0.304	0.316	0.355	0.252	0.379	0.049062	0.298-0.34333
1939-1989	Gastrointestinal & metabolism	[Drug - target median]	18	0	0.12639	0.059	0.114	0.216	-0.137	0.276	0.10632	0.07727-0.17551
1939-1989	Genito urinary	Drug	16	0	0.49669	0.446	0.493	0.543	0.333	0.644	0.087048	0.45403-0.53934
1939-1989	Genito urinary	Target median	16	1	0.34191	0.332	0.348	0.368	0.292	0.38	0.031356	0.32654-0.35727
1939-1989	Genito urinary	[Drug - target median]	16	0	0.15478	0.1045	0.16	0.1985	-0.009	0.304	0.090334	0.11052-0.19905
1939-1989	Infection	Drug	5	0	0.415	0.28575	0.413	0.56175	0.192	0.606	0.16826	0.26752-0.56248
1939-1989	Infection	Target median	5	0	0.3617	0.31262	0.355	0.424	0.283	0.424	0.062325	0.30707-0.41633
1939-1989	Infection	[Drug - target median]	5	0	0.0533	-0.06113	0.123	0.143	-0.1305	0.182	0.13174	-0.062176-0.16878
1939-1989	Multiple/other	Drug	32	1	0.52897	0.4105	0.5355	0.592	0.181	0.837	0.16397	0.47216-0.58578
1939-1989	Multiple/other	Target median	32	3	0.31811	0.2915	0.317	0.3225	0.268	0.355	0.037246	0.3052-0.33101
1939-1989	Multiple/other	[Drug - target median]	32	1	0.21086	0.092	0.19275	0.283	-0.088	0.52	0.1564	0.15667-0.26505
1939-1989	Musculo-skeletal	Drug	15	2	0.45627	0.40125	0.446	0.518	0.267	0.532	0.14487	0.38295-0.52958
1939-1989	Musculo-skeletal	Target median	15	1	0.31777	0.2915	0.324	0.324	0.286	0.352	0.028187	0.3035-0.33203
1939-1989	Musculo-skeletal	[Drug - target median]	15	2	0.1385	0.073	0.122	0.21962	-0.019	0.2395	0.1382	0.068562-0.20844
1939-1989	Respiratory	Drug	23	0	0.5233	0.483	0.532	0.57725	0.345	0.627	0.073657	0.4932-0.55341
1939-1989	Respiratory	Target median	23	1	0.31307	0.27075	0.317	0.32	0.261	0.3795	0.040474	0.29652-0.32961
1939-1989	Respiratory	[Drug - target median]	23	4	0.21024	0.20037	0.221	0.2525	0.167	0.31	0.08112	0.17709-0.24339
1939-1989	Skin & eye	Drug	19	0	0.53984	0.37825	0.467	0.64425	0.33	0.904	0.18297	0.45757-0.62212
1939-1989	Skin & eye	Target median	19	4	0.33597	0.321	0.321	0.357	0.29	0.403	0.046705	0.31497-0.35697
1939-1989	Skin & eye	[Drug - target median]	19	0	0.20387	0.05725	0.122	0.35525	0.009	0.547	0.16464	0.12984-0.2779
1990-2009	Cancer	Drug	30	0	0.34827	0.264	0.344	0.412	0.13	0.606	0.12761	0.3026-0.39393
1990-2009	Cancer	Target median	30	0	0.31233	0.286	0.307	0.341	0.223	0.382	0.046187	0.29581-0.32886
1990-2009	Cancer	[Drug - target median]	30	0	0.035933	-0.034	0.0545	0.115	-0.156	0.279	0.10532	-0.001755-0.073622
1990-2009	Cardiovascular	Drug	37	1	0.36973	0.3155	0.369	0.4125	0.222	0.502	0.085182	0.34228-0.39718
1990-2009	Cardiovascular	Target median	37	1	0.29736	0.26	0.287	0.31488	0.237	0.364	0.067695	0.27555-0.31918
1990-2009	Cardiovascular	[Drug - target median]	37	0	0.072365	0.02125	0.079	0.12325	-0.103	0.228	0.072836	0.048896-0.095834
1990-2009	Central nervous system	Drug	57	3	0.53537	0.44125	0.521	0.5875	0.307	0.781	0.13096	0.50137-0.56937
1990-2009	Central nervous system	Target median	57	3	0.36607	0.31825	0.366	0.401	0.27	0.432	0.064067	0.34944-0.3827
1990-2009	Central nervous system	[Drug - target median]	57	3	0.1693	0.08275	0.171	0.21888	-0.009	0.422	0.12509	0.13682-0.20177
1990-2009	Gastrointestinal & metabolism	Drug	23	0	0.42935	0.3575	0.441	0.499	0.271	0.589	0.087072	0.39376-0.46493
1990-2009	Gastrointestinal & metabolism	Target median	23	0	0.34083	0.304	0.329	0.363	0.264	0.437	0.057707	0.31724-0.36441
1990-2009	Gastrointestinal & metabolism	[Drug - target median]	23	0	0.088522	0.04775	0.089	0.13525	-0.044	0.204	0.064217	0.062277-0.11477
1990-2009	Genito urinary	Drug	18	4	0.38256	0.368	0.3785	0.413	0.302	0.42	0.085173	0.34321-0.4219
1990-2009	Genito urinary	Target median	18	1	0.333	0.323	0.332	0.351	0.314	0.358	0.022021	0.32283-0.34317
1990-2009	Genito urinary	[Drug - target median]	18	2	0.049556	0.021	0.044	0.099	-0.049	0.157	0.079731	0.012722-0.086389
1990-2009	Infection	Drug	19	1	0.34689	0.27425	0.322	0.38175	0.251	0.517	0.096579	0.30347-0.39032
1990-2009	Infection	Target median	19	0	0.30324	0.253	0.274	0.355	0.253	0.3615	0.050482	0.28054-0.32594
1990-2009	Infection	[Drug - target median]	19	1	0.043658	-0.00175	0.044	0.07475	-0.101	0.1555	0.076964	0.0090506-0.078265
1990-2009	Multiple/other	Drug	10	0	0.3789	0.305	0.3725	0.464	0.194	0.565	0.12651	0.30049-0.45731
1990-2009	Multiple/other	Target median	10	0	0.2903	0.256	0.29675	0.324	0.2345	0.3345	0.038897	0.26619-0.31441
1990-2009	Multiple/other	[Drug - target median]	10	0	0.0886	0.029	0.08575	0.1295	-0.0405	0.242	0.096289	0.02892-0.14828
1990-2009	Musculo-skeletal	Drug	18	0	0.50889	0.389	0.513	0.603	0.351	0.679	0.11231	0.457-0.56077
1990-2009	Musculo-skeletal	Target median	18	0	0.35344	0.324	0.324	0.412	0.2915	0.412	0.04891	0.33085-0.37604
1990-2009	Musculo-skeletal	[Drug - target median]	18	0	0.15544	0.083	0.144	0.222	0.027	0.3445	0.093421	0.11229-0.1986
1990-2009	Respiratory	Drug	20	1	0.3944	0.3545	0.3865	0.4275	0.279	0.526	0.078061	0.36019-0.42861
1990-2009	Respiratory	Target median	20	0	0.3046	0.288	0.317	0.317	0.261	0.357	0.024282	0.29396-0.31524
1990-2009	Respiratory	[Drug - target median]	20	0	0.0898	0.0355	0.0675	0.1545	-0.038	0.211	0.075296	0.0568-0.1228
1990-2009	Skin & eye	Drug	11	0	0.48918	0.39025	0.494	0.554	0.356	0.693	0.10872	0.42493-0.55343
1990-2009	Skin & eye	Target median	11	0	0.34455	0.283	0.327	0.38725	0.268	0.529	0.076915	0.29909-0.39
1990-2009	Skin & eye	[Drug - target median]	11	0	0.14464	0.0905	0.164	0.18875	0.016	0.244	0.0699	0.10333-0.18594
2010-2020	Cancer	Drug	61	4	0.35775	0.313	0.351	0.3815	0.251	0.471	0.071658	0.33977-0.37574
2010-2020	Cancer	Target median	61	4	0.30864	0.2895	0.298	0.325	0.273	0.374	0.03283	0.3004-0.31688
2010-2020	Cancer	[Drug - target median]	61	4	0.049115	0.01075	0.042	0.0745	-0.057	0.163	0.063817	0.0331-0.06513
2010-2020	Cardiovascular	Drug	12	0	0.3255	0.2945	0.3145	0.3585	0.268	0.408	0.04674	0.29905-0.35195
2010-2020	Cardiovascular	Target median	12	0	0.27658	0.2605	0.285	0.286	0.244	0.313	0.020389	0.26505-0.28812
2010-2020	Cardiovascular	[Drug - target median]	12	0	0.048917	0.0185	0.048	0.0835	-0.022	0.123	0.04667	0.022511-0.075323
2010-2020	Central nervous system	Drug	13	1	0.45838	0.38725	0.444	0.5055	0.326	0.587	0.10836	0.39948-0.51729
2010-2020	Central nervous system	Target median	13	0	0.3555	0.3285	0.347	0.38675	0.273	0.432	0.046615	0.33016-0.38084
2010-2020	Central nervous system	[Drug - target median]	13	1	0.10288	0.041	0.097	0.1465	0.013	0.177	0.087381	0.055384-0.15039
2010-2020	Gastrointestinal & metabolism	Drug	22	1	0.40718	0.358	0.4065	0.433	0.28	0.453	0.1085	0.36184-0.45252
2010-2020	Gastrointestinal & metabolism	Target median	22	0	0.33586	0.31	0.339	0.363	0.256	0.368	0.033393	0.32191-0.34982
2010-2020	Gastrointestinal & metabolism	[Drug - target median]	22	3	0.071318	0.043	0.062	0.077	0.003	0.09	0.093041	0.032439-0.1102
2010-2020	Genito urinary	Drug	4	0	0.31475	0.2895	0.308	0.34	0.275	0.368	0.038896	0.27663-0.35287
2010-2020	Genito urinary	Target median	4	0	0.279	0.255	0.269	0.303	0.255	0.323	0.032166	0.24748-0.31052
2010-2020	Genito urinary	[Drug - target median]	4	0	0.03575	0.0045	0.0345	0.067	-0.011	0.085	0.040966	-0.0043971-0.075897
2010-2020	Infection	Drug	8	0	0.31887	0.2425	0.269	0.394	0.214	0.526	0.11349	0.24023-0.39752
2010-2020	Infection	Target median	8	0	0.23769	0.182	0.2115	0.29	0.173	0.3615	0.069771	0.18934-0.28604
2010-2020	Infection	[Drug - target median]	8	0	0.081187	0.0515	0.067	0.133	-0.018	0.1645	0.059711	0.03981-0.12257
2010-2020	Multiple/other	Drug	11	0	0.47373	0.38	0.468	0.54975	0.284	0.686	0.1211	0.40216-0.5453
2010-2020	Multiple/other	Target median	11	2	0.34532	0.32625	0.351	0.36075	0.29	0.401	0.04644	0.31787-0.37276
2010-2020	Multiple/other	[Drug - target median]	11	0	0.12841	0.04525	0.1275	0.17525	-0.004	0.339	0.10786	0.064669-0.19215
2010-2020	Musculo-skeletal	Drug	3	0	0.424	0.33425	0.458	0.50525	0.293	0.521	0.11774	0.29076-0.55724
2010-2020	Musculo-skeletal	Target median	3	0	0.33533	0.3255	0.33	0.3465	0.324	0.352	0.014742	0.31865-0.35202
2010-2020	Musculo-skeletal	[Drug - target median]	3	0	0.088667	-0.01075	0.134	0.17675	-0.059	0.191	0.13102	-0.059597-0.23693
2010-2020	Respiratory	Drug	7	0	0.35614	0.26625	0.349	0.42875	0.231	0.491	0.096669	0.28453-0.42776
2010-2020	Respiratory	Target median	7	0	0.298	0.264	0.264	0.3475	0.261	0.357	0.045166	0.26454-0.33146
2010-2020	Respiratory	[Drug - target median]	7	0	0.058143	-0.00325	0.072	0.08575	-0.033	0.172	0.069665	0.0065347-0.10975
