# Supplementary material for: Target-based evaluation of ‘drug-like’ properties and ligand efficiencies
Source: J Med Chem. Author manuscript; Available in PMC 2021 Jun 11. (PMC7610969; doi:10.1021/acs.jmedchem.1c00416)
Supplement: Supp Fig S9 values Fsp3 [file EMS123358-supplement-Supp_Fig_S9_values_Fsp3.pdf]

| Fsp3 | Approval period | Group | Total Count | Outlier Count | Mean Value | 1st Quartile | Median | 3rd Quartile | Lower Adjacent Limit | Upper Adjacent Limit | Standard Deviation | Confidence Interval (95%) |
|---|---|---|---|---|---|---|---|---|---|---|---|---|
| Carboaromatic drugs | 1939-1989 | Drug | 151 | 0 | 0.39563 | 0.26 | 0.38 | 0.5475 | 0 | 0.79 | 0.17964 | 0.36698-0.42428 |
| Carboaromatic drugs | 1939-1989 | Target median | 151 | 0 | 0.33937 | 0.25125 | 0.37 | 0.41 | 0.1 | 0.48 | 0.10127 | 0.32322-0.35552 |
| Carboaromatic drugs | 1939-1989 | [Drug - target median] | 151 | 0 | 0.05626 | -0.08 | 0.05 | 0.18 | -0.37 | 0.42 | 0.15904 | 0.030891-0.081625 |
| Carboaromatic drugs | 1990-2009 | Drug | 98 | 1 | 0.43408 | 0.35 | 0.43 | 0.55 | 0.07 | 0.81 | 0.16423 | 0.40156-0.4666 |
| Carboaromatic drugs | 1990-2009 | Target median | 98 | 0 | 0.35413 | 0.29 | 0.39 | 0.41 | 0.16 | 0.59 | 0.0998 | 0.33437-0.37389 |
| Carboaromatic drugs | 1990-2009 | [Drug - target median] | 98 | 1 | 0.07995 | -0.03 | 0.085 | 0.19 | -0.27 | 0.38 | 0.15205 | 0.049846-0.11005 |
| Carboaromatic drugs | 2010-2020 | Drug | 29 | 1 | 0.42793 | 0.3275 | 0.45 | 0.5225 | 0.13 | 0.7 | 0.17043 | 0.3659-0.48996 |
| Carboaromatic drugs | 2010-2020 | Target median | 29 | 2 | 0.36793 | 0.33 | 0.38 | 0.43 | 0.19 | 0.58 | 0.10547 | 0.32954-0.40632 |
| Carboaromatic drugs | 2010-2020 | [Drug - target median] | 29 | 1 | 0.06 | 0.015 | 0.05 | 0.1425 | -0.12 | 0.3 | 0.1188 | 0.01676-0.10324 |
| | | | | | | | | | | | | |
| Heteroaromatic drugs | 1939-1989 | Drug | 65 | 1 | 0.35523 | 0.2425 | 0.35 | 0.4625 | 0 | 0.75 | 0.17797 | 0.31197-0.3985 |
| Heteroaromatic drugs | 1939-1989 | Target median | 65 | 0 | 0.32062 | 0.245 | 0.35 | 0.41 | 0.1 | 0.5 | 0.10279 | 0.29563-0.3456 |
| Heteroaromatic drugs | 1939-1989 | [Drug - target median] | 65 | 1 | 0.03462 | -0.0825 | -0.01 | 0.12 | -0.24 | 0.41 | 0.16401 | -0.0052575-0.074488 |
| Heteroaromatic drugs | 1990-2009 | Drug | 116 | 1 | 0.3381 | 0.26 | 0.32 | 0.435 | 0 | 0.69 | 0.13967 | 0.31269-0.36352 |
| Heteroaromatic drugs | 1990-2009 | Target median | 116 | 0 | 0.31379 | 0.24 | 0.31 | 0.4 | 0.13 | 0.58 | 0.10672 | 0.29437-0.33321 |
| Heteroaromatic drugs | 1990-2009 | [Drug - target median] | 116 | 3 | 0.02431 | -0.07 | 0.02 | 0.11 | -0.28 | 0.35 | 0.13706 | -0.00063157-0.049252 |
| Heteroaromatic drugs | 2010-2020 | Drug | 107 | 2 | 0.32804 | 0.23 | 0.32 | 0.41 | 0 | 0.63 | 0.1435 | 0.30085-0.35523 |
| Heteroaromatic drugs | 2010-2020 | Target median | 107 | 3 | 0.30589 | 0.215 | 0.3 | 0.36 | 0.09 | 0.48 | 0.10294 | 0.28638-0.32539 |
| Heteroaromatic drugs | 2010-2020 | [Drug - target median] | 107 | 2 | 0.02215 | -0.0675 | 0.01 | 0.09 | -0.3 | 0.31 | 0.12261 | -0.0010829-0.045382 |
| | | | | | | | | | | | | |
| Aliphatic drugs | 1939-1989 | Drug | 43 | 1 | 0.80349 | 0.73 | 0.79 | 0.8575 | 0.68 | 1 | 0.10843 | 0.77108-0.8359 |
| Aliphatic drugs | 1939-1989 | Target median | 43 | 7 | 0.37349 | 0.315 | 0.33 | 0.375 | 0.25 | 0.46 | 0.14171 | 0.33113-0.41584 |
| Aliphatic drugs | 1939-1989 | [Drug - target median] | 43 | 6 | 0.43 | 0.3825 | 0.42 | 0.5 | 0.21 | 0.62 | 0.15271 | 0.38436-0.47564 |
| Aliphatic drugs | 1990-2009 | Drug | 29 | 0 | 0.8269 | 0.7175 | 0.83 | 1 | 0.45 | 1 | 0.15712 | 0.76971-0.88408 |
| Aliphatic drugs | 1990-2009 | Target median | 29 | 0 | 0.44621 | 0.27 | 0.44 | 0.57 | 0.2 | 0.76 | 0.17053 | 0.38414-0.50827 |
| Aliphatic drugs | 1990-2009 | [Drug - target median] | 29 | 0 | 0.38069 | 0.235 | 0.41 | 0.505 | -0.06 | 0.8 | 0.20819 | 0.30492-0.45646 |
| Aliphatic drugs | 2010-2020 | Drug | 5 | 0 | 0.828 | 0.755 | 0.81 | 0.9525 | 0.62 | 0.96 | 0.13846 | 0.70664-0.94936 |
| Aliphatic drugs | 2010-2020 | Target median | 5 | 0 | 0.435 | 0.2325 | 0.52 | 0.59875 | 0.18 | 0.64 | 0.20676 | 0.25377-0.61623 |
| Aliphatic drugs | 2010-2020 | [Drug - target median] | 5 | 0 | 0.393 | 0.145 | 0.225 | 0.725 | 0.1 | 0.77 | 0.32054 | 0.11204-0.67396 |
