# Supplementary material for: Target-based evaluation of ‘drug-like’ properties and ligand efficiencies
Source: J Med Chem. Author manuscript; Available in PMC 2021 Jun 11. (PMC7610969; doi:10.1021/acs.jmedchem.1c00416)
Supplement: Supp manual curation approval dates [file EMS123358-supplement-Supp_manual_curation_approval_dates.pdf]

CMPD_PREF_NAME	First approval dates added, not in CHEMBL_v26	Reference (Annual Reports in Medicinal Chemistry Reviews (formerly Annual Reports in Medicinal Chemistry): Vol, page; or other date reference)
AMISULPRIDE	1986	22, 319
AMSACRINE	1987	23, 327
CIPROFIBRATE	1985	21, 326
DEXIBUPROFEN	1994	30, 298
DEXKETOPROFEN	1994	30, 298
FADROZOLE	1995	31, 342
GOSOGLIPTIN	2016	52, 557
IBUDILAST	1989	25, 313
MELAGATRAN	2004	40, 470 (ximelagatran)
MIZOLASTINE	1998	34, 325
MOCLOBEMIDE	1990	26, 305
MOFEZOLAC	1994	30, 304
MOXONIDINE	1991	27, 330
OZAGREL	1988	24, 308
REBOXETINE	1997	33, 342
SERATRODAST	1995	31, 349
SERTINDOLE	1996	32, 318
TOLOXATONE	1984	20, 324
TOLRESTAT	1989	25, 319
VOGLIBOSE	1994	30, 313
AGOMELATINE	2009	ma.europa.eu/en/medicines/human/EPAR/valdoxan
LASOFOXIFENE	2009	ema.europa.eu/en/medicines/human/EPAR/fablyn
PITOLISANT	2016	i/en/medicines/human/EPAR/wakix#authorisation-details-section
LUMIRACOXIB	2005	swers-recommendation-withdraw-marketing-authorisations-lumiracoxib-containing-medicines_en.pdf
TIVOZANIB	2017	/en/medicines/human/EPAR/fotivda#authorisation-details-section
VILDAGLIPTIN	2007	ema.europa.eu/en/medicines/human/EPAR/galvus
ATOSIBAN	2000	ma.europa.eu/en/medicines/human/EPAR/tractocile
TEPOTINIB	2020	s://www.pmda.go.jp/files/000235289.pdf
TIRABRUTINIB	2020	s://www.pmda.go.jp/files/000235289.pdf
NALFURAFINE	2009	ps://www.pmda.go.jp/files/000211074.pdf;https://www.pmda.go.jp/files/000232775.pdf
PEFICITINIB	2019	s://www.pmda.go.jp/files/000235288.pdf
QUIZARTINIB	2019	s://www.pmda.go.jp/files/000235289.pdf
RELUGOLIX	2019	s://www.pmda.go.jp/files/000235288.pdf
VIBEGRON	2018	s://www.pmda.go.jp/files/000235288.pdf
NINAMIVIR OCTANOA	2010	s://www.pmda.go.jp/files/000211260.pdf
ESFLURBIPROFEN	2015	s://www.pmda.go.jp/files/000225702.pdf
IPRAGLIFLOZIN	2014	pmda.go.jp/files/000206796.pdf;PMID: 24668021
TOFOGLIFLOZIN	2014	s://www.pmda.go.jp/files/000232771.pdf
ANAGLIPTIN	2012	s://www.pmda.go.jp/files/000232773.pdf
TENELIGLIPTIN	2012	s://www.pmda.go.jp/files/000232773.pdf
MINODRONIC ACID	2009	s://www.pmda.go.jp/files/000232775.pdf
TAMIBAROTENE	2005	s://www.pmda.go.jp/files/000153119.pdf
BLONANSERIN	2008	s://www.pmda.go.jp/files/000152974.pdf
PEMAFIBRATE	2017	s://www.pmda.go.jp/files/000226672.pdf
TRELAGLIPTIN	2015	s://www.pmda.go.jp/files/000213963.pdf
ISTRADEFYLLINE	2013	s://www.pmda.go.jp/files/000232773.pdf
CAPMATINIB	2020	ta.fda.gov/drugsatfda_docs/label/2020/213591s000lbl.pdf
OSILODROSTAT	2020	ta.fda.gov/drugsatfda_docs/label/2020/212801s000lbl.pdf
OZANIMOD	2020	ta.fda.gov/drugsatfda_docs/label/2020/209899s000lbl.pdf
RIMEGEPANT	2020	ta.fda.gov/drugsatfda_docs/label/2020/212728s000lbl.pdf
SELUMETINIB	2020	ta.fda.gov/drugsatfda_docs/label/2020/213756s000lbl.pdf
ENTRECTINIB	2019	ta.fda.gov/drugsatfda_docs/label/2019/212725s000lbl.pdf
FEDRATINIB	2019	ta.fda.gov/drugsatfda_docs/label/2019/212327s000lbl.pdf
UPADACITINIB	2019	ta.fda.gov/drugsatfda_docs/label/2019/211675s000lbl.pdf
ZANUBRUTINIB	2019	ta.fda.gov/drugsatfda_docs/label/2019/213217s000lbl.pdf
LUMATEPERONE	2019	ta.fda.gov/drugsatfda_docs/label/2019/209500s000lbl.pdf
OPICAPONE	2020	ta.fda.gov/drugsatfda_docs/label/2020/212489s000lbl.pdf
TRAMETINIB	2013	v/drugsatfda_docs/label/2018/204114s007lbl.pdf;PMID: 23846731
TAT (from telotristat	2017	s000lbl.pdf;https://www.ema.europa.eu/en/medicines/human/EPAR/xermelo#authorisation-details-section
ONE (from abirateron	2011	/scripts/cder/daf/index.cfm?event=overview.process&ApplNo=202379
ETORICOXIB	2002	L/pdfStore.nsf&docid=36577003B3D24322CA2586A0000394DC&agid=(PrintDetailsPublic)&actionid=1
RALTITREXED	1996	B67FCA258676003CAE8D&agid=(PrintDetailsPublic)&actionid=1;https://health-products.canada.ca/dpd-bdpp/info.do?lang=en&code=47482
MOZAVAPTAN	2006	595;https://drugs.ncats.io/drug/17OJ42922Y;https://www.pmda.go.jp/files/000153730.pdf
RUPATADINE	2001	es/cima/pdfs/ipe/81543/IPE_81543.pdf;PMID: 31231589
EVOGLIPTIN	2015	PMID: 26541763
IMIDAPRIL	1993	orts in Medicinal Chemistry, Vol 48, 2013, p 591
ASUNAPREVIR	2014	Vol 54, 2019, p661;https://www.pmda.go.jp/files/000209023.pdf
NAFTOPIDIL	1999	.io/drug/R9PHW59SFN;https://www.asahi-kasei.com/news/2020/e200602.html;PMID: 21753885;PMID: 32727149
EPALRESTAT	1992	ID: 16801576;https://www.sciencedirect.com/topics/agricultural-and-biological-sciences/epalrestat
OLMUTINIB	2016	harm.com/ehanmi/handler/Board-Read?board_id=INFORMATION_ENGLISH_NEWS&seq=90038101&pageNo=0&search_type=
OMARIGLIPTIN	2015	ttps://en.wikipedia.org/wiki/Omarigliptin;https://www.pmda.go.jp/files/000229077.pdf
RAMATROBAN	2000	: 32952595;https://www.sciencedirect.com/topics/medicine-and-dentistry/ramatroban
EBASTINE	1990	nts/portlet_file_entry/4257831/150414_2014_Annual_Report_Baixa.pdf/c426706e-6512-4247-b1ae-5cafd2bf52d3;PMID: 21437146;https://mri.cts-mrp.eu/human/downloads/NL_H_2308_001_PAR.pdf
PRANLUKAST	1995	20of,allergic%20rhinitis%20in%20pediatric%20patients;PMID: 12699401;https://www.genome.jp/dbget-bin/www_bget?D08408+D02732
LOXOPROFEN	1986	rad-ar.or.jp/siori/english/print.cgi?n=1944;https://www.pmda.go.jp/files/000153119.pdf;PMID: 21042022
RILMENIDINE	1987	ivative%2C%20is,for%20general%20practitioners%20in%201990;http://agence-prd.ansm.sante.fr/php/ecodex/extrait.php?specid=67541600
