# Supplementary material for: Target-based evaluation of ‘drug-like’ properties and ligand efficiencies
Source: J Med Chem. Author manuscript; Available in PMC 2021 Jun 11. (PMC7610969; doi:10.1021/acs.jmedchem.1c00416)
Supplement: Supp manual curation_prodrugs [file EMS123358-supplement-Supp_manual_curation_prodrugs.pdf]

CMPD_PREF_NAME (Active metabolite)	CMPD_PREF_NAME (Prodrug removed, potency data for active metabolite-drug target interaction used in analysis)
2',3'-DIDEOXYADENOSINE TRIPHOSPHATE	Didanosine
7-ETHYL-10-HYDROXYCAMPTOTHECIN	Irinotecan
ABIRATERONE	Abiraterone acetate
AFIMOXIFENE	Tamoxifen
CANDESARTAN	Candesartan cilexetil
CLOFIBRIC ACID	Clofibrate
DABIGATRAN	Dabigatran etexilate
DESGLYMIDODRINE	Midodrine
DESLORATADINE	Loratidine
ENALAPRILAT	Enalapril
ETHINYL ESTRADIOL	Mestranol
FENOFIBRIC ACID	Fenofibrate
FEXOFENADINE	Terfenadine
FINGOLIMOD PHOSPHATE/FTY720-P	Fingolimod
FLUPROSTENOL	Travoprost
FOSINOPRILAT	Fosinopril
HALOPERIDOL	Haloperidol decanoate
HYDROXYFLUTAMIDE	Flutamide
LAMIVUDINE TRIPHOSPHATE	Lamivudine
LANINAMIVIR	Laninamivir octanoate
LOXOPROFEN TRANS ALCOHOL	Loxoprofen
OSELTAMIVIR CARBOXYLIC ACID	Oseltamivir
OXYPURINOL	Allopurinol
PREDNISOLONE	Prednisone
QUINAPRILAT	Quinapril
R-406	Fostamatinib
ROMIDESPSIN_DITHIOL	Romidepsin
SPIRAPRILAT	Spirapril
STAVUDINE TRIPHOSPHATE	Stavudine
SULINDAC SULFIDE	Sulindac
TESTOSTERONE	Testosterone propionate
ZIDOVUDINE TRIPHOSPHATE	Zidovudine
BETAMETHASONE	Betamethasone phosphoric acid
TERIFLUNOMIDE	Leflunomide
IMIDAPRILAT	Imidapril
ESTRADIOL	Estradiol benzoate
CYCLOGUANIL	Chloroguanide
MELAGATRAN	Ximelagatran

CMPD_PREF_NAME (Active metabolite)	CMPD_PREF_NAME (Prodrug removed, not used in analysis since no potency data for active metabolite-drug target interaction)
CARBOVIR TRIPHOSPHAT	Abacavir
5-AZA-2′-DEOXYCYTIDINE TRIPHC	Azacitidine
DECITABINE TRIPHOSPHAT	Decitabine
DIFLORASONE	Diflorasone diacetate
MOEXIPRILAT	Moexipril
PERINDOPRILAT	Perindopril
RAMIPRILAT	Ramipril
TAZAROTENIC ACID	Tazarotene
TRANDOLAPRILAT	Trandolapril
BENAZEPRILAT	Benazepril
MEVINOLINIC ACID	Lovastatin
TENIVASTATIN	Simvastatin
GS-461203	Sofosbuvir
DDCTP	Zalcitabine
OMEPRAZOLE SULFENIC AC	Omeprazole
ROXATIDINE	Roxatidine acetate
CAREBASTINE	Ebastine
4-OH-ENCLOMIPHENE	Enclomiphene
