# Supplementary material for: Target-based evaluation of ‘drug-like’ properties and ligand efficiencies
Source: J Med Chem. Author manuscript; Available in PMC 2021 Jun 11. (PMC7610969; doi:10.1021/acs.jmedchem.1c00416)
Supplement: Supporting figures S1-S10 [file EMS123358-supplement-Supporting_figures_S1_S10.pdf]

Supporting Information

Target-based evaluation of 'drug-like' properties and ligand efficiencies

Paul D. Leeson,[*1] A. Patricia Bento,[2] Anna Gaulton,[2] Anne Hersey,[2] Emma J. Manners,[2] Chris J. Radoux,[2] Andrew R. Leach.[2]

[1] Paul Leeson Consulting Ltd, The Malt House, Main Street, Congerstone, Nuneaton, Warkwickshire CV13 6LZ, UK

[2] European Molecular Biology Laboratory, European Bioinformatics Institute, Wellcome Genome Campus, Hinxton, Cambridgeshire CB10 1SD, UK

Contents of Supporting information

Supporting Figures S1-S10. Pages S2-S12

Additional Excel Files.

Supporting Spreadsheet S1. Excel file containing: 1) drug, target median and [drug-target median] property values examined, together with target name and class, therapeutic use, approval year and period, and route of administration; 2) summary of drug count per target; 3) manual curation data: approval dates, prodrugs, and route of administration.

Supporting Spreadsheet S2. Excel file containing values of means, medians, 1st and 3rd quartiles, lower and upper adjacent limits, standard deviations and 95% confidence intervals (from DataWarrior[1]), for drugs, target medians and [drug-target median] differences, in the boxplots of Figures 5, 6, 7, 9, S2, S3, S4, S5, S6, S8, and S9.

1. Sander, T.; Freyss, J.; von Korff, M.; Rufener, C. DataWarrior: An Open-Source Program for Chemistry Aware Data Visualization and Analysis. *J. Chem. Inf. Model.* **2015,** *55*, 460–473. Downloaded from www.openmolecules.org.

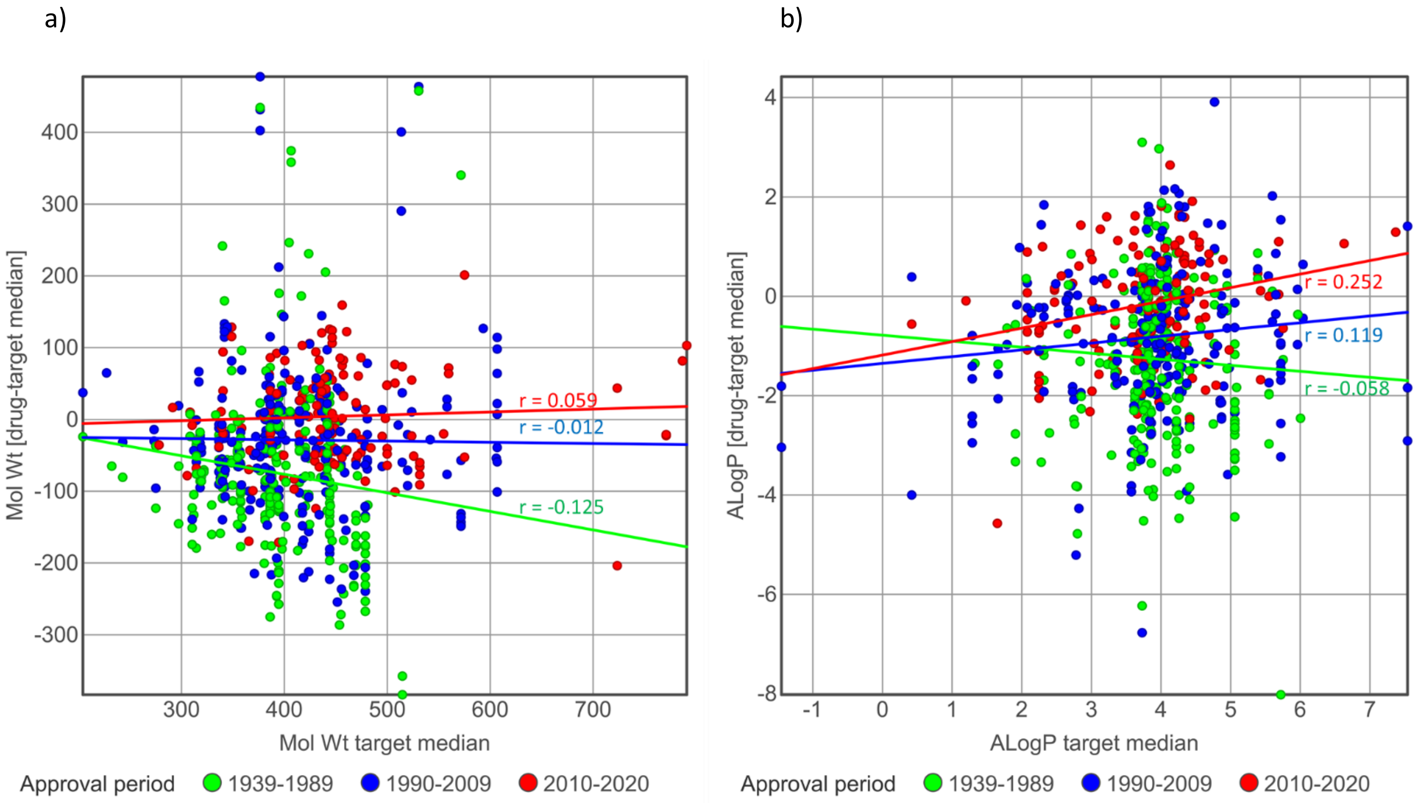

Supporting Figure S1. Correlations between target medians and [drug-target median] differences for a) MW and b) ALogP for 643 drugs acting at their primary targets. The median properties of the target have little impact on the [drug-target] difference.

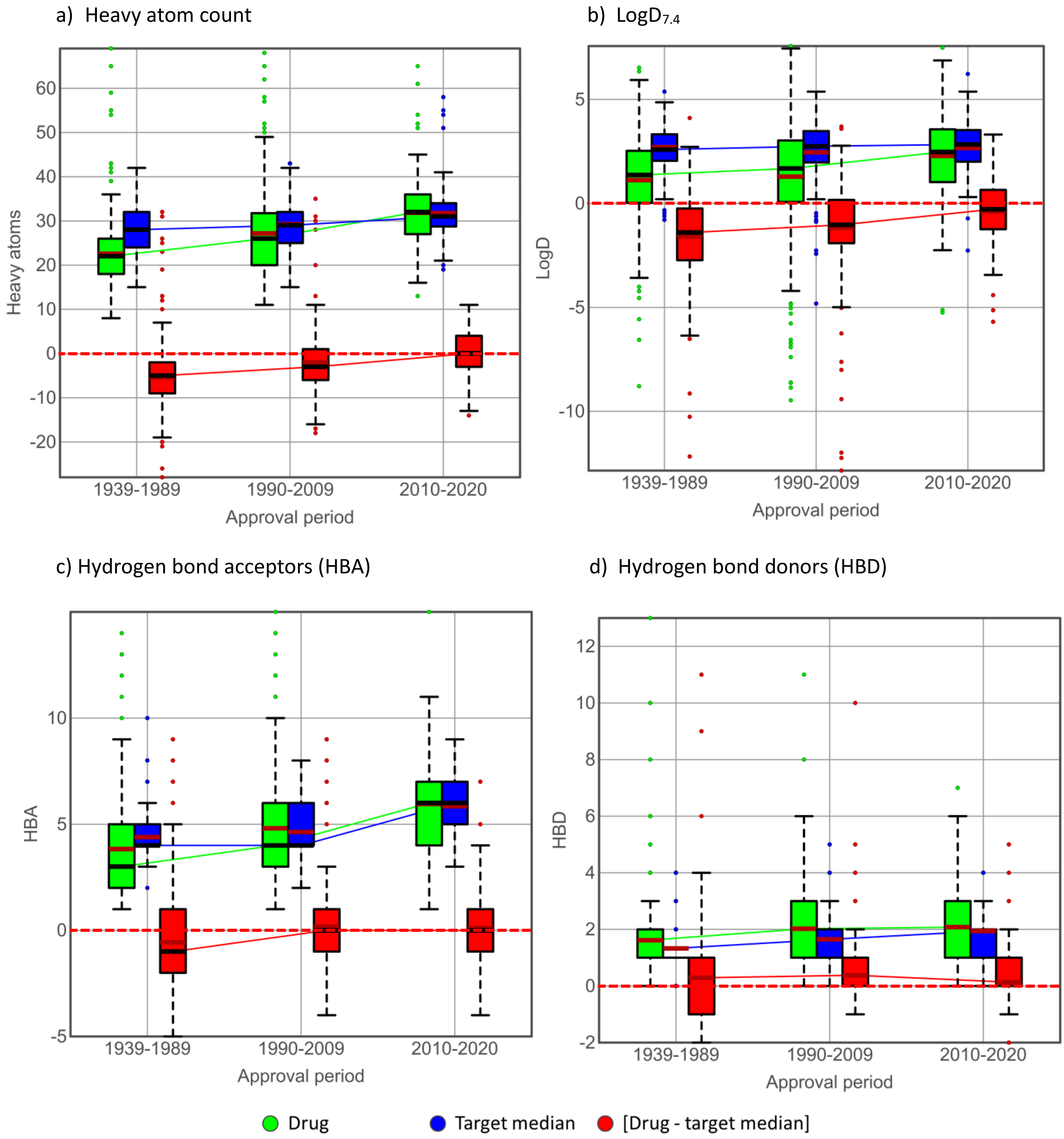

Supporting Figure S2. Box plots showing drug, target median and [drug-target median] properties in the three time periods for drug primary targets for a) heavy atom count, b) $LogD_{7.4}$, c) HBA and d) HBD. The time periods are connected by median values, except for HBD, where means are used. Mean and median values for all properties are in Tables 3-6, statistical values are in Supporting Spreadsheet 2.

a)

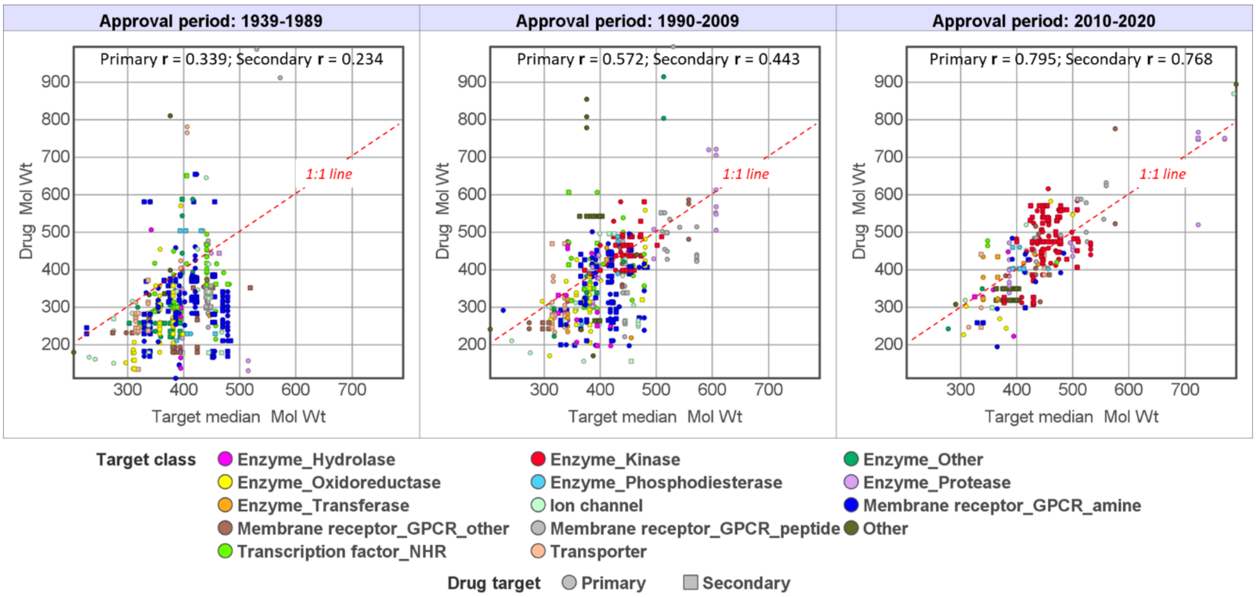

b)

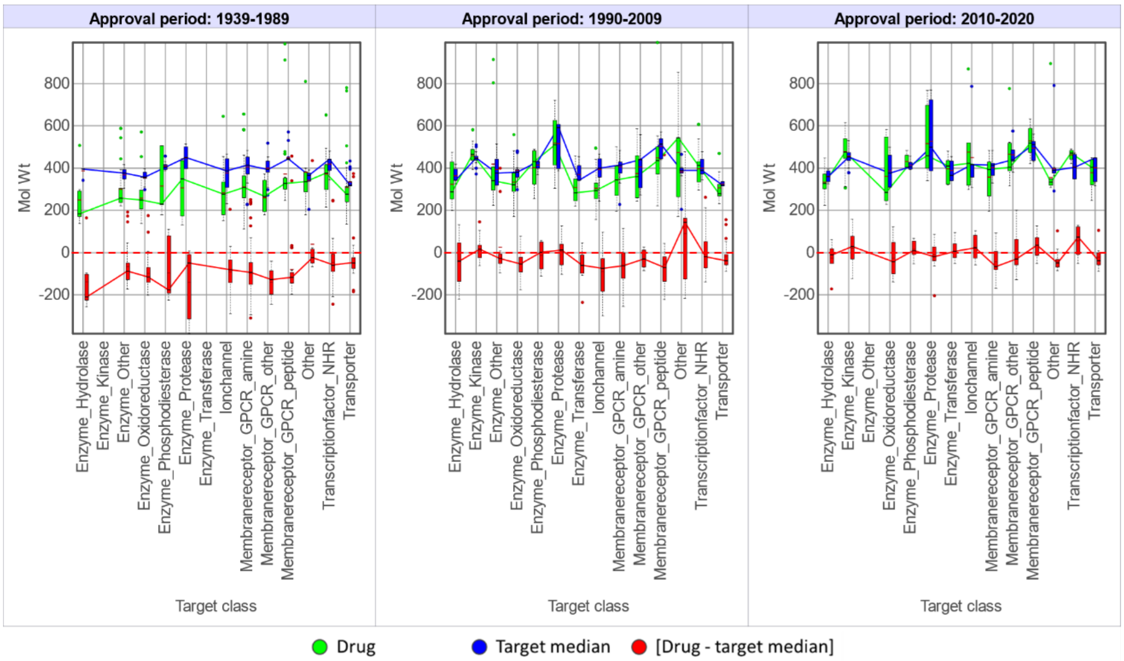

c)

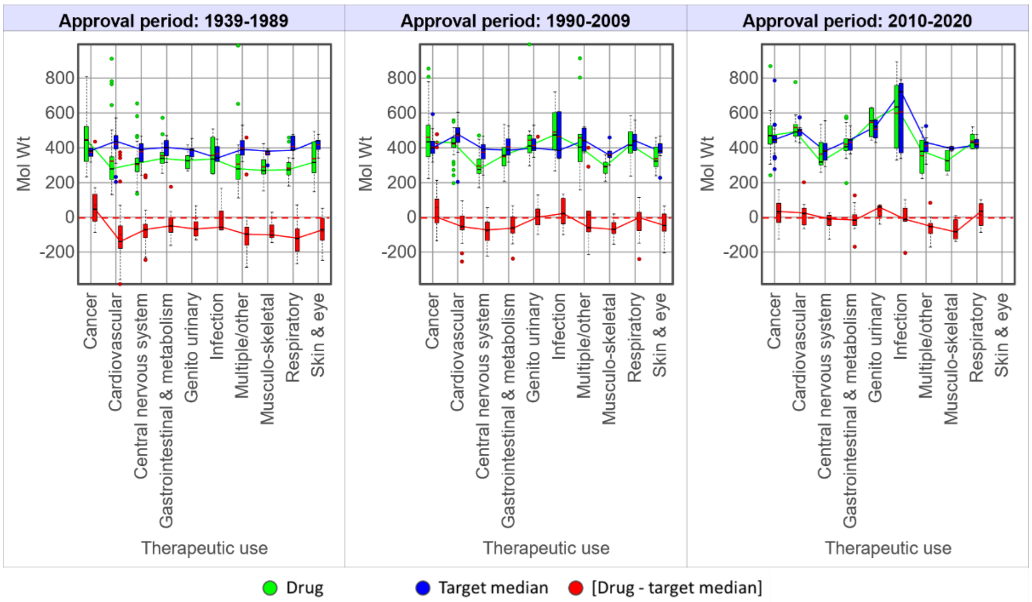

Supporting Figure S3. Drug and target comparator MW values by drug approval time period. a) Drug versus corresponding target medians. b) The data in a) split by target class. c) Therapeutic use. Statistical values for boxplots b) and c) are in Supporting Spreadsheet S2.

a)

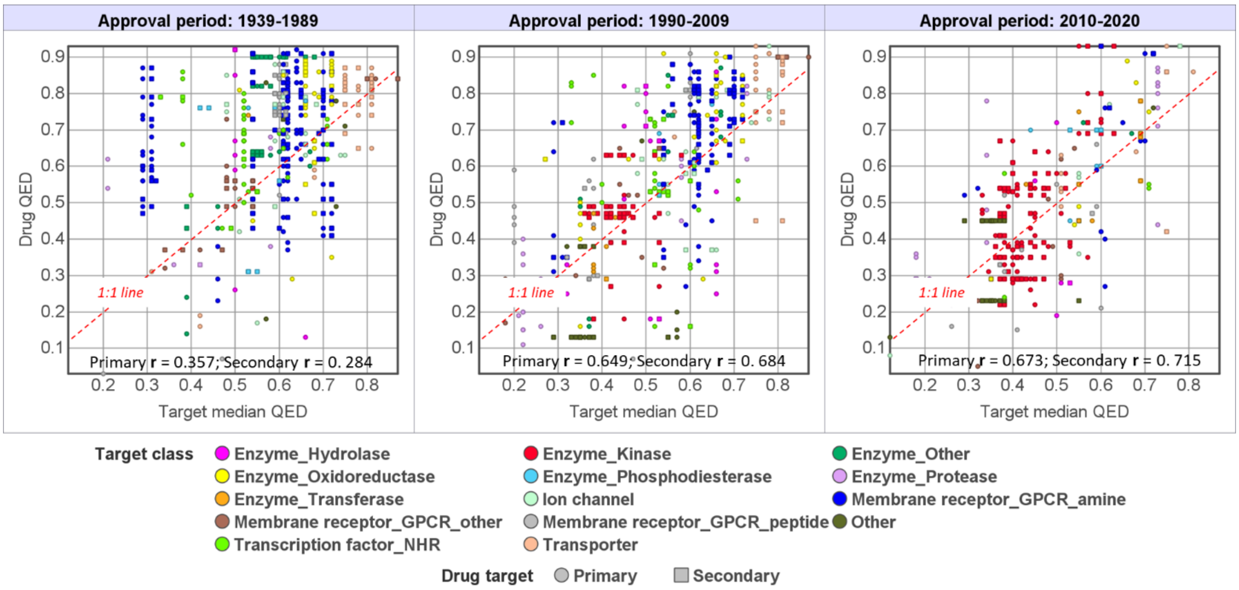

b)

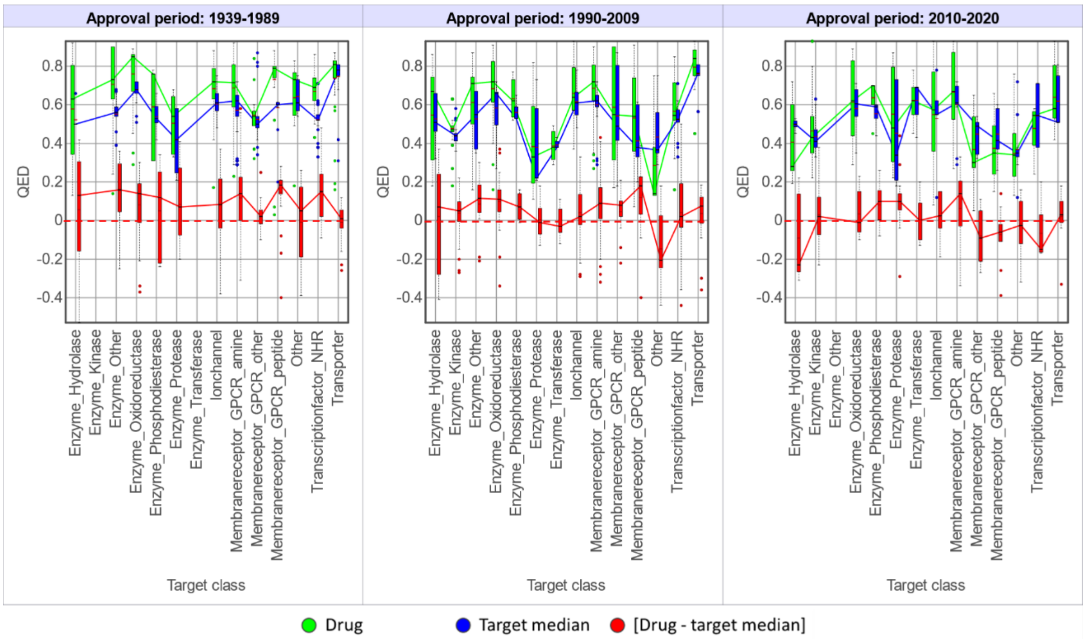

c)

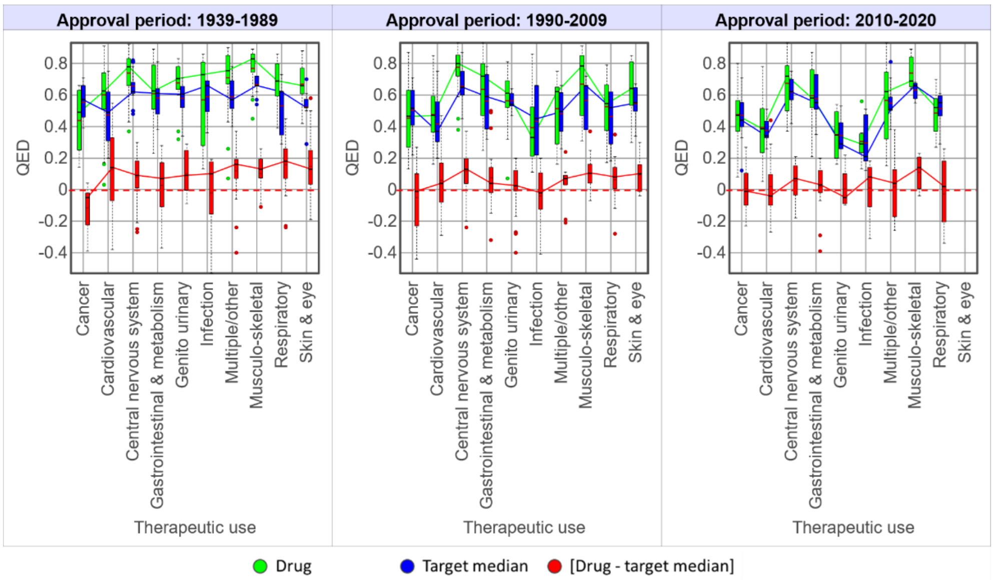

Supporting Figure S4. Drug and target comparator QED values by drug approval time period. a) Drug versus corresponding target medians. b) The data in a) split by target class. c) Therapeutic use. Statistical values for boxplots b) and c) are in Supporting Spreadsheet S2.

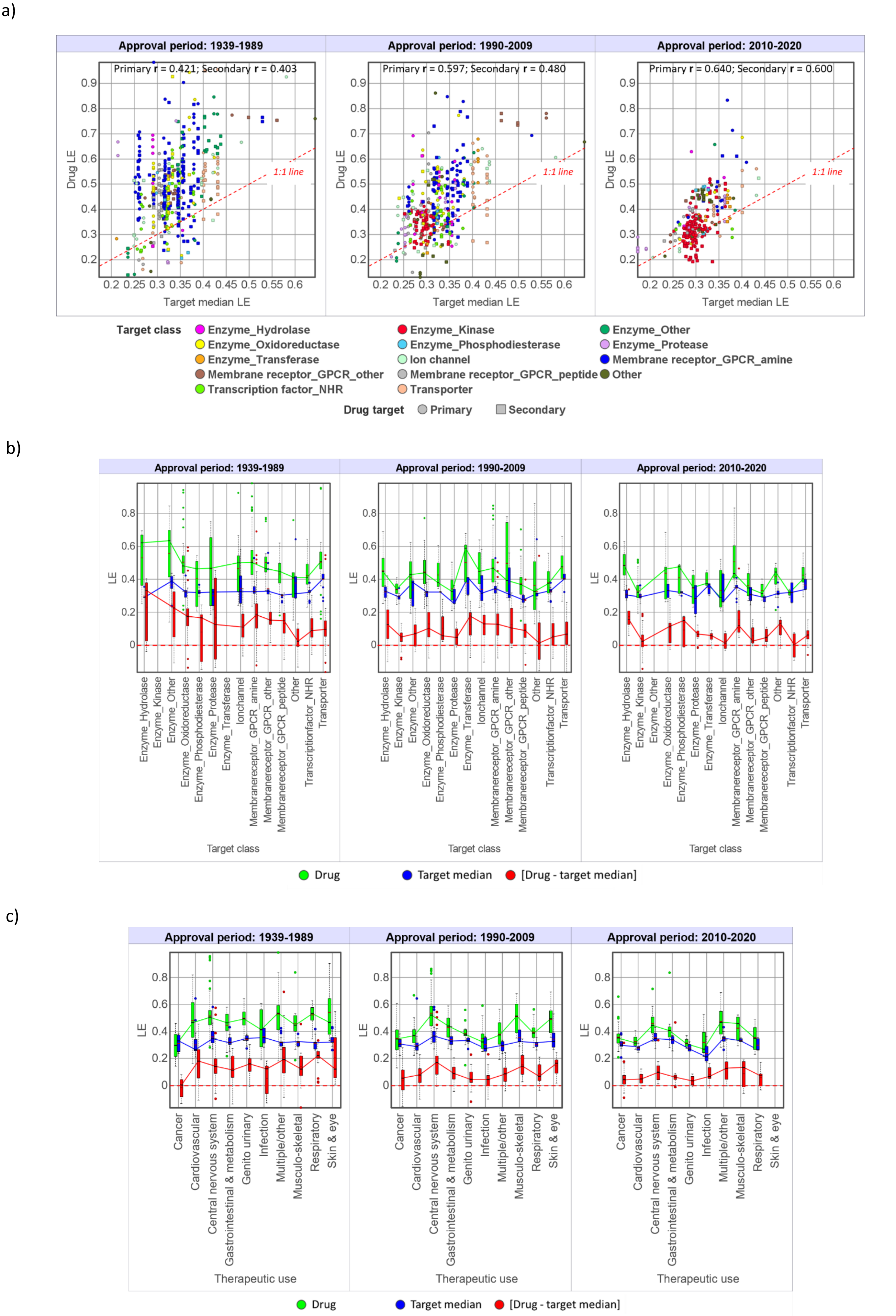

Supporting Figure S5. Drug and target comparator LE values by drug approval time period. a) Drug versus corresponding target medians. b) The data in a) split by target class. c) Therapeutic use. Statistical values for boxplots b) and c) are in Supporting Spreadsheet S2.

a)

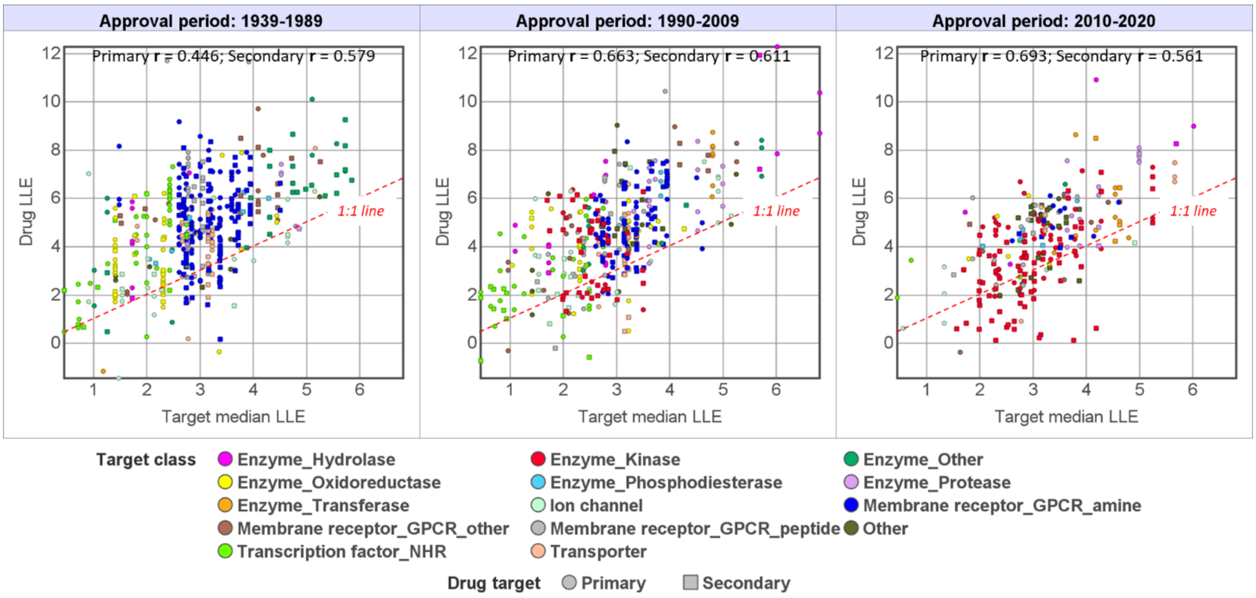

b)

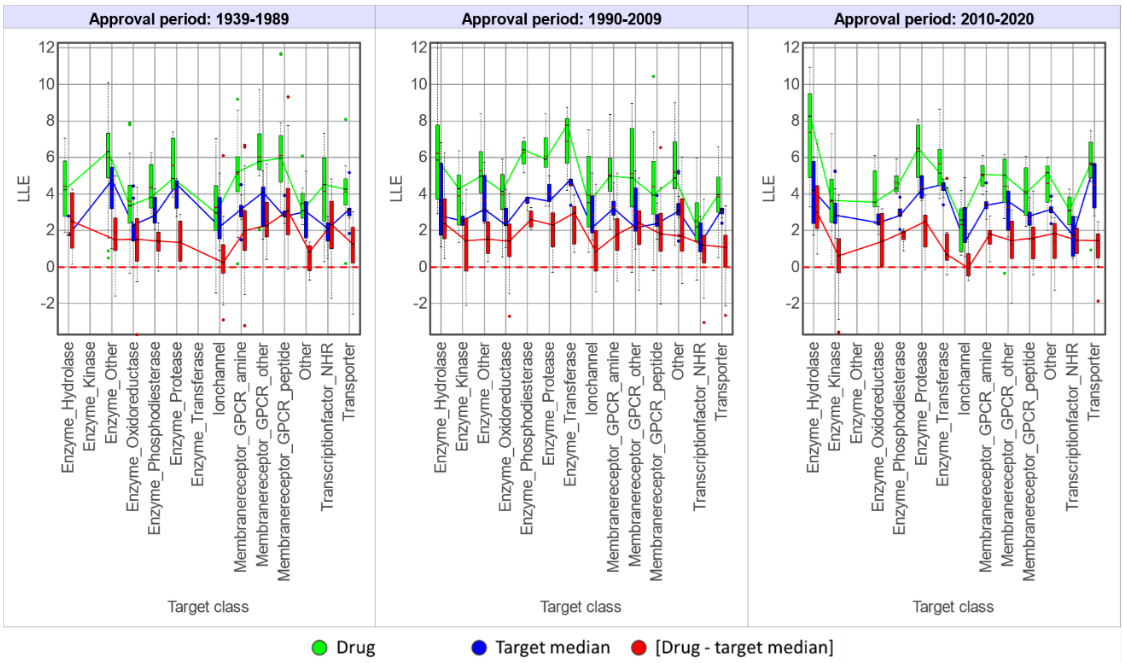

c)

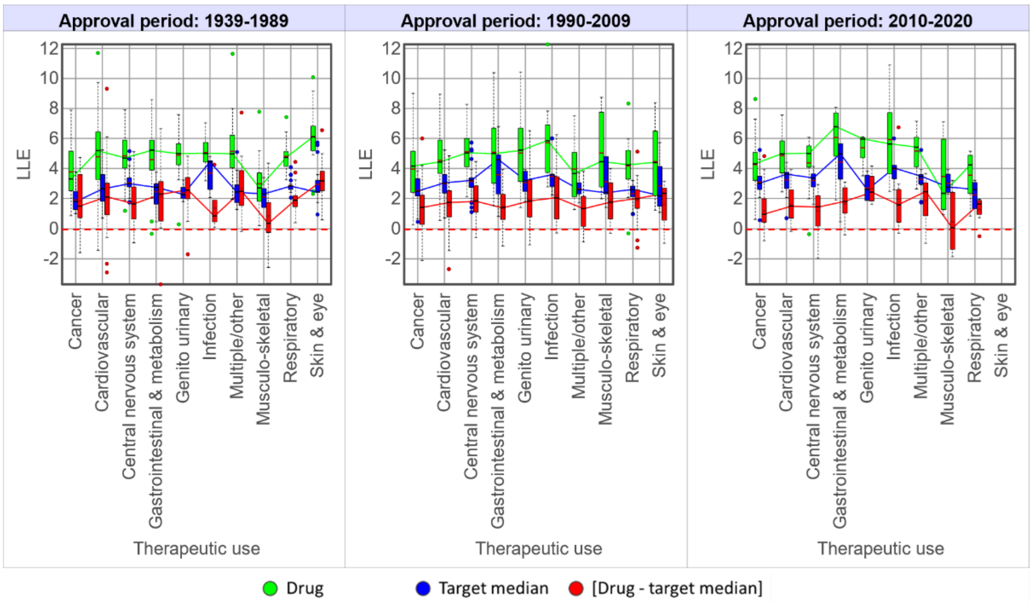

Supporting Figure S6. Drug and target comparator LLE values by drug approval time period. a) Drug versus corresponding target medians. b) The data in a) split by target class. c) Therapeutic use. Statistical values for boxplots b) and c) are in Supporting Spreadsheet S2.

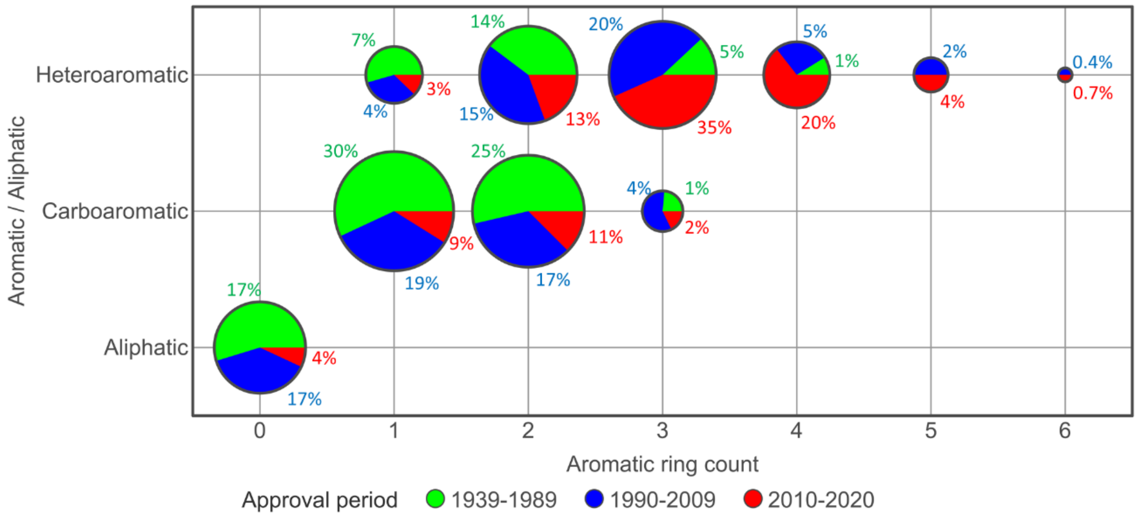

Supporting Figure S7. The balance of aliphatic and aromatic properties of drugs in this study. Carboaromatic and heteroaromatic drugs are defined as those with zero and ≥ 1 aromatic heteroatoms respectively. In each category, the percentages are shown for the total in each time frame.

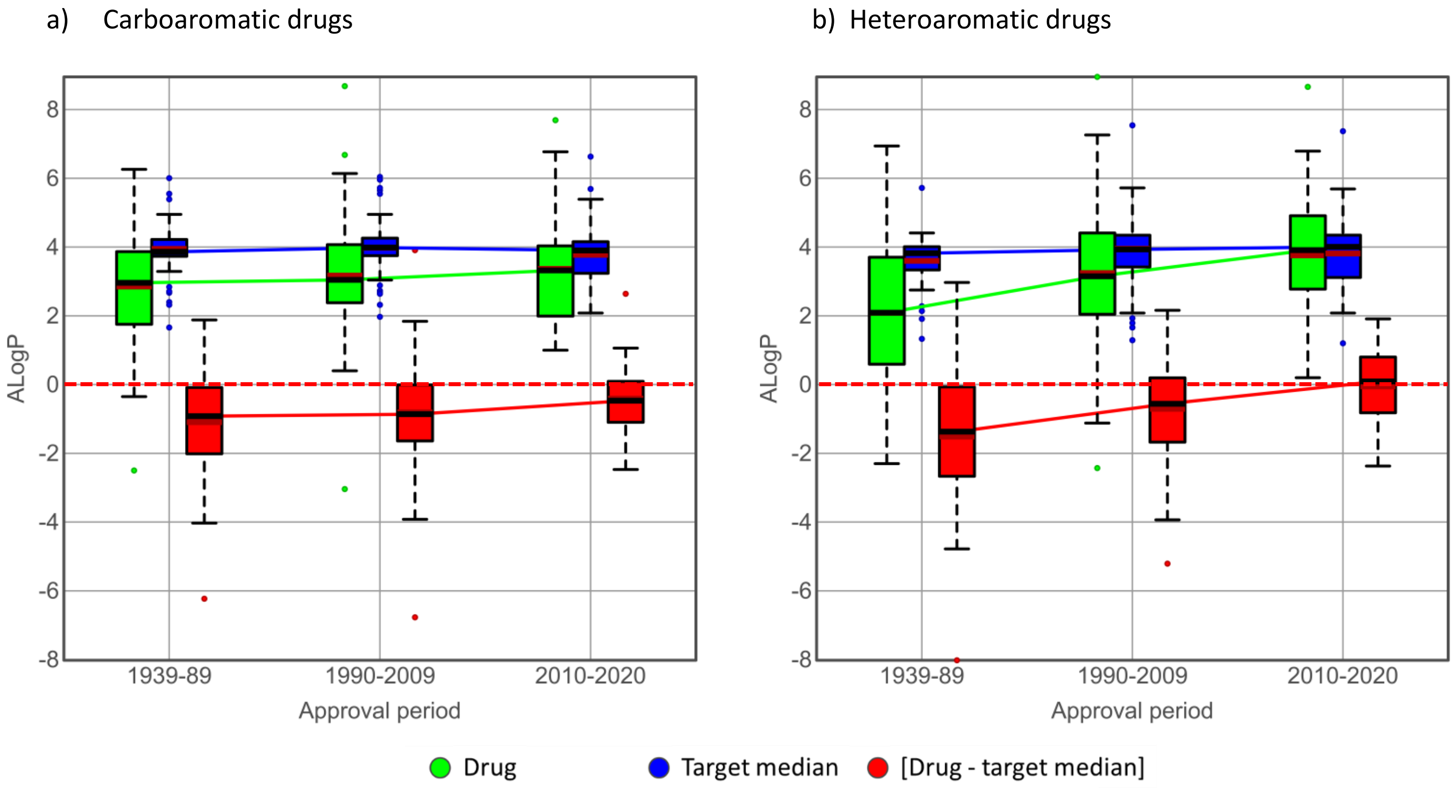

Supporting Figure S8. Time dependence of drug, target and [drug-target] ALogP by a) carboaromatic drugs (aromatic heteroatoms = 0) and b) heteroaromatic drugs (aromatic heteroatoms ≥ 1). Statistical values are in Supporting Spreadsheet S2.

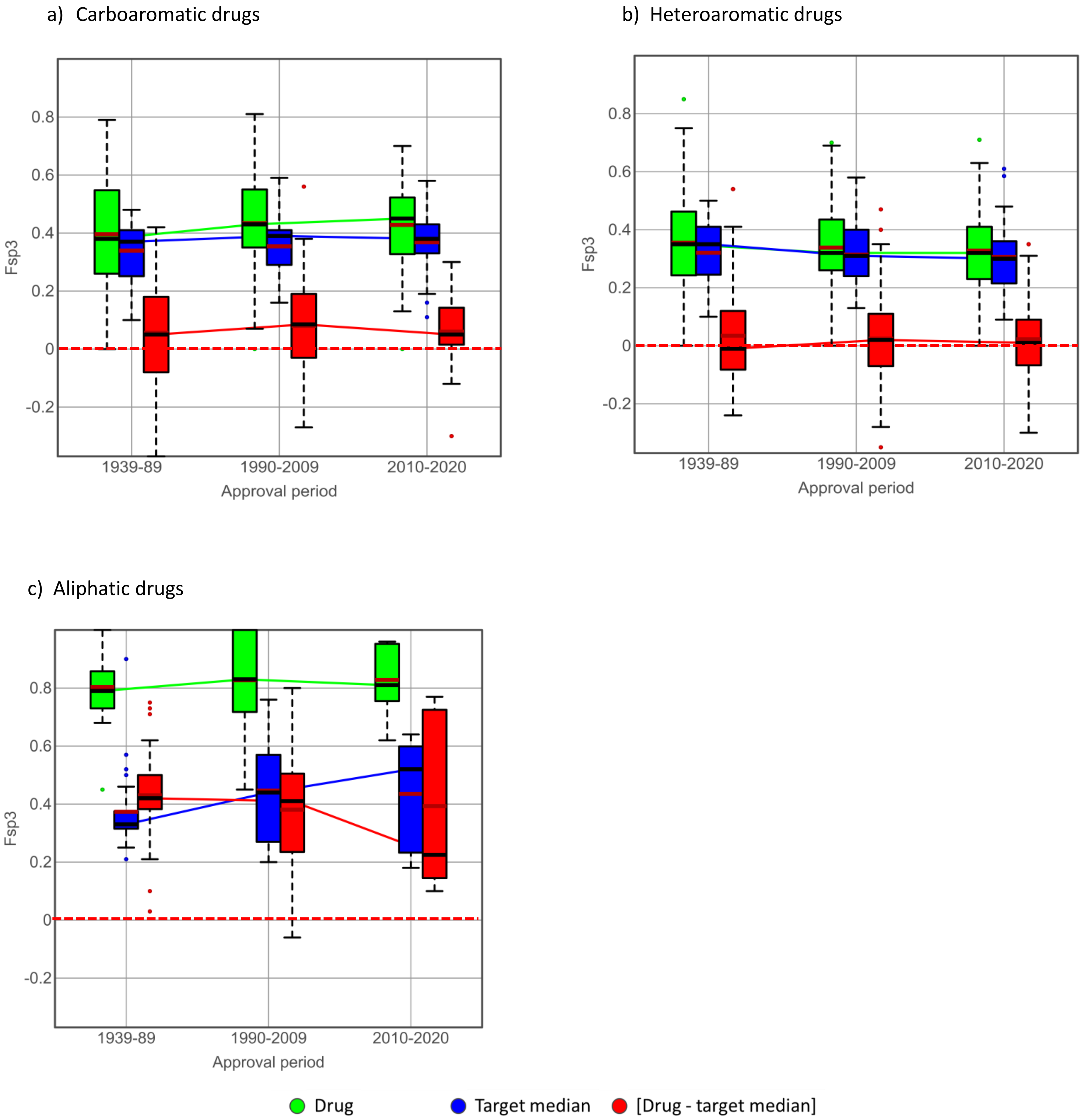

Supporting Figure S9. Time dependence of drug, target and [drug-target] Fsp3 by a) carboaromatic drugs (aromatic heteroatoms = 0), b) heteroaromatic drugs (aromatic heteroatoms ≥ 1) and c) aliphatic drugs. Statistical values are in Supporting Spreadsheet S2.

a)

	pCHEMBL	LE	BEI	FQ	SILE	LLE	SEI	LLEAT	LELP	AEI	Mol Wt	Heavy atoms	ALogP
LE	0.101												
BEI	0.114	0.975											
FQ	0.882	0.548	0.549										
SILE	0.819	0.643	0.640	0.987									
LLE	0.438	0.376	0.348	0.534	0.541								
SEI	0.102	0.298	0.328	0.222	0.258	-0.104							
LLEAT	0.043	0.819	0.775	0.411	0.482	0.727	0.038						
LELP	0.108	-0.557	-0.530	-0.172	-0.249	-0.746	-0.012	-0.774					
AEI	0.129	0.420	0.455	0.299	0.342	0.061	0.939	0.214	-0.159				
Mol Wt	0.345	-0.776	-0.791	-0.097	-0.217	-0.074	-0.243	-0.591	0.553	-0.313			
Heavy atoms	0.353	-0.793	-0.772	-0.098	-0.221	-0.090	-0.228	-0.615	0.576	-0.297	0.981		
ALogP	0.261	-0.329	-0.291	0.073	0.018	-0.753	0.187	-0.750	0.881	0.029	0.332	0.355	
PSA	0.061	-0.505	-0.553	-0.211	-0.280	0.360	-0.466	-0.064	-0.051	-0.444	0.667	0.633	-0.342

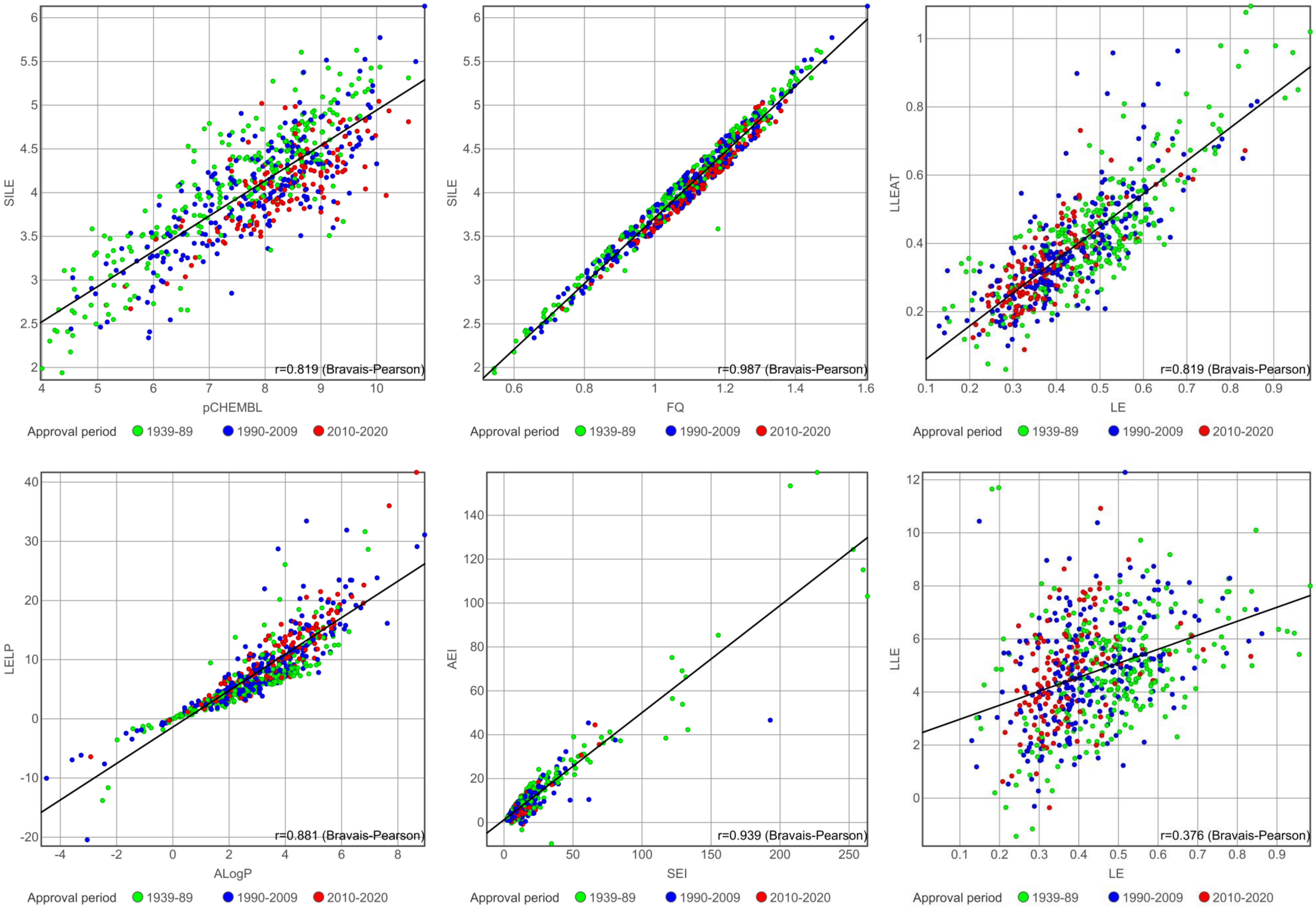

Supporting Figure S10. Cross correlations between efficiency metrics and parent properties of the drugs (n=643) used in this study. a) Correlation matrix and r values. b) Plots showing the highlighted correlations: SILE vs pChEMBL; SILE vs FQ; LLEAT vs LE; LELP vs ALogP; AEI vs SEI; and LLE vs LE.
