# Supplementary material for: Target-based evaluation of ‘drug-like’ properties and ligand efficiencies
Source: J Med Chem. Author manuscript; Available in PMC 2021 Jun 11. (PMC7610969; doi:10.1021/acs.jmedchem.1c00416)
Supplement: Fig 7 pChEMBL values [file EMS123358-supplement-Fig_7_pChEMBL_values.pdf]

pCHEMBL

Approval period	Target class	Group	Total Count	Outlier Count	Mean Value	1st Quartile	Median	3rd Quartile	Lower Adjacent Limit	Upper Adjacent Limit	Standard Deviation	Confidence Interval (95%)
1939-89	Enzyme_Hydrolase	Drug	7	0	5.8543	4.725	6.11	7.0175	4.07	7.37	1.2783	4.9073-6.8013
1939-89	Enzyme_Hydrolase	Target median	7	1	6.0807	6.05	6.05	6.05	6.05	6.05	0.081262	6.0205-6.1409
1939-89	Enzyme_Hydrolase	[Drug - target median]	7	0	-0.22643	-1.325	0.06	0.80625	-1.98	1.32	1.2424	-1.1468-0.69397
1939-89	Enzyme_Other	Drug	32	0	6.7475	5.61	7.255	7.6	4.28	9.13	1.3304	6.2865-7.2085
1939-89	Enzyme_Other	Target median	32	0	6.3972	6.0025	6.37	7.2375	4.89	7.55	0.88069	6.092-6.7023
1939-89	Enzyme_Other	[Drug - target median]	32	0	0.35031	-0.095	0.32	1.07	-1.78	1.935	0.89556	0.040016-0.66061
1939-89	Enzyme_Oxidoreductase	Drug	46	1	6.3446	5.77	6.475	6.91	4.28	8.17	0.97721	6.0622-6.627
1939-89	Enzyme_Oxidoreductase	Target median	46	0	5.7377	5.14	5.75	6.04	5.14	7.285	0.63444	5.5544-5.9211
1939-89	Enzyme_Oxidoreductase	[Drug - target median]	46	0	0.60685	-0.06	0.6	1.36	-1.99	2.75	1.1158	0.28441-0.92929
1939-89	Enzyme_Phosphodiesterase	Drug	9	0	5.8878	5.44	6.05	6.3425	4.17	6.78	0.78884	5.3724-6.4032
1939-89	Enzyme_Phosphodiesterase	Target median	9	0	6.9133	6.3125	7.02	7.3212	5.76	7.96	0.67762	6.4706-7.356
1939-89	Enzyme_Phosphodiesterase	[Drug - target median]	9	0	-1.0256	-1.9488	-1.06	-0.38	-2.15	0.49	0.8897	-1.6068--0.44429
1939-89	Enzyme_Protease	Drug	7	0	6.1014	4.4275	4.94	8.09	4.4	8.54	1.9434	4.6617-7.5411
1939-89	Enzyme_Protease	Target median	7	0	6.64	5.8675	6.82	7.135	5.55	7.68	0.80777	6.0416-7.2384
1939-89	Enzyme_Protease	[Drug - target median]	7	0	-0.53857	-2.335	-0.61	1.27	-3.28	1.72	1.9819	-2.0068-0.92966
1939-89	Ion channel	Drug	24	0	6.2679	4.935	5.52	7.96	4.25	9.2	1.6453	5.6097-6.9262
1939-89	Ion channel	Target median	24	0	6.2865	5.58	5.885	6.85	5.35	8	0.9211	5.9179-6.655
1939-89	Ion channel	[Drug - target median]	24	1	-0.01854	-0.785	-0.02	0.5075	-1.9	2.22	1.2862	-0.53312-0.49604
1939-89	Membrane receptor_GPCR_amine	Drug	180	0	7.7139	6.895	7.86	8.61	4.69	10.57	1.1852	7.5408-7.8871
1939-89	Membrane receptor_GPCR_amine	Target median	180	3	6.8312	6.57	6.73	7.2	6.065	7.82	0.49052	6.7595-6.9028
1939-89	Membrane receptor_GPCR_amine	[Drug - target median]	180	1	0.88278	0.2025	0.845	1.8	-2.16	3.84	1.2127	0.70562-1.0599
1939-89	Membrane receptor_GPCR_other	Drug	23	0	6.7926	4.9275	6.51	8.505	4.36	9.64	1.9396	5.9999-7.5853
1939-89	Membrane receptor_GPCR_other	Target median	23	3	6.9826	6.74	6.89	7.1825	6.27	7.54	0.49247	6.7813-7.1839
1939-89	Membrane receptor_GPCR_other	[Drug - target median]	23	0	-0.19	-2	-0.01	1.4275	-2.55	2.51	1.7932	-0.92285-0.54285
1939-89	Membrane receptor_GPCR_peptide	Drug	31	1	7.971	7.235	8.39	9.125	4.71	9.79	1.4257	7.4691-8.4728
1939-89	Membrane receptor_GPCR_peptide	Target median	31	7	7.1287	7.07	7.09	7.09	7.07	7.09	0.23504	7.046-7.2115
1939-89	Membrane receptor_GPCR_peptide	[Drug - target median]	31	2	0.84226	0.235	1.26	1.9125	-0.95	2.72	1.3723	0.35917-1.3253
1939-89	Other	Drug	7	0	6.6757	6.19	6.61	7.1625	5.66	7.71	0.69183	6.1632-7.1882
1939-89	Other	Target median	7	0	6.3386	5.63	5.96	7.325	5.25	7.4	0.92785	5.6512-7.0259
1939-89	Other	[Drug - target median]	7	0	0.33714	0.055	0.14	0.8125	-0.94	1.71	0.82724	-0.27568-0.94997
1939-89	Transcription factor_NHR	Drug	46	7	7.6909	7.41	8.26	8.73	5.58	9.9	1.5828	7.2335-8.1483
1939-89	Transcription factor_NHR	Target median	46	0	6.9917	6.645	6.82	7.68	6.09	7.68	0.57825	6.8246-7.1588
1939-89	Transcription factor_NHR	[Drug - target median]	46	0	0.69913	-0.04	0.975	1.55	-2.24	2.96	1.4614	0.27681-1.1214
1939-89	Transporter	Drug	35	2	7.3517	6.885	7.52	8.0525	5.58	8.88	1.1127	6.9831-7.7203
1939-89	Transporter	Target median	35	2	6.8209	6.6975	6.82	7.1125	6.61	7.21	0.33167	6.711-6.9307
1939-89	Transporter	[Drug - target median]	35	3	0.53086	0.105	0.69	1.085	-1.24	2.3	1.1092	0.1634-0.89832
1990-2009	Enzyme_Hydrolase	Drug	19	0	6.9579	6.045	6.73	7.6675	5.43	8.7	1.0132	6.5023-7.4135
1990-2009	Enzyme_Hydrolase	Target median	19	0	5.9555	5.5887	6.05	6.265	5.295	6.33	0.36667	5.7907-6.1204
1990-2009	Enzyme_Hydrolase	[Drug - target median]	19	0	1.0024	0.03875	0.77	1.7837	-0.37	2.835	1.0333	0.53775-1.467
1990-2009	Enzyme_Kinase	Drug	51	3	7.9198	7.5125	7.81	8.355	6.69	9.59	0.84261	7.6885-8.1511
1990-2009	Enzyme_Kinase	Target median	51	1	6.9031	6.66	6.8	7.11	6.275	7.7	0.40611	6.7917-7.0146
1990-2009	Enzyme_Kinase	[Drug - target median]	51	4	1.0167	0.4125	0.99	1.39	-0.85	2.8	1.0431	0.73037-1.303
1990-2009	Enzyme_Other	Drug	12	0	7.54	6.51	7.765	8.565	5.31	9.22	1.2283	6.845-8.235
1990-2009	Enzyme_Other	Target median	12	0	6.5417	6.24	6.275	7.035	5.86	7.37	0.56048	6.2245-6.8588
1990-2009	Enzyme_Other	[Drug - target median]	12	0	0.99833	0.385	1.205	1.85	-1	2.52	1.1026	0.37447-1.6222
1990-2009	Enzyme_Oxidoreductase	Drug	32	0	7.2109	6.3	7.08	8.075	5.05	9.04	1.0011	6.8641-7.5578
1990-2009	Enzyme_Oxidoreductase	Target median	32	0	6.2689	5.62	6.04	6.56	5.14	7.88	0.93093	5.9464-6.5915
1990-2009	Enzyme_Oxidoreductase	[Drug - target median]	32	1	0.94203	0.195	0.885	1.53	-0.62	3.4	1.2357	0.5139-1.3702
1990-2009	Enzyme_Phosphodiesterase	Drug	4	0	8.225	7.71	8.325	8.74	7.1	9.15	0.84485	7.3971-9.053
1990-2009	Enzyme_Phosphodiesterase	Target median	4	0	6.9125	6.525	7.3	7.3	5.75	7.3	0.775	6.153-7.672
1990-2009	Enzyme_Phosphodiesterase	[Drug - target median]	4	0	1.3125	1.025	1.19	1.6	1.02	1.85	0.38973	0.93056-1.6944
1990-2009	Enzyme_Protease	Drug	19	0	8.7174	8.13	8.8	9.1825	7.55	10	0.70726	8.3993-9.0354
1990-2009	Enzyme_Protease	Target median	19	0	7.3361	6.82	7.26	7.92	6.405	7.92	0.59636	7.0679-7.6042
1990-2009	Enzyme_Protease	[Drug - target median]	19	0	1.3813	0.93	1.31	1.8875	0.45	2.28	0.54474	1.1364-1.6263
1990-2009	Enzyme_Transferase	Drug	9	0	6.5633	5.91	6.5	7.15	4.63	8.62	1.1976	5.7809-7.3458
1990-2009	Enzyme_Transferase	Target median	9	3	6.1844	6.045	6.07	6.07	6.07	6.07	0.42057	5.9097-6.4592
1990-2009	Enzyme_Transferase	[Drug - target median]	9	1	0.37889	-0.06	0.43	1.08	-0.84	2.55	1.4697	-0.58132-1.3391
1990-2009	Ion channel	Drug	25	0	6.6508	5.1	6.4	8.02	4.02	9.49	1.7042	5.9827-7.3189
1990-2009	Ion channel	Target median	25	0	6.563	5.58	6.42	7.3525	5.35	8	0.93015	6.1984-6.9276
1990-2009	Ion channel	[Drug - target median]	25	0	0.0878	-0.9825	0.18	0.98125	-2.69	2.63	1.4276	-0.47181-0.64741
1990-2009	Membrane receptor_GPCR_amine	Drug	107	3	8.0061	7.3825	8.11	8.7275	5.45	10.7	1.1009	7.7975-8.2147
1990-2009	Membrane receptor_GPCR_amine	Target median	107	0	7.0293	6.73	6.85	7.22	6.065	7.82	0.42908	6.948-7.1106
1990-2009	Membrane receptor_GPCR_amine	[Drug - target median]	107	3	0.97678	0.345	1	1.7038	-1.24	3.13	1.0883	0.77057-1.183
1990-2009	Membrane receptor_GPCR_other	Drug	17	0	8.7753	8.06	8.44	9.955	6.53	10.86	1.3057	8.1546-9.396
1990-2009	Membrane receptor_GPCR_other	Target median	17	0	7.4659	6.855	7.54	8.14	6.27	8.23	0.67458	7.1452-7.7866
1990-2009	Membrane receptor_GPCR_other	[Drug - target median]	17	0	1.3094	0.485	1.33	2.18	-0.77	3.32	1.1381	0.76837-1.8505
1990-2009	Membrane receptor_GPCR_peptide	Drug	27	0	8.2548	7.52	8.52	9.3675	5.43	9.92	1.2956	7.7661-8.7435
1990-2009	Membrane receptor_GPCR_peptide	Target median	27	0	7.4006	7.09	7.3	7.9763	5.95	8.1	0.52077	7.2041-7.597
1990-2009	Membrane receptor_GPCR_peptide	[Drug - target median]	27	0	0.85426	-0.09	0.86	1.9375	-1.63	2.85	1.2502	0.38268-1.3258
1990-2009	Other	Drug	20	0	7.072	5.92	7.215	7.855	4.96	9.92	1.3047	6.5002-7.6438
1990-2009	Other	Target median	20	0	6.3982	6.02	6.5	6.75	5.63	7.1	0.47523	6.19-6.6065
1990-2009	Other	[Drug - target median]	20	2	0.67375	0.1675	0.64	1.225	-1	2.47	1.1274	0.17967-1.1678
1990-2009	Transcription factor_NHR	Drug	36	1	7.8036	7.415	7.8	8.58	5.96	9.71	1.1002	7.4442-8.163
1990-2009	Transcription factor_NHR	Target median	36	4	6.8161	6.52	6.8025	6.94	6.09	7.05	0.38874	6.6891-6.9431
1990-2009	Transcription factor_NHR	[Drug - target median]	36	9	0.9875	0.635	0.93	1.205	0.06	2.03	0.98083	0.6671-1.3079
1990-2009	Transporter	Drug	24	0	7.2196	6.12	7.13	8.28	5.25	9.93	1.2939	6.7019-7.7372
1990-2009	Transporter	Target median	24	2	6.8171	6.715	6.82	7.21	6.61	7.21	0.53551	6.6028-7.0313
1990-2009	Transporter	[Drug - target median]	24	0	0.4025	-0.505	0.425	1.21	-1.57	2.72	1.1708	-0.065904-0.8709
2010-2020	Enzyme_Hydrolase	Drug	5	0	7.98	7.505	8	8.31	7.37	8.85	0.5781	7.4733-8.4867
2010-2020	Enzyme_Hydrolase	Target median	5	0	5.756	5.46	5.59	6.12	5.295	6.33	0.42251	5.3857-6.1263
2010-2020	Enzyme_Hydrolase	[Drug - target median]	5	0	2.224	2.0213	2.41	2.5438	1.32	2.615	0.5228	1.7657-2.6823
2010-2020	Enzyme_Kinase	Drug	116	2	7.9158	7.385	8.13	8.505	5.84	9.98	0.94003	7.7447-8.0868
2010-2020	Enzyme_Kinase	Target	116	0	7.1961	6.8	7.18	7.495	6.35	8.22	0.46053	7.1123-7.2799
2010-2020	Enzyme_Kinase	[Drug - target median]	116	0	0.7197	0.045	0.78	1.385	-1.72	2.95	0.99766	0.53814-0.90125
2010-2020	Enzyme_Oxidoreductase	Drug	6	0	7.41	6.52	7.72	7.92	6.04	8.54	0.93926	6.6584-8.1616
2010-2020	Enzyme_Oxidoreductase	Target median	6	0	6.5117	6.04	6.525	7	5.75	7.23	0.55819	6.065-6.9583
2010-2020	Enzyme_Oxidoreductase	[Drug - target median]	6	0	0.89833	0.01	0.745	1.89	0	2	0.88334	0.19151-1.6052
2010-2020	Enzyme_Phosphodiesterase	Drug	7	0	8.46	7.58	9.06	9.255	7.13	9.35	0.94991	7.7563-9.1637
2010-2020	Enzyme_Phosphodiesterase	Target median	7	0	6.7621	6.32	6.92	7.1788	6.29	7.3	0.44831	6.43-7.0943
2010-2020	Enzyme_Phosphodiesterase	[Drug - target median]	7	0	1.6979	0.6525	2.085	2.62	0.46	2.99	1.0581	0.91399-2.4817
2010-2020	Enzyme_Protease	Drug	19	1	8.6632	8.3225	8.66	9.0375	7.85	9.8	0.6922	8.3519-8.9744
2010-2020	Enzyme_Protease	Target median	19	0	7.0505	6.615	7.26	7.26	6.37	7.7	0.43792	6.8536-7.2474
2010-2020	Enzyme_Protease	[Drug - target median]	19	3	1.6126	1.3175	1.6	1.835	0.66	2.29	0.81376	1.2467-1.9785
2010-2020	Enzyme_Transferase	Drug	14	0	8.3364	7.82	8.265	9.07	7.21	9.3	0.64099	8.0007-8.6722
2010-2020	Enzyme_Transferase	Target median	14	0	7.0271	6.84	7.05	7.26	6.48	7.3	0.25348	6.8944-7.1599
2010-2020	Enzyme_Transferase	[Drug - target median]	14	0	1.3093	0.78	1.325	1.81	0.25	2.41	0.69279	0.94638-1.6722
2010-2020	Ion channel	Drug	6	0	7.3833	6.57	7.405	8.04	5.59	9.29	1.2708	6.3665-8.4002
2010-2020	Ion channel	Target median	6	0	6.565	5.68	6.265	7.69	5.68	7.81	1.0244	5.7453-7.3847
2010-2020	Ion channel	[Drug - target median]	6	0	0.81833	0.23	0.85	1.47	-0.09	1.6	0.66514	0.28611-1.3506
2010-2020	Membrane receptor_GPCR_amine	Drug	15	0	8.566	7.8475	8.6	9.3625	7.16	10.22	1.0115	8.0541-9.0779
2010-2020	Membrane receptor_GPCR_amine	Target median	15	0	7.1373	6.73	7.2	7.59	6.29	7.82	0.49224	6.8882-7.3864
2010-2020	Membrane receptor_GPCR_amine	[Drug - target median]	15	0	1.4287	0.795	1.51	2.155	0.13	2.63	0.81598	1.0157-1.8416
2010-2020	Membrane receptor_GPCR_other	Drug	9	0	8.1944	7.575	8.25	9.4	5.49	9.8	1.3469	7.3145-9.0744
2010-2020	Membrane receptor_GPCR_other	Target median	9	0	7.2533	6.7475	7.34	7.92	6.46	7.92	0.573	6.879-7.6277
2010-2020	Membrane receptor_GPCR_other	[Drug - target median]	9	1	0.94111	0.4825	1.36	1.5575	0.4	1.88	1.0197	0.27489-1.6073
2010-2020	Membrane receptor_GPCR_peptide	Drug	14	0	8.6964	8.07	9.01	9.22	6.41	10.57	1.0482	8.1474-9.2455
2010-2020	Membrane receptor_GPCR_peptide	Target median	14	1	7.1664	6.86	7.085	7.4	6.66	8.005	0.5544	6.876-7.4568
2010-2020	Membrane receptor_GPCR_peptide	[Drug - target median]	14	0	1.53	1.015	1.455	2	0.2	2.75	0.75881	1.1325-1.9275
2010-2020	Other	Drug	26	1	7.5088	7.07	7.56	8.18	5.55	8.75	1.0036	7.1231-7.8946
2010-2020	Other	Target median	26	1	6.4681	6.08	6.47	6.62	5.69	6.92	0.62751	6.2269-6.7093
2010-2020	Other	[Drug - target median]	26	7	1.0408	0.8	1.095	1.28	0.5	1.7	0.69434	0.77387-1.3077
2010-2020	Transcription factor_NHR	Drug	4	0	7.635	6.875	7.395	8.395	6.75	9	1.0122	6.643-8.627
2010-2020	Transcription factor_NHR	Target median	4	0	6.5175	6.215	6.58	6.82	6.09	6.82	0.3639	6.1609-6.8741
2010-2020	Transcription factor_NHR	[Drug - target median]	4	0	1.1175	0.295	0.815	1.94	-0.07	2.91	1.272	-0.1291-2.3641
2010-2020	Transporter	Drug	11	1	8.0055	7.5775	8.51	8.63	6.5	9.35	1.215	7.2875-8.7235
2010-2020	Transporter	Target median	11	0	7.3073	7.21	7.52	7.52	6.81	7.52	0.28011	7.1417-7.4728
2010-2020	Transporter	[Drug - target median]	11	2	0.69818	0.5825	0.99	1.275	0.58	2.14	1.0429	0.081897-1.3145

Approval period	Therapeutic use	Group	Total Count	Outlier Count	Mean Value	1st Quartile	Median	3rd Quartile	Lower Adjacent Limit	Upper Adjacent Limit	Standard Deviation	Confidence Interval (95%)
1939-1989	Cancer	Drug	11	0	6.3282	5.2425	6.07	7.81	4.36	8.48	1.4726	5.4579-7.1984
1939-1989	Cancer	Target median	11	0	5.9173	5.29	6.24	6.5975	4.89	6.82	0.74158	5.479-6.3555
1939-1989	Cancer	[Drug - target median]	11	0	0.41091	-0.72	0.78	1.4325	-1.78	1.82	1.2151	-0.30718-1.129
1939-1989	Cardiovascular	Drug	46	0	6.8496	5.5	6.91	8.1	4.14	9.7	1.5723	6.3952-7.3039
1939-1989	Cardiovascular	Target median	46	0	6.2332	5.76	6.09	6.73	4.81	8.1	0.6321	6.0505-6.4158
1939-1989	Cardiovascular	[Drug - target median]	46	0	0.61641	-0.175	0.7275	1.4	-2.095	3.5	1.3086	0.23824-0.99459
1939-1989	Central nervous system	Drug	74	2	7.8482	7.1	8.2	8.68	4.77	10.57	1.3842	7.5329-8.1636
1939-1989	Central nervous system	Target median	74	10	6.8839	6.73	6.865	7.2	6.05	7.6	0.55728	6.7569-7.0109
1939-1989	Central nervous system	[Drug - target median]	74	4	0.96432	0.44	1.035	1.8	-1.31	3.84	1.226	0.68499-1.2437
1939-1989	Gastrointestinal & metabolism	Drug	18	0	7.8161	7.2	7.805	8.77	4.97	9.63	1.1243	7.2967-8.3355
1939-1989	Gastrointestinal & metabolism	Target median	18	3	6.5742	6.42	6.73	6.89	6.34	7.59	0.62446	6.2857-6.8627
1939-1989	Gastrointestinal & metabolism	[Drug - target median]	18	2	1.2419	1	1.18	1.95	0.46	3.12	1.059	0.75272-1.7312
1939-1989	Genito urinary	Drug	16	1	8.4587	8.195	8.695	9.025	7.83	9.9	1.0989	7.9203-8.9972
1939-1989	Genito urinary	Target median	16	3	6.8222	6.68	6.77	6.835	6.675	6.94	0.29302	6.6786-6.9658
1939-1989	Genito urinary	[Drug - target median]	16	1	1.6366	1.36	1.755	2.2575	1.03	2.96	1.0607	1.1168-2.1563
1939-1989	Infection	Drug	5	1	6.608	6.2425	6.96	7.2925	6.82	7.54	1.2043	5.5524-7.6636
1939-1989	Infection	Target median	5	0	6.635	6.0113	7.21	7.2175	5.25	7.24	0.87784	5.8655-7.4045
1939-1989	Infection	[Drug - target median]	5	0	-0.027	-0.975	0.33	1.1363	-2.73	1.71	1.6976	-1.515-1.461
1939-1989	Multiple/other	Drug	32	0	7.3744	6.045	7.785	8.595	4.54	9.24	1.4838	6.8603-7.8885
1939-1989	Multiple/other	Target median	32	0	6.57	6.05	6.6625	7.24	5.14	7.89	0.88551	6.2632-6.8768
1939-1989	Multiple/other	[Drug - target median]	32	0	0.80438	-0.325	0.95	1.9375	-2.1	2.79	1.5043	0.28317-1.3256
1939-1989	Musculo-skeletal	Drug	15	1	6.3953	5.96	6.47	7.09	5.52	7.4	0.89131	5.9443-6.8464
1939-1989	Musculo-skeletal	Target median	15	0	5.83	5.14	6.04	6.04	5.14	6.82	0.63608	5.5081-6.1519
1939-1989	Musculo-skeletal	[Drug - target median]	15	1	0.56533	0.0625	0.43	1.3325	-0.44	2.25	1.2304	-0.057347-1.188
1939-1989	Respiratory	Drug	23	0	7.6787	6.81	7.89	8.5625	5.28	9.9	1.3173	7.1403-8.217
1939-1989	Respiratory	Target median	23	3	6.807	6.57	6.82	6.82	6.29	6.82	0.33245	6.6711-6.9428
1939-1989	Respiratory	[Drug - target median]	23	0	0.87174	0.315	0.82	1.6775	-1.46	3.39	1.2193	0.37341-1.3701
1939-1989	Skin & eye	Drug	19	1	8.1574	7.4125	8.53	8.74	6.62	9.68	1.0531	7.6838-8.6309
1939-1989	Skin & eye	Target median	19	0	7.3305	7.0075	7.59	7.68	6.05	7.68	0.50595	7.103-7.558
1939-1989	Skin & eye	[Drug - target median]	19	0	0.82684	-0.0925	0.95	1.3175	-0.97	3.11	1.0722	0.34474-1.3089
1990-2009	Cancer	Drug	30	0	7.455	6.5	7.59	8.05	5.73	9.92	1.0966	7.0626-7.8474
1990-2009	Cancer	Target median	30	1	6.4472	6.24	6.59	6.8	5.63	6.96	0.47865	6.2759-6.6184
1990-2009	Cancer	[Drug - target median]	30	2	1.0078	0.3	1.075	1.39	-1.07	2.27	0.97438	0.65916-1.3565
1990-2009	Cardiovascular	Drug	37	4	7.8765	7.51	8.08	8.8275	6.45	9.87	1.3084	7.4549-8.2981
1990-2009	Cardiovascular	Target median	37	0	7.1535	6.725	7.3	7.88	5.35	8.1	0.82099	6.889-7.4181
1990-2009	Cardiovascular	[Drug - target median]	37	2	0.72297	-0.03	0.73	1.65	-1.57	3.805	1.3694	0.28173-1.1642
1990-2009	Central nervous system	Drug	57	0	7.9018	6.975	8.11	8.6525	4.89	10.86	1.3218	7.5586-8.2449
1990-2009	Central nervous system	Target median	57	8	6.9503	6.7	7.2	7.2125	6.05	7.97	0.68311	6.7729-7.1276
1990-2009	Central nervous system	[Drug - target median]	57	0	0.95149	0.22625	0.89	1.715	-2	3.34	1.1756	0.6463-1.2567
1990-2009	Gastrointestinal & metabolism	Drug	23	0	7.533	6.19	7.78	8.59	4.63	9.68	1.4509	6.9401-8.126
1990-2009	Gastrointestinal & metabolism	Target median	23	0	6.8898	6.46	7.09	7.8	5.255	8.005	0.90318	6.5207-7.2589
1990-2009	Gastrointestinal & metabolism	[Drug - target median]	23	2	0.64326	0.0125	0.57	1.2975	-0.53	1.84	1.1599	0.16923-1.1173
1990-2009	Genito urinary	Drug	18	0	8.4572	7.75	8.48	8.99	7.36	9.89	0.75494	8.1085-8.806
1990-2009	Genito urinary	Target median	18	0	7.2178	6.77	7.26	7.59	6.65	7.89	0.41672	7.0253-7.4103
1990-2009	Genito urinary	[Drug - target median]	18	0	1.2394	0.7	1.325	1.84	-0.49	2.35	0.80312	0.86842-1.6105
1990-2009	Infection	Drug	19	0	8.1405	7.09	8.7	9.075	5.96	10	1.264	7.5722-8.7089
1990-2009	Infection	Target median	19	0	6.9808	6.265	6.33	7.92	5.295	7.92	0.91066	6.5713-7.3903
1990-2009	Infection	[Drug - target median]	19	0	1.1597	0.8425	1.135	1.7175	-0.305	2.37	0.74956	0.82269-1.4968
1990-2009	Multiple/other	Drug	10	0	7.865	7.15	8.09	9	4.55	9.64	1.4847	6.9447-8.7853
1990-2009	Multiple/other	Target median	10	2	6.707	6.52	6.7	6.82	6.44	7.07	0.3616	6.4829-6.9311
1990-2009	Multiple/other	[Drug - target median]	10	1	1.158	0.72	1.52	2.18	-0.25	2.57	1.4209	0.27735-2.0387
1990-2009	Musculo-skeletal	Drug	18	3	6.9528	6.23	6.955	7.07	5.98	7.48	0.83831	6.5655-7.3401
1990-2009	Musculo-skeletal	Target median	18	6	5.9717	6.04	6.04	6.07	6.04	6.07	0.51373	5.7343-6.209
1990-2009	Musculo-skeletal	[Drug - target median]	18	1	0.98111	0.34	0.91	1.41	-1.14	2.92	1.1166	0.46527-1.497
1990-2009	Respiratory	Drug	20	2	8.235	7.59	8.44	8.715	5.96	9.71	1.2089	7.7052-8.7648
1990-2009	Respiratory	Target median	20	0	7.1285	6.82	6.82	7.68	5.96	8.23	0.64153	6.8473-7.4097
1990-2009	Respiratory	[Drug - target median]	20	0	1.1065	0.48	1.125	1.7	-1.24	3.11	1.007	0.66517-1.5478
1990-2009	Skin & eye	Drug	11	0	7.8055	6.79	8.32	8.5875	4.96	9.51	1.332	7.0183-8.5926
1990-2009	Skin & eye	Target median	11	0	6.5055	6.0825	6.33	7.05	5.49	7.37	0.61758	6.1405-6.8704
1990-2009	Skin & eye	[Drug - target median]	11	0	1.3	0.8875	1.12	2.2525	-1	3.3	1.1899	0.5968-2.0032
2010-2020	Cancer	Drug	61	3	8.4298	8.0075	8.44	8.7525	7.5	9.65	0.67685	8.26-8.5997
2010-2020	Cancer	Target median	61	1	7.1202	6.84	7.06	7.445	6.35	8.22	0.51019	6.9921-7.2482
2010-2020	Cancer	[Drug - target median]	61	1	1.3097	0.8575	1.26	1.7625	-0.35	2.95	0.81492	1.1052-1.5142
2010-2020	Cardiovascular	Drug	12	0	8.2667	7.785	8.475	8.84	6.68	9.3	0.77864	7.8261-8.7072
2010-2020	Cardiovascular	Target median	12	1	6.9892	6.795	7.14	7.255	6.44	7.34	0.38988	6.7686-7.2098
2010-2020	Cardiovascular	[Drug - target median]	12	0	1.2775	0.74	1.44	1.785	-0.46	2.91	0.91326	0.76078-1.7942
2010-2020	Central nervous system	Drug	13	0	8.3062	7.515	8.54	9.295	5.49	10.57	1.42	7.5342-9.0781
2010-2020	Central nervous system	Target median	13	0	6.9492	6.7125	6.89	7.435	5.69	7.82	0.6751	6.5822-7.3162
2010-2020	Central nervous system	[Drug - target median]	13	1	1.3569	0.795	1.51	2.215	0.4	2.75	1.1186	0.74884-1.965
2010-2020	Gastrointestinal & metabolism	Drug	22	0	8.4623	7.94	8.52	9	7	9.43	0.57583	8.2216-8.7029
2010-2020	Gastrointestinal & metabolism	Target median	22	4	7.3523	7.26	7.26	7.52	7.09	7.8	0.36043	7.2017-7.5029
2010-2020	Gastrointestinal & metabolism	[Drug - target median]	22	0	1.11	0.66	1.08	1.37	0.13	2.17	0.49712	0.90227-1.3177
2010-2020	Genito urinary	Drug	4	0	8.87	8.32	8.965	9.42	7.76	9.79	0.83885	8.0479-9.6921
2010-2020	Genito urinary	Target median	4	0	7.2463	7.0925	7.35	7.4	6.885	7.4	0.2454	7.0058-7.4867
2010-2020	Genito urinary	[Drug - target median]	4	0	1.6237	1.055	1.8225	2.1925	0.46	2.39	0.83266	0.80774-2.4398
2010-2020	Infection	Drug	8	0	8.815	7.925	8.925	9.55	7.55	10.17	0.94662	8.159-9.471
2010-2020	Infection	Target median	8	0	6.7994	5.96	6.37	7.7	5.295	9.04	1.2511	5.9324-7.6664
2010-2020	Infection	[Drug - target median]	8	0	2.0156	1.39	1.9275	2.465	1.13	3.43	0.77679	1.4773-2.5539
2010-2020	Multiple/other	Drug	11	0	8.1645	7.325	8.1	9.2225	6.52	9.8	1.0762	7.5286-8.8005
2010-2020	Multiple/other	Target median	11	0	7.1868	6.61	7.08	7.92	6.05	8.22	0.71295	6.7655-7.6081
2010-2020	Multiple/other	[Drug - target median]	11	0	0.97773	0.14375	1.32	1.72	-0.12	2	0.82393	0.49081-1.4646
2010-2020	Musculo-skeletal	Drug	3	0	6.78	5.3725	6.04	8.3725	5.15	9.15	2.1002	4.4034-9.1566
2010-2020	Musculo-skeletal	Target median	3	0	6.58	6.2325	6.81	6.87	6.04	6.89	0.46936	6.0489-7.1111
2010-2020	Musculo-skeletal	[Drug - target median]	3	0	0.2	-1.245	0	1.695	-1.66	2.26	1.9676	-2.0266-2.4266
2010-2020	Respiratory	Drug	7	0	8.0186	6.715	7.41	9.7175	5.59	10.22	1.7822	6.6983-9.3389
2010-2020	Respiratory	Target median	7	0	6.5793	5.68	6.57	7.5087	5.68	7.59	0.90733	5.9071-7.2514
2010-2020	Respiratory	[Drug - target median]	7	0	1.4393	0.8525	1.47	2.2088	-0.09	2.63	0.95678	0.7305-2.1481
