# Supplementary material for: Target-based evaluation of ‘drug-like’ properties and ligand efficiencies
Source: J Med Chem. Author manuscript; Available in PMC 2021 Jun 11. (PMC7610969; doi:10.1021/acs.jmedchem.1c00416)
Supplement: Supp drugs and compounds per target [file EMS123358-supplement-Supp_drugs_and_compounds_per_target.pdf]

TARGET (ChEMBL code_name_species)	TARGET_PREF_NAME	Target class	Number of drugs / target, all drug-target pairs	Number of drugs / target, primary targets	Number of compounds with pChEMBL values
72_Dopamine D2 receptor_Homo sapiens	Dopamine D2 receptor	Membrane receptor_GPCR_amine	36	16	6009
126_Cyclooxygenase-2_Homo sapiens	Cyclooxygenase-2	Enzyme_Oxidoreductase	26	15	3122
127_Histamine H1 receptor_Homo sapiens	Histamine H1 receptor	Membrane receptor_GPCR_amine	25	25	1072
100_Norepinephrine transporter_Homo sapiens	Norepinephrine transporter	Transporter	24	12	2947
129_Mu opioid receptor_Homo sapiens	Mu opioid receptor	Membrane receptor_GPCR_peptide	23	22	4167
121_Serotonin transporter_Homo sapiens	Serotonin transporter	Transporter	22	13	4301
43_Beta-2 adrenergic receptor_Homo sapiens	Beta-2 adrenergic receptor	Membrane receptor_GPCR_amine	22	18	1056
25_Glucocorticoid receptor_Homo sapiens	Glucocorticoid receptor	Transcription factor_NHR	21	19	2113
96_Cyclooxygenase-1_Homo sapiens	Cyclooxygenase-1	Enzyme_Oxidoreductase	21	16	1324
107_Serotonin 2a (5-HT2a) receptor_Homo sapiens	Serotonin 2a (5-HT2a) receptor	Membrane receptor_GPCR_amine	20	13	3893
130_Dopamine D3 receptor_Homo sapiens	Dopamine D3 receptor	Membrane receptor_GPCR_amine	17	8	4043
219_Muscarinic acetylcholine receptor M3_Homo sapiens	Muscarinic acetylcholine receptor M3	Membrane receptor_GPCR_amine	17	12	1500
50_Beta-1 adrenergic receptor_Homo sapiens	Beta-1 adrenergic receptor	Membrane receptor_GPCR_amine	16	8	866
218_Alpha-2c adrenergic receptor_Homo sapiens	Alpha-2c adrenergic receptor	Membrane receptor_GPCR_amine	15	2	608
52_Alpha-2a adrenergic receptor_Homo sapiens	Alpha-2a adrenergic receptor	Membrane receptor_GPCR_amine	14	10	810
103_Alpha-1d adrenergic receptor_Homo sapiens	Alpha-1d adrenergic receptor	Membrane receptor_GPCR_amine	12	3	1208
125_Alpha-1a adrenergic receptor_Homo sapiens	Alpha-1a adrenergic receptor	Membrane receptor_GPCR_amine	12	3	1415
61_Muscarinic acetylcholine receptor M1_Homo sapiens	Muscarinic acetylcholine receptor M1	Membrane receptor_GPCR_amine	12	11	1329
11140_Dipeptidyl peptidase IV_Homo sapiens	Dipeptidyl peptidase IV	Enzyme_Protease	11	11	3731
93_Acetylcholinesterase_Homo sapiens	Acetylcholinesterase	Enzyme_Hydrolase	11	8	3994
108_Serotonin 2c (5-HT2c) receptor_Homo sapiens	Serotonin 2c (5-HT2c) receptor	Membrane receptor_GPCR_amine	10	2	2763
10980-WT_Vascular endothelial growth factor receptor 2_Homo sapiens	Vascular endothelial growth factor receptor 2	Enzyme_Kinase	10	4	7468
137_Kappa opioid receptor_Homo sapiens	Kappa opioid receptor	Membrane receptor_GPCR_peptide	10	2	3560
155_Dopamine transporter_Homo sapiens	Dopamine transporter	Transporter	10	7	2607
19_Estrogen receptor alpha_Homo sapiens	Estrogen receptor alpha	Transcription factor_NHR	10	8	2491
216_Alpha-2b adrenergic receptor_Homo sapiens	Alpha-2b adrenergic receptor	Membrane receptor_GPCR_amine	10	0	504
90_Dopamine D4 receptor_Homo sapiens	Dopamine D4 receptor	Membrane receptor_GPCR_amine	10	1	2214
128_Alpha-1b adrenergic receptor_Homo sapiens	Alpha-1b adrenergic receptor	Membrane receptor_GPCR_amine	9	3	1233
11752-WT_Vascular endothelial growth factor receptor 3_Homo sapiens	Vascular endothelial growth factor receptor 3	Enzyme_Kinase	8	0	382
191_Human immunodeficiency virus type 1 protease_Human immunodeficiency virus 1	Human immunodeficiency virus type 1 protease	Enzyme_Protease	8	8	3951
56_Androgen Receptor_Homo sapiens	Androgen Receptor	Transcription factor_NHR	8	8	1756
10979-WT_Vascular endothelial growth factor receptor 1_Homo sapiens	Vascular endothelial growth factor receptor 1	Enzyme_Kinase	7	1	837
11480_Sodium channel protein type V alpha subunit_Homo sapiens	Sodium channel protein type V alpha subunit	Ion channel	7	3	572
115_Type-1 angiotensin II receptor_Homo sapiens	Type-1 angiotensin II receptor	Membrane receptor_GPCR_peptide	7	7	659
12670-WT_Tyrosine-protein kinase receptor FLT3_Homo sapiens	Tyrosine-protein kinase receptor FLT3	Enzyme_Kinase	7	3	1807
15_Carbonic anhydrase II_Homo sapiens	Carbonic anhydrase II	Enzyme_Other	7	4	5435
197-WT_Platelet-derived growth factor receptor beta_Homo sapiens	Platelet-derived growth factor receptor beta	Enzyme_Kinase	7	3	1163
228_Human immunodeficiency virus type 1 reverse transcriptase_Human immunodeficiency virus 1	Human immunodeficiency virus type 1 reverse transcriptase	Enzyme_Hydrolase	7	7	2036
238-WT_Stem cell growth factor receptor_Homo sapiens	Stem cell growth factor receptor	Enzyme_Kinase	7	1	1136
69_Angiotensin-converting enzyme_Homo sapiens	Angiotensin-converting enzyme	Enzyme_Protease	7	7	463
9-WT_Epidermal growth factor receptor erbB1_Homo sapiens	Epidermal growth factor receptor erbB1	Enzyme_Kinase	7	7	5409
100417-WT_ALK tyrosine kinase receptor_Homo sapiens	ALK tyrosine kinase receptor	Enzyme_Kinase	6	3	1550
104_Monoamine oxidase B_Homo sapiens	Monoamine oxidase B	Enzyme_Oxidoreductase	6	5	2910
105_Serotonin 1d (5-HT1d) receptor_Homo sapiens	Serotonin 1d (5-HT1d) receptor	Membrane receptor_GPCR_amine	6	5	1136
136_Delta opioid receptor_Homo sapiens	Delta opioid receptor	Membrane receptor_GPCR_peptide	6	0	3447
163_Peroxisome proliferator-activated receptor alpha_Homo sapiens	Peroxisome proliferator-activated receptor alpha	Transcription factor_NHR	6	5	1379
18061_Sodium channel protein type IX alpha subunit_Homo sapiens	Sodium channel protein type IX alpha subunit	Ion channel	6	4	5312
20092_Sodium/glucose cotransporter 2_Homo sapiens	Sodium/glucose cotransporter 2	Transporter	6	6	1288
24_HMG-CoA reductase_Homo sapiens	HMG-CoA reductase	Enzyme_Oxidoreductase	6	6	221
36_Progesterone receptor_Homo sapiens	Progesterone receptor	Transcription factor_NHR	6	6	1332
65_Cytochrome P450 19A1_Homo sapiens	Cytochrome P450 19A1	Enzyme_Other	6	6	1982
71_Farnesyl diphosphate synthase_Homo sapiens	Farnesyl diphosphate synthase	Enzyme_Transferase	6	6	217
104723_Adrenergic receptor alpha-1_Homo sapiens	Adrenergic receptor alpha-1	Membrane receptor_GPCR_amine	5	1	353
104766_Tubulin_Homo sapiens	Tubulin	Other	5	5	517
104811-WT_Bcr/Abl fusion protein_Homo sapiens	Bcr/Abl fusion protein	Enzyme_Kinase	5	1	375
106_Serotonin 1b (5-HT1b) receptor_Homo sapiens	Serotonin 1b (5-HT1b) receptor	Membrane receptor_GPCR_amine	5	0	942
10919-WT_Tyrosine-protein kinase JAK1_Homo sapiens	Tyrosine-protein kinase JAK1	Enzyme_Kinase	5	2	3955
10938-WT_Tyrosine-protein kinase JAK2_Homo sapiens	Tyrosine-protein kinase JAK2	Enzyme_Kinase	5	2	5009
11440_GABA receptor alpha-5 subunit_Homo sapiens	GABA receptor alpha-5 subunit	Ion channel	5	4	438
12896_Carbonic anhydrase IV_Homo sapiens	Carbonic anhydrase IV	Enzyme_Other	5	0	731
144_Serotonin 3a (5-HT3a) receptor_Homo sapiens	Serotonin 3a (5-HT3a) receptor	Ion channel	5	4	669
174_Estrogen receptor beta_Homo sapiens	Estrogen receptor beta	Transcription factor_NHR	5	3	1857
188-WT_Receptor protein-tyrosine kinase erbB-2_Homo sapiens	Receptor protein-tyrosine kinase erbB-2	Enzyme_Kinase	5	0	1687
252_Adenosine A2a receptor_Homo sapiens	Adenosine A2a receptor	Membrane receptor_GPCR_other	5	3	3990
3_Phosphodiesterase 5A_Homo sapiens	Phosphodiesterase 5A	Enzyme_Phosphodiesterase	5	4	1769
47_Muscarinic acetylcholine receptor M2_Homo sapiens	Muscarinic acetylcholine receptor M2	Membrane receptor_GPCR_amine	5	1	1368
86_Monoamine oxidase A_Homo sapiens	Monoamine oxidase A	Enzyme_Oxidoreductase	5	3	1998
8-WT_Tyrosine-protein kinase ABL_Homo sapiens	Tyrosine-protein kinase ABL	Enzyme_Kinase	5	2	1917
100079-WT_Tyrosine-protein kinase receptor RET_Homo sapiens	Tyrosine-protein kinase receptor RET	Enzyme_Kinase	4	2	571
100097-WT_Tyrosine-protein kinase BTK_Homo sapiens	Tyrosine-protein kinase BTK	Enzyme_Kinase	4	4	2128
100933_Poly [ADP-ribose] polymerase 2_Homo sapiens	Poly [ADP-ribose] polymerase 2	Enzyme_Transferase	4	2	257
10193_Carbonic anhydrase I_Homo sapiens	Carbonic anhydrase I	Enzyme_Other	4	0	4783
104688_GABA-A receptor; anion channel_Homo sapiens	GABA-A receptor; anion channel	Ion channel	4	3	355
104706_Glutamate [NMDA] receptor_Homo sapiens	Glutamate [NMDA] receptor	Ion channel	4	3	364
11206_Histone deacetylase 2_Homo sapiens	Histone deacetylase 2	Other	4	0	1172
11208_Histone deacetylase 3_Homo sapiens	Histone deacetylase 3	Other	4	0	1287
11307_Histone deacetylase 6_Homo sapiens	Histone deacetylase 6	Other	4	2	2473
11451-WT_Hepatocyte growth factor receptor_Homo sapiens	Hepatocyte growth factor receptor	Enzyme_Kinase	4	3	3022
11663_Poly [ADP-ribose] polymerase-1_Homo sapiens	Poly [ADP-ribose] polymerase-1	Enzyme_Transferase	4	2	3007
12226_Hepatitis C virus NS3 protease/helicase_Hepatitis C virus	Hepatitis C virus NS3 protease/helicase	Enzyme_Protease	4	2	526
12627-WT_Platelet-derived growth factor receptor alpha_Homo sapiens	Platelet-derived growth factor receptor alpha	Enzyme_Kinase	4	0	418
12697_Histone deacetylase 1_Homo sapiens	Histone deacetylase 1	Other	4	2	3935
12699-WT_Receptor protein-tyrosine kinase erbB-4_Homo sapiens	Receptor protein-tyrosine kinase erbB-4	Enzyme_Kinase	4	0	176
133_Peroxisome proliferator-activated receptor gamma_Homo sapiens	Peroxisome proliferator-activated receptor gamma	Transcription factor_NHR	4	3	2617
18072_Sodium channel protein type II alpha subunit_Homo sapiens	Sodium channel protein type II alpha subunit	Ion channel	4	3	119
194_Coagulation factor X_Homo sapiens	Coagulation factor X	Enzyme_Protease	4	4	5399
278_Adenosine A2b receptor_Homo sapiens	Adenosine A2b receptor	Membrane receptor_GPCR_other	4	1	1680
51_Serotonin 1a (5-HT1a) receptor_Homo sapiens	Serotonin 1a (5-HT1a) receptor	Membrane receptor_GPCR_amine	4	2	3774
57_Retinoic acid receptor alpha_Homo sapiens	Retinoic acid receptor alpha	Transcription factor_NHR	4	1	195
63_DNA topoisomerase II alpha_Homo sapiens	DNA topoisomerase II alpha	Enzyme_Other	4	2	211
87_Cannabinoid CB1 receptor_Homo sapiens	Cannabinoid CB1 receptor	Membrane receptor_GPCR_other	4	4	3647
88_Dopamine D1 receptor_Homo sapiens	Dopamine D1 receptor	Membrane receptor_GPCR_amine	4	1	1039
100126-V600E_Serine/threonine-protein kinase B-raf_Homo sapiens	Serine/threonine-protein kinase B-raf	Enzyme_Kinase	3	3	1471
102_Histamine H2 receptor_Homo sapiens	Histamine H2 receptor	Membrane receptor_GPCR_amine	3	3	399
10266-WT_Fibroblast growth factor receptor 1_Homo sapiens	Fibroblast growth factor receptor 1	Enzyme_Kinase	3	0	2139
102767_Solute carrier family 22 member 12_Homo sapiens	Solute carrier family 22 member 12	Transporter	3	3	363
10434-WT_Tyrosine-protein kinase SRC_Homo sapiens	Tyrosine-protein kinase SRC	Enzyme_Kinase	3	0	2944
104679_Phosphodiesterase 4_Homo sapiens	Phosphodiesterase 4	Enzyme_Phosphodiesterase	3	0	1494
104733_Melatonin receptor_Homo sapiens	Melatonin receptor	Membrane receptor_GPCR_other	3	1	147
104743_Adrenergic receptor alpha-2_Homo sapiens	Adrenergic receptor alpha-2	Membrane receptor_GPCR_amine	3	1	199
10480-WT_Cyclin-dependent kinase 6_Homo sapiens	Cyclin-dependent kinase 6	Enzyme_Kinase	3	0	330
10599_Phosphodiesterase 4B_Homo sapiens	Phosphodiesterase 4B	Enzyme_Phosphodiesterase	3	1	1383
10738_Neuraminidase_Influenza B virus (strain B/Lee/1940)	Neuraminidase	Enzyme_Hydrolase	3	0	194
10869_Histone deacetylase 8_Homo sapiens	Histone deacetylase 8	Other	3	0	1307
10945_Histone deacetylase 10_Homo sapiens	Histone deacetylase 10	Other	3	0	271
11_Thrombin_Homo sapiens	Thrombin	Enzyme_Protease	3	3	4494
11084_Melatonin receptor 1A_Homo sapiens	Melatonin receptor 1A	Membrane receptor_GPCR_other	3	2	981
11085_Melatonin receptor 1B_Homo sapiens	Melatonin receptor 1B	Membrane receptor_GPCR_other	3	0	866
112_Vasopressin V2 receptor_Homo sapiens	Vasopressin V2 receptor	Membrane receptor_GPCR_peptide	3	3	465
11232_Histone deacetylase 4_Homo sapiens	Histone deacetylase 4	Other	3	0	576
114_Adenosine A1 receptor_Homo sapiens	Adenosine A1 receptor	Membrane receptor_GPCR_other	3	0	3789
11409-WT_Dual specificity mitogen-activated protein kinase kinase 1_Homo sapiens	Dual specificity mitogen-activated protein kinase kinase 1	Enzyme_Kinase	3	3	696
11540_Cystic fibrosis transmembrane conductance regulator_Homo sapiens	Cystic fibrosis transmembrane conductance regulator	Ion channel	3	3	343
11631_Sphingosine 1-phosphate receptor Edg-1_Homo sapiens	Sphingosine 1-phosphate receptor Edg-1	Membrane receptor_GPCR_other	3	3	1583
11926-WT_Fibroblast growth factor receptor 3_Homo sapiens	Fibroblast growth factor receptor 3	Enzyme_Kinase	3	0	1317
12021_Histone deacetylase 11_Homo sapiens	Histone deacetylase 11	Other	3	0	219
12209_Carbonic anhydrase XII_Homo sapiens	Carbonic anhydrase XII	Enzyme_Other	3	1	2760
12443_Histone deacetylase 7_Homo sapiens	Histone deacetylase 7	Other	3	0	182
12840-WT_Macrophage colony stimulating factor receptor_Homo sapiens	Macrophage colony stimulating factor receptor	Enzyme_Kinase	3	1	1046
12946-WT_Cyclin-dependent kinase 4_Homo sapiens	Cyclin-dependent kinase 4	Enzyme_Kinase	3	2	1011
165_HERG_Homo sapiens	HERG	Ion channel	3	3	7039
168_Serotonin 4 (5-HT4) receptor_Homo sapiens	Serotonin 4 (5-HT4) receptor	Membrane receptor_GPCR_amine	3	3	519
179_Cysteinyl leukotriene receptor 1_Homo sapiens	Cysteinyl leukotriene receptor 1	Membrane receptor_GPCR_other	3	3	111
21_Neuraminidase_Influenza A virus (A/Puerto Rico/8/1934(H1N1))	Neuraminidase	Enzyme_Hydrolase	3	3	320
226_Beta-3 adrenergic receptor_Homo sapiens	Beta-3 adrenergic receptor	Membrane receptor_GPCR_amine	3	1	452
240_Retinoic acid receptor gamma_Homo sapiens	Retinoic acid receptor gamma	Transcription factor_NHR	3	1	242
249_Endothelin receptor ET-A_Homo sapiens	Endothelin receptor ET-A	Membrane receptor_GPCR_peptide	3	3	1287
250_Neurokinin 1 receptor_Homo sapiens	Neurokinin 1 receptor	Membrane receptor_GPCR_peptide	3	3	2372
270_Retinoic acid receptor beta_Homo sapiens	Retinoic acid receptor beta	Transcription factor_NHR	3	2	225
280_Adenosine A3 receptor_Homo sapiens	Adenosine A3 receptor	Membrane receptor_GPCR_other	3	2	3689
64_Vitamin D receptor_Homo sapiens	Vitamin D receptor	Transcription factor_NHR	3	3	354
1_Maltase-glucoamylase_Homo sapiens	Maltase-glucoamylase	Enzyme_Hydrolase	2	2	127
100126-WT_Serine/threonine-protein kinase B-raf_Homo sapiens	Serine/threonine-protein kinase B-raf	Enzyme_Kinase	2	0	2431
100413-WT_Neurotrophic tyrosine kinase receptor type 2_Homo sapiens	Neurotrophic tyrosine kinase receptor type 2	Enzyme_Kinase	2	0	177
101400_Smoothened homolog_Homo sapiens	Smoothened homolog	Membrane receptor_GPCR_peptide	2	2	568
10140-WT_Tyrosine-protein kinase LCK_Homo sapiens	Tyrosine-protein kinase LCK	Enzyme_Kinase	2	0	1587
101608_Toll-like receptor 7_Homo sapiens	Toll-like receptor 7	Other	2	1	243
10264-WT_Fibroblast growth factor receptor 2_Homo sapiens	Fibroblast growth factor receptor 2	Enzyme_Kinase	2	1	968
104283_Neuronal acetylcholine receptor; alpha4/beta2_Homo sapiens	Neuronal acetylcholine receptor; alpha4/beta2	Ion channel	2	2	702
104681_Histone deacetylase_Homo sapiens	Histone deacetylase	Other	2	0	1883
104727_DNA topoisomerase II_Homo sapiens	DNA topoisomerase II	Enzyme_Other	2	2	159
104758_Potassium-transporting ATPase_Homo sapiens	Potassium-transporting ATPase	Transporter	2	2	178
104770_Sodium/potassium-transporting ATPase_Homo sapiens	Sodium/potassium-transporting ATPase	Transporter	2	2	232
104803_Hepatitis C virus serine protease, NS3/NS4A_Hepatitis C virus	Hepatitis C virus serine protease, NS3/NS4A	Enzyme_Protease	2	2	606
10532_Butyrylcholinesterase_Homo sapiens	Butyrylcholinesterase	Enzyme_Hydrolase	2	2	2121
10849-WT_Tyrosine-protein kinase JAK3_Homo sapiens	Tyrosine-protein kinase JAK3	Enzyme_Kinase	2	2	2731
11060_Carbonic anhydrase VII_Homo sapiens	Carbonic anhydrase VII	Enzyme_Other	2	2	746
11177_PI3-kinase p110-delta subunit_Homo sapiens	PI3-kinase p110-delta subunit	Enzyme_Transferase	2	1	2280
11180_Bile acid receptor FXR_Homo sapiens	Bile acid receptor FXR	Transcription factor_NHR	2	2	746
11272_Sigma opioid receptor_Homo sapiens	Sigma opioid receptor	Other	2	2	2201
11359_Phosphodiesterase 4D_Homo sapiens	Phosphodiesterase 4D	Enzyme_Phosphodiesterase	2	1	800
11362_PI3-kinase p110-alpha subunit_Homo sapiens	PI3-kinase p110-alpha subunit	Enzyme_Transferase	2	2	5069
116_Oxytocin receptor_Homo sapiens	Oxytocin receptor	Membrane receptor_GPCR_peptide	2	2	451
11684_Nischarin_Homo sapiens	Nischarin	Other	2	2	171
11785-WT_Tyrosine-protein kinase HCK_Homo sapiens	Tyrosine-protein kinase HCK	Enzyme_Kinase	2	0	414
11797_Histone deacetylase 9_Homo sapiens	Histone deacetylase 9	Other	2	0	161
118_Gonadotropin-releasing hormone receptor_Homo sapiens	Gonadotropin-releasing hormone receptor	Membrane receptor_GPCR_peptide	2	2	1121
11842-WT_Tyrosine-protein kinase Lyn_Homo sapiens	Tyrosine-protein kinase Lyn	Enzyme_Kinase	2	0	283
11902-WT_Nerve growth factor receptor Trk-A_Homo sapiens	Nerve growth factor receptor Trk-A	Enzyme_Kinase	2	2	2522
11925-WT_Fibroblast growth factor receptor 4_Homo sapiens	Fibroblast growth factor receptor 4	Enzyme_Kinase	2	0	869
12_Plasminogen_Homo sapiens	Plasminogen	Enzyme_Protease	2	2	828
12283_Histone deacetylase 5_Homo sapiens	Histone deacetylase 5	Other	2	0	158
12456_Human immunodeficiency virus type 1 integrase_Human immunodeficiency virus 1	Human immunodeficiency virus type 1 integrase	Enzyme_Hydrolase	2	2	882
12694-WT_Tyrosine-protein kinase TYK2_Homo sapiens	Tyrosine-protein kinase TYK2	Enzyme_Kinase	2	0	1102
140_Prostanoid FP receptor_Homo sapiens	Prostanoid FP receptor	Membrane receptor_GPCR_other	2	2	109
143_Prostanoid IP receptor_Homo sapiens	Prostanoid IP receptor	Membrane receptor_GPCR_other	2	2	358
149_Xanthine dehydrogenase_Homo sapiens	Xanthine dehydrogenase	Enzyme_Oxidoreductase	2	2	287
169_Voltage-gated L-type calcium channel alpha-1C subunit_Homo sapiens	Voltage-gated L-type calcium channel alpha-1C subunit	Ion channel	2	2	137
176_Purinergic receptor P2Y12_Homo sapiens	Purinergic receptor P2Y12	Membrane receptor_GPCR_other	2	2	952
18080_Sodium channel protein type X alpha subunit_Homo sapiens	Sodium channel protein type X alpha subunit	Ion channel	2	0	182
227_Serotonin 2b (5-HT2b) receptor_Homo sapiens	Serotonin 2b (5-HT2b) receptor	Membrane receptor_GPCR_amine	2	1	1210
242_Aldose reductase_Homo sapiens	Aldose reductase	Enzyme_Oxidoreductase	2	2	709
246_Thromboxane A2 receptor_Homo sapiens	Thromboxane A2 receptor	Membrane receptor_GPCR_other	2	2	599
247_Endothelin receptor ET-B_Homo sapiens	Endothelin receptor ET-B	Membrane receptor_GPCR_peptide	2	0	927
266_Retinoid X receptor gamma_Homo sapiens	Retinoid X receptor gamma	Transcription factor_NHR	2	0	130
275_Retinoid X receptor alpha_Homo sapiens	Retinoid X receptor alpha	Transcription factor_NHR	2	0	417
276_Phosphodiesterase 4A_Homo sapiens	Phosphodiesterase 4A	Enzyme_Phosphodiesterase	2	1	626
277_Retinoid X receptor beta_Homo sapiens	Retinoid X receptor beta	Transcription factor_NHR	2	1	127
28_Thymidylate synthase_Homo sapiens	Thymidylate synthase	Enzyme_Transferase	2	1	547
286_Ceramide glucosyltransferase_Homo sapiens	Ceramide glucosyltransferase	Enzyme_Transferase	2	2	128
30008-WT_Ephrin type-A receptor 2_Homo sapiens	Ephrin type-A receptor 2	Enzyme_Kinase	2	0	373
48_Mineralocorticoid receptor_Homo sapiens	Mineralocorticoid receptor	Transcription factor_NHR	2	2	690
6_Dihydrofolate reductase_Homo sapiens	Dihydrofolate reductase	Enzyme_Oxidoreductase	2	2	899
62_DNA topoisomerase I_Homo sapiens	DNA topoisomerase I	Enzyme_Other	2	2	303
70_Dihydrofolate reductase_Plasmodium falciparum K1	Dihydrofolate reductase	Enzyme_Oxidoreductase	2	2	169
80_FK506-binding protein 1A_Homo sapiens	FK506-binding protein 1A	Enzyme_Other	2	2	550
100010_Phosphodiesterase 10A_Homo sapiens	Phosphodiesterase 10A	Enzyme_Phosphodiesterase	1	0	4347
10009_Orexin receptor 1_Homo sapiens	Orexin receptor 1	Membrane receptor_GPCR_peptide	1	1	2699
100100_Voltage-gated T-type calcium channel alpha-1G subunit_Homo sapiens	Voltage-gated T-type calcium channel alpha-1G subunit	Ion channel	1	0	452
100101_Voltage-gated T-type calcium channel alpha-1I subunit_Homo sapiens	Voltage-gated T-type calcium channel alpha-1I subunit	Ion channel	1	1	191
100129-WT_Ephrin type-B receptor 4_Homo sapiens	Ephrin type-B receptor 4	Enzyme_Kinase	1	0	717
100304_Apoptosis regulator Bcl-2_Homo sapiens	Apoptosis regulator Bcl-2	Ion channel	1	1	1591
100624_Proteasome Macropain subunit MB1_Homo sapiens	Proteasome Macropain subunit MB1	Enzyme_Protease	1	1	396
101019-WT_Proto-oncogene tyrosine-protein kinase ROS_Homo sapiens	Proto-oncogene tyrosine-protein kinase ROS	Enzyme_Kinase	1	0	143
101287_Tryptophan 5-hydroxylase 1_Homo sapiens	Tryptophan 5-hydroxylase 1	Enzyme_Oxidoreductase	1	1	192
10139_Carbonic anhydrase III_Homo sapiens	Carbonic anhydrase III	Enzyme_Other	1	0	134
101412_Transient receptor potential cation channel subfamily A member 1_Homo sapiens	Transient receptor potential cation channel subfamily A member 1	Ion channel	1	1	419
101567_Toll-like receptor 9_Homo sapiens	Toll-like receptor 9	Other	1	1	325
10186_Carbonic anhydrase VI_Homo sapiens	Carbonic anhydrase VI	Enzyme_Other	1	0	276
10209_Serotonin 7 (5-HT7) receptor_Homo sapiens	Serotonin 7 (5-HT7) receptor	Membrane receptor_GPCR_amine	1	0	1966
102438_Neuraminidase_Influenza A virus	Neuraminidase	Enzyme_Hydrolase	1	1	534
10260_Vanilloid receptor_Homo sapiens	Vanilloid receptor	Ion channel	1	1	2108
10280_Histamine H3 receptor_Homo sapiens	Histamine H3 receptor	Membrane receptor_GPCR_amine	1	1	3216
103167_Transient receptor potential cation channel subfamily M member 8_Homo sapiens	Transient receptor potential cation channel subfamily M member 8	Ion channel	1	0	498
10329_Prostanoid EP3 receptor_Homo sapiens	Prostanoid EP3 receptor	Membrane receptor_GPCR_other	1	0	565
104288_Neuronal acetylcholine receptor; alpha3/beta4_Homo sapiens	Neuronal acetylcholine receptor; alpha3/beta4	Ion channel	1	1	368
104295-WT_Cyclin-dependent kinase 4/cyclin D1_Homo sapiens	Cyclin-dependent kinase 4/cyclin D1	Enzyme_Kinase	1	1	1154
104483-R132C-H_Isocitrate dehydrogenase [NADP] cytoplasmic_Homo sapiens	Isocitrate dehydrogenase [NADP] cytoplasmic	Enzyme_Oxidoreductase	1	1	388
10460_Dihydrofolate reductase_Pneumocystis carinii	Dihydrofolate reductase	Enzyme_Oxidoreductase	1	1	584
104685_Integrin alpha-IIb/beta-3_Homo sapiens	Integrin alpha-IIb/beta-3	Other	1	1	1303
104707_Phosphodiesterase 3_Homo sapiens	Phosphodiesterase 3	Enzyme_Phosphodiesterase	1	1	714
10473_C-X-C chemokine receptor type 4_Homo sapiens	C-X-C chemokine receptor type 4	Membrane receptor_GPCR_peptide	1	1	421
104773-WT_Platelet-derived growth factor receptor_Homo sapiens	Platelet-derived growth factor receptor	Enzyme_Kinase	1	0	266
10580_C-C chemokine receptor type 5_Homo sapiens	C-C chemokine receptor type 5	Membrane receptor_GPCR_peptide	1	1	1706
10594_Phosphodiesterase 2A_Homo sapiens	Phosphodiesterase 2A	Enzyme_Phosphodiesterase	1	0	788
10653_Proteinase-activated receptor 1_Homo sapiens	Proteinase-activated receptor 1	Membrane receptor_GPCR_peptide	1	1	771
10765_Carnitine palmitoyltransferase 2_Homo sapiens	Carnitine palmitoyltransferase 2	Enzyme_Transferase	1	1	268
108438_Nonstructural protein 5A_Hepatitis C virus	Nonstructural protein 5A	Other	1	1	138
10878-WT_Protein kinase C epsilon_Homo sapiens	Protein kinase C epsilon	Enzyme_Kinase	1	0	421
10904-WT_Serine/threonine-protein kinase RAF_Homo sapiens	Serine/threonine-protein kinase RAF	Enzyme_Kinase	1	0	1057
10906-WT_Tyrosine-protein kinase SYK_Homo sapiens	Tyrosine-protein kinase SYK	Enzyme_Kinase	1	1	3155
10950_Sphingosine 1-phosphate receptor Edg-3_Homo sapiens	Sphingosine 1-phosphate receptor Edg-3	Membrane receptor_GPCR_other	1	0	711
110_Enoyl-[acyl-carrier-protein] reductase_Escherichia coli K-12	Enoyl-[acyl-carrier-protein] reductase	Enzyme_Oxidoreductase	1	1	103
11016_Carbonic anhydrase VB_Homo sapiens	Carbonic anhydrase VB	Enzyme_Other	1	0	262
11024_Matrix metalloproteinase 13_Homo sapiens	Matrix metalloproteinase 13	Enzyme_Protease	1	0	2497
11042_Carbonic anhydrase VA_Homo sapiens	Carbonic anhydrase VA	Enzyme_Other	1	0	322
11063_Carbonic anhydrase XIV_Homo sapiens	Carbonic anhydrase XIV	Enzyme_Other	1	0	575
11110_Matrix metalloproteinase 8_Homo sapiens	Matrix metalloproteinase 8	Enzyme_Protease	1	1	1130
11225_Renin_Homo sapiens	Renin	Enzyme_Protease	1	1	2810
11396_Phosphodiesterase 4C_Homo sapiens	Phosphodiesterase 4C	Enzyme_Phosphodiesterase	1	0	111
11489_11-beta-hydroxysteroid dehydrogenase 1_Homo sapiens	11-beta-hydroxysteroid dehydrogenase 1	Enzyme_Oxidoreductase	1	1	3220
11536_Ghrelin receptor_Homo sapiens	Ghrelin receptor	Membrane receptor_GPCR_peptide	1	1	1101
11617-WT_Protein kinase C eta_Homo sapiens	Protein kinase C eta	Enzyme_Kinase	1	0	225
11635-WT_Protein kinase C alpha_Homo sapiens	Protein kinase C alpha	Enzyme_Kinase	1	0	1052
11691-WT_Tyrosine-protein kinase BLK_Homo sapiens	Tyrosine-protein kinase BLK	Enzyme_Kinase	1	1	105
11723_Thymidine phosphorylase_Homo sapiens	Thymidine phosphorylase	Enzyme_Other	1	1	171
11755-WT_Tyrosine-protein kinase FYN_Homo sapiens	Tyrosine-protein kinase FYN	Enzyme_Kinase	1	0	261
11939_PI3-kinase p110-gamma subunit_Homo sapiens	PI3-kinase p110-gamma subunit	Enzyme_Transferase	1	1	2061
120_Steroid 5-alpha-reductase 2_Homo sapiens	Steroid 5-alpha-reductase 2	Enzyme_Oxidoreductase	1	1	393
12227_Peroxisome proliferator-activated receptor delta_Homo sapiens	Peroxisome proliferator-activated receptor delta	Transcription factor_NHR	1	1	736
12247-WT_Protein kinase C delta_Homo sapiens	Protein kinase C delta	Enzyme_Kinase	1	0	620
12665-WT_Protein kinase C theta_Homo sapiens	Protein kinase C theta	Enzyme_Kinase	1	0	746
12704_Transthyretin_Homo sapiens	Transthyretin	Other	1	1	101
12913-WT_Tyrosine-protein kinase TIE-2_Homo sapiens	Tyrosine-protein kinase TIE-2	Enzyme_Kinase	1	0	790
12923_Carbonic anhydrase XIII_Homo sapiens	Carbonic anhydrase XIII	Enzyme_Other	1	0	226
12949_Cytochrome P450 17A1_Homo sapiens	Cytochrome P450 17A1	Enzyme_Other	1	1	667
12952_Carbonic anhydrase IX_Homo sapiens	Carbonic anhydrase IX	Enzyme_Other	1	0	3507
12968_Orexin receptor 2_Homo sapiens	Orexin receptor 2	Membrane receptor_GPCR_peptide	1	0	2892
134_Vasopressin V1a receptor_Homo sapiens	Vasopressin V1a receptor	Membrane receptor_GPCR_peptide	1	0	611
142_Prostanoid EP2 receptor_Homo sapiens	Prostanoid EP2 receptor	Membrane receptor_GPCR_other	1	0	488
150_Voltage-gated calcium channel alpha2/delta subunit 1_Homo sapiens	Voltage-gated calcium channel alpha2/delta subunit 1	Other	1	1	151
157_Dihydroorotate dehydrogenase_Homo sapiens	Dihydroorotate dehydrogenase	Enzyme_Oxidoreductase	1	1	760
170_Phosphodiesterase 3A_Homo sapiens	Phosphodiesterase 3A	Enzyme_Phosphodiesterase	1	1	291
175_Equilibrative nucleoside transporter 1_Homo sapiens	Equilibrative nucleoside transporter 1	Transporter	1	1	237
18033-WT_Tyrosine-protein kinase YES_Homo sapiens	Tyrosine-protein kinase YES	Enzyme_Kinase	1	0	154
19904_Calcitonin gene-related peptide type 1 receptor_Homo sapiens	Calcitonin gene-related peptide type 1 receptor	Membrane receptor_GPCR_peptide	1	1	585
20174_G protein-coupled receptor 44_Homo sapiens	G protein-coupled receptor 44	Membrane receptor_GPCR_other	1	0	2638
248_Thromboxane-A synthase_Homo sapiens	Thromboxane-A synthase	Enzyme_Oxidoreductase	1	1	741
251_Platelet activating factor receptor_Homo sapiens	Platelet activating factor receptor	Membrane receptor_GPCR_peptide	1	0	379
261_Prostanoid EP1 receptor_Homo sapiens	Prostanoid EP1 receptor	Membrane receptor_GPCR_other	1	0	644
262_Prostanoid EP4 receptor_Homo sapiens	Prostanoid EP4 receptor	Membrane receptor_GPCR_other	1	1	590
271_Thyroid hormone receptor beta-1_Homo sapiens	Thyroid hormone receptor beta-1	Transcription factor_NHR	1	1	485
282_Calcium sensing receptor_Homo sapiens	Calcium sensing receptor	Membrane receptor_GPCR_other	1	1	304
30026-WT_Tyrosine-protein kinase BRK_Homo sapiens	Tyrosine-protein kinase BRK	Enzyme_Kinase	1	0	157
4_Voltage-gated T-type calcium channel alpha-1H subunit_Homo sapiens	Voltage-gated T-type calcium channel alpha-1H subunit	Ion channel	1	0	345
55_Arachidonate 5-lipoxygenase_Homo sapiens	Arachidonate 5-lipoxygenase	Enzyme_Oxidoreductase	1	1	1428
59_Thyroid hormone receptor alpha_Homo sapiens	Thyroid hormone receptor alpha	Transcription factor_NHR	1	0	350
68_Inosine-5'-monophosphate dehydrogenase 2_Homo sapiens	Inosine-5'-monophosphate dehydrogenase 2	Enzyme_Oxidoreductase	1	1	598
76_Cytochrome P450 11B1_Homo sapiens	Cytochrome P450 11B1	Enzyme_Oxidoreductase	1	1	972
84_Inosine-5'-monophosphate dehydrogenase 1_Homo sapiens	Inosine-5'-monophosphate dehydrogenase 1	Enzyme_Oxidoreductase	1	0	160
9-T790M_Epidermal growth factor receptor erbB1_Homo sapiens	Epidermal growth factor receptor erbB1	Enzyme_Kinase	1	1	332
			1104	643	360049
