# Supplementary material for: Target-based evaluation of ‘drug-like’ properties and ligand efficiencies
Source: J Med Chem. Author manuscript; Available in PMC 2021 Jun 11. (PMC7610969; doi:10.1021/acs.jmedchem.1c00416)
Supplement: Supp Fig S4 values QED [file EMS123358-supplement-Supp_Fig_S4_values_QED.pdf]

QED

Approval period	Target class	Group	Total Count	Outlier Count	Mean Value	1st Quartile	Median	3rd Quartile	Lower Adjacent Limit	Upper Adjacent Limit	Standard Deviation	Confidence Interval (95%)
1939-1989	Enzyme_Hydrolase	Drug	7	0	0.57857	0.3425	0.63	0.805	0.13	0.92	0.28985	0.36385-0.7933
1939-1989	Enzyme_Hydrolase	Target median	7	1	0.52286	0.5	0.5	0.5	0.5	0.5	0.060474	0.47806-0.56766
1939-1989	Enzyme_Hydrolase	[Drug - target median]	7	0	0.055714	-0.1575	0.13	0.305	-0.53	0.42	0.33406	-0.19176-0.30319
1939-1989	Enzyme_Other	Drug	32	1	0.70687	0.63	0.73	0.9	0.24	0.9	0.20813	0.63476-0.77899
1939-1989	Enzyme_Other	Target median	32	9	0.55062	0.54	0.56	0.59	0.54	0.66	0.079116	0.52321-0.57804
1939-1989	Enzyme_Other	[Drug - target median]	32	0	0.15625	0.045	0.16	0.295	-0.25	0.36	0.16226	0.10003-0.21247
1939-1989	Enzyme_Oxidoreductase	Drug	46	2	0.76087	0.67	0.85	0.86	0.45	0.89	0.15659	0.71562-0.80612
1939-1989	Enzyme_Oxidoreductase	Target median	46	2	0.67935	0.66	0.68	0.72	0.61	0.72	0.044542	0.66648-0.69222
1939-1989	Enzyme_Oxidoreductase	[Drug - target median]	46	2	0.081522	-0.01	0.14	0.19	-0.21	0.3	0.15352	0.037156-0.12589
1939-1989	Enzyme_Phosphodiesterase	Drug	9	0	0.59	0.31	0.76	0.76	0.31	0.78	0.22017	0.44616-0.73384
1939-1989	Enzyme_Phosphodiesterase	Target median	9	0	0.53889	0.5075	0.53	0.5925	0.42	0.66	0.07524	0.48973-0.58805
1939-1989	Enzyme_Phosphodiesterase	[Drug - target median]	9	0	0.051111	-0.22	0.12	0.2525	-0.24	0.34	0.2363	-0.10327-0.20549
1939-1989	Enzyme_Protease	Drug	7	0	0.50429	0.3425	0.54	0.6425	0.33	0.68	0.15437	0.38993-0.61864
1939-1989	Enzyme_Protease	Target median	7	0	0.42	0.2475	0.42	0.58	0.21	0.58	0.16763	0.29582-0.54418
1939-1989	Enzyme_Protease	[Drug - target median]	7	0	0.084286	-0.075	0.07	0.2725	-0.2	0.41	0.22029	-0.078909-0.24748
1939-1989	Ionchannel	Drug	24	1	0.68292	0.63	0.72	0.79	0.42	0.88	0.16449	0.61711-0.74873
1939-1989	Ionchannel	Target median	24	0	0.60312	0.57	0.61	0.66	0.48	0.75	0.074335	0.57338-0.63287
1939-1989	Ionchannel	[Drug - target median]	24	0	0.079792	-0.04	0.0825	0.215	-0.38	0.37	0.17407	0.010148-0.14943
1939-1989	Membranereceptor_GPCR_amine	Drug	180	1	0.68889	0.59	0.715	0.81	0.29	0.92	0.14994	0.66698-0.71079
1939-1989	Membranereceptor_GPCR_amine	Target median	180	31	0.57278	0.54	0.62	0.65	0.46	0.72	0.13548	0.55299-0.59257
1939-1989	Membranereceptor_GPCR_amine	[Drug - target median]	180	1	0.11611	0	0.14	0.225	-0.31	0.56	0.18889	0.088517-0.14371
1939-1989	Membranereceptor_GPCR_other	Drug	23	9	0.54696	0.49	0.54	0.5675	0.49	0.6	0.14849	0.48627-0.60764
1939-1989	Membranereceptor_GPCR_other	Target median	23	6	0.52435	0.48	0.5	0.54	0.42	0.54	0.13494	0.4692-0.57949
1939-1989	Membranereceptor_GPCR_other	[Drug - target median]	23	1	0.022609	-0.0175	0.02	0.055	-0.1	0.12	0.071109	-0.0064528-0.05167
1939-1989	Membranereceptor_GPCR_peptide	Drug	31	3	0.73065	0.74	0.79	0.8	0.69	0.88	0.19442	0.6622-0.79909
1939-1989	Membranereceptor_GPCR_peptide	Target median	31	2	0.58516	0.6	0.6	0.61125	0.59	0.615	0.075801	0.55848-0.61185
1939-1989	Membranereceptor_GPCR_peptide	[Drug - target median]	31	3	0.14548	0.13625	0.185	0.21	0.075	0.28	0.13738	0.097124-0.19384
1939-1989	Other	Drug	7	1	0.63571	0.545	0.73	0.7675	0.49	0.83	0.22759	0.46712-0.80431
1939-1989	Other	Target median	7	0	0.64286	0.57	0.61	0.73	0.54	0.75	0.090317	0.57595-0.70976
1939-1989	Other	[Drug - target median]	7	0	-0.00714	-0.19	0.05	0.1725	-0.39	0.26	0.23507	-0.18128-0.167
1939-1989	Transcriptionfactor_NHR	Drug	46	2	0.66935	0.62	0.69	0.74	0.5	0.86	0.10449	0.63915-0.69954
1939-1989	Transcriptionfactor_NHR	Target median	46	19	0.52196	0.52	0.52	0.55	0.49	0.55	0.092247	0.4953-0.54861
1939-1989	Transcriptionfactor_NHR	[Drug - target median]	46	0	0.14739	0.02	0.15	0.24	-0.09	0.48	0.15426	0.10281-0.19197
1939-1989	Transporter	Drug	35	5	0.73829	0.74	0.81	0.83	0.65	0.87	0.17837	0.67919-0.79738
1939-1989	Transporter	Target median	35	3	0.74543	0.75	0.78	0.81	0.68	0.81	0.1188	0.70607-0.78479
1939-1989	Transporter	[Drug - target median]	35	2	-0.00714	-0.04	0.01	0.055	-0.16	0.12	0.088036	-0.036309-0.022024
1990-2009	Enzyme_Hydrolase	Drug	19	0	0.54789	0.315	0.67	0.745	0.18	0.86	0.24346	0.43842-0.65737
1990-2009	Enzyme_Hydrolase	Target median	19	0	0.52789	0.4625	0.51	0.66	0.32	0.66	0.10881	0.47897-0.57682
1990-2009	Enzyme_Hydrolase	[Drug - target median]	19	0	0.02	-0.28	0.07	0.24	-0.41	0.37	0.26272	-0.098134-0.13813
1990-2009	Enzyme_Kinase	Drug	51	18	0.47804	0.46	0.47	0.49	0.42	0.52	0.10358	0.44961-0.50647
1990-2009	Enzyme_Kinase	Target median	51	6	0.43971	0.4125	0.44	0.45	0.36	0.47	0.045193	0.4273-0.45211
1990-2009	Enzyme_Kinase	[Drug - target median]	51	3	0.038333	-0.005	0.05	0.0975	-0.15	0.22	0.10898	0.008422-0.068245
1990-2009	Enzyme_Other	Drug	12	0	0.6125	0.5	0.71	0.745	0.16	0.87	0.23607	0.47893-0.74607
1990-2009	Enzyme_Other	Target median	12	0	0.5275	0.37	0.55	0.67	0.35	0.67	0.13363	0.45189-0.60311
1990-2009	Enzyme_Other	[Drug - target median]	12	2	0.085	0.04	0.115	0.185	0.01	0.3	0.15276	-0.0014335-0.17143
1990-2009	Enzyme_Oxidoreductase	Drug	32	2	0.68375	0.615	0.72	0.825	0.4	0.91	0.18732	0.61885-0.74865
1990-2009	Enzyme_Oxidoreductase	Target median	32	0	0.58344	0.495	0.66	0.665	0.27	0.72	0.14314	0.53384-0.63303
1990-2009	Enzyme_Oxidoreductase	[Drug - target median]	32	4	0.10031	0.045	0.11	0.16	-0.05	0.25	0.1378	0.052566-0.14806
1990-2009	Enzyme_Phosphodiesterase	Drug	4	0	0.6325	0.535	0.62	0.73	0.52	0.77	0.11786	0.51699-0.74801
1990-2009	Enzyme_Phosphodiesterase	Target median	4	0	0.56	0.53	0.53	0.59	0.53	0.65	0.06	0.5012-0.6188
1990-2009	Enzyme_Phosphodiesterase	[Drug - target median]	4	0	0.0725	0.005	0.07	0.14	-0.01	0.16	0.080571	-0.0064595-0.15146
1990-2009	Enzyme_Protease	Drug	19	0	0.38316	0.1925	0.33	0.5925	0.11	0.82	0.22708	0.28105-0.48527
1990-2009	Enzyme_Protease	Target median	19	0	0.38	0.22	0.22	0.58	0.21	0.73	0.21286	0.28428-0.47572
1990-2009	Enzyme_Protease	[Drug - target median]	19	0	0.003158	-0.07	-0.01	0.0625	-0.13	0.18	0.089322	-0.037006-0.043322
1990-2009	Enzyme_Transferase	Drug	9	0	0.38667	0.3175	0.38	0.4625	0.29	0.49	0.077298	0.33617-0.43717
1990-2009	Enzyme_Transferase	Target median	9	0	0.39222	0.38	0.38	0.41	0.38	0.43	0.019221	0.37966-0.40478
1990-2009	Enzyme_Transferase	[Drug - target median]	9	0	-0.00556	-0.0625	-0.03	0.06	-0.12	0.11	0.078758	-0.057011-0.045899
1990-2009	Ionchannel	Drug	25	0	0.6584	0.5725	0.64	0.7925	0.37	0.93	0.15731	0.59673-0.72007
1990-2009	Ionchannel	Target median	25	0	0.6174	0.5475	0.61	0.66625	0.48	0.78	0.097287	0.57926-0.65554
1990-2009	Ionchannel	[Drug - target median]	25	2	0.041	-0.0225	0.02	0.135	-0.22	0.33	0.15556	-0.019981-0.10198
1990-2009	Membranereceptor_GPCR_amine	Drug	107	3	0.68636	0.6225	0.72	0.8075	0.35	0.9	0.14856	0.65821-0.7145
1990-2009	Membranereceptor_GPCR_amine	Target median	107	8	0.6014	0.59	0.62	0.65	0.54	0.72	0.099995	0.58245-0.62035
1990-2009	Membranereceptor_GPCR_amine	[Drug - target median]	107	4	0.084953	0.01	0.09	0.17	-0.2	0.41	0.13266	0.059817-0.11009
1990-2009	Membranereceptor_GPCR_other	Drug	17	0	0.58824	0.315	0.55	0.9	0.17	0.9	0.27068	0.45956-0.71691
1990-2009	Membranereceptor_GPCR_other	Target median	17	0	0.54353	0.415	0.49	0.805	0.18	0.87	0.24635	0.42642-0.66064
1990-2009	Membranereceptor_GPCR_other	[Drug - target median]	17	2	0.044706	0.02	0.08	0.1025	-0.04	0.21	0.10254	-0.0040374-0.093449
1990-2009	Membranereceptor_GPCR_peptide	Drug	27	0	0.53222	0.36	0.54	0.74	0.07	0.91	0.21691	0.4504-0.61404
1990-2009	Membranereceptor_GPCR_peptide	Target median	27	0	0.4137	0.33	0.38	0.6	0.2	0.615	0.15457	0.3554-0.47201
1990-2009	Membranereceptor_GPCR_peptide	[Drug - target median]	27	1	0.11852	0.03125	0.18	0.2275	-0.09	0.39	0.1661	0.055867-0.18117
1990-2009	Other	Drug	20	0	0.286	0.13	0.14	0.38	0.13	0.75	0.21661	0.19107-0.38093
1990-2009	Other	Target median	20	0	0.432	0.35	0.365	0.56	0.29	0.75	0.12677	0.37644-0.48756
1990-2009	Other	[Drug - target median]	20	0	-0.146	-0.245	-0.205	0.025	-0.44	0.18	0.19116	-0.22978--0.062222
1990-2009	Transcriptionfactor_NHR	Drug	36	2	0.56722	0.52	0.545	0.715	0.32	0.85	0.16666	0.51278-0.62166
1990-2009	Transcriptionfactor_NHR	Target median	36	8	0.52556	0.51	0.525	0.575	0.51	0.62	0.090315	0.49605-0.55506
1990-2009	Transcriptionfactor_NHR	[Drug - target median]	36	1	0.041667	-0.035	0.02	0.19	-0.36	0.5	0.22258	-0.031041-0.11437
1990-2009	Transporter	Drug	24	2	0.79542	0.75	0.84	0.885	0.68	0.93	0.12799	0.74421-0.84662
1990-2009	Transporter	Target median	24	2	0.75958	0.75	0.765	0.81	0.75	0.81	0.06549	0.73338-0.78578
1990-2009	Transporter	[Drug - target median]	24	2	0.035833	-0.015	0.075	0.1225	-0.09	0.185	0.13378	-0.01769-0.089356
2010-2020	Enzyme_Hydrolase	Drug	5	0	0.406	0.2575	0.28	0.6	0.19	0.72	0.22401	0.20965-0.60235
2010-2020	Enzyme_Hydrolase	Target median	5	0	0.498	0.4875	0.5	0.515	0.45	0.53	0.029496	0.47215-0.52385
2010-2020	Enzyme_Hydrolase	[Drug - target median]	5	0	-0.092	-0.265	-0.23	0.1375	-0.31	0.22	0.23962	-0.30204-0.11804
2010-2020	Enzyme_Kinase	Drug	116	4	0.46362	0.35	0.42	0.54	0.22	0.8	0.15917	0.43465-0.49259
2010-2020	Enzyme_Kinase	Target	116	2	0.43595	0.38	0.4125	0.47	0.33	0.6	0.070689	0.42308-0.44881
2010-2020	Enzyme_Kinase	[Drug - target median]	116	0	0.027672	-0.075	0.02	0.12	-0.23	0.38	0.12884	0.0042262-0.051119
2010-2020	Enzyme_Oxidoreductase	Drug	6	0	0.615	0.44	0.62	0.83	0.29	0.89	0.24164	0.42165-0.80835
2010-2020	Enzyme_Oxidoreductase	Target median	6	0	0.58167	0.54	0.605	0.68	0.35	0.71	0.13318	0.4751-0.68823
2010-2020	Enzyme_Oxidoreductase	[Drug - target median]	6	0	0.033333	-0.06	-0.01	0.15	-0.1	0.23	0.13231	-0.072539-0.13921
2010-2020	Enzyme_Phosphodiesterase	Drug	7	1	0.63571	0.6	0.7	0.7	0.6	0.7	0.094491	0.56571-0.70571
2010-2020	Enzyme_Phosphodiesterase	Target median	7	0	0.55429	0.53	0.59	0.5975	0.44	0.6	0.059121	0.51049-0.59808
2010-2020	Enzyme_Phosphodiesterase	[Drug - target median]	7	0	0.081429	0.0025	0.1	0.155	-0.08	0.26	0.11452	-0.0034073-0.16626
2010-2020	Enzyme_Protease	Drug	19	0	0.55053	0.305	0.48	0.795	0.22	0.87	0.24029	0.44248-0.65857
2010-2020	Enzyme_Protease	Target median	19	0	0.45263	0.21	0.34	0.73	0.18	0.73	0.24926	0.34055-0.56471
2010-2020	Enzyme_Protease	[Drug - target median]	19	3	0.097895	0.055	0.1	0.14	-0.04	0.18	0.14191	0.034082-0.16171
2010-2020	Enzyme_Transferase	Drug	14	0	0.62286	0.55	0.62	0.69	0.45	0.78	0.11255	0.5639-0.68182
2010-2020	Enzyme_Transferase	Target median	14	0	0.62357	0.55	0.69	0.69	0.43	0.69	0.086078	0.57848-0.66866
2010-2020	Enzyme_Transferase	[Drug - target median]	14	0	-7.14E-04	-0.1	0	0.09	-0.13	0.2	0.10057	-0.053397-0.051969
2010-2020	Ionchannel	Drug	6	0	0.54833	0.36	0.575	0.77	0.08	0.93	0.30036	0.30799-0.78867
2010-2020	Ionchannel	Target median	6	2	0.52833	0.55	0.55	0.62	0.55	0.62	0.21904	0.35307-0.7036
2010-2020	Ionchannel	[Drug - target median]	6	0	0.02	-0.04	0.025	0.15	-0.19	0.15	0.12775	-0.082221-0.12222
2010-2020	Membranereceptor_GPCR_amine	Drug	15	0	0.66533	0.525	0.67	0.8725	0.27	0.93	0.20702	0.56057-0.7701
2010-2020	Membranereceptor_GPCR_amine	Target median	15	2	0.602	0.6	0.61	0.6975	0.59	0.72	0.12946	0.53648-0.66752
2010-2020	Membranereceptor_GPCR_amine	[Drug - target median]	15	0	0.063333	-0.0275	0.14	0.205	-0.34	0.33	0.19025	-0.032947-0.15961
2010-2020	Membranereceptor_GPCR_other	Drug	9	0	0.36889	0.275	0.3	0.5375	0.05	0.65	0.19374	0.24231-0.49547
2010-2020	Membranereceptor_GPCR_other	Target median	9	0	0.44778	0.3425	0.49	0.51	0.32	0.54	0.090247	0.38882-0.50674
2010-2020	Membranereceptor_GPCR_other	[Drug - target median]	9	0	-0.07889	-0.2125	-0.09	0.05	-0.27	0.11	0.14066	-0.17079-0.013011
2010-2020	Membranereceptor_GPCR_peptide	Drug	14	0	0.37571	0.24	0.35	0.49	0.15	0.66	0.1556	0.29421-0.45722
2010-2020	Membranereceptor_GPCR_peptide	Target median	14	0	0.44929	0.37	0.415	0.58	0.26	0.6	0.12257	0.38508-0.51349
2010-2020	Membranereceptor_GPCR_peptide	[Drug - target median]	14	3	-0.07357	-0.11	-0.06	-0.02	-0.11	0.07	0.13189	-0.14266--0.0044852
2010-2020	Other	Drug	26	0	0.34808	0.23	0.34	0.45	0.13	0.76	0.14272	0.29322-0.40294
2010-2020	Other	Target median	26	4	0.36731	0.33	0.35	0.37	0.29	0.38	0.10466	0.32708-0.40754
2010-2020	Other	[Drug - target median]	26	0	-0.01923	-0.12	-0.025	0.1	-0.32	0.16	0.12376	-0.066804-0.028342
2010-2020	Transcriptionfactor_NHR	Drug	4	0	0.4775	0.39	0.545	0.565	0.24	0.58	0.15924	0.32144-0.63356
2010-2020	Transcriptionfactor_NHR	Target median	4	0	0.545	0.38	0.545	0.71	0.38	0.71	0.19053	0.35828-0.73172
2010-2020	Transcriptionfactor_NHR	[Drug - target median]	4	0	-0.0675	-0.165	-0.15	0.03	-0.17	0.2	0.17877	-0.24269-0.10769
2010-2020	Transporter	Drug	11	0	0.63818	0.5325	0.58	0.805	0.42	0.93	0.16958	0.53797-0.7384
2010-2020	Transporter	Target median	11	0	0.61818	0.51	0.51	0.75	0.51	0.81	0.12766	0.54274-0.69362
2010-2020	Transporter	[Drug - target median]	11	1	0.02	-0.01	0.03	0.1	-0.04	0.18	0.13229	-0.058177-0.098177

Approval period	Therapeutic use	Group	Total Count	Outlier Count	Mean Value	1st Quartile	Median	3rd Quartile	Lower Adjacent Limit	Upper Adjacent Limit	Standard Deviation	Confidence Interval (95%)
1939-1989	Cancer	Drug	11	0	0.43818	0.2525	0.49	0.63	0.14	0.66	0.19605	0.32232-0.55404
1939-1989	Cancer	Target median	11	0	0.55273	0.46	0.57	0.66	0.39	0.71	0.11533	0.48457-0.62089
1939-1989	Cancer	[Drug - target median]	11	0	-0.11455	-0.225	-0.05	-0.0225	-0.39	0.04	0.14848	-0.20229--0.026797
1939-1989	Cardiovascular	Drug	46	2	0.60239	0.51	0.625	0.74	0.17	0.91	0.2	0.54459-0.66019
1939-1989	Cardiovascular	Target median	46	0	0.4737	0.31	0.49	0.62	0.2	0.75	0.17261	0.42382-0.52358
1939-1989	Cardiovascular	[Drug - target median]	46	0	0.1287	-0.07	0.14	0.33	-0.38	0.55	0.24815	0.056985-0.20041
1939-1989	Central nervous system	Drug	74	1	0.73824	0.66	0.78	0.83	0.43	0.92	0.11696	0.71159-0.76489
1939-1989	Central nervous system	Target median	74	12	0.65716	0.61	0.62	0.68	0.6	0.78	0.07939	0.63907-0.67525
1939-1989	Central nervous system	[Drug - target median]	74	2	0.081081	0.01	0.09	0.18	-0.21	0.3	0.12451	0.052712-0.10945
1939-1989	Gastrointestinal & metabolism	Drug	18	0	0.60889	0.51	0.625	0.79	0.23	0.89	0.20565	0.51388-0.7039
1939-1989	Gastrointestinal & metabolism	Target median	18	0	0.58056	0.48	0.61	0.64	0.38	0.81	0.1068	0.53122-0.62989
1939-1989	Gastrointestinal & metabolism	[Drug - target median]	18	0	0.028333	-0.11	0.07	0.17	-0.37	0.28	0.18523	-0.057237-0.1139
1939-1989	Genito urinary	Drug	16	2	0.6775	0.635	0.705	0.78	0.6	0.83	0.14572	0.6061-0.7489
1939-1989	Genito urinary	Target median	16	0	0.57813	0.52	0.605	0.655	0.34	0.71	0.10591	0.52623-0.63002
1939-1989	Genito urinary	[Drug - target median]	16	0	0.099375	-0.005	0.09	0.25	-0.09	0.29	0.12772	0.036791-0.16196
1939-1989	Infection	Drug	5	0	0.568	0.28	0.73	0.8075	0.13	0.86	0.31987	0.28762-0.84838
1939-1989	Infection	Target median	5	0	0.588	0.495	0.66	0.69	0.36	0.69	0.14167	0.46382-0.71218
1939-1989	Infection	[Drug - target median]	5	0	-0.02	-0.155	0.1	0.175	-0.53	0.19	0.29783	-0.28106-0.24106
1939-1989	Multiple/other	Drug	32	2	0.70969	0.67	0.755	0.85	0.43	0.9	0.18901	0.6442-0.77518
1939-1989	Multiple/other	Target median	32	0	0.58969	0.515	0.57	0.69	0.4	0.78	0.10002	0.55503-0.62434
1939-1989	Multiple/other	[Drug - target median]	32	2	0.12	0.07	0.16	0.19	-0.06	0.37	0.15585	0.066-0.174
1939-1989	Musculo-skeletal	Drug	15	2	0.77	0.745	0.83	0.86	0.59	0.89	0.13088	0.70377-0.83623
1939-1989	Musculo-skeletal	Target median	15	2	0.666	0.66	0.66	0.72	0.62	0.72	0.055007	0.63816-0.69384
1939-1989	Musculo-skeletal	[Drug - target median]	15	1	0.104	0.0725	0.13	0.1925	-0.09	0.26	0.11531	0.045643-0.16236
1939-1989	Respiratory	Drug	23	0	0.68957	0.5925	0.69	0.7975	0.39	0.86	0.12553	0.63826-0.74087
1939-1989	Respiratory	Target median	23	0	0.53087	0.3475	0.62	0.62	0.29	0.73	0.15288	0.46839-0.59335
1939-1989	Respiratory	[Drug - target median]	23	2	0.1587	0.07	0.18	0.26	-0.04	0.45	0.16788	0.090084-0.22731
1939-1989	Skin & eye	Drug	19	0	0.67	0.6075	0.66	0.77	0.42	0.88	0.12342	0.6145-0.7255
1939-1989	Skin & eye	Target median	19	3	0.52316	0.52	0.52	0.5725	0.5	0.61	0.09592	0.48003-0.56629
1939-1989	Skin & eye	[Drug - target median]	19	1	0.14684	0.0325	0.13	0.25	-0.19	0.5	0.18251	0.064773-0.22891
1990-2009	Cancer	Drug	30	0	0.45267	0.27	0.465	0.63	0.13	0.87	0.22497	0.37216-0.53317
1990-2009	Cancer	Target median	30	0	0.50367	0.41	0.515	0.63	0.22	0.71	0.13208	0.4564-0.55093
1990-2009	Cancer	[Drug - target median]	30	0	-0.051	-0.23	-0.01	0.1	-0.44	0.27	0.20516	-0.12442-0.022417
1990-2009	Cardiovascular	Drug	37	0	0.47514	0.365	0.47	0.5925	0.16	0.85	0.16584	0.4217-0.52857
1990-2009	Cardiovascular	Target median	37	0	0.41541	0.305	0.37	0.5575	0.2	0.75	0.159	0.36417-0.46664
1990-2009	Cardiovascular	[Drug - target median]	37	0	0.05973	-0.08	0.04	0.1675	-0.29	0.47	0.17658	0.0028318-0.11663
1990-2009	Central nervous system	Drug	57	2	0.77596	0.72	0.8	0.8525	0.59	0.93	0.11074	0.74722-0.80471
1990-2009	Central nervous system	Target median	57	0	0.65623	0.6	0.65	0.75	0.45	0.87	0.096687	0.63113-0.68133
1990-2009	Central nervous system	[Drug - target median]	57	1	0.11974	0.0375	0.13	0.19625	-0.2	0.37	0.11893	0.088861-0.15061
1990-2009	Gastrointestinal & metabolism	Drug	23	0	0.63696	0.47	0.72	0.7975	0.25	0.93	0.19703	0.55643-0.71748
1990-2009	Gastrointestinal & metabolism	Target median	23	0	0.56174	0.385	0.59	0.73	0.32	0.78	0.17695	0.48942-0.63406
1990-2009	Gastrointestinal & metabolism	[Drug - target median]	23	4	0.075217	-0.02	0.04	0.14125	-0.15	0.19	0.18988	-0.0023856-0.15282
1990-2009	Genito urinary	Drug	18	1	0.56389	0.52	0.61	0.69	0.32	0.81	0.18627	0.47784-0.64994
1990-2009	Genito urinary	Target median	18	0	0.56444	0.53	0.54	0.61	0.47	0.64	0.047555	0.54248-0.58641
1990-2009	Genito urinary	[Drug - target median]	18	3	-5.56E-04	-0.01	0.025	0.12	-0.17	0.2	0.17193	-0.079981-0.07887
1990-2009	Infection	Drug	19	0	0.39263	0.2125	0.33	0.525	0.11	0.86	0.21173	0.29742-0.48784
1990-2009	Infection	Target median	19	0	0.43316	0.22	0.45	0.66	0.22	0.66	0.20257	0.34207-0.52424
1990-2009	Infection	[Drug - target median]	19	0	-0.04053	-0.125	-0.02	0.105	-0.41	0.2	0.19182	-0.12678-0.045726
1990-2009	Multiple/other	Drug	10	0	0.514	0.29	0.62	0.65	0.16	0.75	0.22152	0.3767-0.6513
1990-2009	Multiple/other	Target median	10	0	0.4805	0.37	0.495	0.615	0.26	0.66	0.13305	0.39803-0.56297
1990-2009	Multiple/other	[Drug - target median]	10	3	0.0335	0.03	0.07	0.09	0.03	0.11	0.13622	-0.05093-0.11793
1990-2009	Musculo-skeletal	Drug	18	0	0.66833	0.47	0.785	0.85	0.31	0.91	0.22216	0.5657-0.77097
1990-2009	Musculo-skeletal	Target median	18	0	0.56556	0.38	0.66	0.66	0.38	0.72	0.1437	0.49917-0.63194
1990-2009	Musculo-skeletal	[Drug - target median]	18	1	0.10278	0.04	0.105	0.16	-0.07	0.25	0.11483	0.049729-0.15583
1990-2009	Respiratory	Drug	20	0	0.5255	0.375	0.545	0.665	0.17	0.79	0.17288	0.44973-0.60127
1990-2009	Respiratory	Target median	20	0	0.463	0.29	0.52	0.62	0.18	0.62	0.16781	0.38946-0.53654
1990-2009	Respiratory	[Drug - target median]	20	2	0.0625	0.005	0.08	0.14	-0.12	0.21	0.13722	0.0023592-0.12264
1990-2009	Skin & eye	Drug	11	0	0.64182	0.535	0.64	0.81	0.3	0.85	0.1694	0.54171-0.74193
1990-2009	Skin & eye	Target median	11	0	0.54727	0.4975	0.55	0.6475	0.34	0.67	0.10659	0.48428-0.61026
1990-2009	Skin & eye	[Drug - target median]	11	0	0.094545	-0.0125	0.1	0.1575	-0.04	0.3	0.10624	0.031761-0.15733
2010-2020	Cancer	Drug	61	0	0.47574	0.37	0.47	0.565	0.08	0.8	0.15179	0.43764-0.51383
2010-2020	Cancer	Target median	61	1	0.47025	0.4	0.44	0.5525	0.33	0.71	0.11794	0.44065-0.49984
2010-2020	Cancer	[Drug - target median]	61	0	0.005492	-0.1	-0.01	0.1025	-0.23	0.27	0.12097	-0.024867-0.035851
2010-2020	Cardiovascular	Drug	12	0	0.39583	0.235	0.385	0.515	0.05	0.78	0.20129	0.28194-0.50972
2010-2020	Cardiovascular	Target median	12	0	0.37583	0.33	0.34	0.435	0.26	0.51	0.080505	0.33028-0.42138
2010-2020	Cardiovascular	[Drug - target median]	12	1	0.02	-0.1	-0.04	0.095	-0.27	0.29	0.19424	-0.089899-0.1299
2010-2020	Central nervous system	Drug	13	0	0.67462	0.5	0.72	0.785	0.37	0.93	0.18205	0.57565-0.77358
2010-2020	Central nervous system	Target median	13	0	0.61923	0.57	0.62	0.7	0.42	0.75	0.096735	0.56664-0.67182
2010-2020	Central nervous system	[Drug - target median]	13	0	0.055385	-0.035	0.07	0.15	-0.18	0.21	0.11956	-0.0096067-0.12038
2010-2020	Gastrointestinal & metabolism	Drug	22	0	0.59636	0.52	0.58	0.76	0.21	0.87	0.18477	0.51915-0.67357
2010-2020	Gastrointestinal & metabolism	Target median	22	0	0.57727	0.51	0.55	0.73	0.35	0.73	0.14096	0.51837-0.63617
2010-2020	Gastrointestinal & metabolism	[Drug - target median]	22	2	0.019091	-0.02	0.03	0.12	-0.07	0.2	0.13694	-0.038131-0.076313
2010-2020	Genito urinary	Drug	4	0	0.3475	0.2	0.345	0.495	0.16	0.54	0.17727	0.17377-0.52123
2010-2020	Genito urinary	Target median	4	0	0.3425	0.26	0.29	0.425	0.26	0.53	0.12816	0.2169-0.4681
2010-2020	Genito urinary	[Drug - target median]	4	0	0.005	-0.09	-0.05	0.1	-0.1	0.22	0.14731	-0.13936-0.14936
2010-2020	Infection	Drug	8	1	0.30625	0.235	0.29	0.355	0.13	0.36	0.12817	0.21743-0.39507
2010-2020	Infection	Target median	8	0	0.295	0.18	0.21	0.475	0.12	0.51	0.16204	0.18271-0.40729
2010-2020	Infection	[Drug - target median]	8	0	0.01125	-0.11	0.08	0.14	-0.31	0.18	0.18295	-0.11553-0.13803
2010-2020	Multiple/other	Drug	11	0	0.56636	0.3225	0.62	0.745	0.15	0.93	0.2575	0.41419-0.71854
2010-2020	Multiple/other	Target median	11	1	0.54909	0.5025	0.51	0.5875	0.38	0.71	0.12308	0.47635-0.62183
2010-2020	Multiple/other	[Drug - target median]	11	0	0.017273	-0.1725	0.04	0.125	-0.26	0.38	0.19658	-0.098896-0.13344
2010-2020	Musculo-skeletal	Drug	3	0	0.74	0.6525	0.69	0.84	0.64	0.89	0.13229	0.5903-0.8897
2010-2020	Musculo-skeletal	Target median	3	0	0.63	0.5775	0.66	0.675	0.55	0.68	0.07	0.55079-0.70921
2010-2020	Musculo-skeletal	[Drug - target median]	3	0	0.11	0.005	0.14	0.2075	-0.04	0.23	0.13748	-0.04557-0.26557
2010-2020	Respiratory	Drug	7	0	0.48571	0.37	0.52	0.5775	0.27	0.7	0.14876	0.37551-0.59591
2010-2020	Respiratory	Target median	7	0	0.51429	0.4675	0.55	0.595	0.29	0.61	0.114	0.42984-0.59874
2010-2020	Respiratory	[Drug - target median]	7	0	-0.02857	-0.205	0.02	0.18	-0.34	0.26	0.22799	-0.19747-0.14033
