# Supplementary material for: Target-based evaluation of ‘drug-like’ properties and ligand efficiencies
Source: J Med Chem. Author manuscript; Available in PMC 2021 Jun 11. (PMC7610969; doi:10.1021/acs.jmedchem.1c00416)
Supplement: Supp Fig S6 values LLE [file EMS123358-supplement-Supp_Fig_S6_values_LLE.pdf]

LLE

Approval period	Target class	Group	Total Count	Outlier Count	Mean Value	1st Quartile	Median	3rd Quartile	Lower Adjacent Limit	Upper Adjacent Limit	Standard Deviation	Confidence Interval (95%)
1939-89	Enzyme_Hydrolase	Drug	7	0	4.4379	2.7638	4.235	5.8025	1.87	7.055	1.8935	3.0351-5.8406
1939-89	Enzyme_Hydrolase	Target median	7	1	1.8829	1.73	1.73	1.73	1.73	1.73	0.40442	1.5833-2.1825
1939-89	Enzyme_Hydrolase	[Drug - target median]	7	0	2.555	1.0337	2.505	4.0725	0.14	4.255	1.6779	1.312-3.798
1939-89	Enzyme_Other	Drug	32	2	5.9772	4.8725	6.32	7.3325	1.56	10.1	2.3191	5.1737-6.7807
1939-89	Enzyme_Other	Target median	32	0	4.1252	3.18	4.74	5.46	1.01	5.845	1.6513	3.5531-4.6974
1939-89	Enzyme_Other	[Drug - target median]	32	0	1.852	0.91	1.485	2.6875	-1.61	4.99	1.5013	1.3318-2.3721
1939-89	Enzyme_Oxidoreductase	Drug	46	2	3.6072	2.39	3.3625	4.475	-0.35	6.22	1.6353	3.1346-4.0797
1939-89	Enzyme_Oxidoreductase	Target median	46	4	2.1693	1.41	2.31	2.31	1.41	3.42	0.80766	1.9359-2.4028
1939-89	Enzyme_Oxidoreductase	[Drug - target median]	46	1	1.4378	0.32	1.525	2.64	-0.81	4.37	1.619	0.96996-1.9057
1939-89	Enzyme_Phosphodiesterase	Drug	9	0	4.3628	3.2262	3.82	5.6	2.75	6.26	1.3711	3.467-5.2586
1939-89	Enzyme_Phosphodiesterase	Target median	9	0	3.0422	2.395	2.805	3.8187	2.06	4.29	0.78919	2.5266-3.5578
1939-89	Enzyme_Phosphodiesterase	[Drug - target median]	9	0	1.3206	0.86	1.42	1.8988	-0.235	2.43	0.92937	0.71337-1.9277
1939-89	Enzyme_Protease	Drug	7	0	5.5679	4.2625	4.86	7.0463	3.81	7.41	1.53	4.4344-6.7013
1939-89	Enzyme_Protease	Target median	7	0	4.1079	3.2013	4.525	4.7313	2.76	4.86	0.93097	3.4182-4.7975
1939-89	Enzyme_Protease	[Drug - target median]	7	0	1.46	0.3075	1.34	2.5213	-0.11	2.885	1.2107	0.56307-2.3569
1939-89	Ion channel	Drug	24	0	3.2444	1.975	2.965	4.455	-1.44	7.02	1.9229	2.475-4.0137
1939-89	Ion channel	Target median	24	0	2.8069	1.5375	2.2125	3.825	0.91	5.15	1.3387	2.2713-3.3425
1939-89	Ion channel	[Drug - target median]	24	2	0.4375	-0.35	0.24625	1.2313	-2.08	2.77	1.6999	-0.24258-1.1176
1939-89	Membrane receptor_GPCR_amine	Drug	180	2	5.1045	4.105	5.185	6.0625	1.61	8.57	1.5659	4.8757-5.3332
1939-89	Membrane receptor_GPCR_amine	Target median	180	5	3.1161	2.74	3.16	3.38	2.61	3.95	0.47018	3.0474-3.1848
1939-89	Membrane receptor_GPCR_amine	[Drug - target median]	180	3	1.9884	0.91	1.9725	3.0825	-1.53	5.57	1.5923	1.7558-2.221
1939-89	Membrane receptor_GPCR_other	Drug	23	2	6.0257	5.31	5.78	7.2975	4.985	9.72	1.7609	5.306-6.7453
1939-89	Membrane receptor_GPCR_other	Target median	23	0	3.5073	2.2238	4.09	4.3925	1.5025	5.26	1.1919	3.0202-3.9944
1939-89	Membrane receptor_GPCR_other	[Drug - target median]	23	0	2.5184	1.6387	2.27	3.5587	0.4	5.63	1.3651	1.9605-3.0763
1939-89	Membrane receptor_GPCR_peptide	Drug	31	2	6.0918	4.6425	5.95	7.2025	2.78	7.92	2.0219	5.38-6.8035
1939-89	Membrane receptor_GPCR_peptide	Target median	31	1	2.8884	2.775	2.775	3.05	2.38	3.05	0.23922	2.8042-2.9726
1939-89	Membrane receptor_GPCR_peptide	[Drug - target median]	31	1	3.2034	1.7563	3.06	4.3025	-0.11	7.74	1.9895	2.503-3.9037
1939-89	Other	Drug	7	1	3.4829	2.6625	3.08	4.0275	2.32	4.31	1.3077	2.5141-4.4516
1939-89	Other	Target median	7	0	2.9071	1.58	3.04	3.58	1.42	5.25	1.3876	1.8792-3.9351
1939-89	Other	[Drug - target median]	7	0	0.57571	-0.1925	0.83	1.18	-0.72	1.37	0.84077	-0.047137-1.1986
1939-89	Transcription factor_NHR	Drug	46	0	4.1484	2.5	4.495	5.98	0.28	7.32	1.995	3.5718-4.7249
1939-89	Transcription factor_NHR	Target median	46	0	1.9155	1.41	2.0925	2.43	0.45	2.7525	0.71264	1.7096-2.1215
1939-89	Transcription factor_NHR	[Drug - target median]	46	0	2.2328	1.01	2.3187	3.59	-1.72	4.83	1.5529	1.7841-2.6816
1939-89	Transporter	Drug	35	2	4.1074	3.3775	4.265	4.835	1.73	5.53	1.3644	3.6554-4.5595
1939-89	Transporter	Target median	35	3	3.0511	2.78	3.155	3.23	2.67	3.23	0.51288	2.8812-3.2211
1939-89	Transporter	[Drug - target median]	35	0	1.0563	0.2225	1.25	2.1825	-2.58	3.025	1.2814	0.63177-1.4808
1990-2009	Enzyme_Hydrolase	Drug	19	0	6.2192	3.6925	5.865	7.7712	2.92	12.28	2.9699	4.8838-7.5546
1990-2009	Enzyme_Hydrolase	Target median	19	0	3.4492	1.73	2.8	5.69	1.1	6.815	2.0003	2.5498-4.3486
1990-2009	Enzyme_Hydrolase	[Drug - target median]	19	0	2.77	1.5438	2.36	3.7438	0.44	6.265	1.7341	1.9902-3.5498
1990-2009	Enzyme_Kinase	Drug	51	0	3.8913	2.29	4.285	5.0363	1.34	6.37	1.5291	3.4716-4.3109
1990-2009	Enzyme_Kinase	Target median	51	0	2.5315	2.27	2.53	2.7825	1.7	3.5	0.4402	2.4107-2.6523
1990-2009	Enzyme_Kinase	[Drug - target median]	51	0	1.3598	-0.23	1.445	2.6662	-2.11	4.23	1.6736	0.90048-1.8191
1990-2009	Enzyme_Other	Drug	12	0	5.2717	4.07	4.96	6.315	3.04	8.4	1.7612	4.2752-6.2682
1990-2009	Enzyme_Other	Target median	12	0	3.655	2.48	3.165	5.02	2.37	5.73	1.4221	2.8504-4.4596
1990-2009	Enzyme_Other	[Drug - target median]	12	0	1.6167	0.73	1.515	2.48	0.29	3.14	0.99086	1.056-2.1773
1990-2009	Enzyme_Oxidoreductase	Drug	32	0	3.9539	3.15	4.1425	5.07	0.53	5.9	1.3632	3.4816-4.4262
1990-2009	Enzyme_Oxidoreductase	Target median	32	0	2.5505	2.255	2.31	3.23	1.04	4.27	0.82671	2.264-2.8369
1990-2009	Enzyme_Oxidoreductase	[Drug - target median]	32	1	1.4034	0.555	1.405	2.345	-1.46	4.015	1.5286	0.87381-1.9331
1990-2009	Enzyme_Phosphodiesterase	Drug	4	0	6.2675	5.6425	6.4125	6.8925	5.165	7.08	0.83443	5.4498-7.0852
1990-2009	Enzyme_Phosphodiesterase	Target median	4	0	3.6475	3.465	3.83	3.83	3.1	3.83	0.365	3.2898-4.0052
1990-2009	Enzyme_Phosphodiesterase	[Drug - target median]	4	0	2.62	2.1775	2.5825	3.0625	2.065	3.25	0.54125	2.0896-3.1504
1990-2009	Enzyme_Protease	Drug	19	0	6.0608	5.4587	5.915	7.09	3.32	8.37	1.2606	5.494-6.6276
1990-2009	Enzyme_Protease	Target median	19	0	4.0161	3.61	3.61	4.525	3.32	4.99	0.57944	3.7555-4.2766
1990-2009	Enzyme_Protease	[Drug - target median]	19	0	2.0447	1.1013	2.3	2.9612	-0.29	3.845	1.2284	1.4924-2.5971
1990-2009	Enzyme_Transferase	Drug	9	0	6.8767	5.675	7.765	8.1325	4.19	8.74	1.6665	5.7879-7.9654
1990-2009	Enzyme_Transferase	Target median	9	2	4.4906	4.4412	4.81	4.81	4.795	4.81	0.62964	4.0792-4.9019
1990-2009	Enzyme_Transferase	[Drug - target median]	9	0	2.3861	1.2337	2.955	3.3225	0.81	3.93	1.1603	1.6281-3.1442
1990-2009	Ion channel	Drug	25	0	3.936	2.1775	3.515	6.0687	0.81	7.53	2.0574	3.1295-4.7425
1990-2009	Ion channel	Target median	25	0	2.8335	1.8537	2.14	3.8913	1.47	5.15	1.3123	2.3191-3.3479
1990-2009	Ion channel	[Drug - target median]	25	0	1.1025	-0.22375	0.855	2.0044	-1.36	4.525	1.6178	0.46831-1.7367
1990-2009	Membrane receptor_GPCR_amine	Drug	107	0	5.0394	4.135	4.96	5.96	2.06	8.35	1.3232	4.7887-5.2902
1990-2009	Membrane receptor_GPCR_amine	Target median	107	0	3.2747	2.845	3.205	3.595	2.61	4.61	0.45728	3.188-3.3613
1990-2009	Membrane receptor_GPCR_amine	[Drug - target median]	107	0	1.7648	0.89375	1.79	2.6388	-0.785	5.145	1.2145	1.5347-1.9949
1990-2009	Membrane receptor_GPCR_other	Drug	17	0	5.1209	3.2875	4.88	7.5925	-0.31	8.96	2.7581	3.8097-6.432
1990-2009	Membrane receptor_GPCR_other	Target median	17	0	2.9862	1.9488	2.16	4.3238	0.97	5.26	1.5499	2.2494-3.7229
1990-2009	Membrane receptor_GPCR_other	[Drug - target median]	17	0	2.1347	1.2162	2.425	3.0763	-1.28	4.87	1.6087	1.37-2.8994
1990-2009	Membrane receptor_GPCR_peptide	Drug	27	1	4.4165	2.8775	3.95	5.7737	-0.2	7.41	2.1762	3.5956-5.2373
1990-2009	Membrane receptor_GPCR_peptide	Target median	27	1	2.5422	2.2325	2.38	2.8613	1.51	3.51	0.5656	2.3289-2.7556
1990-2009	Membrane receptor_GPCR_peptide	[Drug - target median]	27	1	1.8743	0.85875	1.8	2.945	-2.05	4.36	1.7811	1.2024-2.5461
1990-2009	Other	Drug	20	0	5.191	4.29	4.8825	6.8525	1.18	9.03	2.0736	4.2822-6.0998
1990-2009	Other	Target median	20	6	3.0635	2.8425	3.0675	3.48	2.72	3.87	1.0411	2.6072-3.5198
1990-2009	Other	[Drug - target median]	20	0	2.1275	0.845	1.6825	3.7575	-0.905	6.02	1.8667	1.3094-2.9456
1990-2009	Transcription factor_NHR	Drug	36	0	2.5169	1.4275	2.205	3.52	-0.72	5.97	1.6466	1.9791-3.0548
1990-2009	Transcription factor_NHR	Target median	36	0	1.5131	0.82	1.265	2.43	0.45	2.7525	0.79537	1.2532-1.7729
1990-2009	Transcription factor_NHR	[Drug - target median]	36	1	1.0039	0.205	1.1937	1.725	-1.73	3.72	1.5168	0.50839-1.4994
1990-2009	Transporter	Drug	24	0	3.8346	2.98	3.96	4.9175	0.51	6.595	1.4632	3.2492-4.42
1990-2009	Transporter	Target median	24	2	3.0417	2.9125	3.155	3.23	2.67	3.23	0.28432	2.9279-3.1554
1990-2009	Transporter	[Drug - target median]	24	1	0.79292	0	1.0825	1.725	-2.15	3.365	1.4319	0.22003-1.3658
2010-2020	Enzyme_Hydrolase	Drug	5	0	7.375	4.8863	8.27	9.4725	3.27	10.92	3.0261	4.7225-10.028
2010-2020	Enzyme_Hydrolase	Target median	5	0	4.0385	2.3637	4.1825	5.7712	1.73	6.015	1.8792	2.3913-5.6857
2010-2020	Enzyme_Hydrolase	[Drug - target median]	5	0	3.3365	2.1087	2.975	4.4556	0.695	6.7375	2.2009	1.4073-5.2657
2010-2020	Enzyme_Kinase	Drug	116	0	3.5487	2.4	3.635	4.78	0.13	7.3	1.7183	3.2361-3.8614
2010-2020	Enzyme_Kinase	Target	116	5	3.0095	2.36	2.84	3.4975	1.57	4.28	0.80523	2.863-3.1561
2010-2020	Enzyme_Kinase	[Drug - target median]	116	2	0.53922	-0.34	0.5875	1.5175	-2.885	3.93	1.4658	0.27248-0.80597
2010-2020	Enzyme_Oxidoreductase	Drug	6	0	4.0242	3.29	3.5575	5.27	2.36	6.11	1.3893	2.9125-5.1358
2010-2020	Enzyme_Oxidoreductase	Target median	6	0	2.53	2.31	2.43	2.9175	1.7925	3.3	0.52447	2.1103-2.9497
2010-2020	Enzyme_Oxidoreductase	[Drug - target median]	6	0	1.4942	0.05	1.3963	2.94	-0.01	3.1925	1.3824	0.38803-2.6003
2010-2020	Enzyme_Phosphodiesterase	Drug	7	0	4.6257	4.115	4.315	4.9925	4.025	5.91	0.67914	4.1226-5.1288
2010-2020	Enzyme_Phosphodiesterase	Target median	7	2	2.9457	2.7713	2.805	3.18	2.76	3.18	0.54011	2.5456-3.3458
2010-2020	Enzyme_Phosphodiesterase	[Drug - target median]	7	0	1.68	1.495	1.895	1.9513	0.88	2.08	0.41593	1.3719-1.9881
2010-2020	Enzyme_Protease	Drug	19	0	6.3471	4.895	6.495	7.7575	3.98	8.095	1.5295	5.6594-7.0349
2010-2020	Enzyme_Protease	Target median	19	0	4.3626	3.7675	4.23	4.99	3.64	4.99	0.58566	4.0993-4.626
2010-2020	Enzyme_Protease	[Drug - target median]	19	0	1.9845	1.0975	2.5	2.84	-0.18	3.93	1.1501	1.4673-2.5016
2010-2020	Enzyme_Transferase	Drug	14	0	5.6261	4.46	5.1425	6.44	3.75	8.64	1.5002	4.8402-6.4119
2010-2020	Enzyme_Transferase	Target median	14	1	4.3446	4.175	4.54	4.63	3.8	4.795	0.41365	4.128-4.5613
2010-2020	Enzyme_Transferase	[Drug - target median]	14	2	1.2814	0.35	0.675	1.81	-0.425	1.9	1.5697	0.45916-2.1037
2010-2020	Ion channel	Drug	6	0	2.3217	0.835	2.5625	3.17	0.625	4.175	1.4039	1.1983-3.445
2010-2020	Ion channel	Target median	6	0	2.12	1.33	1.33	3.26	0.56	4.91	1.6364	0.81058-3.4294
2010-2020	Ion channel	[Drug - target median]	6	0	0.20167	-0.495	-0.0675	0.735	-0.735	1.84	0.94987	-0.55839-0.96172
2010-2020	Membrane receptor_GPCR_amine	Drug	15	0	5.008	4.415	5.08	5.5725	3.96	6.11	0.65948	4.6743-5.3417
2010-2020	Membrane receptor_GPCR_amine	Target median	15	1	3.4143	3.1713	3.37	3.5738	2.61	4.14	0.49555	3.1636-3.6651
2010-2020	Membrane receptor_GPCR_amine	[Drug - target median]	15	0	1.5937	1.235	1.81	1.9988	0.29	2.785	0.70752	1.2356-1.9517
2010-2020	Membrane receptor_GPCR_other	Drug	9	1	4.4417	3.5188	5.02	5.9075	2.63	6.17	2.1481	3.0383-5.8451
2010-2020	Membrane receptor_GPCR_other	Target median	9	0	3.2428	1.9938	3.59	4.1713	1.63	4.415	1.112	2.5163-3.9693
2010-2020	Membrane receptor_GPCR_other	[Drug - target median]	9	0	1.1989	0.4575	1.435	2.5125	-1.99	3.12	1.6804	0.10105-2.2967
2010-2020	Membrane receptor_GPCR_peptide	Drug	14	0	4.1371	2.84	4.0325	5.435	2.23	6.08	1.4165	3.3951-4.8792
2010-2020	Membrane receptor_GPCR_peptide	Target median	14	0	2.6268	2.27	2.795	3	1.51	3.83	0.64538	2.2887-2.9649
2010-2020	Membrane receptor_GPCR_peptide	[Drug - target median]	14	0	1.5104	0.445	1.5975	2.2	-0.415	4.17	1.2179	0.87241-2.1483
2010-2020	Other	Drug	26	0	4.5748	3.545	5.16	5.54	1.995	5.99	1.2741	4.0851-5.0645
2010-2020	Other	Target median	26	3	3.1726	2.97	3.16	3.31	2.72	3.62	0.3489	3.0385-3.3067
2010-2020	Other	[Drug - target median]	26	0	1.4022	0.45	1.84	2.405	-1.28	2.705	1.2077	0.938-1.8664
2010-2020	Transcription factor_NHR	Drug	4	0	3.0888	2.3225	3.1025	3.855	1.89	4.26	1.0084	2.1005-4.077
2010-2020	Transcription factor_NHR	Target median	4	0	1.6662	0.58	1.7313	2.7525	0.45	2.7525	1.2588	0.43265-2.8999
2010-2020	Transcription factor_NHR	[Drug - target median]	4	0	1.4225	0.72125	1.4738	2.1237	0.0025	2.74	1.1195	0.32541-2.5196
2010-2020	Transporter	Drug	11	1	5.5868	4.8775	5.69	6.8475	4.675	7.48	1.8353	4.5022-6.6714
2010-2020	Transporter	Target median	11	0	4.5077	3.23	5.66	5.66	2.78	5.66	1.3296	3.722-5.2935
2010-2020	Transporter	[Drug - target median]	11	1	1.0791	0.47875	1.445	1.8175	0.03	2.475	1.2156	0.36074-1.7974

Approval period	Therapeutic use	Group	Total Count	Outlier Count	Mean Value	1st Quartile	Median	3rd Quartile	Lower Adjacent Limit	Upper Adjacent Limit	Standard Deviation	Confidence Interval (95%)
1939-1989	Cancer	Drug	11	0	3.7732	2.545	3.32	5.1663	0.87	7.895	2.0511	2.5611-4.9853
1939-1989	Cancer	Target median	11	0	1.932	1.26	1.8	2.48	1.01	3.78	0.87043	1.4177-2.4464
1939-1989	Cancer	[Drug - target median]	11	0	1.8411	0.7125	1.52	3.5887	-1.61	4.74	1.8722	0.73474-2.9475
1939-1989	Cardiovascular	Drug	46	1	4.7924	3.265	5.1975	6.445	-1.44	9.72	2.6126	4.0374-5.5474
1939-1989	Cardiovascular	Target median	46	0	2.669	1.825	2.65	3.595	0.71	5.25	1.228	2.3141-3.0239
1939-1989	Cardiovascular	[Drug - target median]	46	3	2.1234	0.925	2.115	3.025	-0.58	6.11	2.0572	1.5289-2.7179
1939-1989	Central nervous system	Drug	74	1	4.8634	4.025	4.72	5.91	1.875	7.92	1.4831	4.5255-5.2013
1939-1989	Central nervous system	Target median	74	7	3.079	2.775	3	3.37	2.02	4.19	0.61248	2.9394-3.2185
1939-1989	Central nervous system	[Drug - target median]	74	0	1.7844	0.63	1.6525	2.775	-0.955	5.145	1.5397	1.4336-2.1352
1939-1989	Gastrointestinal & metabolism	Drug	18	2	4.5983	3.9	5.22	5.89	2.03	8.57	2.3361	3.5191-5.6775
1939-1989	Gastrointestinal & metabolism	Target median	18	0	2.4672	1.63	2.7575	3	0.45	3.77	0.8802	2.0606-2.8739
1939-1989	Gastrointestinal & metabolism	[Drug - target median]	18	1	2.1311	0.51	2.2975	3.135	0.04	6.675	2.3553	1.043-3.2192
1939-1989	Genito urinary	Drug	16	1	4.9125	4.245	5.01	5.665	3.24	7.6	1.7545	4.0528-5.7722
1939-1989	Genito urinary	Target median	16	0	2.3952	2	2.2875	2.7525	1.8	3.235	0.52161	2.1396-2.6507
1939-1989	Genito urinary	[Drug - target median]	16	1	2.5173	1.98	2.59	3.4025	0.4875	4.83	1.562	1.752-3.2827
1939-1989	Infection	Drug	5	0	5.08	4.44	5.02	5.7275	3.18	7.055	1.3788	3.8714-6.2886
1939-1989	Infection	Target median	5	0	3.708	2.615	4.44	4.53	2.06	4.8	1.2046	2.6521-4.7639
1939-1989	Infection	[Drug - target median]	5	1	1.372	0.45	0.845	1.9038	0.06	1.12	1.6584	-0.081676-2.8257
1939-1989	Multiple/other	Drug	32	1	5.1394	4.0625	5	6.2325	1.96	8	1.9468	4.4649-5.8139
1939-1989	Multiple/other	Target median	32	1	2.4519	1.73	2.43	2.9075	1.265	3.91	0.88264	2.1461-2.7577
1939-1989	Multiple/other	[Drug - target median]	32	1	2.6875	1.545	2.5	3.875	-0.18	4.84	1.5625	2.1461-3.2289
1939-1989	Musculo-skeletal	Drug	15	1	2.9783	1.8075	2.715	3.7062	0.2	5.2	1.7917	2.0716-3.8851
1939-1989	Musculo-skeletal	Target median	15	0	2.123	1.41	2.31	2.6625	1.41	3.42	0.66963	1.7841-2.4619
1939-1989	Musculo-skeletal	[Drug - target median]	15	0	0.85533	-0.2475	0.35	1.7375	-2.58	4.37	1.8611	-0.086489-1.7972
1939-1989	Respiratory	Drug	23	1	4.832	4.145	4.745	5.1275	3.25	6.44	1.0068	4.4205-5.2434
1939-1989	Respiratory	Target median	23	5	2.8726	2.61	2.845	2.845	2.43	3	0.4215	2.7003-3.0449
1939-1989	Respiratory	[Drug - target median]	23	2	1.9593	1.4725	1.905	2.2013	0.405	3.235	0.92248	1.5823-2.3364
1939-1989	Skin & eye	Drug	19	3	6.1505	5.18	6.11	6.835	4.91	9.18	1.8916	5.3-7.0011
1939-1989	Skin & eye	Target median	19	4	2.9563	2.43	2.43	3.205	1.73	3.595	1.2476	2.3953-3.5173
1939-1989	Skin & eye	[Drug - target median]	19	1	3.1942	2.52	3.185	3.8263	0.58	4.99	1.3719	2.5773-3.8111
1990-2009	Cancer	Drug	30	0	3.9745	2.42	4.17	5.165	0.27	9.03	1.8603	3.3088-4.6402
1990-2009	Cancer	Target median	30	1	2.5555	2.18	2.505	3.32	0.64	3.85	0.87643	2.2419-2.8691
1990-2009	Cancer	[Drug - target median]	30	1	1.419	0.3	1.4175	2.22	-2.11	4.19	1.6633	0.8238-2.0142
1990-2009	Cardiovascular	Drug	37	0	4.5501	3.675	4.45	5.9037	0.53	8.96	1.9602	3.9185-5.1817
1990-2009	Cardiovascular	Target median	37	0	2.9934	2.38	3.05	3.6137	0.71	5.25	1.0771	2.6464-3.3405
1990-2009	Cardiovascular	[Drug - target median]	37	1	1.5567	0.7825	1.745	2.53	-1.46	4.87	1.6463	1.0262-2.0872
1990-2009	Central nervous system	Drug	57	0	5.1234	4.12	5.04	6.02	2.13	8.29	1.357	4.7711-5.4757
1990-2009	Central nervous system	Target median	57	12	3.1544	2.74	3.23	3.38	2.02	3.95	0.95058	2.9076-3.4012
1990-2009	Central nervous system	[Drug - target median]	57	0	1.969	1.1137	1.83	2.8587	-0.61	4.48	1.2075	1.6556-2.2825
1990-2009	Gastrointestinal & metabolism	Drug	23	0	5.0872	3.0375	5.005	6.6988	1.51	10.38	2.3062	4.1447-6.0297
1990-2009	Gastrointestinal & metabolism	Target median	23	0	3.6035	2.3	4.61	4.91	0.82	6.815	1.8616	2.8427-4.3643
1990-2009	Gastrointestinal & metabolism	[Drug - target median]	23	0	1.4837	0.60875	1.39	2.295	-1.16	3.955	1.3369	0.93731-2.0301
1990-2009	Genito urinary	Drug	18	0	5.0414	3.145	5.22	6.705	1.415	10.44	2.3443	3.9584-6.1244
1990-2009	Genito urinary	Target median	18	0	3.0694	2.49	3.205	3.83	1.8	3.94	0.80471	2.6977-3.4412
1990-2009	Genito urinary	[Drug - target median]	18	0	1.9719	0.78	1.835	3.25	-1.075	6.53	1.8217	1.1304-2.8135
1990-2009	Infection	Drug	19	1	5.7166	3.7362	5.865	6.9075	2.78	7.85	2.2438	4.7076-6.7255
1990-2009	Infection	Target median	19	2	3.4813	2.8	3.61	3.61	2.22	3.64	1.0054	3.0292-3.9334
1990-2009	Infection	[Drug - target median]	19	0	2.2353	0.6325	2.06	3.4588	-0.29	6.265	1.7283	1.4581-3.0124
1990-2009	Multiple/other	Drug	10	0	3.981	2.11	3.68	5.08	1.26	7.555	2.0616	2.7032-5.2588
1990-2009	Multiple/other	Target median	10	0	2.663	2.31	2.6075	3.05	1.72	3.73	0.59257	2.2957-3.0303
1990-2009	Multiple/other	[Drug - target median]	10	0	1.318	0.145	1.34	2.145	-0.89	3.825	1.62	0.3139-2.3221
1990-2009	Musculo-skeletal	Drug	18	0	5.0292	2.77	4.48	7.765	1.995	8.74	2.3238	3.9556-6.1027
1990-2009	Musculo-skeletal	Target median	18	0	3.1364	2.31	2.4	4.81	1.41	4.81	1.3406	2.5171-3.7557
1990-2009	Musculo-skeletal	[Drug - target median]	18	0	1.8928	0.63	1.7525	3.04	-0.315	4.015	1.3655	1.2619-2.5236
1990-2009	Respiratory	Drug	20	2	4.1875	3.3	4.255	5.21	2.06	5.97	1.7203	3.4335-4.9415
1990-2009	Respiratory	Target median	20	3	2.3215	2.12	2.61	2.845	1.04	3.205	0.73223	2.0006-2.6424
1990-2009	Respiratory	[Drug - target median]	20	3	1.866	1.3525	2	2.59	-0.065	3.54	1.4416	1.2342-2.4978
1990-2009	Skin & eye	Drug	11	0	4.4536	2.1912	4.42	6.5025	1.23	8.4	2.5617	2.9398-5.9675
1990-2009	Skin & eye	Target median	11	0	2.8405	1.4837	2.2	4.1425	1.08	5.73	1.7278	1.8194-3.8615
1990-2009	Skin & eye	[Drug - target median]	11	0	1.6132	0.56375	2.35	2.7075	-0.97	3.155	1.5125	0.71937-2.507
2010-2020	Cancer	Drug	61	1	4.2826	3.2125	4.34	5.0675	0.625	7.3	1.4582	3.9167-4.6486
2010-2020	Cancer	Target median	61	2	3.1046	2.62	3.02	3.485	2.06	4.63	0.78401	2.9079-3.3014
2010-2020	Cancer	[Drug - target median]	61	1	1.178	0.43625	0.965	1.9725	-0.81	3.93	1.1999	0.87688-1.4791
2010-2020	Cardiovascular	Drug	12	0	4.8675	3.7175	4.9875	5.845	2.44	7.57	1.4507	4.0467-5.6883
2010-2020	Cardiovascular	Target median	12	1	3.2546	2.68	3.64	3.77	2.055	4.415	1.0732	2.6474-3.8618
2010-2020	Cardiovascular	[Drug - target median]	12	0	1.6129	0.69	1.5	2.59	-0.19	3.93	1.2081	0.92939-2.2964
2010-2020	Central nervous system	Drug	13	1	4.3735	4.0875	5.02	5.5175	1.995	6.08	1.8081	3.3906-5.3563
2010-2020	Central nervous system	Target median	13	0	3.2038	2.7763	3.37	3.6537	1.63	4.14	0.72898	2.8076-3.6001
2010-2020	Central nervous system	[Drug - target median]	13	0	1.1696	0.1675	1.445	2.2125	-1.99	2.94	1.3922	0.41279-1.9264
2010-2020	Gastrointestinal & metabolism	Drug	22	0	6.0761	4.84	6.795	7.72	1.89	8.095	1.9591	5.2575-6.8948
2010-2020	Gastrointestinal & metabolism	Target median	22	0	4.4314	3.3	4.99	5.66	0.45	5.66	1.4243	3.8362-5.0266
2010-2020	Gastrointestinal & metabolism	[Drug - target median]	22	0	1.6448	1.03	1.815	2.73	-0.425	3.105	1.1244	1.1749-2.1146
2010-2020	Genito urinary	Drug	4	0	5.3925	4.71	5.975	6.075	3.51	6.11	1.2577	4.1599-6.6251
2010-2020	Genito urinary	Target median	4	0	2.7237	1.87	2.5975	3.5775	1.87	3.83	1.0072	1.7367-3.7108
2010-2020	Genito urinary	[Drug - target median]	4	0	2.6687	1.86	2.4325	3.4775	1.64	4.17	1.1064	1.5845-3.753
2010-2020	Infection	Drug	8	0	5.9362	3.66	5.645	7.7425	2.475	10.92	2.8731	3.9453-7.9272
2010-2020	Infection	Target median	8	1	3.9731	3.3363	4.0313	4.23	2.575	4.23	1.0496	3.2458-4.7004
2010-2020	Infection	[Drug - target median]	8	1	1.9631	0.4325	1.59	2.62	-0.3175	2.975	2.2174	0.42655-3.4997
2010-2020	Multiple/other	Drug	11	0	5.1918	4.1287	5.435	6.1025	2.63	7.15	1.3458	4.3965-5.9871
2010-2020	Multiple/other	Target median	11	3	3.2923	2.9769	3.48	3.59	2.9175	3.92	0.9688	2.7197-3.8648
2010-2020	Multiple/other	[Drug - target median]	11	0	1.8995	0.86187	2.475	3.0394	-0.96	3.695	1.4702	1.0307-2.7684
2010-2020	Musculo-skeletal	Drug	3	0	3.4717	1.2763	2.36	5.945	0.915	7.14	3.258	-0.2151-7.1584
2010-2020	Musculo-skeletal	Target median	3	0	3.0033	2.4275	2.78	3.635	2.31	3.92	0.82791	2.0665-3.9402
2010-2020	Musculo-skeletal	[Drug - target median]	3	0	0.46833	-1.3863	0.05	2.4275	-1.865	3.22	2.5682	-2.4378-3.3745
2010-2020	Respiratory	Drug	7	0	3.555	2.3412	4.255	4.8888	0.835	5.165	1.6203	2.3547-4.7553
2010-2020	Respiratory	Target median	7	0	2.2529	1.33	2.61	3.0938	1.33	3.205	0.89003	1.5935-2.9122
2010-2020	Respiratory	[Drug - target median]	7	1	1.3021	0.94	1.645	1.8663	0.735	1.96	0.89285	0.64071-1.9636
