# Supplementary material for: Target-based evaluation of ‘drug-like’ properties and ligand efficiencies
Source: J Med Chem. Author manuscript; Available in PMC 2021 Jun 11. (PMC7610969; doi:10.1021/acs.jmedchem.1c00416)
Supplement: Supp manual curation_route of admin [file EMS123358-supplement-Supp_manual_curation_route_of_admin.pdf]

CMPD_PREF_NAME	Routes of admin added, not in CHEMBL_v26	Refs to route of admin not in CHEMBL_v26	All routes of administation
ABIRATERONE	Oral (as prodrug)	https://www.accessdata.fda.gov/drugsatfda_docs/label/2018/202379s024lbl.pdf	Oral
AGOMELATINE	Oral	https://www.ema.europa.eu/en/documents/product-information/valdoxan-epar-product-information_en.pdf	Oral
AMISULPRIDE	Oral	https://bnf.nice.org.uk/drug/amisulpride.html;https://www.accessdata.fda.gov/drugsatfda_docs/label/2020/209510s000lbl.pdf	Oral
AMSACRINE	iv	http://eci2012.net/wp-content/uploads/2015/03/Amsa-PM-En-100449.02-19Feb2015.pdf;https://bnf.nice.org.uk/drug/amsacrine.html	Parenteral
ATOSIBAN	iv	https://www.medicines.org.uk/emc/product/7056/smpc;https://bnf.nice.org.uk/drug/atosiban.html	Parenteral
BENPERIDOL	Oral	https://bnf.nice.org.uk/drug/benperidol.html	Oral
BENZBROMARONE	Oral	Patient Information - Benzbromarone (mymedicines.nz);https://www.sciencedirect.com/topics/neuroscience/benzbromarone	Oral
BEZAFIBRATE	Oral	https://www.drugs.com/international/bezafibrate.html;https://bnf.nice.org.uk/drug/bezafibrate.html	Oral
CARBENOXOLONE	Oral, topical	https://www.mims.com/malaysia/drug/info/carbenoxolone?mtype=generic;http://www.hpra.ie/img/uploaded/swedocuments/bd0b4df6-90dc-43ee-b447-ee9468dde049.pdf;https://www.medsafe.govt.nz/regulatory/ProductDetail.asp?ID=3283	Oral & topical
CARBETAPENTANE	Oral	https://www.drugs.com/dosage/carbetapentane.html;https://mri.cts-mrp.eu/human/downloads/DE_H_4538_001_PAR.pdf	Oral
CARBETOCIN	iv	https://bnf.nice.org.uk/drug/carbetocin.html	Parenteral
CINNARIZINE	Oral	https://bnf.nice.org.uk/drug/cinnarizine.html	Oral
CIPROFIBRATE	Oral	https://bnf.nice.org.uk/drug/ciprofibrate.html	Oral
CLEBOPRIDE	Oral, iv	https://www.mims.com/malaysia/drug/info/clebopride?mtype=generic;https://www.ema.europa.eu/en/documents/psusa/clebopride-list-nationally-authorised-medicinal-products-psusa/00000789/201906_en.pdf	Oral & parenteral
CLENBUTEROL	Oral, inhaled	https://www.healthline.com/health/clenbuterol;BNF version 68,72,76;PMID: 7002574	Oral & topical
CYPROTERONE ACETATE	Oral	https://www.drugs.com/mmx/cyproterone-acetate.html;https://bnf.nice.org.uk/drug/cyproterone-acetate.html;https://www.ebs.tga.gov.au/servlet/xmlmillr6?dbid=ebs/PublicHTML/pdfStore.nsf&docid=DD76B451A1C9CBC8CA258694003CBB3C&agid=(PrintDetailsPublic)&actionid=1	Oral
DEXIBUPROFEN	Oral	https://bnf.nice.org.uk/drug/dexibuprofen.html	Oral
DEXKETOPROFEN	Oral	https://bnf.nice.org.uk/drug/dexketoprofen.html;https://mri.cts-mrp.eu/Human/Product/Details/52090	Oral
DIMETHINDENE	Oral	https://mri.cts-mrp.eu/Human/Downloads/CZ_H_0686_001_FinalLabelling.pdf;https://www.gsk.com/en-gb/products/our-consumer-healthcare-products/skin-health/fenistil/;http://digicollection.org/hss/documents/h5780e/h5780e.pdf	Oral
ENALAPRILAT	Prodrug enalapril is oral, iv	https://bnf.nice.org.uk/drug/enalapril-maleate.html;https://www.accessdata.fda.gov/drugsatfda_docs/label/2010/075458s007lbl.pdf	Oral & parenteral
ESFLURBIPROFEN	Oral	https://bnf.nice.org.uk/drug/flurbiprofen.html	Oral
ESTRIOL	Topical	https://bnf.nice.org.uk/drug/estriol.html	Topical
ETHINYL ESTRADIOL	Prodrug mestranol is oral, topical	nyl Estradiol Dosage Guide with Precautions - Drugs.com;https://mri.cts-mrp.eu/Human/Downloads/NL_H_1902_001_FinalPI_4of4.pdf;https://dailymed.nlm.nih.gov/dailymed/drugInfo.cfm?setid=c6c8512d-b258-4be5-8c2f-0f1893f198b9;https://dailymed.nlm.nih.gov/dailymed/drugInfo.cfm?setid=068199a9-ef22-4061-bb76-1ee58f726	Oral, topical
ETORICOXIB	Oral	https://www.medicines.org.uk/emc/product/743/smpc;https://www.ebs.tga.gov.au/servlet/xmlmillr6?dbid=ebs/PublicHTML/pdfStore.nsf&docid=4B1ED46E7C437363CA2586A0000394DD&agid=(PrintDetailsPublic)&actionid=1;https://bnf.nice.org.uk/drug/etoricoxib.html	Oral
FENOTEROL	Oral, inhaled	https://www.mims.com/thailand/drug/info/fenoterol?mtype=generic;https://www.medsafe.govt.nz/regulatory/ProductDetail.asp?ID=4620;https://www.medsafe.govt.nz/regulatory/ProductDetail.asp?ID=1895;https://www.sciencedirect.com/topics/agricultural-and-biological-sciences/fenoterol	Oral & topical
FLUNITRAZEPAM	Oral, injection	https://www.eisai.com/news/news201703.html;https://www.ebs.tga.gov.au/servlet/xmlmillr6?dbid=ebs/PublicHTML/pdfStore.nsf&docid=3F02E9073B66F85CCA258694003CBE01&agid=(PrintDetailsPublic)&actionid=1	Oral & parenteral
IPRONIAZID	Oral	https://drugs.ncats.io/drug/D892HFI3XA;PMID: 14430381	Oral
LANINAMIVIR	Inhaled (from prodrug)	https://www.daiichisankyo.com/media_investors/media_relations/press_releases/detail/006497.html;https://www.pmda.go.jp/files/000211260.pdf	Topical
LASOFOXIFENE	Oral	https://www.ema.europa.eu/en/documents/assessment-report/fablyn-epar-public-assessment-report_en.pdf	Oral
LUMIRACOXIB	Oral	https://www.nps.org.au/radar/articles/lumiracoxib-prexige-for-osteoarthritis;https://www.medsafe.govt.nz/regulatory/ProductDetail.asp?ID=11860;https://www.ema.europa.eu/en/documents/referral/questions-answers-recommendation-withdraw-marketing-authorisations-lumiracoxib-containing-medicines_en.pdf	Oral
MELAGATRAN	Oral as prodrug, injection	https://drugs.ncats.io/drug/2A9QP32MD4;PMID: 14675083;https://www.sciencedirect.com/topics/pharmacology-toxicology-and-pharmaceutical-science/melagatran;https://www.ema.europa.eu/en/medicines/human/withdrawn-applications/ximelagatran-36-mg-film-coated-tablets	Oral & parenteral
MOFEZOLAC	Oral	https://drugs.ncats.io/drug/RVJ0BV3H3Y;http://digicollection.org/hss/en/d/Js2261e/2.html	Oral
MOXISYLYTE	Oral	https://bnf.nice.org.uk/drug/moxisylyte.html	Oral
MOXONIDINE	Oral	https://www.medicines.org.uk/emc/product/4138/smpc;https://bnf.nice.org.uk/drug/moxonidine.html;https://www.ebs.tga.gov.au/servlet/xmlmillr6?dbid=ebs/PublicHTML/pdfStore.nsf&docid=69034B37810F34B0CA2585D9004251FA&agid=(PrintDetailsPublic)&actionid=1	Oral
NOMIFENSINE	Oral	https://en.wikipedia.org/wiki/Nomifensine;PMID: 3907935;https://www.sciencedirect.com/topics/neuroscience/nomifensine	Oral
PHYSOSTIGMINE	iv	https://www.drugs.com/dosage/physostigmine.html;https://dailymed.nlm.nih.gov/dailymed/drugInfo.cfm?setid=a7d04b2c-fe34-480d-8f7c-7cbcf447a70b	Parenteral
PIRENZEPINE	Oral	https://www.medindia.net/doctors/drug_information/pirenzepine.htm;https://www.merseycare.nhs.uk/media/2179/pirenzepine-2014.pdf	Oral
PITOLISANT	Oral	https://bnf.nice.org.uk/drug/pitolisant.html	Oral
RALTITREXED	iv	https://bnf.nice.org.uk/drug/raltitrexed.html	Parenteral
REBOXETINE	Oral	https://bnf.nice.org.uk/drug/reboxetine.html	Oral
REMOXIPRIDE	Oral	https://drugs.ncats.io/drug/0223RD59PE;PMID: 11607043	Oral
RIMONABANT	Oral	https://www.drugbank.ca/drugs/DB06155;https://www.ema.europa.eu/en/documents/product-information/acomplia-epar-product-information_en.pdf	Oral
SERTINDOLE	Oral	https://www.mims.com/malaysia/drug/info/sertindole?mtype=generic;https://mri.cts-mrp.eu/Human/Downloads/DK_H_2931_001_FinalPL.pdf	Oral
SITAXENTAN	Oral	https://journals.sagepub.com/doi/full/10.4137/CMT.S2344;https://www.ema.europa.eu/en/documents/overview/thelin-epar-summary-public_en.pdf	Oral
TELOTRISTAT	Oral (from prodrug)	https://bnf.nice.org.uk/drug/telotristat-ethyl.html	Oral
TIVOZANIB	Oral	https://www.medicines.org.uk/emc/product/8996/smpc;PMID: 23449252;PMID: 10476617	Oral
TOLRESTAT	Oral	https://pubmed.ncbi.nlm.nih.gov/8681485/	Oral
VILDAGLIPTIN	Oral	https://bnf.nice.org.uk/drug/vildagliptin.html	Oral
DOMPERIDONE	Oral	https://bnf.nice.org.uk/drug/domperidone.html;https://www.ebs.tga.gov.au/servlet/xmlmillr6?dbid=ebs/PublicHTML/pdfStore.nsf&docid=29C44D9A26D476CACA2585E400422665&agid=(PrintDetailsPublic)&actionid=1	Oral
MELATONIN	Oral	https://www.ema.europa.eu/en/medicines/human/EPAR/circadin;https://www.ebs.tga.gov.au/servlet/xmlmillr6?dbid=ebs/PublicHTML/pdfStore.nsf&docid=D741005F34D8546FCA258673003CAB45&agid=(PrintDetailsPublic)&actionid=1	Oral
MIZOLASTINE	Oral	https://bnf.nice.org.uk/drug/mizolastine.html	Oral
MOCLOBEMIDE	Oral	https://bnf.nice.org.uk/drug/moclobemide.html;https://www.ebs.tga.gov.au/servlet/xmlmillr6?dbid=ebs/PublicHTML/pdfStore.nsf&docid=5D619F57F56B24A8CA258664003CABA8&agid=(PrintDetailsPublic)&actionid=1	Oral
OPICAPONE	Oral	https://www.accessdata.fda.gov/drugsatfda_docs/label/2020/212489s000lbl.pdf	Oral
OXYPURINOL	Oral	BNF releases 68/72/76	Oral
SULPIRIDE	Oral	https://bnf.nice.org.uk/drug/sulpiride.html	Oral
TOPOTECAN	Injection (parenteral), oral for certain patient groups	https://www.accessdata.fda.gov/drugsatfda_docs/label/2014/022453s002lbl.pdf;https://www.accessdata.fda.gov/drugsatfda_docs/label/2014/020981s006lbl.pdf;https://www.ema.europa.eu/en/documents/product-information/hycamtin-epar-product-information_en.pdf	Oral & parenteral
TRAMETINIB	Oral	https://www.accessdata.fda.gov/drugsatfda_docs/label/2013/204114s000lbl.pdf	Oral
ZANUBRUTINIB	Oral	https://www.accessdata.fda.gov/drugsatfda_docs/label/2019/213217s000lbl.pdf	Oral
UPADACITINIB	Oral	https://www.accessdata.fda.gov/drugsatfda_docs/label/2019/211675s000lbl.pdf	Oral
OZANIMOD	Oral	https://www.accessdata.fda.gov/drugsatfda_docs/label/2020/209899s000lbl.pdf	Oral
RIMEGEPANT	Oral	https://www.accessdata.fda.gov/drugsatfda_docs/label/2020/212728s000lbl.pdf	Oral
TIRABRUTINIB	Oral	https://www.pmda.go.jp/files/000235289.pdf;PMID: 32382949	Oral
VIBEGRON	Oral	https://www.pmda.go.jp/files/000235288.pdf,PMID: 30411311	Oral
OMARIGLIPTIN	Oral	https://www.pmda.go.jp/files/000229077.pdf;PMID: 26507988	Oral
TEPOTINIB	Oral	https://www.pmda.go.jp/files/000235289.pdf;PMID: 32361823	Oral
CAPMATINIB	Oral	https://www.accessdata.fda.gov/drugsatfda_docs/label/2020/213591s000lbl.pdf	Oral
EVOGLIPTIN	Oral	PMID: 26541763;PMID: 29727638	Oral
FEDRATINIB	Oral	https://www.accessdata.fda.gov/drugsatfda_docs/label/2019/212327s000lbl.pdf	Oral
OSILODROSTAT	Oral	https://www.accessdata.fda.gov/drugsatfda_docs/label/2020/212801s000lbl.pdf	Oral
PEFICITINIB	Oral	https://www.pmda.go.jp/files/000235288.pdf;PMID: 31093950	Oral
QUIZARTINIB	Oral	https://www.pmda.go.jp/files/000235289.pdf;https://www.daiichisankyo.com/media/press_release/detail/index_3179.html	Oral
NALFURAFINE	Oral	https://www.pmda.go.jp/files/000211074.pdf	Oral
TENELIGLIPTIN	Oral	https://www.pmda.go.jp/files/000153594.pdf	Oral
TOFOGLIFLOZIN	Oral	https://www.pmda.go.jp/files/000232771.pdf;PMID: 24848755	Oral
GOSOGLIPTIN	Oral	PMID: 31643671;NCT03088670;https://www.sciencedirect.com/topics/pharmacology-toxicology-and-pharmaceutical-science/diprotin-a	Oral
PEMAFIBRATE	Oral	https://www.pmda.go.jp/files/000226672.pdf;PMID: 28929345	Oral
TRELAGLIPTIN	Oral	https://www.pmda.go.jp/files/000213963.pdf;PMID: 26115728	Oral
ANAGLIPTIN	Oral	https://www.pmda.go.jp/files/000218646.pdf	Oral
IPRAGLIFLOZIN	Oral	https://www.pmda.go.jp/files/000206796.pdf;PMID: 24668021	Oral
RELUGOLIX	Oral	https://www.pmda.go.jp/files/000235288.pdf;PMID: 30937733	Oral
ASUNAPREVIR	Oral	https://www.pmda.go.jp/files/000209023.pdf	Oral
SELUMETINIB	Oral	https://www.accessdata.fda.gov/drugsatfda_docs/label/2020/213756s000lbl.pdf	Oral
ISTRADEFYLLINE	Oral	https://www.accessdata.fda.gov/drugsatfda_docs/label/2019/022075s000lbl.pdf;https://www.pmda.go.jp/files/000153870.pdf	Oral
RUPATADINE	Oral	https://www.pmda.go.jp/files/000232769.pdf;https://mri.cts-mrp.eu/Human/Downloads/ES_H_0105_001_FinalLabelling_1of2.pdf;PMID: 31231589	Oral
MOZAVAPTAN	Oral	https://www.pmda.go.jp/files/000153730.pdf	Oral
BLONANSERIN	Oral	https://www.pmda.go.jp/files/000152974.pdf;PMID: 31471246	Oral
MINODRONIC ACID	Oral	https://www.pmda.go.jp/files/000232775.pdf;PMID: 23440003	Oral
RAMATROBAN	Oral	https://www.pmda.go.jp/files/000153255.pdf;https://pubmed.ncbi.nlm.nih.gov/12649388/;https://www.kegg.jp/entry/D01128	Oral
PRANLUKAST	Oral	PMID: 12699401	Oral
SERATRODAST	Oral	PMID: 9357394;https://www.takeda.com/newsroom/newsreleases/2014/towards-optimization-of-global-production-network/;https://jglobal.jst.go.jp/en/detail?JGLOBAL_ID=200902122515527346	Oral
EBASTINE	Oral	https://mri.cts-mrp.eu/Human/Downloads/DK_H_2694_001_FinalPL.pdf	Oral
FADROZOLE	Oral	PMID: 7487133;PMID: 12488294	Oral
VOGLIBOSE	Oral	https://www.kissei.co.jp/e_contents/news/2011/20110422-669.html;PMID: 24843762;PMID: 24798092	Oral
EPALRESTAT	Oral	PMID: 16801576;PMID: 31636082	Oral
TAMIBAROTENE	Oral	https://www.pmda.go.jp/files/000153119.pdf	Oral
ENTRECTINIB	Oral	https://www.accessdata.fda.gov/drugsatfda_docs/label/2019/212725s000lbl.pdf	Oral
OZAGREL	Oral, parenteral	https://www.kissei.co.jp/e_contents/relation/annual_report/uploaded/kissei_ar2008.pdf;https://www.ono-pharma.com/sites/default/files/en/news/press/sm_cn181001.pdf	Oral & parenteral
NAFTOPIDIL	Oral	https://www.asahi-kasei.com/news/2020/e200602.html;PMID: 30306544	Oral
RILMENIDINE	Oral	https://mri.cts-mrp.eu/Human/Downloads/FR_H_0460_001_FinalLabelling.pdf	Oral
IBUDILAST	Oral, topical	https://www.kyorin-pharm.co.jp/en/news/docs/ms.pdf;PMID: 19032723;https://www.rad-ar.or.jp/siori/english/kekka.cgi?n=1744;PMID: 21636966	Oral & topical
TOLOXATONE	Oral	http://agence-prd.ansm.sante.fr/php/ecodex/extrait.php?specid=66760573	Oral
OLMUTINIB	Oral	PMID: 27357069;https://doi.org/10.1093/annonc/mdx671.001	Oral
LOXOPROFEN	Oral	https://www.pmda.go.jp/files/000153119.pdf	Oral
ROXATIDINE ACETATE	Oral	PMID: 1717223;PMID: 22293541	Oral
IMIDAPRIL	Oral	PMID: 8592249;PMID: 17547476;https://bnf.nice.org.uk/drug/imidapril-hydrochloride.html	Oral
LUMATEPERONE	Oral	https://www.accessdata.fda.gov/drugsatfda_docs/label/2019/209500s000lbl.pdf	Oral
